# Molecular Networking-Guided Phytochemical Profiling and Anti-Inflammatory Evaluation of Honglanqi, an Underutilized Commercial Specification of Astragali Radix

**DOI:** 10.3390/plants15101442

**Published:** 2026-05-08

**Authors:** Xiangmei Tan, Aoao Wang, Hongmei Li, Minzhen Yin, Huasheng Peng

**Affiliations:** 1 State Key Laboratory for Quality Ensurance and Sustainable Use of Dao-di Herbs, National Resource Center for Chinese Materia Medica, China Academy of Chinese Medical Sciences, Beijing 100700, China; xiangmei_t129@163.com; 2 Institute of Chinese Materia Medica, China Academy of Chinese Medical Sciences, Beijing 100700, China; aawang@icmm.ac.cn (A.W.); hmli@icmm.ac.cn (H.L.); 3 Key Scientific Research Base for Traditional Chinese Medicine Heritage (Institute of Chinese Materia Medica, China Academy of Chinese Medical Sciences), National Cultural Heritage Administration, Beijing 100700, China

**Keywords:** Astragali Radix, Honglanqi, chemical profiling, feature-based molecular networking, novel compounds, anti-inflammatory

## Abstract

Honglanqi (HLQ), a distinctive commercial specification of Astragali Radix (AR) characterized by rhytidome and interxylary cork, remains undervalued despite originating from premium long-cultivated material. The ethyl acetate fraction (HLQE) showed the greatest anti-inflammatory activity among the polarity-based fractions of an HLQ ethanol extract. We applied an integrated ultra-high performance liquid chromatography–quadrupole time-of-flight tandem mass spectrometry (UPLC–QTOF-MS/MS) and feature-based molecular networking (FBMN) strategy to systematically profile the chemical composition of HLQE. A total of 102 metabolites were annotated across seven compound classes, of which 18 were predicted as potential new compounds, and 37 were reported in AR for the first time. Three undescribed compounds, astragalinin A, astragalinin B, and astragquinone, were isolated and characterized, together with 19 known compounds. In LPS-stimulated RAW264.7 macrophages, astragalinin A exhibited potent inhibition of TNF-α and IL-6 (IC_50_ < 10 μM). Astragquinone showed significant TNF-α inhibition with a favourable safety profile. Stereochemical configuration critically influenced anti-inflammatory potency, as demonstrated by the enantiomeric pair. Molecular docking predicted favourable binding orientations toward TNF-α and IL-6 in silico. These findings provide a comprehensive phytochemical and bioactivity profile of HLQ, supporting its value-added utilization as an underutilized AR resource.

## 1. Introduction

Astragali Radix (AR, Huangqi in Chinese) is a traditional Chinese medicine widely used in herbal medicine, functional foods, and the nutraceutical industry worldwide [1,2]. It is derived from the dried roots of *Astragalus membranaceus* (Fisch.) Bge. var. *mongholicus* (Bge.) Hsiao or *Astragalus membranaceus* (Fisch.) Bge. With a documented medicinal history spanning over two millennia, AR is officially listed in the pharmacopoeias of China, Japan, Korea, and the United States [3]. Modern pharmacological studies have demonstrated that AR exhibits a broad spectrum of biological activities, including immunomodulatory, anti-inflammatory, antioxidant, and cardiovascular protective effects. These activities are primarily attributed to its rich content of flavonoids, triterpenoid saponins, and polysaccharides [4]. AR production has expanded substantially in recent years, rising from approximately 64,400 tonnes in 2019 to 85,200 tonnes in 2024 [5]. Export volumes reached 3924 tonnes, valued at approximately 21.78 million USD in 2025 [6]. In this context, systematic characterization and rational grading of diverse AR commercial specifications have become increasingly important for optimizing resource utilization, improving grading systems, and enhancing economic value.

The primary source species in the commercial AR market is *A. membranaceus* var. *mongholicus* (AMM), which is mainly distributed in northern and northwestern China, with Gansu, Inner Mongolia, and Shanxi provinces serving as the major production regions [7]. Among current commercial specifications, wild-simulated AR (WAR) involves direct seed sowing and natural growth for more than six years. It is widely regarded as therapeutically superior and commands significantly higher market prices than fast-growing cultivated AR (CAR), which involves transplantation after seedling emergence and is typically harvested within two to three years [8,9]. However, during the production and grading of WAR, a considerable proportion of roots develop distinctive morphological features, including rhytidome and interxylary cork. They are commonly referred to as “rotten skin” and “rotten heart”, respectively. Rhytidome refers to the outermost bark layer composed of dead periderm tissue, while interxylary cork refers to secondary cork formed within the xylem. Both features are associated with programmed cell death (PCD), a genetically regulated process of cellular self-degradation [10]. These roots are routinely separated from standard-grade products and categorized as Honglanqi (HLQ), a special commercial specification that is often undervalued or discarded in industrial supply chains [11].

This grading practice leads to inefficient utilization of valuable plant resources, particularly considering that HLQ originates from long-cultivated, high-input materials with potentially unique metabolic profiles. Increasing evidence suggests that such prolonged cultivation-associated stress may promote the accumulation of defense-related secondary metabolites, especially flavonoids [12]. Preliminary analyses indicate that HLQ is enriched in flavonoids but contains relatively lower levels of certain saponins compared to standard AR [11], supporting a stress-adapted metabolic shift linked to plant maturation [9]. These findings suggest that HLQ should be reconsidered as a valuable resource rather than a low-quality by-product. However, the lack of systematic chemical characterization has hindered its rational grading, quality assessment, and value-added utilization. The systematic characterization of such underutilized commercial specifications holds broader significance for the medicinal plant research community. It provides a methodological framework for uncovering chemically distinct materials overlooked by morphology-based grading systems. Moreover, it offers new opportunities for the discovery of novel lead compounds with therapeutic potential. Meeting this need requires analytical strategies for comprehensive profiling of complex phytochemical matrices. Ultra-high performance liquid chromatography–quadrupole time-of-flight tandem mass spectrometry (UPLC–QTOF-MS/MS) provides high sensitivity and resolution for metabolite detection and structural annotation [13]. Feature-based molecular networking (FBMN) enables systematic annotation of structurally diverse metabolite families and improved isomer discrimination within complex matrices [14,15]. The integration of these approaches offers an efficient platform for the comprehensive characterization of medicinal resources, supporting the rational grading and value-added utilization of underexplored AR commercial specifications, exemplified by HLQ.

In this study, the ethanol extract of HLQ was partitioned into polarity-based fractions, and their anti-inflammatory activities were comparatively evaluated. UPLC–QTOF-MS/MS combined with FBMN was then employed to systematically profile the chemical constituents of the most active fraction. Representative constituents were further isolated and structurally elucidated. Their anti-inflammatory activities were then evaluated in LPS-stimulated RAW 264.7 macrophages. Molecular docking was performed to preliminarily explore potential ligand–target interactions. These findings provide new insights into the chemical basis underlying the anti-inflammatory activity of HLQ and support its rational grading and value-added utilization within the AR production chain.

## 2. Results and Discussion

### 2.1. Comparative Anti-Inflammatory Evaluation of HLQ Fractions

To determine the distribution of anti-inflammatory activity across fractions of different polarities, the ethanol extract of HLQ was partitioned into petroleum ether (HLQP), ethyl acetate (HLQE), and aqueous (HLQW) fractions, which were comparatively evaluated in LPS-stimulated RAW 264.7 macrophages. Before anti-inflammatory evaluation, cytotoxicity of all three fractions was systematically assessed across a seven-point concentration range (200, 100, 50, 10, 5, 1, and 0.1 μg/mL) using the CCK-8 assay. The results demonstrated that concentrations ≥ 50 μg/mL exhibited significant cytotoxicity across all fractions, while concentrations ≤ 10 μg/mL were confirmed to be non-cytotoxic (Figure S1 and Table S1). Accordingly, 10 μg/mL was selected as the highest non-cytotoxic concentration common to all three fractions for comparative anti-inflammatory screening. This concentration maximized detection sensitivity while avoiding cytotoxicity-induced confounding effects. Among the three fractions evaluated, HLQE showed the greatest inhibition rates of TNF-α (52.2 ± 7.8%) and IL-6 (55.6 ± 3.6%) at the tested concentration (10 μg/mL), whereas HLQP and HLQW exhibited markedly weaker or negligible activity (Table S1). The pronounced activity observed in the medium-polarity fraction suggests enrichment of anti-inflammatory constituents in HLQE. This is likely attributable to the concentration of flavonoids known to exert anti-inflammatory effects in AR [16]. Based on these results, HLQE was selected for subsequent phytochemical investigation and functional evaluation of representative constituents.

### 2.2. UPLC–QTOF-MS/MS Profiling of HLQE

**Figure 1:**
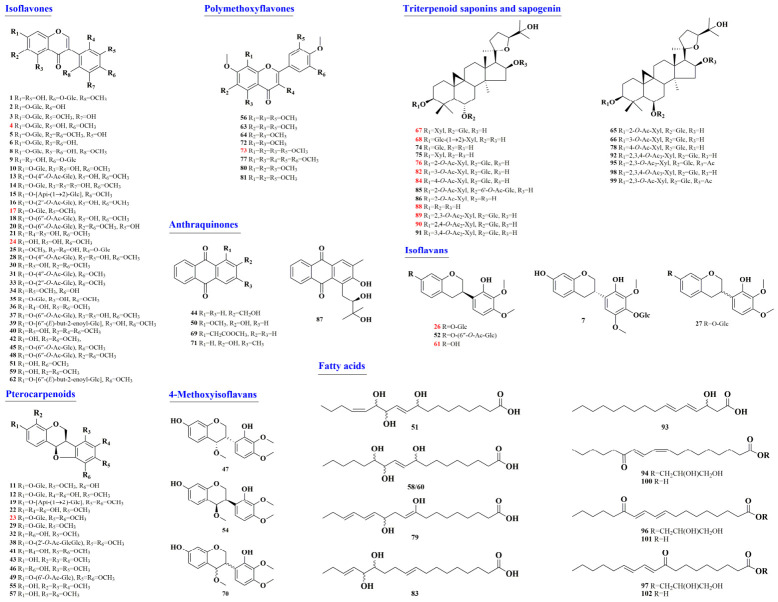
Proposed chemical characterization of the ethyl acetate extract of Honglanqi (HLQE). The red means are identified by comparison with reference standards. glu: glucosyl; xyl: xylosyl; Api: apiosyl.

HLQE was profiled using UPLC–QTOF-MS/MS in positive ion mode (Figure S2). An in-house AR compound library (Table S2) was constructed through literature mining and database searching, and metabolite annotation was performed via FBMN on the GNPS platform [17]. Reference standards and ClogP-assisted retention time analysis were employed to facilitate compound identification [18]. A total of 102 metabolites were characterized, comprising 35 isoflavones, 14 pterocarpenoids, 20 triterpenoid saponins, 8 isoflavans, 8 polymethoxyflavones, 5 anthraquinones, and 12 fatty acids (Figure 1 and Table S3). Among them, 16 were unambiguously confirmed using authentic standards, 68 were putatively annotated based on MS/MS spectral matching and fragmentation pattern analysis, and 18 were proposed as potential novel compounds. The chemical composition of HLQE was consistent with previous reports, which identified flavonoids and triterpenoids as the predominant constituent classes of AR [19]. Notably, flavonoid-type metabolites accounted for 65 of the 102 characterized compounds, indicating that HLQ represents a flavonoid-enriched commercial specification. This finding supports its potential as a value-added functional resource.

### 2.3. Feature-Based Molecular Networking (FBMN) Aided the Identification of Metabolites in HLQE

**Figure 2:**
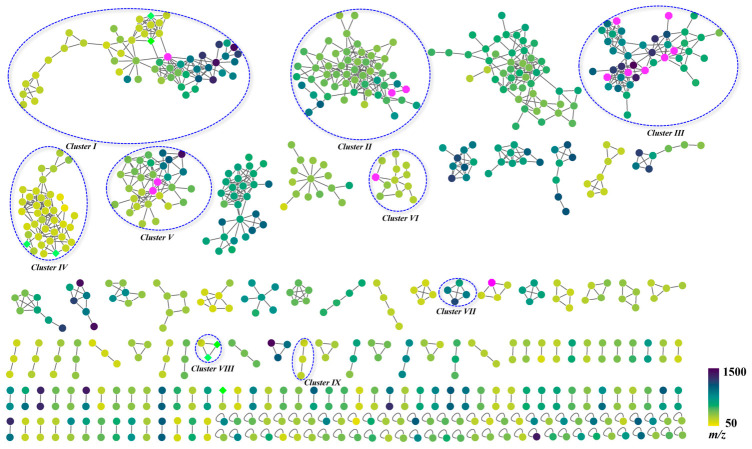
Molecular networks based on mass spectrometry data in positive ion mode of the ethyl acetate extract of Honglanqi (HLQE). Cluster I: pterocarpenoids and 4-methoxyisoflavans; II: isoflavone-*O*-glycosides; III: triterpenoid saponins and sapogenin; IV: fatty acids; V: isoflavans; VI: polymethoxyflavones; VII and IX: isoflavones; VIII: anthraquinones. (The node color corresponds to the precursor mass value, with pink octagons representing compounds matched to reference standards and green diamonds representing those that were isolated and identified).

FBMN facilitates the discovery of new natural products and complex metabolites [14]. In this approach, structurally similar compounds generate comparable MS/MS fragments and therefore cluster together. In this study, a comprehensive FBMN of HLQE was constructed using GNPS based on MS/MS spectral similarity. The initial GNPS analysis yielded a raw network of 2739 precursor ions. To focus on major chemical families and improve network interpretability, the data were filtered to remove background noise and uninformative singletons. This refined network comprised 707 significant precursor ions, organized into 122 clusters (nodes ≥ 2) and 64 prioritized single nodes (Figure 2). The resulting network provided a clear and structured visualization of the chemical space. By automated spectral library matching, 65 nodes were annotated, corresponding to a match rate of 9.2% relative to the 707 retained features. Nine major clusters were characterized, including isoflavones (II, VII, and IX), pterocarpenoids and 4-methoxyisoflavans (I), triterpenoid saponins and sapogenins (III), isoflavans (V), polymethoxyflavones (VI), anthraquinones (VIII), and fatty acids (IV). Each cluster was distinguishable by its characteristic MS/MS fragmentation patterns. After integrating in-house databases, reference standards, literature data, and FBMN spectral analysis, a total of 102 compounds were identified (Figure 1 and Table S3). Notably, 18 candidates were prioritized as potential novel analogues by integrating molecular networking with diagnostic MS/MS fragment analysis. These candidates demonstrated structural affiliation by co-clustering with known constituents or sharing class-specific fragment ions. However, no matches were found in the searched databases (GNPS and SciFinder). Their structures were therefore tentatively assigned as new derivatives, including putative glycosylated analogues, based on systematic mass shifts relative to known standards. The proposed fragmentation pathways and detailed structural characterizations are provided in Figure S4 and Table S3.

#### 2.3.1. Isoflavones

Integration of FBMN with HR–MS/MS analysis enabled the identification of 35 isoflavones, constituting the predominant molecular clusters (II, VII, and IX) in the FBMN (Figure 2 and Figure S5). These compounds were mainly assigned as isoflavone-*O*-glycosides and their corresponding aglycones. Isoflavone-*O*-glycosides are primarily glycosylated at the 7-OH or 4′-OH positions, with representative compounds including calycosin-7-*O*-glucoside and ononin. They exhibited characteristic neutral losses of sugar-related moieties, including glucosyl (162 Da), acetylglucosyl (204 Da), (*E*)-but-2-enoylglucosyl (230 Da), and apiosylglucosyl (294 Da), yielding corresponding aglycone ions. Isoflavone aglycones were mainly distributed in clusters VII and IX. They underwent sequential demethylation, dehydration, CO loss, and Retro-Diels-Alder (RDA) cleavage, generating diagnostic ^1,3^A^+^ and ^1,3^B^+^-15 fragment ions that supported reliable structural annotation within the molecular network [19].

**Figure 3:**
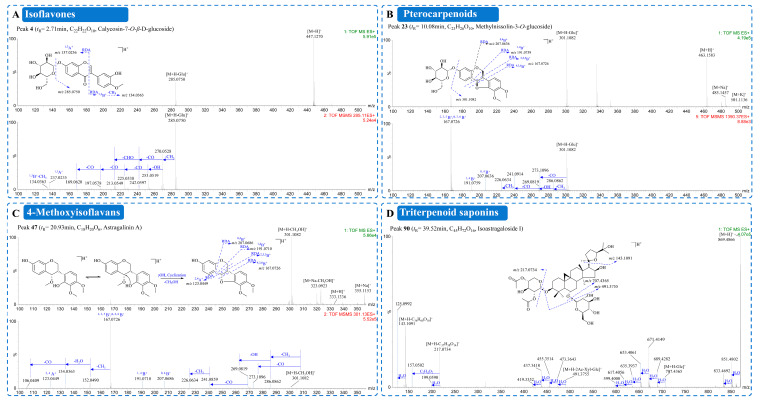
MS/MS spectra and proposed fragmentation pathways of representative compounds from the ethyl acetate fraction of Honglanqi (HLQE). (**A**–**D**) Representative positive MS^E^ spectra and fragmentation schemes for (**A**) isoflavones (e.g., peak **4**), (**B**) pterocarpenoids (e.g., peak **23**), (**C**) 4-methoxyisoflavans (e.g., peak **47**), and (**D**) triterpenoids (e.g., peak **90**).

Peak **4** (t_R_ = 2.71 min, *m*/*z* 447.1270 [M+H]^+^, C_22_H_22_O_10_), confirmed against an authentic standard, was selected as a representative anchor compound to establish the diagnostic fragmentation framework (Figure 3A). The MS1 spectrum displayed a protonated molecular ion at *m*/*z* 447.1270 [M+H]^+^. A diagnostic aglycone ion at *m*/*z* 285.0750 [M+H-Glc]^+^ was observed via neutral loss of glucosyl (162 Da). MS2 fragmentation of *m*/*z* 285.0750 revealed two principal pathways. The first involved sequential losses of CH_3_, CO, and CHO, yielding ions at *m*/*z* 270.0527, 242.0597, and 213.0549. The second was an alternative pathway involving losses of CH_3_, OH, and consecutive CO units, generating ions at *m*/*z* 270.0527, 253.0519, 225.0538, 197.0578, and 169.0620. Concurrent RDA cleavage of the C-ring produced a ^1,3^A^+^ ion at *m*/*z* 137.0236, indicative of A-ring hydroxyl substitution, and a ^1,3^B^+^-15 ion at *m*/*z* 134.0363, indicative of hydroxyl and methoxyl substitution on the B-ring. Together, these ions established a reliable fragmentation reference for cluster-based annotation. Peak **4** was thus identified as calycosin-7-*O*-*β*-D-glucoside. Peaks **17** (t_R_ = 7.42 min, *m*/*z* 431.1368 [M+H]^+^, C_22_H_22_O_9_) and **24** (t_R_ = 7.42 min, *m*/*z* 285.0750 [M+H]^+^, C_16_H_12_O_5_), both confirmed against authentic standards, served as additional anchor nodes for clusters II and IX, respectively. Peak **17** yielded RDA fragment ions at *m*/*z* 137.0235 (^1,3^A^+^) and *m*/*z* 118.0369 (^1,3^B^+^-15) and was identified as ononin, while peak **24** was identified as calycosin.

Guided by these validated fragmentation rules, FBMN topology, and reported MS/MS fragmentation data [19,20,21], the remaining isoflavones were systematically annotated according to their respective structural subtypes. Glucoside-type isoflavones were characterized by a neutral loss of 162 Da, yielding the corresponding aglycone ions. Peak **10** (t_R_ = 4.90 min, *m*/*z* 463.1280 [M+H]^+^) was tentatively assigned as pratensein-7-*O*-*β*-D-glucopyranoside. This assignment was based on its aglycone ion at *m*/*z* 301.0711 and a ^1,3^A^+^ ion at *m*/*z* 153.0184, indicative of an additional A-ring hydroxyl substituent relative to peak **4**. Peaks **1**, **8**, and **25** were tentatively assigned as isomers of peak **10** based on identical precursor masses but distinct retention times and ClogP values. Among them, peak **8** showed no match in GNPS or SciFinder and was tentatively proposed as a novel isoflavone-*O*-glycoside derivative. Similarly, peak **14** (t_R_ = 5.86 min, *m*/*z* 479.1175 [M+H]^+^), generating an aglycone ion at *m*/*z* 317.0659 and a ^1,3^A^+^ ion at *m*/*z* 153.0184. It was tentatively assigned as another potentially novel derivative. Peaks **6** and **9**, sharing precursor ions at *m*/*z* 433.1123 [M+H]^+^, were tentatively assigned as genistin and sophoricoside based on their diagnostic RDA fragments and literature comparison.

Acetylated derivatives (+42 Da) included peaks **13**, **18**, **31**, and **45**. Peaks **16**, **28**, and **33** shared precursor masses and aglycone ions with known analogues but displayed retention behaviors inconsistent with reported isomers, suggesting that they may represent novel acetylated isoflavone derivatives. (*E*)-But-2-enoyl- and apiosylglucoside-type derivatives, characterized by mass increments of +68 Da and +132 Da, respectively, were tentatively assigned and represented by peaks **39**, **62**, and **15**. Isoflavone aglycones, including odoratin (peak **30**) and afrormosin (peak **59**), were tentatively assigned based on their diagnostic RDA fragment ions and literature comparison. Seven additional isoflavones were independently annotated through in-house library matching and published MS/MS data. In total, 35 isoflavones were characterized, including five tentatively proposed novel derivatives (peaks **8**, **14**, **16**, **28**, and **33**), with detailed fragmentation pathways provided in Figure S4.

#### 2.3.2. Pterocarpenoids and 4-Methoxyisoflavans

Pterocarpenoids and 4-methoxyisoflavans colocalized within a single, well-defined molecular cluster (Cluster I) in the FBMN (Figure 2 and Figure S6), reflecting their shared structural core. Pterocarpenoids are characterized by a tetracyclic skeleton bridged at C-6a and C-11a. In contrast, 4-methoxyisoflavans are biosynthetic precursors that can undergo cyclization under acidic or MS-induced conditions to form pterocarpan-like structures [22]. Structural annotation of this cluster relied on common diagnostic MS/MS features, including glycosidic bond cleavage, RDA fragmentation, and characteristic neutral losses of H_2_O, CO, and CH_3_ [19].

**Figure 4:**
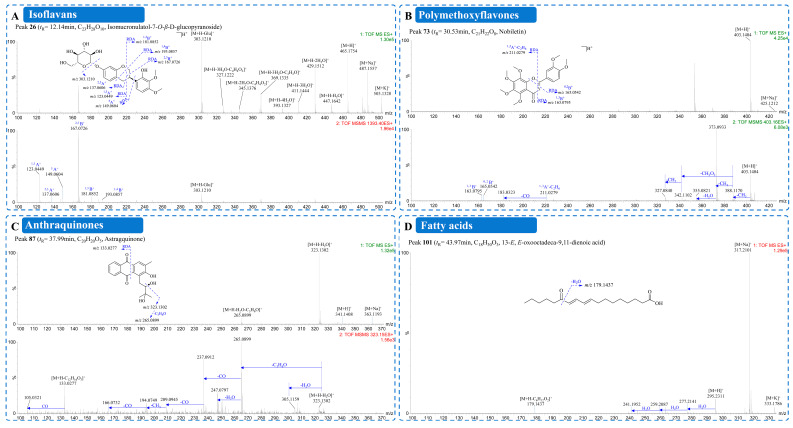
MS/MS spectra and proposed fragmentation pathways of representative compounds from the ethyl acetate fraction of Honglanqi (HLQE). (**A**–**D**) Representative positive MS^E^ spectra and fragmentation schemes for (**A**) isoflavans (e.g., peak **26**), (**B**) polymethoxyflavones (e.g., peak **73**), (**C**) anthraquinones (e.g., peak **87**), and (**D**) fatty acids (e.g., peak **101**).

The diagnostic fragmentation pattern was rigorously validated using the reference standard (-)-methylnissolin-3-*O*-glucoside (peak **23**) (Figure 3B). Its protonated molecular ion (t_R_ = 10.08 min, *m*/*z* 463.1583 [M+H]^+^, C_23_H_26_O_10_) initially lost a glucosyl moiety (162 Da) to yield the aglycone ion at *m*/*z* 301.1082. The aglycone then underwent characteristic RDA cleavage, generating a base peak at *m*/*z* 167.0726 (^2,3,5^B^+^/^4,3,6^B^+^) and diagnostic low-mass ions at *m*/*z* 191.0759 (^1,4^B^+^) and *m*/*z* 207.0637 (^0,4^B^+^). These fragments collectively established the diagnostic fingerprint of the methylnissolin-type skeleton. This fingerprint provided direct structural evidence for peak **47** (t_R_ = 20.93 min, *m*/*z* 355.1153 [M+Na]^+^, C_18_H_20_O_6_), which initially underwent a neutral loss of CH_3_OH (32 Da) to generate an ion at *m*/*z* 301.1082. The MS^2^ spectrum of this product ion was virtually indistinguishable from that of the aglycone of peak **23** (Figure 4B,C). This observation supports a demethoxylation–cyclization mechanism. Elimination of the labile 4-methoxy group triggers intramolecular cyclization with the B-ring hydroxyl, forming a pseudopterocarpan structure analogous to authentic pterocarpans [22]. Peak **47** was thus tentatively assigned as a novel 4-methoxyisoflavan.

Guided by the established fragmentation rules, FBMN topology, and literature MS/MS data, the remaining compounds in cluster I were systematically annotated based on characteristic mass shifts [19,20,23]. Among the 14 pterocarpenoids characterized, peaks **12** and **22** showed no match in GNPS or SciFinder and were tentatively proposed as novel pterocarpan derivatives. Peak **38** displayed fragmentation patterns highly similar to those of a known analogue but with a distinct retention time, and was therefore proposed as a novel isomeric derivative. Among 4-methoxyisoflavans, peaks **54** and **70** shared the same precursor ion at *m*/*z* 355.1153 [M+Na]^+^ as peak **47** and displayed identical demethoxylation–cyclization fragmentation. All three showed no database records and were therefore established as previously unreported derivatives. Peaks **47** and **54** were subsequently confirmed by isolation and full spectroscopic characterization (Section 2.4). In total, 14 pterocarpenoids and 3 novel 4-methoxyisoflavans (peaks **47**, **54**, and **70**) were characterized, with fragmentation pathways provided in Figure S4.

#### 2.3.3. Triterpenoid Saponins

Triterpenoid saponins, particularly cycloastragenol-type glycosides, are considered the principal bioactive constituents of AR. These compounds are characterized by a conserved cycloastragenol aglycone core that features a distinctive 9,19-cyclopropane ring and a 20,24-epoxy (tetrahydrofuran) side chain. Structural diversity arises from glycosylation at the C-3, C-6, and C-25 hydroxyl positions with glucose and xylose. This generates mono- to trisaccharide chains that are frequently decorated with acetyl substitutions [24,25].

A total of 20 triterpenoids were characterized in HLQE, predominantly belonging to the cycloastragenol-type glycoside class (Figure 2 and Figure S7). Identification was based on diagnostic fragmentation patterns in positive ion mode. They were dominated by sequential glycosidic bond cleavage, including neutral losses of 132 Da for pentosyl, 162 Da for hexosyl, and 174/216 Da for acetylated pentosyl units, followed by successive H_2_O (18 Da) losses from the aglycone core. The fragmentation mechanism was exemplified by isoastragaloside I (peak **90**), which is a representative diglycosylated saponin (Figure 4D). Its protonated molecular ion ([M+H]^+^ at *m*/*z* 869.4866, C_45_H_73_O_16_) first lost an outer glucose unit (162 Da) to yield a prominent ion at *m*/*z* 707.4365, followed by cleavage of a diacetylxylose moiety (216 Da) to generate the diagnostic cycloastragenol aglycone ion at *m*/*z* 491.3755 ([M+H-Glc-DiAcXyl]^+^). This aglycone ion further underwent characteristic neutral losses of H_2_O and cleavage of the oxygenated side chain, producing signature low-mass fragment ions at *m*/*z* 143.1091 and 125.0992. This fragmentation pathway was confirmed by comparison with an authentic reference standard.

Guided by this established fragmentation pattern and literature MS/MS data, the remaining saponins in cluster III were systematically annotated [19,26]. Key benchmarks, including astragaloside IV (peak **67**), astragaloside II (peak **76**), and the aglycone cycloastragenol (peak **88**), were unambiguously confirmed against reference standards to anchor annotation confidence. Acetylated derivatives were tentatively assigned by a +42 Da mass increment relative to known precursors, exemplified by acetylastragaloside I (peak **98**) and trojanoside I (peak **95**). Peaks **66**, **92**, and **99** displayed fragmentation patterns consistent with the cycloastragenol-type skeleton but showed no match in GNPS or SciFinder. They were therefore tentatively proposed as novel triterpenoid saponin derivatives. In total, 20 triterpenoids were characterized, including 3 tentatively proposed novel derivatives (peaks **66**, **92**, and **99**), with fragmentation pathways provided in Figure S4.

#### 2.3.4. Isoflavans

Isoflavans, characterized by a 3-phenylchroman skeleton, were identified as a minor chemical class in the HLQE (Figure 2 and Figure S8). Their MS/MS behavior resembles that of isoflavones, with glycosides readily losing glucosyl or acetylglucosyl moieties in MS1, and the C-ring undergoing characteristic RDA cleavage in MS2 to generate diagnostic ions including ^1,3^A^+^, ^2,3^B^+^, ^1,4^B^+^, and ^5^A^+^ [19,27]. Using these diagnostic features and FBMN topology, isomucronulatol-7-*O*-*β*-D-glucopyranoside (peak **26**) and isomucronulatol (peak **61**) were confirmed against authentic standards and served as anchor compounds (Figure 4A). Peak **7** (*m*/*z* 481.1690 [M+H]^+^) showed a diagnostic glucosyl loss yielding an ion at *m*/*z* 319.1187. RDA fragments at *m*/*z* 123.0449 (^1,3^A^+^) and *m*/*z* 183.0657 (^2,3^B^+^) were indicative of one A-ring hydroxyl and two hydroxyls with two methoxyls on the B-ring. Peak **7** was therefore tentatively identified as astraflavonoid C. Peak **52** showed a +42 Da mass increment relative to peak **26** and was tentatively assigned as an acetylated derivative. Peak **27** (*m*/*z* 465.1754 [M+H]^+^) displayed a fragmentation pattern nearly identical to that of peak **26** but showed no match in GNPS or SciFinder. It was therefore tentatively proposed as a novel isoflavan derivative. In total, 5 isoflavans were characterized, including one tentatively proposed novel derivative (peak **27**), with fragmentation pathways provided in Figure S4.

#### 2.3.5. Polymethoxyflavones

Polymethoxyflavones (PMFs) are flavonoids with multiple methoxy groups on the benzo-*γ*-pyrone skeleton and exhibit distinctive mass spectrometric behavior. In positive ion mode, PMFs typically undergo stepwise demethylation ([M+H-nCH_3_]^+^) and RDA cleavage of the C-ring, generating diagnostic fragment ions [28].

A total of eight PMFs were detected with the aid of FBMN (Figure 2 and Figure S9), including isosinensetin (peak **56**), sinensetin (peak **63**), 5,6,7,4′-tetramethoxyflavone (peak **64**), 5,7,8,4′-tetramethoxyflavone (peak **72**), nobiletin (peak **73**), 3,5,6,7,8,3′,4′-heptamethoxyflavone (peak **77**), tangeretin (peak **80**), and 5-hydroxy-3,6,7,8,3′,4′-hexamethoxyflavone (peak **81**) [29]. Nobiletin (peak **73**), which was confirmed with an authentic reference standard, served as the representative anchor compound (Figure 4B). Its MS/MS spectrum was dominated by stepwise demethylation and characteristic RDA fragments, establishing a diagnostic fingerprint for PMF annotation. Guided by this fingerprint, peaks **56**, **63**, and **80** showed precursor ions 30 Da lower than that of peak **73**, consistent with the loss of one methoxy group. They were therefore tentatively identified as isosinensetin, sinensetin, and tangeretin, respectively. Peaks **64** and **72** were tentatively assigned as demethoxylated tetramethoxyflavone isomers. Peak **77** displayed additional methoxylation, and peak **81** was characterized by distinct A- and B-ring RDA fragments. PMFs are typically found in Rutaceae (e.g., *Citrus*) [28] and are reported here for the first time in AR, potentially contributing to the chemical differentiation of HLQ. Fragmentation pathways are provided in Figure S4.

#### 2.3.6. Anthraquinones

Anthraquinones, characterized by a 9,10-anthracenedione core, were identified as a distinct chemical cluster in the HLQE (Figure 2 and Figure S10). Their mass spectrometric behavior is dominated by the successive elimination of CO (28 Da) and H_2_O (18 Da). Additionally, substituents on the aromatic ring, such as COOH, CH_3_O, and CH_2_OH, are prone to neutral loss, yielding corresponding diagnostic fragments [30].

Peak **87** (t_R_ = 37.99 min, *m*/*z* 341.1408 [M+H]^+^, C_20_H_20_O_5_) was selected as a representative compound to illustrate the diagnostic fragmentation pathway (Figure 4C). Its protonated precursor ([M+H]^+^ at *m*/*z* 341.1408) initially lost H_2_O to yield a diagnostic ion at *m*/*z* 323.1302. This ion underwent a signature neutral loss of 58 Da (C_3_H_6_O), generating the key fragment at *m*/*z* 265.0899. This loss is diagnostic of retro-aldol fragmentation of a 2,3-dihydroxy-3-methylbutyl side chain. It thus provides direct MS/MS evidence for the presence of this substituent. Sequential losses of CO, H_2_O, and CH_3_ confirmed the anthraquinone skeleton. RDA cleavage further generated a diagnostic ion at *m*/*z* 133.0277, indicative of substituents on opposite rings. On the basis of these MS/MS features, peak **87** was tentatively assigned as a novel anthraquinone derivative.

Guided by these fragmentation rules and spectral networking within cluster VIII, 2-hydroxy-3-methylanthraquinone (peak **71**) and methyl anthraquinone-1-acetate (peak **69**) were tentatively annotated based on consistent neutral losses. Peaks **44** and **50** were tentatively assigned via manual MS/MS interpretation [30,31]. In total, five anthraquinones were characterized, with fragmentation pathways provided in Figure S4.

#### 2.3.7. Fatty Acids

A total of 12 fatty acids were characterized in the HLQE, encompassing both common and previously unreported polyunsaturated types (Figure 2 and Figure S11). Peak **101** (t_R_ = 43.97 min, *m*/*z* 317.2101 [M+Na]^+^, C_18_H_30_O_3_), isolated and fully characterized in this study, served as an anchor compound for spectral annotation. Its MS/MS spectrum (Figure 4D) exhibited characteristic losses of H_2_O, CO, and double-bond cleavage [32,33]. On the basis of molecular networking (cluster IV), peak **101** served as a reference for annotating structurally related analogues, including hydroxylated fatty acids (peaks **51**, **58**, **83**, and **93**), oxooctadecadienoic acid isomers (peaks **100** and **102**), and glycerol esters (peaks **94**, **96**, and **97**) [34]. Peaks **60** and **79** showed no match in GNPS or SciFinder and were tentatively proposed as novel fatty acid derivatives. Peak **79** exhibited a mass 20 Da greater than that of peak **60** and a fragmentation pattern indicative of two additional double bonds. It was therefore tentatively identified as an octadecatrienoic acid isomer. All characterized fatty acids are polyunsaturated and are reported here for the first time in AR. Fragmentation pathways are provided in Figure S4.

FBMN-based annotation revealed that HLQE was enriched with multiple inflammation-associated compound classes, including isoflavones, pterocarpenoids, isoflavans, PMFs, cycloastragenol-type saponins, anthraquinones, and fatty acids. Previous studies have suggested that isoflavones downregulate iNOS and COX-2 expression and reduce pro-inflammatory cytokine production. These effects are mediated through inhibition of NF-κB DNA-binding activity and MAPK phosphorylation [34]. Pterocarpenoids represent a characteristic bioactive scaffold in AR. Medicarpin-type derivatives have been shown to prevent articular cartilage erosion by modulating the TH-17/Treg cell ratio and suppressing TNF-α and IL-6 production in arthritis models [35]. Astragaloside IV has been demonstrated to target macrophages in adjuvant-induced arthritis models, inhibiting inflammatory mediator production and protecting cartilage from IL-1β-induced damage [36]. PMFs have been reported to exert anti-inflammatory effects through the NF-κB, MAPK, and Nrf2 pathways [37]. The anthraquinone peak **44** (2-(hydroxymethyl)-anthraquinone) has been shown to suppress inflammation via the TLR4-NF-κB axis [38]. The distinct and complementary mechanisms of these compound classes suggest that the anti-inflammatory potential of HLQE may reflect synergistic contributions across multiple constituent groups. This chemically enriched profile provided a rational basis for the targeted isolation and functional evaluation of representative constituents to further elucidate the characteristic bioactive composition of HLQ.

### 2.4. Structural Elucidation of Isolated Compounds

**Figure 5:**
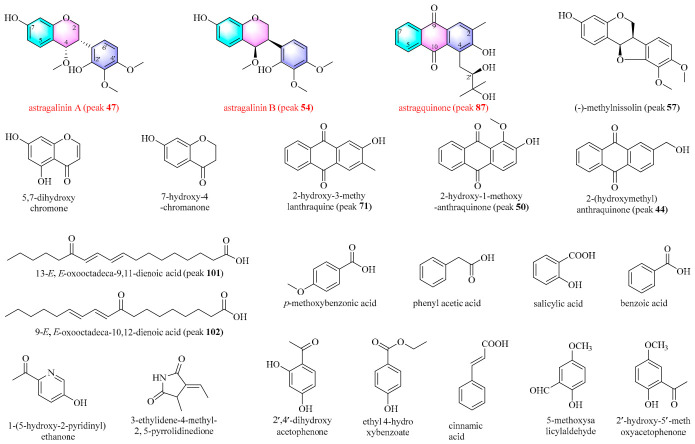
Chemical structures of isolated compounds in the ethyl acetate fraction of Honglanqi (HLQE). (Novel compounds are labeled in red, and known compounds are labeled in black).

Guided by the FBMN-based annotation, candidate novel compounds were prioritized for targeted isolation. A total of 22 compounds were isolated from the HLQE, comprising three novel compounds, astragalinin A (peak **47**), astragalinin B (peak **54**), and astragquinone (peak **87**), together with 19 known compounds (Figure 5). Detailed spectroscopic data for the novel compounds, including infrared (IR), ultraviolet (UV), circular dichroism (CD), electronic circular dichroism (ECD), and nuclear magnetic resonance (NMR) spectra, are provided in Table 1 and Figures S12–S41. The known compounds were identified as (-)-methylnissolin (peak **57**) [39], 5,7-dihydroxychromone [40], 7-hydroxy-4-chromanone [41], 2-hydroxy-3-methylanthraquinone (peak **71**) [42], 2-hydroxy-1-methoxy-anthraquinone (peak **50**) [31], 2-(hydroxymethyl)anthraquinone (peak **44**) [43], 13-(*E*,*E*)-oxooctadeca-9,11-dienoic acid (peak **101**), 9-(*E*,*E*)-oxooctadeca-10,12-dienoic acid (peak **102**) [33], *p*-methoxybenzoic acid [44], phenyl acetic acid [45], salicylic acid, benzoic acid, cinnamic acid [46], 2′,4′-dihydroxyacetophenone [47], ethyl 4-hydroxybenzoate [48], 5-methoxysalicylaldehyde [49], 2′-hydroxy-5′-methoxyacetophenone [50], 1-(5-Hydroxy-2-pyridinyl)ethenone [51], and 3-ethylidene-4-methyl-2,5-pyrrolidinedione [52], on the basis of NMR data and comparison with previously reported values.

High-resolution mass spectrometry (HRMS) analysis revealed that astragalinin A (peak **47**) and astragalinin B (peak **54**) are isomers with the molecular formula C_18_H_20_O_6_ (observed *m*/*z* 355.1153 [M+Na]^+^; calcd 355.1158). Detailed analysis of the NMR spectroscopic data established that peaks **47** and **54** share the same planar structure, identified as 3-(2-hydroxy-3,4-dimethoxyphenyl)-4-methoxychroman-7-ol (Table 1 and Figures S12–S16). The characteristic isoflavan core of peak **47** was established by ^1^H NMR signals at *δ*_H_ 4.34 (d, *J* = 4.0 Hz, H-2), 3.58 (q, *J* = 4.0 Hz, H-3), and 4.39 (d, *J* = 4.0 Hz, H-4), along with corresponding ^13^C signals at *δ*_C_ 65.1 (C-2), 35.1 (C-3), and 75.5 (C-4). The spin system of H-2/H-3/H-4 was further confirmed by ^1^H−^1^H COSY correlations. The ring substitution patterns were definitively established through key 2D NMR correlations. For peak **47**, key HMBC correlations from H-5 (*δ*_H_ 7.04) to C-4 (*δ*_C_ 75.5), C-7 (*δ*_C_ 158.4), and C-8a (*δ*_C_ 155.4) confirmed the presence of a 7-hydroxyl group on ring A. Diagnostic correlations from H-5′ (*δ*_H_ 6.32) to C-1′ (*δ*_C_ 118.9), C-3′ (*δ*_C_ 135.7), and C-4′ (*δ*_C_ 151.5), and from H-6′ (*δ*_H_ 6.75) to C-2′ (*δ*_C_ 147.6) and C-4′, defined the 2′-hydroxy-3′,4′-dimethoxy substitution pattern on ring B. The linkage between the C- and B-rings was secured by the pivotal HMBC correlation from H-3 to C-1′. The relative *cis*-configuration between H-3 and H-4 was deduced from their characteristic small coupling constant (*J*_3,4_ = 4.0 Hz) and confirmed by strong ROESY correlations. The NMR data of peak **54** were indistinguishable from those of peak **47**, indicating an enantiomeric relationship. They were differentiated by chiroptical analysis. Peak **47** showed a negative specific rotation ([α${]}_{D}^{25}$ −4.0, c 0.1, MeOH), whereas peak **54** displayed a positive value ([α${]}_{D}^{25}$ +31.0, c 0.1, MeOH). Their experimental ECD spectra exhibited mirror-image Cotton effects. Comparison with TD-DFT calculated curves enabled unambiguous assignment of absolute configurations as (3*S*,4*S*) for peak **47** and (3*R*,4*R*) for peak **54** (Figures S21 and S31). Accordingly, peaks **47** and **54** were conclusively identified as (3*S*,4*S*)-3-(2-hydroxy-3,4-dimethoxyphenyl)-4-methoxychroman-7-ol and (3*R*,4*R*)-3-(2-hydroxy-3,4-dimethoxyphenyl)-4-methoxychroman-7-ol, respectively.

Peak **87** was obtained as a yellow amorphous powder. By HRMS analysis, on the basis of the prominent dehydrated ion at *m*/*z* 323.1275 [M+H-H_2_O]^+^ (calcd for C_20_H_19_O_4_, 323.1283), its molecular formula was established as C_20_H_20_O_5_. The ^1^H and ^13^C NMR spectra (Table 1) revealed a trisubstituted anthraquinone skeleton. The aromatic ring B was characterized by two quinone carbonyls at *δ*_C_ 183.4 (C-9) and *δ*_C_ 185.3 (C-10), and a diagnostic singlet at *δ*_H_ 7.93 (H-1). The substituents included a methyl group at C-2 (*δ*_C_ 126.8), a hydroxyl group at C-3 (*δ*_C_ 166.0), and a 2,3-dihydroxy-3-methylbutyl side chain at C-4. The connectivity within the side chain was supported by ^1^H−^1^H COSY correlations between H-1′ and H-2′. Key HMBC correlations from the side-chain methylene protons H_2_-1′ (*δ*_H_ 3.65) to C-3 and C-4 (*δ*_C_ 130.3) unambiguously confirmed the attachment point at C-4. The side chain architecture was verified by the diagnostic MS/MS fragment at *m*/*z* 265.0899. This ion was generated from the dehydrated precursor [M+H-H_2_O]^+^ (*m*/*z* 323.1275) via retro-aldol cleavage, involving a neutral loss of acetone (C_3_H_6_O, 58 Da). This fragmentation is highly characteristic of the 2,3-dihydroxy-3-methylbutyl moiety. The absolute *R*-configuration at the stereogenic center C-2′ was assigned using ECD spectroscopy supported by TD-DFT calculations. The experimental and calculated spectra showed high congruence. Accordingly, peak **87** was identified as (*R*)-1-(2,3-dihydroxy-3-methylbutyl)-2-hydroxy-3-methylanthracene-9,10-dione.

Astragalinin A (peak **47**). Colourless oil: [α${]}_{D}^{25}$ −4.0 (c 0.1, MeOH); UV (MeOH) λ_max_ (log *ε*) 212 (4.03), 249 (2.54), 279 (3.26) nm; CD (c 0.75 × 10^−4^ M, MeOH): λ (Δε) 203 (−29.64), 219 (+1.43), 279 (+3.54); IR (neat) *ν*_max_ 3376, 2938, 2835, 1618, 1596, 1507, 1464, 1431, 1315, 1276, 1225, 1163, 1123, 1098, 1023, 958, 846, 796 cm^−1^; for ^1^H NMR and ^13^C NMR data see Table 1; Positive HR-ESI-MS: *m*/*z* 355.1153 [M+Na]^+^ (calcd. for C_18_H_20_O_6_Na, 355.1158).

Astragalinin B (peak **54**). Colourless oil: [α${]}_{D}^{25}$ +31.0 (c 0.1, MeOH); UV (MeOH) λ_max_ (log *ε*) 207 (3.95), 246 (2.22), 279 (2.88) nm; CD (c 0.75 × 10^−4^ M, MeOH): λ (Δε) 207 (+3.11), 221 (+0.07), 229 (+0.27), 279 (+0.29); IR (neat) *ν*_max_ 3382, 2934, 2835, 1619, 1596, 1507, 1464, 1431, 1331, 1280, 1225, 1164, 1123, 1096, 1024, 846, 794 cm^−1^; for ^1^H NMR and ^13^C NMR data see Table 1; Positive HR-ESI-MS: *m*/*z* 355.1153 [M+Na]^+^ (calcd. for C_18_H_20_O_6_Na, 355.1158).

Astragquinone (peak **87**). Yellow amorphous powder: [α${]}_{D}^{25}$ −5.0 (c 0.1, MeOH); UV (MeOH) λ_max_ (log *ε*) 206 (3.97), 214 (3.83), 241 (4.06), 260 (3.87), 279 (4.08), 348 (3.13), 394 (3.33) nm; CD (c 0.8 × 10^−4^ M, MeOH): λ (Δε) 200 (+1.62), 211 (+0.17), 241 (+0.06), 253 (+0.13), 314 (+0.17); IR (neat) *ν*_max_ 3461, 2975, 2932, 1667, 1583, 1573, 1465, 1418, 1382, 1329, 1293, 998, 959, 803, 716 cm^−1^; for ^1^H NMR and ^13^C NMR data see Table 1; Positive HR-ESI-MS: *m*/*z* 323.1275 [M+H-H_2_O]^+^ (calcd. for C_20_H_19_O_4_, 323.1283).

### 2.5. Anti-Inflammatory Evaluation of Representative Isolated Constituents

**Figure 6:**
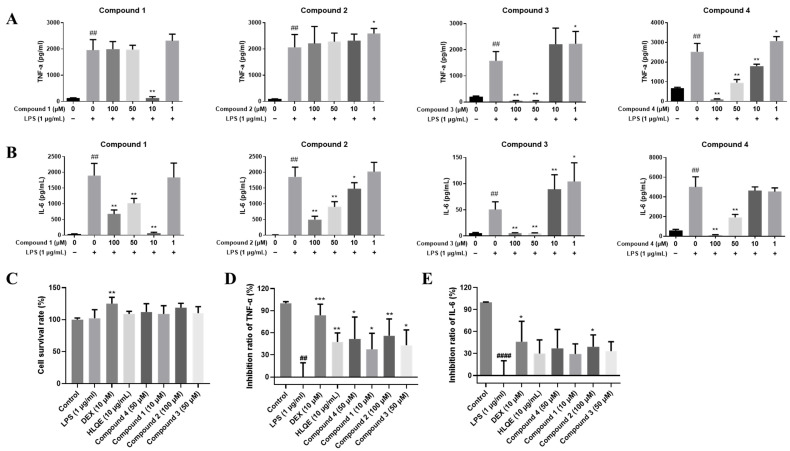
Preliminary anti-inflammatory screening and comparative evaluation of isolated compounds and HLQE in RAW 264.7 macrophages. (**A**,**B**) Preliminary screening of compounds **1**–**4**: effects on (**A**) TNF-α level and (**B**) IL-6 level in LPS-stimulated RAW 264.7 cells. (**C**–**E**) Comparative evaluation of (**C**) cell viability and the inhibition rates of (**D**) TNF-α and (**E**) IL-6 for the four compounds at their respective optimal concentrations (compound 1 at 10 μM, compound 2 at 100 μM, compound 3 at 50 μM, and compound 4 at 50 μM), HLQE (10 μg/mL), and dexamethasone (DEX, 10 μM) under identical conditions. Data are displayed as mean ± SD (*n* ≥ 4). No significant cytotoxicity was observed for any tested sample. Statistical significance: ^####^ *p* < 0.0001 or ^##^ *p* < 0.01 vs. the control group; * *p* < 0.05, ** *p* < 0.01, and *** *p* < 0.001 vs. the LPS-stimulated model group. Corresponding numerical inhibition rates are provided in Table S4. LPS: lipopolysaccharide; DEX: dexamethasone; HLQE: ethyl acetate fraction of Honglanqi; Compound **1**: astragalinin A; Compound **2**: astragalinin B; Compound **3**: astragquinone; Compound **4**: 2-(hydroxymethyl)anthraquinone, internal reference.

To evaluate the contribution of representative isolated constituents to the anti-inflammatory activity of HLQ, four compounds were assessed for their inhibitory effects on TNF-α and IL-6 production in LPS-stimulated RAW 264.7 macrophages. These included three novel compounds: astragalinin A(compound **1**, peak **47**), astragalinin B (compound **2**, peak **54**), and astragquinone (compound **3**, peak **87**), together with the previously reported anti-inflammatory constituent 2-(hydroxymethyl)anthraquinone (compound **4**, peak **44**) [38]. Cytotoxicity screening by CCK-8 assay confirmed that compounds **1** and **2** were well tolerated up to 1000 μM, compound **3** up to 500 μM, and compound **4** up to 50 μM (Figure S3). Based on these results, safe concentration ranges were established for subsequent anti-inflammatory assessment (Figure 6). ELISA results demonstrated that compounds **1**, **3**, and **4** significantly inhibited the release of TNF-α (Figure 6A). Compounds **1** (a 4-methoxyisoflavan) and **3** (a novel anthraquinone with a 2,3-dihydroxy-3-methylbutyl side chain) exhibited potent inhibitory activity. Compounds **1** and **4** showed significant effects at 10 μM (*p* < 0.01 vs. model group). Regarding IL-6, all tested compounds effectively suppressed its secretion at 50 μM (*p* < 0.01) (Figure 6B). Compound **1** maintained significant inhibitory activity at 10 μM (*p* < 0.01), whereas **2** showed moderate suppression at the same dosage (*p* < 0.05). These findings, supported by the IC_50_ values (Table 2), demonstrate that the novel anthraquinone and 4-methoxyisoflavans contribute to the superior anti-inflammatory potential of HLQ relative to traditional markers.

Comparative evaluation of the isolated compounds and HLQE against dexamethasone (DEX) at their respective optimal inhibitory concentrations further confirmed the anti-inflammatory potential of HLQ constituents (Table S4 and Figure 6C–E). All tested samples maintained acceptable cell viability under assay conditions (Figure 6C). As shown in Figure 6D,E, DEX (10 μM) strongly inhibited TNF-α (83.8%, *p* < 0.001) and IL-6 (46.1%, *p* < 0.05), confirming the responsiveness and reliability of the assay system. For TNF-α inhibition, HLQE (47.6 ± 6.0% at 10 μg/mL) and all tested compounds showed significant suppression (*p* < 0.05). Among them, compound **2** (56.0 ± 10.2% at 100 μM) and compound **4** (51.6 ± 14.9% at 50 μM) showed the strongest effects. Notably, compound 1 achieved significant inhibition at only 10 μM, suggesting a lower minimum effective concentration. For IL-6, compound **2** showed statistically significant inhibition (39.2 ± 7.3%, *p* < 0.05). HLQE and the remaining compounds exhibited a decreasing trend that did not reach statistical significance.

### 2.6. Molecular Docking Analysis

**Figure 7:**
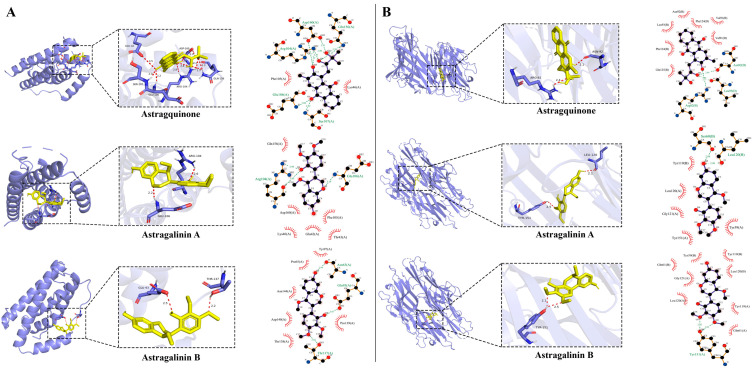
Molecular docking study of novel compounds (astragalinin A, astragalinin B, and astragquinone) with inflammatory cytokine (IL-6 and TNF-α) target proteins. (**A**) 3D and 2D ligand interaction diagram of novel compounds and IL-6 (PDB ID: 1ALU). (**B**) 3D and 2D ligand interaction diagram of novel compounds and TNF-α (PDB ID: 2AZ5).

To further explore the potential binding interactions of the novel compounds with inflammatory targets, molecular docking simulations were performed to evaluate their interactions with TNF-α (PDB: 2AZ5) and IL-6 (PDB: 1ALU). All compound–protein pairs exhibited binding energies below −5 kcal/mol, indicating potentially favorable ligand–protein interactions (Table S5). Docking analysis suggested that astragalinin A, astragalinin B, and astragquinone may adopt stable binding poses within the binding pockets of both TNF-α and IL-6 (Figure 7). Among them, astragquinone exhibited the strongest predicted binding affinity toward both TNF-α (−9.1 kcal/mol) and IL-6 (−7.5 kcal/mol), which may be attributable to its broader predicted hydrogen-bonding and hydrophobic interaction network relative to astragalinin A and astragalinin B. Notably, the enantiomeric pair astragalinin A and astragalinin B adopted distinct predicted binding orientations and interacted with different residues despite occupying similar binding regions. This observation suggests that stereochemical configuration may influence protein recognition and binding mode. For TNF-α, TYR-151 was identified as a common interacting residue for astragalinin B and astragquinone. For IL-6, ARG-104 and GLU-106 were involved in the predicted binding of astragalinin A and astragquinone. These residues may represent key pharmacophoric sites worthy of further experimental investigation. Overall, the docking results were consistent with the observed cellular anti-inflammatory activity, providing preliminary computational insights into potential ligand–protein interactions. These findings may inform future target-based mechanistic studies.

Bioactivity evaluation combined with docking analysis provided preliminary structure–activity relationship insights into the novel compounds. The enantiomeric isoflavans exhibited marked differences in potency. Astragalinin A strongly inhibited both TNF-α and IL-6 (IC_50_ < 10 μM), whereas astragalinin B was substantially less active. This difference indicates that stereochemical configuration may critically influence anti-inflammatory potency in this structural class. Astragquinone, bearing a distinctive 2,3-dihydroxy-3-methylbutyl substituent, showed balanced cytokine suppression with a favorable safety profile. It also exhibited the strongest predicted binding affinity toward TNF-α (−9.1 kcal/mol) among the tested compounds. The present study provides a comprehensive phytochemical characterization and preliminary anti-inflammatory evaluation of HLQ; however, certain limitations should be acknowledged. The biological evaluation was confined to cytokine-level readouts (TNF-α and IL-6). Mechanistic endpoints at the signaling level, such as NF-κB nuclear translocation, MAPK phosphorylation, and iNOS/COX-2 expression, were not investigated. The quantitative contribution of individual isolated constituents to the overall anti-inflammatory activity of HLQE remains to be formally established. Furthermore, the 18 proposed novel compounds represent putative structural assignments based on mass spectral evidence. Definitive confirmation requires isolation and comprehensive spectroscopic characterization. These findings nonetheless highlight the contribution of structurally diverse metabolites to the anti-inflammatory potential of HLQ. Collectively, these results support HLQ as a chemically enriched functional resource worthy of further mechanistic and pharmacological investigation.

## 3. Materials and Methods

### 3.1. Reagents and Cell Lines

LC–MS grade methanol (purity ≥ 99.9%), acetonitrile (purity ≥ 99.9%), and formic acid (purity ≥ 98%) were purchased from Merck (Darmstadt, Germany). Reference standards, including isoflavonoids (calycosin-7-*O*-*β*-D-glucoside, ononin, calycosin, and formononetin), pterocarpenoids ((−)-methylnissolin-3-*O*-glucoside), isoflavans (isomucronulatol-7-*O*-*β*-D-glucopyranoside and isomucronulatol), polymethoxyflavones (nobiletin), triterpenoid saponins (astragalosides I–IV, isoastragalosides I–II, cyclocephaloside II, and cycloastragenol), and the triterpenoid aglycone cycloastragenol were purchased from Beijing BetterRen Biological Technology Co., Ltd. (Beijing, China) and Shanghai Yuanye Bio-Technology Co., Ltd. (Shanghai, China), with purity ≥ 98%. Lipopolysaccharide (LPS, *Escherichia coli* O111:B4, purity ≥ 98%) was purchased from Sigma–Aldrich (St. Louis, MO, USA). ELISA kits for TNF-α and IL-6 were purchased from MultiSciences (Hangzhou, China). RAW264.7 macrophages were obtained from the Cell Resource Center of the Chinese Academy of Medical Sciences and Peking Union Medical College (Beijing, China). Cells were cultured in high-glucose Dulbecco’s Modified Eagle’s Medium (DMEM; Thermo Fisher Scientific, Waltham, MA, USA) supplemented with 10% fetal bovine serum (FBS; Thermo Fisher Scientific, Waltham, MA, USA). Culture conditions were maintained at 37 °C in a humidified 5% CO_2_ incubator.

### 3.2. Sample Collection and Preparation

**Figure 8:**
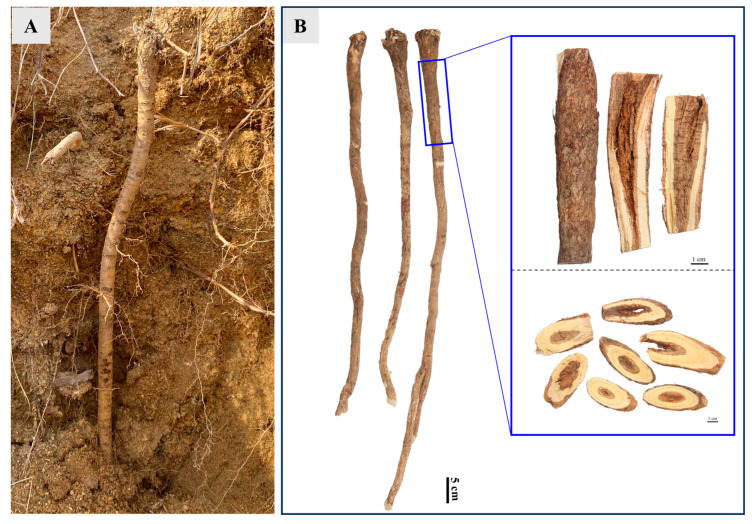
Collection of wild-simulated *Astragalus membranaceus* var. *mongholicus* and presentation of Honglanqi (HLQ) samples. (**A**) Collection of wild-simulated *A. membranaceus* var. *mongholicus* samples. (**B**) Commercial specification of HLQ and its longitudinal and transverse sections.

WAR samples exhibiting rhytidome and interxylary cork characteristics were freshly collected from Hunyuan County, Shanxi Province, China, in November 2022. The samples were authenticated as the roots of *Astragalus membranaceus* (Fisch.) Bge. var. *mongholicus* (Bge.) Hsiao by Prof. Huasheng Peng of the National Resource Center for Chinese Materia Medica, China Academy of Chinese Medical Sciences. The collected samples were air-dried at room temperature to a constant weight before further processing. HLQ materials were prepared according to standard processing specifications (Figure 8) [11]. The air-dried HLQ (36.5 kg) was extracted three times with 75% ethanol under reflux at 80 °C (2 h each). The combined extracts were filtered and concentrated under reduced pressure to approximately 10 L of a semiliquid extract. The concentrate was then suspended in deionized water (1:1, *v*/*v*). The suspension was sequentially partitioned with equal volumes of petroleum ether and ethyl acetate (four times each). The petroleum ether and ethyl acetate layers were each separately combined and concentrated under reduced pressure. This yielded the petroleum ether fraction (HLQP) and the ethyl acetate fraction (HLQE, 318 g, yield 0.80% *w*/*w*), respectively. The remaining aqueous layer was partially concentrated to obtain the aqueous fraction (HLQW) for bioassay evaluation. All fractions were stored at −20 °C until use.

### 3.3. UPLC–QTOF-MS/MS Analysis

Chemical profiling of HLQE was performed on a Waters ACQUITY UPLC system coupled to a Synapt XS quadrupole time-of-flight mass spectrometer (QTOF-MS, Waters, Milford, MA, USA), equipped with an HSS T3 column (2.1 mm × 100 mm, 1.8 μm). The mobile phase consisted of 0.1% formic acid in water (A) and acetonitrile (B) with a flow rate of 0.4 mL/min. The gradient elution program was as follows: 0–1 min, 18% B; 1–3 min, 18–20% B; 3–16 min, 20–22% B; 16–22 min, 22–30% B; 22–35 min, 30–40% B; 35–39 min, 40–50% B; 39–43 min, 50–63% B; 43–44 min, 63–66% B; 44–45 min, 66–70% B; 45–47 min, 70–72% B; 47–48 min, 72–18% B; and 48–50 min, 18% B. The column temperature was maintained at 38 °C, and the injection volume was 3 μL. MS data were acquired in positive ion mode (*m*/*z* 50–1500) using fast data-dependent acquisition (fast-DDA). Source parameters were set as follows: capillary voltage, 0.5 kV; cone voltage, 30 V; desolvation temperature, 450 °C; desolvation gas flow, 900 L/h. The 20 most intense precursor ions per scan were selected for MS/MS fragmentation using collision energy ramps of 15–25 V and 65–75 V. Leucine enkephalin (*m*/*z* 556.2771) was used as a lock mass for real-time mass accuracy calibration. Data acquisition and processing were performed using MassLynx software (version 4.1, Waters).

### 3.4. Feature-Based Molecular Networking (FBMN) Analysis

Fast-DDA data acquired in positive ion mode were processed using Progenesis QI (version 2.0, Waters). Features were detected within a retention time window of 0.5–45 min and filtered using an intensity threshold of >3000 counts. Features detected in procedural blank samples were excluded. MS1 and MS2 spectra were aligned based on chromatographic co-elution, with a mass tolerance of 10 ppm. The exported feature table (.csv) and MS/MS summary (.msp) files were uploaded to the GNPS platform (https://gnps.ucsd.edu) via the Progenesis-QI workflow [53]. Feature-based molecular networks were constructed using the following parameters: precursor and fragment ion tolerances of 0.02 Da, a minimum cosine score of 0.7, and a minimum of six matched fragment ions. Spectral library matches were filtered using the same criteria [20]. The resulting molecular networks were visualized and annotated using Cytoscape (version 3.10.2). Network data and parameters are publicly accessible at https://gnps.ucsd.edu/ProteoSAFe/status.jsp?task=5b6a03f2ecad4fe989fd2563f7fadd8e (access on 16 November 2025).

### 3.5. Isolation of Novel Compounds

General experimental procedures for column chromatography and spectroscopic analysis are provided in Appendix A.1. The HLQE (318 g) was first separated into four fractions (Fr.1–Fr.4) using a silica gel column with petroleum ether–acetone (1:0−0:1). Fr.2 (4.2 g) was fractionated on a silica gel column using a petroleum ether–acetone (100:1−5:1) gradient to yield five fractions (Fr.2.1 to Fr.2.5). Fr.2.4 (450.8 mg) was purified by semipreparative high-performance liquid chromatography (HPLC) (80% MeCN in H_2_O, 2 mL/min) to yield peak **87** (2.2 mg, t_R_ 13.7 min). Fr. 3 (11.5 g) was subjected to silica gel using column chromatography using a petroleum ether-ethyl acetate (50:1–0:1) gradient to yield three subfractions (Fr. 3.1 to Fr. 3.3). Fr. 3.1 (1.0 g) was separated by ODS CC to obtain Fr. 3.1.1 (175 mg). It was then purified by semipreparative HPLC (55% MeCN in H_2_O, 2 mL/min) to yield peak **54** (2.0 mg, t_R_ 14.3 min). Fr. 3.2 (1.3 g) was separated into two subfractions (Fr. 3.2.1 and Fr. 3.2.2) by a silica gel column with petroleum ether-ethyl acetate (20:1–0:1) gradient elution. Fr. 3.2.2 (312.7 mg) was purified by Sephadex LH-20 and semi-preparative HPLC (55% MeCN in H_2_O, 2 mL/min) to obtain peak **47** (1.6 mg, t_R_ 12.1 min). The absolute configurations of peaks **47**, **54**, and **87** were determined by ECD calculations; detailed computational procedures are described in Appendix A.2.

### 3.6. In Vitro Anti-Inflammatory Activity Evaluation

RAW264.7 cells were cultured as described in Section 3.1, with the addition of 1% penicillin–streptomycin. For preliminary fraction screening, the cytotoxicity of HLQP, HLQE, and HLQW was assessed by CCK-8 assay in RAW264.7 macrophages across a seven-point concentration range (200, 100, 50, 10, 5, 1, and 0.1 μg/mL). Inhibitory effects on TNF-α and IL-6 production were subsequently evaluated at the confirmed non-cytotoxic concentration of 10 μg/mL in LPS-stimulated RAW 264.7 macrophages. For the evaluation of isolated compounds, the cytotoxicity of compounds **1**–**4** (peaks **47**, **54**, **87**, and **44**, respectively) was assessed by CCK-8 assay across a series of concentrations. Only non-cytotoxic concentrations were used in subsequent experiments. Cells were then divided into the normal control group, the LPS-stimulated model group (LPS, 1 μg/mL), and the compound-treated groups. In the presence of LPS (1 μg/mL), compounds **1**–**4** were each tested at four concentrations within their respective non-cytotoxic ranges for 24 h. Cell viability was assessed by measuring absorbance at 450 nm, and culture supernatants were collected for quantification of TNF-α and IL-6 levels using ELISA kits according to the manufacturer’s instructions. IC_50_ values for cytokine inhibition were determined by nonlinear regression analysis. Based on the resulting IC_50_ values, the optimal concentration for each compound was selected. Each compound was then compared with dexamethasone (DEX, 10 μM) as a positive control to calculate inhibition rates of TNF-α and IL-6. The final DMSO concentration in the medium was maintained below 0.1% (*v*/*v*). All experiments were independently repeated at least three times with six replicate wells per condition (*n* = 6).

### 3.7. Molecular Docking

The three-dimensional structures of IL-6 (PDB: 1ALU) and TNF-α (PDB: 2AZ5) were retrieved from the RCSB Protein Data Bank (https://www.rcsb.org). Protein structures were prepared using PyMOL (version 3.0.4) and AutoDockTools (version 1.5.7). Preparation steps included removal of water molecules and heteroatoms, addition of polar hydrogens, and assignment of Gasteiger charges. The prepared receptor files were saved in PDBQT format. Ligand structures of astragalinin A, astragalinin B, and astragquinone were drawn in ChemDraw (version 14.0) and converted to MOL2 format. Geometry optimization was performed using AutoDockTools before docking. Molecular docking was performed using AutoDock Vina, with a grid box centered on the active site of each target protein. Docking results were visualized and analyzed using PyMOL and LigPlot+ v2.3.

### 3.8. Statistical Analysis

Data were collected from at least three independent experiments. Statistical analysis was performed using GraphPad Prism (version 6.01). Results are expressed as mean ± standard deviation (SD), unless otherwise stated. IC_50_ values were calculated by nonlinear regression analysis and expressed as mean ± standard error of the mean (SEM). One-way analysis of variance (ANOVA) followed by Tukey’s test was used to compare groups. Statistical significance was set at *p* < 0.05.

## 4. Conclusions

In the present study, an integrated UPLC–QTOF-MS/MS and FBMN strategy was employed to systematically characterize the chemical composition of HLQ. Comparative anti-inflammatory evaluation showed that HLQE exhibited the strongest inhibitory effects on TNF-α and IL-6. HLQE was therefore selected for subsequent phytochemical investigation. A total of 102 metabolites were characterized, including isoflavones, pterocarpenoids, triterpenoid saponins, isoflavans, polymethoxyflavones, anthraquinones, and fatty acids. Among these, 18 were proposed as potentially novel compounds, and 37 were reported in AR for the first time. Further isolation afforded three undescribed compounds, namely astragalinin A (peak **47**), astragalinin B (peak **54**), and astragquinone (peak **87**), together with 19 known compounds. Biological evaluation demonstrated that astragalinin A exhibited potent inhibition of TNF-α and IL-6 (IC_50_ < 10 μM), whereas astragquinone showed significant TNF-α inhibition (IC_50_ = 36.8 μM) with reduced cytotoxicity relative to the reference anthraquinone. Comparative evaluation of the enantiomeric pair further revealed that stereochemical configuration critically influences anti-inflammatory potency. Molecular docking suggested potentially favorable binding orientations toward TNF-α and IL-6 in silico, with astragquinone exhibiting the strongest predicted affinity toward TNF-α (−9.1 kcal/mol). Overall, these findings indicate that HLQ represents a chemically enriched AR commercial specification containing structurally diverse anti-inflammatory constituents. Collectively, these results provide a scientific basis for the rational grading and value-added utilization of HLQ within the AR production chain.

## Figures and Tables

**Table 1:** ^1^H NMR and ^13^C NMR data of peaks **47**, **54**, and **87** in CD_3_OD. (^1^H: 500 MHz; ^13^C: 125 MHz).

Pos	Peak 47		Peak 54		Pos	Peak 87	
	*δ*_C_, Type	*δ*_H_, Mult. (*J* in Hz)	*δ*_C_, Type	*δ*_H_, Mult. (*J* in Hz)		*δ*_C_, Type	*δ*_H_, Mult. (*J* in Hz)
1					1	131.2	7.93, s
2	65.1	4.34, d, 4.0	65.8	4.49, dd, 12.0, 10.0 4.16, ddd, 10.0, 3.5, 1.5	2	126.8	
3	35.1	3.58, q, 4.0, 4.0, 4.0	37.8	3.56, dt, 12.0, 3.5	3	166.0	
4	75.5	4.39, d, 4.0	76.3	4.33, d, 3.5	4	130.3	
5	131.1	7.04, d, 8.0	132.6	7.04, d, 8.0	5	127.8	8.19, m
6	107.7	6.36, dd, 8.0, 2.5	108.5	6.35, dd, 8.0, 2.5	6	135.0	7.80, m
7	158.4		159.9		7	134.8	7.80, m
8	101.8	6.23, d, 2.5	103.8	6.26, d, 2.5	8	127.6	8.19, m
8a	155.4		156.5		9	183.4	
4a	112.2		115.4		10	185.3	
1′	118.9		119.6		11	135.1	
2′	147.6		149.5		12	135.2	
3′	135.7		137.3		13	129.6	
4′	151.5		153.2		14	128.2	
5′	101.4	6.32, d, 8.5	104.1	6.47, d, 8.5	1′	33.1	3.65, m
6′	121.9	6.75, d, 8.5	124.1	6.84, d, 8.5	2′	92.6	4.82, dd, 9.0, 8.0
4-OCH_3_	54.3	3.44, s	57.5	3.14, s	3′	72.5	
3′-OCH_3_	59.3	3.78, s	61.0	3.79, s	4′	25.4	1.33, s
4′-OCH_3_	54.5	3.76, s	56.2	3.82, s	5′	25.4	1.28, s
					2-CH_3_	15.7	2.33, s

**Table 2:** IC_50_ values of isolated compounds for inhibition of IL-6 and TNF-α production in LPS-stimulated RAW264.7 macrophages.

Compounds	IC_50_ (μM) for TNF-α	IC_50_ (μM) for IL-6
**1**	<10	9.1
**2**	>100	43.1
**3**	36.8	37.9
**4**	25.2	44.8

Notes: IC_50_ values were determined by nonlinear regression analysis of dose–response curves generated at four concentrations (1, 10, 50, and 100 μM); representative curves are provided in Figures S42–S45. For compounds exhibiting >50% inhibition at the lowest tested concentration (10 μM), IC_50_ values are reported as <10 μM. For compounds showing <50% inhibition at the highest concentration (100 μM), IC_50_ values are expressed as >100 μM. Compound **1**: astragalinin A; Compound **2**: astragalinin B; Compound **3**: astragquinone; Compound **4**: 2-hydroxymethyl anthraquinone, internal reference.

## Data Availability

The original contributions presented in this study are included in the article/Supplementary Materials. Further inquiries can be directed to the corresponding authors.

## Supplementary Materials

The following supporting information can be downloaded at: https://www.mdpi.com/article/10.3390/plants15101442/s1. Figure S1: Cytotoxicity screening and anti-inflammatory evaluation of HLQ fractions at 10 μg/mL in RAW 264.7 macrophages. Figure S2: Base peak ion (BPI) chromatogram of HLQE in positive ion mode. Figure S3: Effects of compounds **1**–**4** on RAW 264.7 cell viability assessed by CCK-8 assay. Figure S4: Fragmentation deduction of the annotated metabolites. (peaks **1**–**102**). Figure S5: A: Isoflavones tentatively assigned in HLQE samples. B: Proposed MS2 fragmentation pathways of representative compound (calycosin-7-*O*-*β*-D-glucoside). Figure S6: A: 4-methoxyisoflavans and pterocarpenoids tentatively assigned in HLQE samples. B: Proposed MS2 fragmentation pathways of representative compound (astragalinin A). Figure S7: A: Saponins tentatively assigned in HLQE samples. B: Proposed MS2 fragmentation pathways of representative compound (isoastragaloside I). Figure S8: A: Isoflavans tentatively assigned in HLQE samples. B: Proposed MS2 fragmentation pathways of representative compounds (isomucronulatol-7-*O*-*β*-D-glucopyranoside and isomucronulatol). Figure S9: A: Polymethoxyflavones tentatively assigned in HLQE samples. B: Proposed MS2 fragmentation pathways of representative compound (nobiletin). Figure S10: A: Anthraquinones tentatively assigned in HLQE samples. B: Proposed MS2 fragmentation pathways of representative compound (astragquinone). Figure S11: A: Fatty acids tentatively assigned in HLQE samples. B: Proposed MS2 fragmentation pathways of representative compound (13-*E*, *E*-oxooctadeca-9,11-dienoic acid). Figure S12: 1H NMR spectrum (500 MHz) of astragalinin A (peak **47**) in CD_3_OD. Figure S13: ^13^C NMR spectrum (125 MHz) of astragalinin A (peak **47**) in CD_3_OD. Figure S14: ^1^H−^1^H COSY spectrum (500 MHz) of astragalinin A (peak **47**) in CD_3_OD. Figure S15: HSQC spectrum (500 MHz) of astragalinin A (peak **47**) in CD_3_OD. Figure S16: HMBC spectrum (500 MHz) of astragalinin A (peak **47**) in CD_3_OD. Figure S17: ROESY spectrum (500 MHz) of astragalinin A (peak **47**) in CD_3_OD. Figure S18: UV spectrum of astragalinin A (peak **47**). Figure S19: IR spectrum of astragalinin A (peak **47**). Figure S20: CD spectrum of astragalinin A (peak **47**). Figure S21: ECD spectrum of astragalinin A (peak **47**). Figure S22: ^1^H NMR spectrum (500 MHz) of astragalinin B (peak **54**) in CD_3_OD. Figure S23: ^13^C NMR spectrum (125 MHz) of astragalinin B (peak **54**) in CD_3_OD. Figure S24: 1H-1H COSY spectrum (500 MHz) of astragalinin B (peak **54**) in CD_3_OD. Figure S25: HSQC spectrum (500 MHz) of astragalinin B (peak **54**) in CD_3_OD. Figure S26: HMBC spectrum (500 MHz) of astragalinin B (peak **54**) in CD_3_OD. Figure S27: ROESY spectrum (500 MHz) of astragalinin B (peak **54**) in CD_3_OD. Figure S28: UV spectrum of astragalinin B (peak **54**). Figure S29: IR spectrum of astragalinin B (peak **54**). Figure S30: CD spectrum of astragalinin B (peak **54**). Figure S31: ECD spectrum of astragalinin B (peak **54**). Figure S32: 1H NMR spectrum (500 MHz) of astragquinone (peak **87**) in CD_3_OD. Figure S33: ^13^C NMR spectrum (125 MHz) of astragquinone (peak **87**) in CD_3_OD. Figure S34: ^1^H−^1^H COSY spectrum (500 MHz) of astragquinone (peak **87**) in CD_3_OD. Figure S35: HSQC spectrum (500 MHz) of astragquinone (peak **87**) in CD_3_OD. Figure S36: HMBC spectrum (500 MHz) of astragquinone (peak **87**) in CD_3_OD. Figure S37: ROESY spectrum (500 MHz) of astragquinone (peak **87**) in CD_3_OD. Figure S38: UV spectrum of astragquinone (peak **87**). Figure S39: IR spectrum of astragquinone (peak **87**). Figure S40: CD spectrum of astragquinone (peak **87**). Figure S41: ECD spectrum of astragquinone (peak **87**). Figure S42: Dose–response curves of compound **1** on TNF-α and IL-6 production in LPS-stimulated RAW264.7 cells. Figure S43: Dose–response curves of compound **2** on TNF-α and IL-6 production in LPS-stimulated RAW264.7 cells. Figure S44: Dose–response curves of compound **3** on TNF-α and IL-6 production in LPS-stimulated RAW264.7 cells. Figure S45: Dose–response curves of compound **4** on TNF-α and IL-6 production in LPS-stimulated RAW264.7 cells. Table S1: Cell survival rate and inhibition of TNF-α and IL-6 by different polarity fractions of Honglanqi at 10 μg/mL in LPS-stimulated RAW264.7 cells. Table S2: Chemical constituents isolated from Huangqi. Table S3: Information of metabolites in HLQE identified by UPLC–Q-TOF/MS combined MS/MS-based mass spectral molecular networking. Table S4: Inhibition rates of TNF-α and IL-6 by HLQE, isolated compounds, and dexamethasone in LPS-stimulated RAW 264.7 macrophages. Table S5: Binding energy between key targets (TNF-α and IL-6) and key anti-inflammatory compounds in HLQE. Reference [54] is cited in the supplementary materials.

## Abbreviations

The following abbreviations are used in this manuscript:

HLQ	Honglanqi
AR	Astragali Radix
HLQE	HLQ ethyl acetate fraction
HLQP	HLQ petroleum ether fraction
HLQW	HLQ aqueous fraction
UPLC-QTOF-MS/MS	Ultra-high performance liquid chromatography–quadrupole time-of-flight tandem mass spectrometry
FBMN	Feature-based molecular networking
LPS	Lipopolysaccharide
TNF-α	Tumor necrosis factor-α
IL-6	Interleukin-6
ECD	Electronic circular dichroism
DFT	Density functional theory
ELISA	Enzyme-linked immunosorbent assay
CCK-8	Cell Counting Kit-8
DMEM	Dulbecco’s Modified Eagle’s Medium
FBS	Fetal bovine serum
IC50	Half maximal inhibitory concentration
DEX	Dexamethasone
CC	Column chromatography
HPLC	High-performance liquid chromatography
NMR	Nuclear magnetic resonance
SD	Standard deviation
SEM	Standard error of the mean
ANOVA	Analysis of variance

## Appendix A

### Appendix A.1. General Experimental Procedures

The Sephadex LH-20 and silica gel (60–100 mesh and 100–200 mesh) were purchased from Pharmacia Biotech (Uppsala, Sweden) and Yantai Wish Chemical Products Co., Ltd. (Shandong, China), respectively, and applied for column chromatography (CC). Semi-preparative high-performance liquid chromatography (HPLC) separation was performed on a SEP LC-52 (Separation (Beijing) Technology Co., Ltd., Beijing, China) using a C18 column (5 μm, 250 mm × 10 mm) with a UV detector. One-dimensional (1D) and two-dimensional (2D) NMR spectra (^1^H, 500MHz; ^13^C, 125MHz) were measured on a Bruker 500 spectrometer (Bremen, Germany). The chemical shifts are measured as *δ* values (ppm) using DMSO-*d*_6_ (*δ*_H_/*δ*_C_ 2.50/39.5) or CD_3_OD (*δ*_H_/*δ*_C_ 3.31/49.00) signals as references. UV data were acquired by UV-2102 (Unico, Shanghai, China). CD spectra were recorded by a JASCO J-815 spectropolarimeter (Tokyo, Japan). Optical rotations were recorded on a 241 polarimeter (PerkinElmer, Waltham, MA, USA). An FTIR-8400S spectrophotometer (Shimadzu, Kyoto, Japan) was used to measure the IR spectra. HR-ESI-MS spectra were obtained using a TOF-ESI-MS instrument.

### Appendix A.2. Computational Methods

Conformational analyses were performed using the MMFF94S force field, followed by geometry optimizations at the B3LYP-D3BJ/6-31G(d) level in MeOH (CPCM model). Conformers within 0–4 kcal/mol were re-optimized at the B3LYP-D3BJ/6-311G (2d, p) level, and frequency analyses confirmed all were true minima. ECD spectra of peaks **47**, **54**, and **87** were calculated by TD-DFT at the B3LYP/6-311+G (2d, p) level in MeOH using Gaussian09. The CD spectra were generated by the program SpecDis 1.6 (University of Würzburg, Würzburg, Germany) using a Gaussian band shape with 0.3 eV exponential half-width from dipole-length dipolar and rotational strengths.
