# Supplementary material for: Molecular Networking-Guided Phytochemical Profiling and Anti-Inflammatory Evaluation of Honglanqi, an Underutilized Commercial Specification of Astragali Radix
Source: Plants (Basel). 2026 May 8;15(10):1442. doi: 10.3390/plants15101442 (PMC13211090; doi:10.3390/plants15101442)
Supplement: Supplementary file 1 [file plants-15-01442-s001.zip › Supporting information_1 (Track Changes).pdf]

Supplementary Material

Molecular networking-guided phytochemical profiling and anti-inflammatory evaluation of Honglanqi, an underutilized commercial specification of Astragali Radix

Xiangmei Tan [a], Aoao Wang [b], Hongmei Li [b], Minzhen Yin [a,c,*], Huasheng Peng [a,c,*]

Authors Information

[a] State Key Laboratory for Quality Ensurance and Sustainable Use of Dao-di Herbs, National Resource Center for Chinese Materia Medica, China Academy of Chinese Medical Sciences, Beijing, 100700, P. R. China.
[b] Institute of Chinese Materia Medica, China Academy of Chinese Medical Sciences, Beijing 100700, P. R. China
[c] Key Scientific Research Base for Traditional Chinese Medicine Heritage (Institute of Chinese Materia Medica, China Academy of Chinese Medical Sciences), National Cultural Heritage Administration, Beijing, 100700, P. R. China.

* Corresponding author: National Resource Center for Chinese Materia Medica, China Academy of Chinese Medical Sciences, No. 16, Nanxiaojie, Dongzhimen Nei, Beijing 100700, China
E-mail addresses: minzheny@126.com (Minzhen Yin), hspeng@126.com (Huasheng Peng)

Content

Table S1 Cell survival rate and inhibition of TNF-α and IL-6 by different polarity fractions of Honglanqi at 10 μg/mL in LPS-stimulated RAW264.7 cells. 4

Table S2 Chemical constituents isolated from Huangqi. 5

Table S3 Information of metabolites in HLQE identified by UPLC-Q-TOF/MS combined MS/MS-based mass spectral molecular networking. .... 13~~14~~

Table S4 Inhibition rates of TNF-α and IL-6 by HLQE, isolated compounds, and dexamethasone in LPS-stimulated RAW 264.7 macrophages. .... 21~~22~~

Table S5 Binding energy between key targets (TNF-α and IL-6) and key anti-inflammatory compounds in HLQE. .... 21~~22~~

Fig. S1 Cytotoxicity screening and anti-inflammatory evaluation of HLQ fractions at 10 μg/mL in RAW 264.7 macrophages. (A) Cell viability of RAW 264.7 macrophages treated with HLQE, HLQP, and HLQW at a series of concentrations assessed by CCK-8 assay. (B) Cell viability, (C) TNF-α levels, and (D) IL-6 levels in LPS-stimulated RAW 264.7 macrophages treated with HLQE, HLQP, and HLQW at 10 μg/mL. C: normal control group; M: LPS-stimulated model group. Data are presented as mean ± SD ($n = 6$). Statistical significance: #$p < 0.05$, ##$p < 0.01$ vs. normal control group; *$p < 0.05$, **$p < 0.01$ vs. LPS-stimulated model group. .... 22~~23~~

Fig. S2 Base peak ion (BPI) chromatogram of HLQE in positive ion mode. .... 22~~23~~

Fig. S3 Effects of compounds 1–4 on RAW 264.7 cell viability assessed by CCK-8 assay. C: normal control group; V: vehicle control group (DMSO); NA: data invalid at this concentration due to solvent-induced cytotoxicity of the vehicle control. Statistical significance: *$p < 0.05$, **$p < 0.01$ vs. vehicle control group; ##$p < 0.01$ vs. normal control group. Data are presented as mean ± SEM ($n = 6$). Compound 1: astragalinin A (peak **47**); Compound 2: astragalinin B (peak **54**); Compound 3: astragaquinone (peak **87**); Compound 4: 2-(hydroxymethyl)anthraquinone (peak **44**, reference compound). .... 23~~24~~

Fig. S5 A: Isoflavones tentatively assigned in HLQE samples. B: Proposed MS^2 fragmentation pathways of representative compound (calycosin-7-*O*-*β*-D-glucoside). .... 24~~25~~

Fig. S6 A: 4-methoxyisoflavans and pterocarpenoids tentatively assigned in HLQE samples. B: Proposed MS^2 fragmentation pathways of representative compound (astragalinin A). .... 25~~26~~

Fig. S7 A: Saponins tentatively assigned in HLQE samples. B: Proposed MS^2 fragmentation pathways of representative compound (isoastragaloside I). .... 26~~27~~

Fig. S8 A: Isoflavans tentatively assigned in HLQE samples. B: Proposed MS^2 fragmentation pathways of representative compounds (isomucronulatol-7-*O*-*β*-D-glucopyranoside and isomucronulatol). .... 27~~28~~

Fig. S9 A: Polymethoxyflavones tentatively assigned in HLQE samples. B: Proposed MS^2 fragmentation pathways of representative compound (nobiletin). .... 28~~29~~

Fig. S10 A: Anthraquinones tentatively assigned in HLQE samples. B: Proposed MS^2 fragmentation pathways of representative compound (astragquinone). .... 29~~30~~

Fig. S11 A: Fatty acids tentatively assigned in HLQE samples. B: Proposed MS^2 fragmentation pathways of representative compound (13-*E*, *E*-oxooctadeca-9,11-dienoic acid). .... 30~~31~~
Fig. S12 ^{1}H NMR spectrum (500 MHz) of astragalinin A (peak **47**) in CD_3OD. .... 31~~32~~
Fig. S13 ^{13}C NMR spectrum (125 MHz) of astragalinin A (peak **47**) in CD_3OD. .... 31~~32~~
Fig. S14 ^{1}H-^{1}H COSY spectrum (500 MHz) of astragalinin A (peak **47**) in CD_3OD. .... 32~~33~~
Fig. S15 HSQC spectrum (500 MHz) of astragalinin A (peak **47**) in CD_3OD. .... 32~~33~~
Fig. S16 HMBC spectrum (500 MHz) of astragalinin A (peak **47**) in CD_3OD. .... 33~~34~~
Fig. S17 ROESY spectrum (500 MHz) of astragalinin A (peak **47**) in CD_3OD. .... 33~~34~~
Fig. S18 UV spectrum of astragalinin A (peak **47**). .... 34~~35~~
Fig. S19 IR spectrum of astragalinin A (peak **47**). .... 34~~35~~
Fig. S20 CD spectrum of astragalinin A (peak **47**). .... 35~~36~~
Fig. S21 ECD spectrum of astragalinin A (peak **47**). .... 35~~36~~
Fig. S22 ^{1}H NMR spectrum (500 MHz) of astragalinin B (peak **54**) in CD_3OD. .... 36~~37~~
Fig. S23 ^{13}C NMR spectrum (125 MHz) of astragalinin B (peak **54**) in CD_3OD. .... 36~~37~~
Fig. S24 ^{1}H-^{1}H COSY spectrum (500 MHz) of astragalinin B (peak **54**) in CD_3OD. .... 37~~38~~
Fig. S25 HSQC spectrum (500 MHz) of astragalinin B (peak **54**) in CD_3OD. .... 37~~38~~
Fig. S26 HMBC spectrum (500 MHz) of astragalinin B (peak **54**) in CD_3OD. .... 38~~39~~
Fig. S27 ROESY spectrum (500 MHz) of astragalinin B (peak **54**) in CD_3OD. .... 38~~39~~
Fig. S28 UV spectrum of astragalinin B (peak **54**). .... 39~~40~~
Fig. S29 IR spectrum of astragalinin B (peak **54**). .... 39~~40~~
Fig. S30 CD spectrum of astragalinin B (peak **54**). .... 40~~41~~
Fig. S31 ECD spectrum of astragalinin B (peak **54**). .... 40~~41~~
Fig. S32 ^{1}H NMR spectrum (500 MHz) of astragquinone (peak **87**) in CD_3OD. .... 41~~42~~
Fig. S33 ^{13}C NMR spectrum (125 MHz) of astragquinone (peak **87**) in CD_3OD. .... 41~~42~~
Fig. S34 ^{1}H-^{1}H COSY spectrum (500 MHz) of astragquinone (peak **87**) in CD_3OD. .... 42~~43~~
Fig. S35 HSQC spectrum (500 MHz) of astragquinone (peak **87**) in CD_3OD. .... 42~~43~~
Fig. S36 HMBC spectrum (500 MHz) of astragquinone (peak **87**) in CD_3OD. .... 43~~44~~
Fig. S37 ROESY spectrum (500 MHz) of astragquinone (peak **87**) in CD_3OD. .... 43~~44~~
Fig. S38 UV spectrum of astragquinone (peak **87**). .... 44~~45~~
Fig. S39 IR spectrum of astragquinone (peak **87**). .... 44~~45~~
Fig. S40 CD spectrum of astragquinone (peak **87**). .... 45~~46~~
Fig. S41 ECD spectrum of astragquinone (peak **87**). .... 45~~46~~
Figure S42 Dose–response curves of compound 1 on TNF-α and IL-6 production in LPS-stimulated RAW264.7 cells .... 46~~47~~
Figure S43 Dose–response curves of compound 2 on TNF-α and IL-6 production in LPS-stimulated RAW264.7 cells .... 46~~47~~
Figure S44 Dose–response curves of compound 3 on TNF-α and IL-6 production in LPS-stimulated RAW264.7 cells .... 46~~47~~

Figure S45 Dose–response curves of compound 4 on TNF-α and IL-6 production in LPS-stimulated RAW264.7 cells .... 46~~47~~
References .... 47~~48~~

Table S1 Cell survival rate and inhibition of TNF-α and IL-6 by different polarity fractions of Honglanqi at 10 μg/mL in LPS-stimulated RAW264.7 cells.

Sample	Cell survival rate (%)	TNF-*α* inhibition (%)	IL-6 inhibition (%)
HLQE	143.1 ± 15.6	52.2 ± 7.8	55.6 ± 3.6
HLQP	157.1 ± 16.9	31.5 ± 15.3	17.2 ± 6.0
HLQW	107.2 ± 3.0	-8.6 ± 7.5	5.8 ± 5.5

Note: Cell viability and inhibition rates of TNF-*α* and IL-6 were calculated for the petroleum ether fraction (HLQP), ethyl acetate fraction (HLQE), and aqueous fraction (HLQW) at a non-cytotoxic concentration of 10 μg/mL. Cell viability is expressed as percentage relative to the untreated control group. Cytokine inhibition rates were calculated relative to the LPS-stimulated model group. Data are presented as Mean ± SEM (n=6).

Table S2 Chemical constituents isolated from Huangqi.

Types	Name	Formula	Molecular Weight
Triterpenoid saponins and sapogenins			
1	Astragaloside A	$C_{41}H_{68}O_{14}$	784
2	Astragaloside III	$C_{41}H_{68}O_{14}$	784
3	Huangqiyegenin I	$C_{30}H_{48}O_{5}$	488
4	Huangqiyegenin V	$C_{30}H_{46}O_{6}$	502
5	Isoastragaloside II	$C_{43}H_{70}O_{15}$	826
6	Astragaloside I	$C_{45}H_{72}O_{16}$	868
7	Isoastragaloside I	$C_{45}H_{72}O_{16}$	868
8	Acetylastragaloside I	$C_{47}H_{74}O_{16}$	910
9	Isoastragaloside IV	$C_{41}H_{68}O_{14}$	784
10	Astragaloside VII	$C_{47}H_{78}O_{19}$	946
11	Huangqiyenin A	$C_{36}H_{58}O_{10}$	650
12	Brachyoside B	$C_{36}H_{60}O_{10}$	652
13	Astragaloside II	$C_{43}H_{70}O_{15}$	826
14	Astragaloside VI	$C_{47}H_{78}O_{19}$	946
15	Astragaloside V	$C_{47}H_{78}O_{19}$	946
16	Cyclocephaloside II	$C_{43}H_{70}O_{15}$	826
17	Astrolanosaponin C	$C_{36}H_{58}O_{10}$	650
18	Astramembranoside A	$C_{42}H_{70}O_{15}$	814
19	Agroastragaloside IV	$C_{49}H_{80}O_{20}$	988
20	Agroastragaloside III	$C_{51}H_{82}O_{21}$	1030
21	Cycloaraloside A	$C_{36}H_{60}O_{10}$	652
22	Astramembrannin II	$C_{35}H_{58}O_{9}$	622
23	Huangqiyenin K	$C_{37}H_{60}O_{10}$	664
24	Astraverrucin II	$C_{38}H_{62}O_{11}$	694
25	Astrolanosaponin A_1	$C_{42}H_{70}O_{15}$	814
26	Cycloaraloside E	$C_{42}H_{70}O_{15}$	814
27	Astrolanosaponin A_2	$C_{44}H_{72}O_{16}$	856
28	Astrolanosaponin B	$C_{42}H_{68}O_{15}$	812
29	Astrolanosaponin E	$C_{36}H_{60}O_{11}$	668
30	Astrolanosaponin D	$C_{36}H_{60}O_{10}$	652
31	4´-*O*-crotonyl-astragaloside I	$C_{49}H_{76}O_{17}$	936
32	Huangqiyenin D	$C_{38}H_{62}O_{11}$	694
33	Astralanosaponin F	$C_{43}H_{70}O_{15}$	826
34	Astralanosaponin G	$C_{41}H_{66}O_{14}$	782
35	Astralanosaponin H	$C_{43}H_{70}O_{15}$	826
36	Astralanosaponin I	$C_{43}H_{70}O_{15}$	826
37	Astralanosaponin J	$C_{45}H_{72}O_{16}$	868
38	Astralanosaponin K	$C_{41}H_{68}O_{13}$	768
39	Huangqiyesaponin A	$C_{36}H_{63}O_{10}$	678
40	Huangqiyesaponin B	$C_{41}H_{64}O_{14}$	778
41	Huangqiyesaponin C	$C_{36}H_{60}O_{11}$	668
42	Huangqiyesaponin D	$C_{38}H_{60}O_{11}$	692

43	Huangqiyesaponin E	$C_{38}H_{60}O_{11}$	692
44	Huangqiyesaponin F	$C_{38}H_{60}O_{11}$	692
45	Huangqiyesaponin G	$C_{40}H_{62}O_{12}$	734

Table S2 (*continued*)

Types	Name	Formula	Molecular Weight
46	Huangqiyesaponin H	$C_{36}H_{58}O_{11}$	666
47	Huangqiyesaponin I	$C_{38}H_{62}O_{10}$	678
48	Huangqiyesaponin J	$C_{38}H_{62}O_{10}$	678
49	Huangqiyesaponin K	$C_{35}H_{58}O_{8}$	606
50	Huangqiyesaponin L	$C_{36}H_{60}O_{9}$	636
51	Huangqiyesaponin M	$C_{40}H_{64}O_{11}$	720
52	Huangqiyesaponin N	$C_{40}H_{64}O_{12}$	736
53	Huangqiyesaponin O	$C_{35}H_{56}O_{9}$	620
54	Huangqiyenin B	$C_{36}H_{60}O_{10}$	652
55	Mongholicoside II	$C_{38}H_{62}O_{11}$	694
56	Cyclocanthoside A	$C_{35}H_{60}O_{9}$	624
57	Astramembranoside B	$C_{41}H_{70}O_{14}$	786
58	Cyclocanthoside E	$C_{41}H_{70}O_{14}$	786
59	Agroastragaloside II	$C_{43}H_{72}O_{15}$	828
60	Agroastragaloside I	$C_{45}H_{74}O_{16}$	870
61	Agroastragaloside V	$C_{43}H_{72}O_{14}$	812
62	Huangqiyegenin VI	$C_{30}H_{48}O_{5}$	488
63	Mongholicoside I	$C_{36}H_{60}O_{9}$	636
64	Huangqiyenin L	$C_{43}H_{70}O_{14}$	810
65	Aleksandroside I	$C_{36}H_{62}O_{10}$	654
66	Alexandroside I	$C_{36}H_{62}O_{10}$	654
67	Mongholicoside A	$C_{36}H_{62}O_{11}$	670
68	Mongholicoside B	$C_{36}H_{60}O_{11}$	668
69	20,24-non-epoxy-astragaloside III	$C_{41}H_{70}O_{15}$	802
70	20,24-non-epoxyastragaloside IV	$C_{41}H_{70}O_{15}$	802
71	Cyclounifolioside D	$C_{38}H_{64}O_{11}$	696
72	3-O-[*α*-L-rhamnopyranosyl-(1→2)-*β*-D-xylopyranosyl]-6-*O*-*β*-D-glucopyranosyl-2,4-*O*-*α*-L-arabinopyranosyl-16-*O*-acetoxy-3*β*,6*α*,16*β*,24(*S*),25-pentahydroxycycloartane	$C_{54}H_{90}O_{23}$	1106
73	Astralanosaponin L	$C_{47}H_{76}O_{17}$	912
74	Huangqiyenin F	$C_{40}H_{64}O_{12}$	736
75	Huangqiyenin G	$C_{40}H_{62}O_{13}$	750
76	Huangqiyenin E	$C_{42}H_{66}O_{14}$	794
77	Huangqiyenin E	$C_{40}H_{60}O_{13}$	748
78	Huangqiyenin J	$C_{40}H_{62}O_{12}$	734
79	Huangqiyenin I	$C_{40}H_{64}O_{13}$	752
80	Huangqiyenin M	$C_{40}H_{64}O_{13}$	752
81	Huangqiyenin N	$C_{38}H_{60}O_{12}$	708
82	Huangqiyenin O	$C_{36}H_{60}O_{11}$	668
83	Huangqiyenin P	$C_{38}H_{60}O_{12}$	708
84	Huangqiyenin Q	$C_{38}H_{62}O_{12}$	732
85	Huangqiyenin R	$C_{38}H_{62}O_{12}$	732
86	Huangqiyenin S	$C_{38}H_{60}O_{12}$	708

87	Huangqiyenin T	$C_{36}H_{60}O_{10}$	674
88	Huangqiyenin U	$C_{36}H_{55}O_{10}$	646
89	Huangqiyenin V	$C_{36}H_{63}O_{10}$	678

Table S2 (*continued*)

Types	Name	Formula	Molecular Weight
90	Huangqiyenin W	$C_{41}H_{64}O_{14}$	778
91	Huangqiyenin X	$C_{36}H_{60}O_{11}$	668
92	Azukisaponin V	$C_{48}H_{78}O_{18}$	942
93	Astragaloside VIII	$C_{47}H_{76}O_{17}$	912
94	Soyasaponin I	$C_{48}H_{78}O_{18}$	942
95	Soyasapogenol B	$C_{30}H_{50}O_3$	458
96	Soyasapogenol E	$C_{30}H_{48}O_3$	456
97	(3*β*,22*β*)-olean-12-ene-3,22,24,29-tetrol	$C_{30}H_{50}O_4$	474
98	(3*β*,21*α*)-olean-12-ene-3,21,24-triol	$C_{30}H_{50}O_3$	458
99	Azukisaponin V methyl ester	$C_{49}H_{80}O_{18}$	956
100	Astragaloside VIII methyl ester	$C_{48}H_{78}O_{17}$	926
101	Robinioside F	$C_{48}H_{78}O_{19}$	958
102	Astroolesaponin C_1	$C_{43}H_{68}O_{16}$	840
103	Robinioside B	$C_{48}H_{76}O_{20}$	972
104	Astroolesaponin C_2	$C_{49}H_{78}O_{20}$	986
105	Astroolesaponin D	$C_{48}H_{74}O_{19}$	954
106	Astroolesaponin B	$C_{48}H_{78}O_{19}$	958
107	Astroolesaponin F	$C_{48}H_{74}O_{19}$	954
108	Astroolesaponin A	$C_{48}H_{76}O_{18}$	940
109	Cloversaponin III	$C_{42}H_{64}O_{16}$	824
110	Astroolesaponin E1	$C_{42}H_{64}O_{17}$	840
111	Astroolesaponin E2	$C_{41}H_{62}O_{16}$	810
112	Astraisoolesaponin B	$C_{42}H_{62}O_{16}$	822
113	Astraisoolesaponin A2	$C_{42}H_{62}O_{17}$	838
114	Astraisoolesaponin A3	$C_{41}H_{60}O_{16}$	808
115	Astraisoolesaponin A1	$C_{48}H_{72}O_{21}$	984
116	Pisumsaponins I	$C_{51}H_{80}O_{21}$	1028
117	Soyasapogenol B-3-*O*-*β*-D-glucopyranosyl-(1→2)-*β*-D-glucopyranoside-(1→4)-*α*-L-rhamnopyranosyl	$C_{48}H_{78}O_{18}$	942
118	Astraoleanoside A	$C_{48}H_{76}O_{19}$	956
119	Astraoleanoside B	$C_{48}H_{72}O_{20}$	968
120	Astraoleanoside C	$C_{42}H_{60}O_{17}$	836
121	Astraoleanoside D	$C_{48}H_{70}O_{21}$	982
122	Astraoleanoside E	$C_{54}H_{86}O_{24}$	1118
123	Astraoleanoside F	$C_{48}H_{76}O_{20}$	972
124	Astraoleanoside G	$C_{54}H_{86}O_{24}$	1118
125	Astraoleanoside H	$C_{36}H_{56}O_{11}$	664
126	Astraoleanoside I	$C_{42}H_{66}O_{17}$	842
127	Astraoleanoside K	$C_{48}H_{76}O_{21}$	988
128	Astraoleanoside L	$C_{48}H_{74}O_{21}$	986
129	Astraoleanoside M	$C_{48}H_{76}O_{19}$	956
130	Astraoleanoside N	$C_{54}H_{86}O_{24}$	1118
131	Astraoleanoside O	$C_{48}H_{74}O_{21}$	986

132	Astraoleanoside P	$C_{54}H_{84}O_{25}$	1132
133	Azukisaponin II	$C_{42}H_{68}O_{14}$	796
134	Astraoleanoside A	$C_{48}H_{76}O_{19}$	956

Table S2 (*continued*)

Types	Name	Formula	Molecular Weight
135	3-*O*-[*α*-L-rhamnopyranosyl-(1→2)-*β*-D -glucopyranosyl-(1→2)-*β*-D-glucuronopy-ranosyl]-29-*O*-*β*-D-glucopyranosyl-3*β*,22*β*,24,29-tetrahydroxyolean-12-ene	$C_{54}H_{88}O_{24}$	1120
136	3-*O*-[*β*-D-glucopyranosyl-(1→2)-*β*-D-glucur-onopyranosyl]-3*β*,22*β*,24-trihydroxyolean-12-en-29-oic acid	$C_{42}H_{66}O_{16}$	826
137	3-*O*-[*α*-L-rhamnopyranosyl-(1→2)-*β*-D -glucopyranosyl-(1→2)-*β*-D-glucuronopyra-nosyl]-29-*O*-*β*-D-glucopyranosyl-3*β*,22*β*,24-trihydroxyolean-12-en-29-oic acid	$C_{54}H_{86}O_{25}$	1134
138	3-*O*-[*α*-Lrhamnopyranosyl-(1→2)-*β*-D-gluco-pyranosyl-(1→2)-*β*-D-glucuronopyranosyl]-3*β*,24-dihydroxyolean-12-en-22-oxo-29-oic acid	$C_{48}H_{74}O_{20}$	970
139	Astraoleanoside B	$C_{48}H_{72}O_{20}$	968
140	Astraoleanoside C	$C_{42}H_{60}O_{17}$	836
141	Astraoleanoside D	$C_{48}H_{70}O_{21}$	982
142	Astramalabaricoside A	$C_{50}H_{87}NO_{20}$	1021
143	Astramalabaricoside B	$C_{55}H_{95}NO_{24}$	1153
144	Astramalabaricoside C	$C_{60}H_{103}NO_{28}$	1285
145	Astramalabaricoside D	$C_{57}H_{97}NO_{25}$	1195
146	Astramalabaricoside E	$C_{50}H_{87}NO_{21}$	1037
147	Astramalabaricoside F	$C_{47}H_{82}O_{20}$	966
148	Astramalabaricoside G	$C_{55}H_{95}NO_{25}$	1169
149	Astramalabaricoside H	$C_{60}H_{103}NO_{29}$	1301
150	Astramalabaricoside I	$C_{57}H_{97}NO_{26}$	1211
151	Astramalabaricoside J	$C_{53}H_{92}O_{25}$	1128
152	Astramalabaricoside K	$C_{61}H_{105}NO_{30}$	1331
153	Astramalabaricoside L	$C_{66}H_{113}NO_{34}$	1463
154	Astramalabaricoside M	$C_{54}H_{90}O_{26}$	1154
155	Astramalabaricoside N	$C_{62}H_{103}NO_{31}$	1357
156	Astramalabaricoside O	$C_{48}H_{80}O_{21}$	992
157	Astramalabaricoside P	$C_{56}H_{93}NO_{26}$	1195
158	Astramalabaricoside Q	$C_{61}H_{101}NO_{30}$	1327
159	Astramalabaricoside R	$C_{58}H_{95}NO_{27}$	1237
160	Astramalabaricoside S	$C_{58}H_{95}NO_{27}$	1237
162	Astramalabaricoside T	$C_{56}H_{93}NO_{25}$	1179
163	Lupeol	$C_{30}H_{50}O$	426
164	Ursolic acid	$C_{30}H_{48}O_3$	456
165	lupenone	$C_{30}H_{48}O$	424
Flavonoids			
166	4',7-dihydroxyflavone	$C_{15}H_{10}O_4$	254
167	3',4',7-trihydroxyflavone	$C_{15}H_{10}O_5$	270
168	Oroxylin-A	$C_{16}H_{12}O_5$	284
169	Wogonin	$C_{16}H_{12}O_5$	284

170	3′,7-dihydroxy-5′,6-dimethoxy isoflavone	$C_{17}H_{14}O_6$	314
171	7-Methoxy-3′,4′,5-trihydroxy isoflavone	$C_{16}H_{12}O_6$	300
172	Astramonghoside A	$C_{28}H_{32}O_{15}$	608

Table S2 (*continued*)

Types	Name	Formula	Molecular Weight
173	Astramonghoside B	$C_{31}H_{34}O_{18}$	694
174	(−)-Liquiritigenin	$C_{15}H_{12}O_4$	256
175	4'-hydroxyflavanone 7-*O*-*β*-D-glucoside	$C_{21}H_{22}O_9$	418
176	Quercetin	$C_{15}H_{10}O_7$	302
177	Quercetin 3-*O*-*β*-D-glucopyranoside	$C_{21}H_{20}O_{12}$	464
178	Rhamnocitrin 3-*O*-*β*-D-glucopyranoside	$C_{22}H_{22}O_{11}$	462
179	Complanatuside	$C_{28}H_{32}O_{16}$	624
180	Tiliroside	$C_{30}H_{26}O_{13}$	594
181	Isorhamnetin 3-*O*-(6-*O*-*α*-L-fucopyranosyl) -*β*-D-glucopyranoside	$C_{28}H_{32}O_{16}$	624
182	Isorhamnetin 3-*O*-(6-*O*-*α*-L-fucopyranosyl) -*β*-D-galactopyranoside	$C_{28}H_{32}O_{16}$	624
183	Isorhamnetin 3-*O*-(4-*O*-[*E*]-coumaroyl-3,6-*α*-L-*O*-fucopyranosyl)-*β*-D-galactopyranoside	$C_{43}H_{48}O_{22}$	916
184	Rhamnocitrin 3-*O*-*β*-D-glucopyranoside (1→2) -*β*-D-apiofuranosyl	$C_{27}H_{30}O_{15}$	594
185	Rhamnocitrin 3-*O*-*β*-neohesperidoside	$C_{28}H_{32}O_{15}$	608
186	Kaempferol	$C_{15}H_{10}O_6$	286
187	Kaempferol 3-*O*-*β*-D-glucoside	$C_{21}H_{20}O_{11}$	448
188	Kaempferol 3-*O*-(2-*O*-*α*-L-rhamnopyranosy -l)-*β*-D-glucopyranoside	$C_{27}H_{30}O_{15}$	594
189	Astraflavonoid A	$C_{36}H_{36}O_{18}$	756
190	Rhamnocitrin 3-*O*-neohesperoside	$C_{28}H_{32}O_{15}$	608
191	Kaempferol 3,7-di-*O*-*β*-D-glucopyranoside	$C_{27}H_{30}O_{16}$	610
192	Astraflavonoid B	$C_{43}H_{48}O_{23}$	932
193	Isorhamnetin	$C_{16}H_{12}O_7$	316
194	Quercetin 3-*O*-*β*-D-neospheroside	$C_{27}H_{30}O_{16}$	610
195	Isorhamnetin 3-*O*-*β*-D-glucoside	$C_{22}H_{22}O_{12}$	478
196	Kaempferol 4'-methoxyl-3-*O*-*β*-D-glucoside	$C_{22}H_{22}O_{11}$	462
197	Quercetin 3-*O*-[*β*-D-apiofuranosyl-(1→2)-*β*-D-glucopyranoside]	$C_{26}H_{28}O_{16}$	596
198	Quercetin 3,4′-diglucoside	$C_{27}H_{30}O_{17}$	626
199	Kaempferol 3,4′-diglucoside	$C_{27}H_{30}O_{16}$	610
200	Astraside A	$C_{35}H_{34}O_{18}$	742
201	Astraside B	$C_{35}H_{34}O_{18}$	742
202	Astraside C	$C_{36}H_{36}O_{19}$	772
203	3-*O*-[5'''-*O*-feruloyl-*β*-D-apiofuranosyl (1'''→2'')-*β*-D-glucopyranosyl] rhamnocitrin	$C_{37}H_{38}O_{18}$	770
204	Isorhamnetin 3-*O*-[(5-*O*-trans-feruloyl-*β*-D-apiofuranosyl)-(1→2)-*β*-D-glucopyranoside	$C_{37}H_{38}O_{19}$	786
205	Formononetin	$C_{16}H_{12}O_4$	268
206	Formononetin 7-*O*-*β*-D-glucoside	$C_{22}H_{22}O_9$	430
207	6''-Acetyl-ononin	$C_{24}H_{24}O_{10}$	472
208	Calycosin	$C_{16}H_{12}O_5$	284
209	Calycosin 7-*O*-*β*-D-glucopyranoside	$C_{22}H_{22}O_{10}$	446
210	Calycosin 7-*O*-*β*-D-(6-*O*-acetly) -	$C_{24}H_{24}O_{11}$	488

	glucopyranoside		
211	Genistein	$C_{15}H_{10}O_5$	270
212	Genistin	$C_{21}H_{20}O_{10}$	432

Table S2 (*continued*)

Types	Name	Formula	Molecular Weight
213	Pratensein	$C_{16}H_{12}O_6$	300
214	Odoratin 7-*O*-*β*-D-glucopyranoside	$C_{23}H_{24}O_{11}$	476
215	3'-methoxy-5'-hydroxy-isoflavone 7-*O*-*β*-D-glucoside	$C_{22}H_{22}O_{10}$	446
216	Glycitein	$C_{16}H_{12}O_5$	284
217	Glycitin	$C_{22}H_{22}O_{10}$	446
218	3',7,8-trihydroxy-4'-methoxyisoflavone	$C_{16}H_{12}O_6$	300
219	8,3'-dihydroxy-7,4'-dimethoxyisoflavone	$C_{17}H_{14}O_6$	314
220	7,3'-dihydroxy-8,4'-dimethoxyisoflavone	$C_{17}H_{14}O_6$	314
221	Calycosin 7-*O*-*β*-D-{6''-[(*E*)-but-2-enoyl} -glucopyranoside	$C_{26}H_{26}O_{11}$	514
222	4',7-Dihydroxy-3'-methoxy isoflavone	$C_{16}H_{12}O_5$	284
223	Ammopiptanoside A	$C_{26}H_{26}O_{10}$	498
224	3',7-Dihydroxy-5'-methoxyisoflavone	$C_{16}H_{12}O_5$	284
225	4',5,7-Trihydroxy-3'-methoxyisoflavone	$C_{16}H_{12}O_6$	300
226	Afrormosin	$C_{17}H_{14}O_5$	298
227	Odoratin	$C_{17}H_{14}O_6$	314
228	Pratensein 7-*O*-*β*-D-glucopyranoside	$C_{22}H_{22}O_{11}$	462
229	Sissotrin	$C_{22}H_{22}O_{10}$	446
230	Sophorabioside	$C_{27}H_{30}O_{14}$	578
231	Calycosin 7-*O*-*β*-D-{6''-[(*E*)-but-2-enoyl} -galcopyranoside	$C_{26}H_{26}O_{11}$	514
232	Cajanin	$C_{16}H_{12}O_6$	300
233	Santal	$C_{16}H_{12}O_6$	300
234	(−) -methylnissolin	$C_{17}H_{16}O_5$	300
235	(6a*R*,11a*R*) -3,9,10-Tri-methoxypterocarpan	$C_{18}H_{18}O_5$	314
236	(−) -Methylinissolin 3-*O*-*β*-D-glucoside	$C_{23}H_{26}O_{10}$	462
237	(−) -Methylinissolin 3-*O*-*β*-D-(6'-acetyl) -glucoside	$C_{25}H_{28}O_{11}$	504
238	(6a*R*,11a*R*)-3,9-Dimethoxy-10-hydroxypterocarpan	$C_{17}H_{16}O_5$	300
239	(6a*R*,11a*R*)-3,8-dihydroxy-9,10-dimethoxypterocarpan	$C_{17}H_{16}O_6$	316
240	Vesticarpan	$C_{16}H_{14}O_5$	286
241	Licoagroside D	$C_{22}H_{24}O_{10}$	448
242	(−)-Methylinissolin 3-*O*-*β*-D-{6'-*O*-[(*E*)-but -2-enoyl]}-glucoside	$C_{27}H_{30}O_{11}$	530
243	Trifolinhizin	$C_{22}H_{22}O_{10}$	446
244	Astrapterocarpanoside A	$C_{28}H_{34}O_{14}$	594
245	Astrapterocarpanoside B	$C_{34}H_{44}O_{19}$	756
246	Astrapterocarpanoside C	$C_{38}H_{42}O_{17}$	770
247	Astrapterocarpanoside D	$C_{38}H_{42}O_{17}$	770
248	Astrapterocarpanoside E	$C_{44}H_{52}O_{22}$	932
249	Astrapterocarpanoside F	$C_{44}H_{52}O_{22}$	932
250	Astrapterocarpanoside G	$C_{41}H_{48}O_{21}$	876
251	Astrapterocarpanoside H	$C_{56}H_{64}O_{28}$	1184

252	Astrapterocarpanoside I	$C_{28}H_{34}O_{15}$	610
253	Astrapterocarpanoside J	$C_{22}H_{24}O_{10}$	448
254	Astrapterocarpanoside K	$C_{27}H_{32}O_{14}$	580

Table S2 (*continued*)

Types	Name	Formula	Molecular Weight
255	(-)-maackiain	$C_{16}H_{12}O_5$	284
256	Daidzein	$C_{15}H_{12}O_4$	256
257	(3*R*)-8,2'-Dihydroxy-7,4'-dimethoxyisoflavan	$C_{17}H_{18}O_5$	302
258	Isomucronulatol	$C_{17}H_{18}O_5$	302
259	7-*O*-methylisomucronulatol	$C_{18}H_{20}O_5$	316
260	Isomucronulatol-7-*β*-*O*-glucoside	$C_{23}H_{28}O_{10}$	464
261	Isomucronulatol 7,2'-di-*O*-*β*-glucoside	$C_{29}H_{38}O_{15}$	626
262	(3*R*)-7,2',3'-Trihydroxy-4'-methoxy-isoflavane	$C_{16}H_{16}O_5$	288
263	(*R*)-3-(5-Hydroxy-2,3,4-trimethoxyphenyl) -chroman-7-ol	$C_{18}H_{20}O_6$	332
264	Sphaerophyside SB	$C_{23}H_{28}O_{10}$	464
265	Pendulone	$C_{17}H_{16}O_6$	316
266	(3*R*) -(−) -Mucronulatol 7-*O*-*β*-D-glucoside	$C_{23}H_{28}O_{10}$	464
267	6''-*O*-Acetyl-(3*R*)-2'-hydroxy-3',4'-dimethoyl-isoflavan 7-*O*-*β*-D-glucopyranoside	$C_{25}H_{30}O_{11}$	506
268	Astramembraflavane A	$C_{20}H_{22}O_7$	374
269	(3*R*)-7-hydroxy-3′,4′-dimethoxyisoflavane	$C_{17}H_{18}O_4$	286
270	(*R*)-5-(6-hydroxychroman-3-yl)-2,3-dimethoxybenzene-1,4-diol	$C_{17}H_{18}O_6$	318
271	Millepurpan	$C_{18}H_{20}O_6$	332
272	Astraciceran	$C_{17}H_{16}O_5$	300
273	3'-Hydroxy-2',4'-dimethoxyisoflavan 6-*O*-*β*-D -glucopyranoside	$C_{23}H_{28}O_{11}$	480
274	Astraflavonoid C	$C_{23}H_{28}O_{11}$	480
275	Astramemside A	$C_{27}H_{32}O_{11}$	532
276	(3*R*)-astragaluquinone	$C_{17}H_{16}O_6$	316
277	Astragaisoflavan A	$C_{17}H_{18}O_6$	318
278	3,2'-Dihydroxyl-3',4'-methoxyisoflavanone 7-*O*-*β*-D-glucoside	$C_{23}H_{28}O_{11}$	480
279	(3*R*,4*R*)-4,7-hydroxy-2',3'-dimethoxyisoflavane 4'-*O*-*β*-D-glucoside	$C_{23}H_{28}O_{11}$	480
280	2',5'-Dicarbonyl-3',4'-dimethoxyisoflavanequinone 7-*O*-*β*-D-glucoside	$C_{23}H_{26}O_{11}$	478
281	Echinatin	$C_{16}H_{14}O_4$	270
282	Licochalcone B	$C_{16}H_{14}O_5$	286
283	2',4,4'-Trihydroxychalcone	$C_{15}H_{12}O_4$	256
284	2'-Methoxyisoliquiritigenin	$C_{16}H_{14}O_4$	270
285	4,4',6'-Trihydroxychalcone	$C_{15}H_{12}O_4$	256
286	4-Methoxy-4',6'-dihydroxychalcone	$C_{16}H_{14}O_4$	270
287	4,4'-Dimethyl-6'-hydroxychalcone	$C_{17}H_{16}O_2$	252
288	Loureirin A	$C_{17}H_{18}O_4$	286
289	(*Z*)-2,4-dimethoxy-4-hydroxychalcone	$C_{17}H_{16}O_4$	284
290	Sophorophenolone	$C_{16}H_{10}O_6$	298
Others			

291	Guanosine	$C_{10}H_{14}N_6O_4$	282
292	Dehydrodiconiferyl alcohol 4, *γ*'-di-*O*-*β*-D-glucopyranoside	$C_{32}H_{42}O_{16}$	682

Table S2 (*continued*)

Types	Name	Formula	Molecular Weight
294	*β*-Sitosterol	$C_{29}H_{50}O$	414
295	*β*-Daucosterol	$C_{35}H_{60}O_6$	576
296	Ciwujiatone	$C_{22}H_{26}O_9$	434
297	(+)-neoolivil	$C_{20}H_{24}O_7$	376
298	(-)-secoisolariciresinol	$C_{20}H_{26}O_6$	362
299	cis-dehydrodiconiferyl alcohol	$C_{20}H_{22}O_6$	358
300	(2-cis, 4-trans)-abscisic acid	$C_{15}H_{20}O_4$	264
301	Astramemoside A	$C_{18}H_{22}O_{11}$	413
302	*α*-DDB (Dimethyl 4,4'-dimethoxy-5,6,5',6'-di-(methylenedioxy)-biphenyl-2,2'-dicarboxylate)	$C_{20}H_{18}O_{10}$	418
303	Adenosine (Adenineriboside)	$C_{10}H_{13}N_5O_4$	267
304	3-(*β*-D-Ribofuranosyl) -2,3-dihydro-6*H*-1,3-oxazine-2,6-dione	$C_9H_{11}NO_7$	245
305	Uridine	$C_9H_{12}N_2O_6$	244
306	Adenine	$C_5H_5N_5$	135
307	4-Hydroxycinnamic acid	$C_9H_8O_3$	164
308	Gallic acid	$C_7H_6O_5$	170
309	Emodin	$C_{15}H_{10}O_5$	270
310	Gentisin	$C_{14}H_{10}O_5$	258
311	Maltol-*β*-D-glucopyranoside	$C_{12}H_{16}O_8$	288
312	2,6-Dimethoxy-4-hydroxyphenyl-1-*O*-*β*-D-glucopyranoside	$C_{14}H_{20}O_9$	332
313	Benzyl-*α*-L-arabinopyranosyl (1''→6') -*β*-D-glucopyranoside	$C_{18}H_{26}O_{10}$	402
314	1-Hydroxy-5-methylolbenzol-2-*O*-*β*-D-glucoside	$C_{13}H_{18}O_8$	302
315	D-3-Methoxy-chior-inositol	$C_7H_{14}O_6$	194
316	*γ*-Aminobutanoic acid	$C_4H_9NO_2$	102
317	Butyl 1*H*-imidazole-4-carboxylate	$C_8H_{12}N_2O_2$	168
318	Palmitic acid	$C_{16}H_{32}O_2$	256
319	Monopalmitin	$C_{19}H_{38}O_4$	330
320	L-Asparagine	$C_4H_8N_2O_3$	132

Table S3 Information of metabolites in HLQE identified by UPLC-Q-TOF/MS combined MS/MS-based mass spectral molecular networking.

No.	t_R (min)	PI (*m/z*)	Adduct ions	Error (ppm)	Molecular formula	MS/MS fragments (*m/z*)	Tentative identification	Annotation levels
**1◆**	1.72	485.1069	$[M+Na]^+$	1.9	$C_{22}H_{22}O_{11}$	301.0716, 286.0565, 283.0581, 269.0415, 255.0603, 241.0477, 213.0524, 197.0578, 151.0351, 137.0276	Isoflavone3-(4′ -glucopyranosyl-5′-hydroxy-2′-methoxyphenyl)-7-hydroxy-4*H*-chromen-4-one	c
**2◆**	1.91	417.1182	$[M+H]^+$	-1.0	$C_{21}H_{20}O_9$	417.1182, 255.0660, 199.0739, 137.0193	Daidzin	b
**3◆**	2.21	447.1270	$[M+H]^+$	-4.7	$C_{22}H_{22}O_{10}$	285.0750, 270.0470, 253.0519, 242.0597, 225.0538, 213.0627, 197.0578, 137.0235	3′-Hydroxy-formononetin- 7-*O* -glucoside	c
**4**	2.71	447.1270	$[M+H]^+$	-4.7	$C_{22}H_{22}O_{10}$	285.0750, 270.0528, 253.0519, 242.0597, 225.0538, 213.0549, 197.0578, 169.0620, 137.0235, 134.0363	Calycosin 7-*O*-*β*-D-glucoside*	a
**5**	2.91	477.1395	$[M+H]^+$	-0.4	$C_{23}H_{24}O_{11}$	477.1395, 315.0885, 300.0709, 167.0316, 166.0323, 134.0323	Odoratin 7-*O*-*β*-D-glucopyranoside	b
**6**	3.55	433.1123	$[M+H]^+$	-2.8	$C_{21}H_{20}O_{10}$	433.1123, 271.0602, 253.0519, 243.0629, 215.0711, 197.0528, 153.0184, 119.0482	Genistin	b
**7**	3.80	481.1690	$[M+H]^+$	-4.2	$C_{23}H_{28}O_{11}$	503.1485, 481.1690, 319.1187, 256.0732, 228.0789, 209.0843, 197.0825, 183.0657, 177.0534, 149.0604, 137.0606, 123.0449	Astraflavonoid C	b
**8★**	4.02	485.1069	$[M+Na]^+$	1.9	$C_{22}H_{22}O_{11}$	485.1069, 463.1280, 301.0716, 286.0446, 269.0473, 241.0531, 213.0473, 150.0315, 137.0236	Isoflavone-*O*-glycoside	d
**9◆**	4.60	433.1123	$[M+H]^+$	-2.8	$C_{21}H_{20}O_{10}$	433.1123, 271.0602, 253.0463, 243.0684, 225.0485, 215.0711, 197.0578, 153.0184	Sophoricoside	b
**10**	4.90	485.1069	$[M+Na]^+$	1.9	$C_{22}H_{22}O_{11}$	925.2356, 485.1069, 463.1280, 301.0716, 286.0446, 269.0473, 241.0477, 229.0474, 213.0575, 153.0184, 134.0364	Pratensein 7-*O*-*β*-D-glucopyranoside	b
**11**	5.02	449.1462	$[M+H]^+$	3.1	$C_{22}H_{24}O_{10}$	471.1232, 449.1462, 287.0932, 272.0694, 259.0954, 255.0660, 237.0479, 227.0701, 209.0639, 199.0689, 177.0534, 153.0532, 138.0315	Licoagroside D	b
**12★**	5.44	465.1374	$[M+H]^+$	-4.9	$C_{22}H_{24}O_{11}$	487.1246, 465.1374, 303.0843, 285.0750, 275.0968, 270.0528, 257.0824, 253.0519, 243.0684, 229.0847, 225.0538, 215.0659, 197.0627, 169.0529, 163.0390, 153.0576, 135.0448, 107.0509	Pterocarpane-*O*-glycoside	d
**13**	5.62	489.1387	$[M+H]^+$	-2.0	$C_{24}H_{24}O_{11}$	511.1224, 489.1387, 285.0750, 270.0528, 253.0463, 225.0538, 214.0605, 197.0677, 169.0575, 137.0235, 134.0445	Calycosin 7-*O*-glc-4″-*O*-ace	b
**14★**	5.86	479.1175	$[M+H]^+$	-3.1	$C_{22}H_{22}O_{12}$	479.1175, 317.0659, 302.0435, 153.0184	Isoflavone-*O*-glycoside	d
**15**	6.57	563.1771	$[M+H]^+$	1.1	$C_{27}H_{30}O_{13}$	563.1771, 431.1368, 269.0819, 254.0607, 237.0587, 137.0235	Formononetin-glc-api	b
**16★**	6.63	489.1387	$[M+H]^+$	-2.0	$C_{24}H_{24}O_{11}$	489.1387, 285.0750, 270.0528, 253.0463, 137.0235	Calycosin 7-*O*-glc-2″-*O*-ace isomer	d
**17**	7.42	453.1159	$[M+Na]^+$	-0.7	$C_{22}H_{22}O_9$	453.1159, 431.1368, 269.0819	Ononin*	a

Continued on next page

Table S3 (*continued*)

No.	t_R (min)	PI (*m/z*)	Adduct ions	Error (ppm)	Molecular formula	MS/MS fragments (*m/z*)	Tentative identification	Annotation levels
**18**	8.07	489.1387	$[M+H]^+$	-2.0	$C_{24}H_{24}O_{11}$	511.1224, 489.1387, 285.0750, 270.0586, 253.0519, 242.0542, 225.0538, 214.0605, 213.0524, 197.0578, 137.0235, 134.0363	Calycosin 7-*O*-glc-6″-*O*-ace	b
**19**	8.78	617.1870	$[M+Na]^+$	3.9	$C_{28}H_{34}O_{14}$	617.1870, 595.2021, 463.1583, 301.1082, 286.0862, 269.0819, 241.0914, 226.0634, 207.0636, 191.0759, 179.0730, 167.0726, 152.0446, 134.0363	Astrapterocarpanoside A	b
**20**	9.00	519.1510	$[M+H]^+$	1.3	$C_{25}H_{26}O_{12}$	519.1510, 315.0823, 300.0648, 283.0522, 272.0636, 255.0660, 243.0629, 167.0407	Odoratin-7-*O*-Glc-Ace	b
**21◆**	9.14	301.0716	$[M+H]^+$	1.3	$C_{16}H_{12}O_6$	323.0543, 301.0716, 286.0505, 283.0640, 269.0415, 255.0660, 241.0477, 227.0754, 213.0575, 197.0578, 150.0332, 137.0276	Koparin	b
**22★**	9.41	325.0692	$[M+Na]^+$	1.2	$C_{16}H_{14}O_6$	325.0692, 303.0843, 285.0750, 275.0909, 270.0528, 257.0824, 253.0519, 243.0629, 229.0847, 225.0591, 215.0711, 197.0578, 169.0529, 163.0390, 153.0532, 135.0448, 107.0509	Pterocarpans	d
**23**	10.08	463.1583	$[M+H]^+$	-4.5	$C_{23}H_{26}O_{10}$	501.1136, 485.1457, 463.1583, 301.1082, 286.0862, 273.1096, 269.0819, 241.0914, 226.0634, 207.0636, 191.0710, 167.0726	(-)-Methylnissolin-3-*O*-glucoside*	a
**24**	11.53	285.0750	$[M+H]^+$	-4.6	$C_{16}H_{12}O_5$	307.0557, 285.0750, 270.0528, 253.0519, 225.0538, 213.0575, 197.0578, 169.0666, 137.0235, 134.0363	Calycosin*	a
**25◆**	11.73	485.1069	$[M+Na]^+$	1.9	$C_{22}H_{22}O_{11}$	485.1069, 463.1204, 301.0716, 286.0505, 283.0604, 273.0758, 269.0415, 255.0603, 245.0813, 241.0477, 167.0362, 134.0363	5-Hydroxy-3-[4-hydroxy-2-[(2*S*,3*R*,4*S*,5*S*,6*R*)-3,4,5-trihydroxy-6-(hydroxymethyl)oxan-2-yl]oxyphenyl]-7-methoxychromen-4-one	b
**26**	12.14	465.1754	$[M+H]^+$	-1.5	$C_{23}H_{28}O_{10}$	503.1328, 487.1557, 465.1754, 447.1642, 429.1512, 411.1444, 393.1327, 369.1335, 345.1376, 327.1222, 303.1210, 249.0755, 231.0600, 193.0857, 189.0524, 181.0852, 167.0726, 165.0542, 149.0604, 147.0475, 137.0606, 123.0449	Isomucronulatol-7-*O*-*β*-D-glucopyranoside*	a
**27★**	12.41	487.1557	$[M+Na]^+$	-4.7	$C_{23}H_{28}O_{10}$	503.1328, 487.1557, 465.1754, 429.1512, 411.1444, 303.1210, 193.0857, 181.0852, 167.0680, 161.0586, 149.0604, 133.0642, 123.0449, 118.0407	Isomucronulatol-7-*O*-*β*-D-glucopyranoside isomer	d
**28★**	13.10	505.1322	$[M+H]^+$	-4.8	$C_{24}H_{24}O_{12}$	505.1322, 301.0716, 286.0446, 269.0473, 241.0477, 213.0575, 153.0184	4"-*O*-acetyl-pratensein-7-*O*-*β*-D-glucoside isomer	d
**29◆**	13.40	433.1489	$[M+H]^+$	-2.3	$C_{22}H_{24}O_9$	471.1003, 455.1261, 433.1489, 271.0949, 255.0660, 243.0958, 161.0586, 137.0606, 123.0449, 109.0670	Medicarpin -3-*O*-*β*-D glucopyranoside	b
**30**	13.69	337.0704	$[M+Na]^+$	4.7	$C_{17}H_{14}O_6$	337.0704, 315.0885, 300.0648, 283.0581, 272.0636, 255.0660, 227.0701, 167.0316, 166.0278, 134.0363	Odoratin	b
**31**	14.82	495.1277	$[M+Na]^+$	2.0	$C_{24}H_{24}O_{10}$	495.1277, 473.1422, 269.0819, 254.0551, 237.0479, 137.0235, 118.0369	Formononetin-7-*O*-glc-4"-*O*-ace	b

Continued on next page

Table S3 (*continued*)

No.	t_R (min)	PI (*m/z*)	Adduct ions	Error (ppm)	Molecular formula	MS/MS fragments (*m/z*)	Tentative identification	Annotation levels
**32**	15.20	287.0932	$[M+H]^+$	4.5	$C_{16}H_{14}O_5$	309.0758, 287.0932, 272.0636, 269.0819, 259.0954, 255.0660, 237.0479, 227.0701, 209.0690, 199.0689, 177.0534, 153.0532, 138.0306	Vesticarpan	b
**33★**	15.22	495.1277	$[M+Na]^+$	2.0	$C_{24}H_{24}O_{10}$	495.1277, 473.1422, 269.0819, 254.0607, 237.0533, 137.0193, 118.0369	2''-Acetyl-ononin isomer	d
**34◆**	15.57	299.0900	$[M+H]^+$	-6.4	$C_{17}H_{14}O_5$	299.0900, 284.0657, 266.0593, 238.0623, 210.0677, 151.0394, 134.0404	4´-Hydroxy-7,3´-dimethoxyisoflavone	b
**35**	15.69	447.1270	$[M+H]^+$	-4.7	$C_{22}H_{22}O_{10}$	447.1270, 285.0750, 270.0528, 253.0519, 153.0184, 118.0369	Sissotrin	b
**36◆**	15.77	337.0704	$[M+Na]^+$	4.7	$C_{17}H_{14}O_6$	315.0885, 300.0648, 282.0522, 254.0551, 226.0634, 197.0627, 164.0473, 137.0193	7,2´-Dihydroxy-3´,4´-dimethoxyisoflavone	b
**37**	15.82	505.1322	$[M+H]^+$	-4.8	$C_{24}H_{24}O_{12}$	527.1129, 505.1322, 301.0716, 286.0505, 269.0415, 241.0477, 153.0227, 134.0323	6''-*O*-acetyl-pratensein-7-*O*-*β*-D-glucoside	b
**38★**	18.25	527.1533	$[M+Na]^+$	0.8	$C_{25}H_{28}O_{11}$	527.1533, 505.1718, 301.1082, 286.0862, 273.1096, 269.0761, 241.0859, 226.0634, 207.0636, 191.0710, 167.0726, 152.0490, 134.0363, 106.0409	(-)-Methylinissolin 3-*O*-*β*-D-(6'-acetyl) -glucoside isomer	d
**39**	18.28	515.1569	$[M+H]^+$	3.1	$C_{26}H_{26}O_{11}$	537.1401, 515.1569, 285.0750, 270.0528, 253.0463, 225.0538, 214.0554, 213.0524, 197.0578, 137.0235, 134.0363	Calycosin 7-*O*-*β*-D-{6''-(*E*)-but-2-enoyl} -glucopyranoside	b
**40◆**	18.29	367.0805	$[M+Na]^+$	3.0	$C_{18}H_{16}O_7$	345.0984, 330.0635, 312.0594, 284.0657, 269.0473, 257.0768, 256.0732, 242.0542, 167.0316	3',7-Dihydroxy-2',4',6-trimethoxyisoflavone	c
**41**	18.49	317.1035	$[M+H]^+$	3.2	$C_{17}H_{16}O_6$	339.0831, 317.1035, 299.0961, 289.1125, 284.0657, 257.0824, 255.0660, 238.0623, 229.0847, 227.0701, 207.0585, 197.0627, 183.0657, 179.0730, 167.0726, 163.0390, 155.0672, 135.0448, 123.0449, 107.0509	(6a*R*,11a*R*)-3,8-Dihydroxy-9,10-dimethoxypterocarpan	b
**42**	19.26	299.0900	$[M+H]^+$	-6.4	$C_{17}H_{14}O_5$	321.0709, 299.0900, 284.0657, 283.0460, 266.0535, 255.0660, 238.0623, 237.0587, 137.0235	7-Hydroxy-3',4'-dimethoxyisoflavone	b
**43◆**	19.68	337.0704	$[M+Na]^+$	4.7	$C_{17}H_{14}O_6$	337.0704, 315.0885, 300.0648, 283.0699, 255.0660, 227.0701, 211.0790, 196.0511, 168.0567, 153.0576	Fistulaflavonoid B	c
**44◆**	19.81	261.0537	$[M+Na]^+$	3.4	$C_{15}H_{10}O_3$	261.0537, 239.0735, 221.0586, 211.0790	2-Hydroxymethyl anthraquinone	b
**45**	20.34	495.1277	$[M+Na]^+$	2.0	$C_{24}H_{24}O_{10}$	495.1277, 473.1422, 269.0819, 137.0235	Formononetin-7-*O*-glc-6''-*O*-ace	b
**46◆**	20.60	317.1035	$[M+H]^+$	3.2	$C_{17}H_{16}O_6$	339.0831, 317.1035, 302.0740, 287.0515, 285.0750, 257.0824, 242.0597, 183.0657, 168.0430, 155.0716, 137.0606, 123.0449, 109.0633	3,10-Dihydroxy-7,9-dimethoxypterocarpan	c

Continued on next page

Table S3 (*continued*)

No.	t_R (min)	PI (*m/z*)	Adduct ions	Error (ppm)	Molecular formula	MS/MS fragments (*m/z*)	Tentative identification	Annotation levels
**47★**	20.93	355.1153	$[M+Na]^+$	-1.4	$C_{18}H_{20}O_6$	355.1153, 333.1336, 323.0859, 301.1082, 286.0862, 273.1096, 269.0819, 255.0660, 241.0859, 226.0634, 213.0935, 209.0589, 207.0686, 198.0670, 191.0710, 167.0726, 152.0490, 147.0433, 134.0363, 123.0449, 106.0409	Astragalinin A	d
**48◆**	21.24	503.1564	$[M+H]^+$	2.2	$C_{25}H_{26}O_{11}$	503.1564, 299.0900, 284.0716, 166.0233, 133.0642	6″-*O*-Acetylwistin	c
**49**	21.74	505.1718	$[M+H]^+$	1.6	$C_{25}H_{28}O_{11}$	543.1281, 527.1533, 505.1718, 301.1082, 286.0862, 273.1154, 269.0819, 241.0859, 226.0634, 207.0636, 191.0710, 167.0726, 152.0490, 134.0363, 106.0409	(−) -Methylinissolin 3-*O*-*β*-D-(6'-acetyl) -glucoside	b
**50◆**	21.93	255.0660	$[M+H]^+$	1.2	$C_{15}H_{10}O_4$	277.0501, 255.0660, 237.0533, 227.0701, 209.0589, 199.0739, 181.0662	2-Hydroxy-1-methoxy-anthraquinone	b
**51◆**	22.02	367.1884	$[M+K]^+$	-0.8	$C_{18}H_{32}O_5$	367.1884, 351.2127, 329.2327, 311.2206, 293.2086, 275.2018, 257.1896, 239.1824, 229.1966, 213.1500, 195.1404	9,12,13-Trihydroxy-10(*E*),15(*Z*)-octadecadienoic acid	b
**52**	23.21	529.1674	$[M+Na]^+$	-2.3	$C_{25}H_{30}O_{11}$	529.1674, 507.1911, 489.1699, 471.1690, 453.1534, 411.1444, 393.1327, 369.1335, 327.1222, 303.1210, 273.0689, 231.0654, 213.0524, 193.0857, 189.0524, 181.0946, 167.0726, 147.0433, 123.0488	6''-*O*-Acetyl-(3*R*) -2'-hydroxy-3',4'-dimethoyl-isoflavan -7-*O*-*β*-D-glucopyranoside	b
**53**	23.51	269.0819	$[M+H]^+$	1.9	$C_{16}H_{12}O_4$	291.0609, 269.0819, 254.0607, 253.0519, 237.0553, 226.0634, 213.0884, 197.0578, 137.0235, 118.0445	Formononetin*	a
**54★**	23.72	355.1153	$[M+Na]^+$	-1.4	$C_{18}H_{20}O_6$	355.1153, 323.0859, 333.1336, 301.1082, 286.0862, 273.1096, 269.0819, 255.0716, 241.0859, 226.0634, 213.0885, 209.0589, 207.0636, 198.0720, 191.0710, 167.0726, 152.0490, 147.0433, 134.0363, 123.0449, 106.0409	Astragalinin B	d
**55◆**	23.95	331.1195	$[M+H]^+$	3.9	$C_{18}H_{18}O_6$	331.1195, 316.0890, 299.0900, 285.0750, 267.0649, 255.0603, 237.0750, 221.0796, 197.0825, 182.0574, 169.0895, 163.0390, 154.0651, 137.0606, 123.0449	Micanspterocarpan	c
**56◆**	24.02	373.1273	$[M+H]^+$	-3.8	$C_{20}H_{20}O_7$	373.1273, 358.1063, 357.1013, 343.0809, 329.1050, 327.0522, 315.0885, 299.0535, 267.0649, 211.0688, 181.0189, 167.0680, 165.0587, 163.0705, 153.0184, 123.0488	Isosinensetin	b
**57**	24.24	301.1082	$[M+H]^+$	2.0	$C_{17}H_{16}O_5$	323.0923, 301.1082, 286.0803, 273.1154, 269.0819, 241.0859, 226.0634, 207.0636, 191.0710, 167.0726, 152.0490, 134.0363, 123.0449, 106.0409	(-)-Methylnissolin	b
**58◆**	24.77	331.2476	$[M+H]^+$	-2.4	$C_{18}H_{34}O_5$	369.2011, 353.2340, 331.2476, 313.2357, 295.2251, 277.2141, 259.2087, 213.1500, 195.1355, 173.1180, 155.1066	9,12,13-Trihydroxy-10(*E*)-octadecadienoic acid	c

Continued on next page

Table S3 (*continued*)

No.	t_R (min)	PI (*m/z*)	Adduct ions	Error (ppm)	Molecular formula	MS/MS fragments (*m/z*)	Tentative identification	Annotation levels
**59**	24.97	299.0900	$[M+H]^+$	-6.4	$C_{17}H_{14}O_5$	321.0709, 299.0900, 284.0657, 256.0732, 239.0681, 167.0367, 166.0278, 133.0642, 118.0445	Afrormosin	b
**60★**	25.08	331.2476	$[M+H]^+$	-2.4	$C_{18}H_{34}O_5$	369.2011, 353.2274, 331.2357, 313.2357, 295.2251, 277.2141, 259.2087, 213.1500, 195.1355, 173.1180, 155.1066	9,12,13-Trihydroxy-10(*E*)-octadecadienoic acid isomer	d
**61**	25.93	303.1210	$[M+H]^+$	-7.3	$C_{17}H_{18}O_5$	303.1210, 193.0857, 181.0852, 167.0726, 161.0586, 149.0561, 133.0642, 123.0449, 118.0407	Isomucronulatol*	a
**62**	26.03	499.1615	$[M+H]^+$	2.2	$C_{26}H_{26}O_{10}$	499.1615, 269.0819, 254.0607, 237.0533, 213.0935, 137.0193, 118.0407	Ammopiptanoside A	b
**63◆**	26.93	373.1273	$[M+H]^+$	-3.8	$C_{20}H_{20}O_7$	373.1273, 357.1013, 343.0809, 329.0986, 312.1029, 297.0786, 211.0637, 181.0141, 167.0726, 165.0587, 163.0795	Sinensetin	b
**64◆**	27.04	365.1006	$[M+Na]^+$	1.4	$C_{19}H_{18}O_6$	343.1200, 328.0969, 313.0737, 299.0900, 285.0750, 181.0141, 153.0227, 135.0488, 133.0683	5,6,7,4′-tetramethoxyflavone	b
**65**	28.29	827.4745	$[M+H]^+$	-5.8	$C_{43}H_{70}O_{15}$	827.4745, 809.4702, 783.4457, 765.4426, 647.4098, 629.4013, 607.4149, 589.4114, 571.3926, 553.3886, 475.3805, 457.3665, 439.3536, 421.3486, 175.0592, 143.1049	Astralanosaponin H	b
**66★**	28.71	827.4745	$[M+H]^+$	-5.8	$C_{43}H_{70}O_{15}$	827.4745, 809.4702, 665.4271, 647.4188, 629.4013, 611.3944, 473.3566, 455.3514, 437.3345, 175.0592, 143.1049	Astralanosaponin H isomer	d
**67**	28.90	785.4666	$[M+H]^+$	-2.7	$C_{41}H_{68}O_{14}$	807.4485, 785.4666, 767.4597, 749.4399, 731.4437, 653.4241, 635.4114, 617.4056, 605.4041, 599.4000, 587.3965, 569.3836, 491.3755, 473.3643, 455.3514, 437.3418, 419.3280, 143.1091, 125.0992	Astragaloside IV*	a
**68**	29.23	785.4666	$[M+H]^+$	-2.7	$C_{41}H_{68}O_{14}$	807.4485, 785.4666, 767.4499, 749.4495, 731.4437, 623.4198, 605.4041, 587.3879, 569.3836, 491.3755, 473.3643, 455.3514, 437.3418, 419.3280, 143.1091, 125.0992	Astragaloside III*	a
**69◆**	29.29	281.0836	$[M+H]^+$	7.8	$C_{17}H_{12}O_4$	281.0836, 263.0708, 253.0855, 235.0740, 225.0907, 207.0788, 194.0749, 179.0825, 165.0677	Me anthraquinone-1-acetate	c
**70★**	29.52	355.1153	$[M+Na]^+$	-1.4	$C_{18}H_{20}O_6$	355.1153, 333.1336, 301.1082, 286.0922, 273.1096, 269.0819, 255.0716, 241.0859, 226.0634, 209.0538, 207.0636, 198.0720, 191.0710, 167.0726, 152.0446, 147.0433, 134.0363, 123.0449, 106.0409	Astragalinin isomer	d
**71◆**	29.67	261.0537	$[M+Na]^+$	3.4	$C_{15}H_{10}O_3$	261.0537, 239.0735, 221.0586, 211.0790, 193.0661, 183.0799, 165.0677, 155.0847	2-Hydroxy-3-methylanthraquine	b

Continued on next page

Table S3 (*continued*)

No.	t_R (min)	PI (*m/z*)	Adduct ions	Error (ppm)	Molecular formula	MS/MS fragments (*m/z*)	Tentative identification	Annotation levels
**72**◆	30.36	365.1006	$[M+Na]^+$	1.4	$C_{19}H_{18}O_6$	343.1200, 328.0969, 327.0904, 313.0675, 299.0900, 282.0936, 254.0888, 239.0735, 181.0141, 167.0680, 153.0227, 135.0488, 133.0683	5,7,8,4′-tetramethoxyflavone	b
**73**◆	30.53	403.1404	$[M+H]^+$	2.7	$C_{21}H_{22}O_8$	403.1404, 388.1170, 373.0933, 355.0821, 342.1102, 327.0840, 211.0279, 183.0323, 165.0542, 163.0795	Nobiletin*	a
**74**	31.97	675.4064	$[M+Na]^+$	-3.0	$C_{36}H_{60}O_{10}$	675.4064, 653.4241, 635.4114, 617.4056, 605.4041, 599.4000, 587.3965, 569.3836, 491.3755, 473.3566, 455.3514, 437.3418, 419.3280, 143.1091, 125.0953	Cycloaraloside A	b
**75**	32.09	645.3963	$[M+Na]^+$	-2.5	$C_{35}H_{58}O_9$	645.3963, 623.4110, 605.4041, 587.3879, 569.3752, 491.3676, 473.3643, 455.3514, 437.3418, 419.3352, 143.1091, 125.0953	Astramembrannin II	b
**76**	32.47	849.4581	$[M+Na]^+$	-3.6	$C_{43}H_{70}O_{15}$	849.4581, 827.4745, 809.4702, 791.4559, 773.4485, 667.3716, 665.4271, 653.4241, 647.4188, 629.4013, 617.4056, 611.3944, 599.3914, 593.3831, 491.3755, 473.3643, 455.3514, 437.3418, 419.3352, 175.0592, 157.0502, 143.1091, 125.0992	Astragaloside II*	a
**77**◆	32.88	433.1489	$[M+H]^+$	-2.3	$C_{22}H_{24}O_9$	433.1489, 418.1252, 403.1051, 385.0927, 375.1084, 373.0593, 357.0946, 345.0657, 317.0659, 299.0535, 289.0766, 271.0602, 241.0641, 165.0542	3, 5, 6, 7, 8, 3′, 4′-Heptamethoxyflavone	b
**78**	33.05	849.4581	$[M+Na]^+$	-3.6	$C_{43}H_{70}O_{15}$	827.4745, 809.4702, 791.4658, 665.4271, 647.4188, 629.4013, 611.3944, 473.3566, 455.3514, 437.3345, 419.3280, 175.0592, 143.1049	Astralanosaponin I	c
**79**★	33.92	333.2043	$[M+Na]^+$	0.3	$C_{18}H_{30}O_4$	349.1774, 333.2043, 311.2206, 293.2086, 275.2018, 257.1896, 213.1500, 195.1404	Octadecatrienoic acid isomer	d
**80**◆	33.96	373.1273	$[M+H]^+$	-3.8	$C_{20}H_{20}O_7$	373.1273, 358.1063, 343.0809, 325.0692, 315.0885, 312.0967, 297.0725, 241.0750, 211.0228, 177.0581, 135.0448, 133.0642	Tangeretin	b
**81**◆	34.16	419.1334	$[M+H]^+$	-1.9	$C_{21}H_{22}O_9$	419.1334, 404.1092, 389.0884, 371.0701, 361.0965, 343.0744, 328.0587, 237.0750, 221.0796, 193.0857, 183.0657, 165.0542	5-Hydroxy-3, 6, 7, 8, 3′, 4′-hexamethoxyflavone	b
**82**	34.80	849.4581	$[M+Na]^+$	-3.6	$C_{43}H_{70}O_{15}$	849.4581, 827.4745, 809.4702, 791.4559, 773.4485, 665.4271, 653.4241, 647.4188, 635.4114, 629.4013, 617.4056, 611.3944, 593.3831, 491.3755, 473.3643, 455.3514, 437.3418, 419.3352, 175.0592, 143.1091, 125.0953	Isoastragaloside II*	a
**83**◆	35.25	351.1929	$[M+K]^+$	-2.6	$C_{18}H_{32}O_4$	351.1929, 335.2248, 313.2357, 295.2311, 277.2141, 259.2087, 241.1952, 227.1655, 209.1555, 191.1440, 173.1319	12,13-Dihydroxy-9,15-octadecadienoic acid	c

Continued on next page

Table S3 (*continued*)

No.	t_R (min)	PI (*m/z*)	Adduct ions	Error (ppm)	Molecular formula	MS/MS fragments (*m/z*)	Tentative identification	Annotation levels
**84**	36.66	849.4581	$[M+Na]^+$	-3.6	$C_{43}H_{70}O_{15}$	849.4581, 827.4745, 809.4702, 791.4559, 773.4485, 665.4271, 653.4331, 647.4188, 635.4114, 629.4102, 617.4056, 611.3944, 593.3831, 491.3755, 473.3643, 455.3514, 437.3418, 419.3352, 175.0592, 143.1091, 125.0953	Cyclocephaloside II*	a
**85**	36.96	869.4866	$[M+H]^+$	-3.8	$C_{45}H_{72}O_{16}$	891.4711, 869.4866, 851.4802, 833.4692, 695.4391, 677.4294, 659.4119, 647.4096, 641.4055, 629.4013, 611.3944, 491.3755, 473.3643, 455.3514, 437.3345, 175.0592, 143.1091, 125.0992	Cycloglobiceposide A	b
**86**	37.08	687.4057	$[M+Na]^+$	-3.9	$C_{37}H_{60}O_{10}$	687.4057, 665.4180, 647.4188, 629.4102, 611.3944, 491.3755, 473.3643, 455.3514, 437.3418, 419.3280, 175.0592, 157.0502, 143.1091, 125.0992	Cyclogaleginoside A	b
**87★**	37.99	363.1193	$[M+Na]^+$	-4.1	$C_{20}H_{20}O_5$	363.1193, 341.1408, 323.1302, 305.1159, 265.0899, 247.0797, 237.0912, 209.0945, 194.0749, 166.0732, 133.0277, 105.0321	Astragquinone	d
**88**	38.05	981.7349	$[2M+H]^+$	-4.7	$C_{30}H_{50}O_5$	513.3610, 491.3755, 473.3643, 455.3589, 437.3418, 419.3352, 143.1091, 125.0953	Cycloastragenol*	a
**89**	38.34	869.4866	$[M+H]^+$	-3.8	$C_{45}H_{72}O_{16}$	869.4866, 851.4802, 833.4692, 707.4365, 689.4189, 671.4149, 653.4241, 653.4061, 635.4114, 617.4056, 599.3914, 491.3755, 473.3643, 455.3514, 437.3418, 419.3280, 217.0734, 157.0502, 143.1091, 125.0992	Astragaloside I*	a
**90**	39.52	869.4866	$[M+H]^+$	-3.8	$C_{45}H_{72}O_{16}$	869.4866, 851.4802, 833.4692, 707.4365, 689.4282, 671.4149, 653.4241, 653.4061, 635.4114, 617.4056, 599.3914, 491.3755, 473.3643, 455.3514, 437.3418, 419.3280, 217.0734, 199.0590, 157.0502, 143.1091, 125.0992	Isoastragaloside I*	a
**91**	40.83	869.4866	$[M+H]^+$	-3.8	$C_{45}H_{72}O_{16}$	869.4866, 851.4802, 833.4590, 707.4365, 689.4282, 671.4149, 653.4241, 653.4061, 635.4025, 617.4056, 599.3914, 491.3755, 473.3643, 455.3514, 437.3418, 419.3280, 217.0734, 157.0502, 143.1091, 125.0992	Neoastragaloside I	b
**92★**	40.93	911.4972	$[M+H]^+$	-3.5	$C_{47}H_{74}O_{17}$	911.4972, 893.4901, 875.4855, 749.4495, 731.4342, 713.4219, 491.3755, 473.3643, 455.3514, 437.3418, 419.3352, 259.0784, 217.0734, 199.0639, 157.0502, 143.1091, 125.0953	Acetylastragaloside I isomer	d
**93◆**	41.37	291.1930	$[M+Na]^+$	-2.1	$C_{16}H_{28}O_3$	307.1605, 291.1930, 269.2090, 251.1964, 233.1905, 215.1743, 205.1989	3-Hydroxy-4,6-hexadecadienoic acid	c
**94◆**	41.78	407.2214	$[M+K]^+$	3.4	$C_{21}H_{36}O_5$	407.2214, 391.2507, 369.2687, 351.2589, 295.2251, 279.2338, 277.2141, 261.2244, 243.2056	13-*Z*, *E*-KODE glycerol ester	b

Continued on next page

Table S3 (*continued*)

No.	t_R (min)	PI (*m/z*)	Adduct ions	Error (ppm)	Molecular formula	MS/MS fragments (*m/z*)	Tentative identification	Annotation levels
**95**◆	41.92	911.4972	$[M+H]^+$	-3.5	$C_{47}H_{74}O_{17}$	911.4972, 893.4901, 875.4750, 749.4495, 731.4342, 689.4189, 671.4149, 653.3971, 515.3726, 497.3623, 455.3514, 437.3418, 419.3280, 217.0682, 199.0590, 157.0458, 143.1049, 125.0953	Trojanoside I	c
**96**◆	42.12	407.2214	$[M+K]^+$	3.4	$C_{21}H_{36}O_5$	407.2214, 391.2507, 369.2620, 351.2523, 295.2311, 277.2200, 259.2031, 241.1952	13-*E*, *E*-KODE glycerol ester	b
**97**◆	42.70	407.2214	$[M+K]^+$	3.4	$C_{21}H_{36}O_5$	407.2214, 391.2437, 369.2620, 351.2523, 295.2251, 277.2141, 259.2087	9-*E*, *E*-KODE glycerol ester	b
**98**	42.77	911.4972	$[M+H]^+$	-3.5	$C_{47}H_{74}O_{17}$	911.4972, 893.4795, 875.4750, 749.4495, 731.4342, 713.4219, 695.4113, 491.3755, 473.3643, 455.3514, 437.3418, 419.3352, 259.0841, 217.0734, 199.0590, 157.0502, 143.1091, 125.0992	Acetylastragaloside I	b
**99**★	42.99	911.4972	$[M+H]^+$	-3.5	$C_{47}H_{74}O_{17}$	911.4972, 893.4901, 875.4855, 749.4495, 731.4342, 689.4189, 671.4149, 653.3971, 515.3726, 497.3623, 455.3439, 437.3345, 419.3352, 217.0682, 199.0590, 157.0502, 143.1049	Trojanoside I isomer	d
**100**◆	43.72	317.2101	$[M+Na]^+$	2.5	$C_{18}H_{30}O_3$	333.1786, 317.2101, 295.2251, 277.2200, 259.2031, 241.1952, 179.1437	13-*Z*, *E*-oxooctadeca-9,11-dienoic acid	b
**101**◆	43.97	317.2101	$[M+Na]^+$	2.5	$C_{18}H_{30}O_3$	333.1786, 317.2101, 295.2311, 277.2141, 259.2087, 241.1952, 179.1437	13-*E*, *E*-oxooctadeca-9,11-dienoic acid	b
**102**◆	44.42	317.2101	$[M+Na]^+$	2.5	$C_{18}H_{30}O_3$	333.1850, 317.2101, 295.2251, 277.2141, 259.2087, 241.1952, 171.1001	9-*E*, *E*-oxooctadeca-10,12-dienoic acid	b

Note: Level a: the compound was identified using at least two independent parameters that were compared with an authentic compound. Level b: the compound was identified provisionally through spectral comparison with public or commercial spectral libraries. Level c: the compound was assigned to a compound class based on spectral similarity to a known compound of a chemical class. Level d: unknown compound quantified from spectral data without further identification and classification (Chen et al., 2025).

★ potentially new compounds tentatively identified according to the search results in SciFinder database.

◆ Known compounds identified in AR for the first time.

* Confirmed with corresponding reference substances.

Table S4 Inhibition rates of TNF-α and IL-6 by HLQE, isolated compounds, and dexamethasone in LPS-stimulated RAW 264.7 macrophages.

Group	Concentration	Cell Viability (%)	TNF-*α* Inhibition (%)	IL-6 Inhibition (%)
LPS	1 µg/mL	102.2 ± 6.6	-	-
HLQE	10 µg/mL	108.8 ± 2.1	47.6 ± 6.0**	30.1 ± 9.3
Compound 1	10 µM	108.8 ± 5.8	37.6 ± 9.7*	29.3 ± 6.2
Compound 2	100 µM	118.8 ± 3.0	56.0 ± 10.2**	39.2 ± 7.3*
Compound 3	50 µM	110.2 ± 4.5	43.1 ± 9.3*	33.3 ± 5.8
Compound 4	50 µM	112.1 ±6.4	51.6 ± 14.9*	37.3 ± 12.8
DEX	10 µM	125.0 ± 4.0	83.8 ± 6.2***	46.1 ± 11.4*

Note: Data are presented as mean ± SEM (n ⩾ 4). Statistical significance relative to the LPS-stimulated model group is indicated as *$P < 0.05$, **$P < 0.01$, and ***$P < 0.001$. No significant cytotoxicity was observed at the indicated concentrations. LPS, lipopolysaccharide; HLQE, ethyl acetate fraction; DEX, dexamethasone; TNF-*α*, tumor necrosis factor-alpha; IL-6, interleukin-6. Compound 1: astragalinin A; Compound 2: astragalinin B; Compound 3: astragquinone; Compound 4: 2-(hydroxymethyl)anthraquinone, internal reference.

Table S5 Binding energy between key targets (TNF-α and IL-6) and key anti-inflammatory compounds in HLQE.

Docking run	Binding energy (kcal mol^{-1})							
	Compound 4		Compound 1		Compound 2		Compound 3	
	2AZ5	1ALU	2AZ5	1ALU	2AZ5	1ALU	2AZ5	1ALU
1	-7.7	-6.5	-6.9	-6.0	-7.2	-5.6	-9.1	-7.5
2	-7.6	-6.4	-6.8	-5.9	-7.1	-5.6	-8.4	-7.4
3	-7.5	-6.1	-6.7	-5.7	-7.0	-5.4	-8.4	-7.1
4	-7.5	-6.1	-6.6	-5.6	-6.9	-5.4	-8.3	-7.0
5	-7.4	-6.0	-6.5	-5.5	-6.8	-5.4	-8.1	-7.0
6	-7.4	-5.8	-6.4	-5.5	-6.7	-5.3	-8.0	-6.9
7	-7.4	-5.7	-6.4	-5.4	-6.6	-5.4	-8.0	-6.9
8	-7.3	-5.7	-6.3	-5.4	-6.6	-5.4	-8.0	-6.9
9	-7.1	-5.6	-6.2	-5.3	-6.6	-5.3	-8.0	-6.8

Note: Compound 1: astragalinin A (peak **47**); Compound 2: astragalinin B (peak **54**); Compound 3: astragaquinone (peak **87**); Compound 4: 2-(hydroxymethyl)anthraquinone (peak **44**, reference compound).

Fig. S1 Cytotoxicity screening and anti-inflammatory evaluation of HLQ fractions at 10 μg/mL in RAW 264.7 macrophages. (A) Cell viability of RAW 264.7 macrophages treated with HLQE, HLQP, and HLQW at a series of concentrations assessed by CCK-8 assay. (B) Cell viability, (C) TNF-α levels, and (D) IL-6 levels in LPS-stimulated RAW 264.7 macrophages treated with HLQE, HLQP, and HLQW at 10 μg/mL. C: normal control group; M: LPS-stimulated model group. Data are presented as mean ± SD ($n = 6$). Statistical significance: #$p < 0.05$, ##$p < 0.01$ vs. normal control group; *$p < 0.05$, **$p < 0.01$ vs. LPS-stimulated model group.

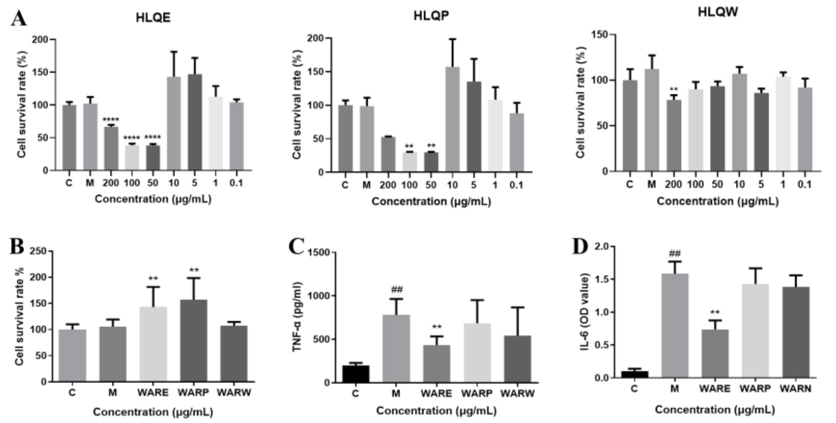

Fig. S2 Base peak ion (BPI) chromatogram of HLQE in positive ion mode.

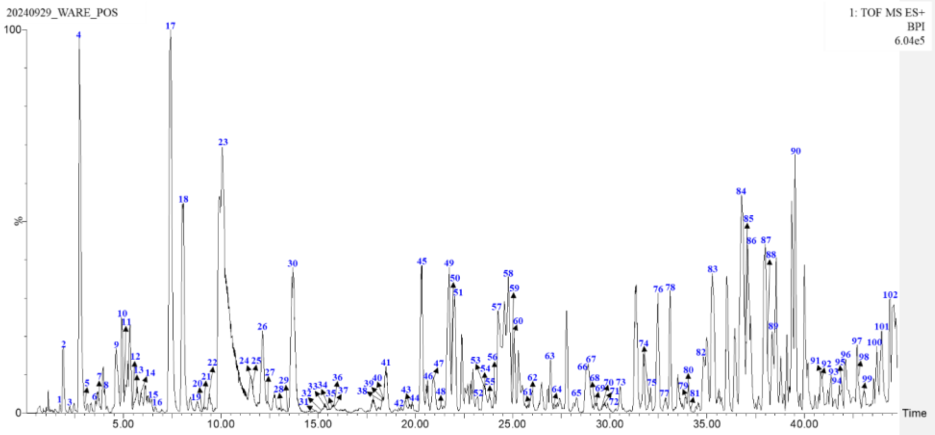

Fig. S3 Effects of compounds 1–4 on RAW 264.7 cell viability assessed by CCK-8 assay. C: normal control group; V: vehicle control group (DMSO); NA: data invalid at this concentration due to solvent-induced cytotoxicity of the vehicle control. Statistical significance: *p < 0.05, **p < 0.01 vs. vehicle control group; ##p < 0.01 vs. normal control group. Data are presented as mean ± SEM (n = 6). Compound 1: astragalinin A (peak **47**); Compound 2: astragalinin B (peak **54**); Compound 3: astragaquinone (peak **87**); Compound 4: 2-(hydroxymethyl)anthraquinone (peak **44**, reference compound).

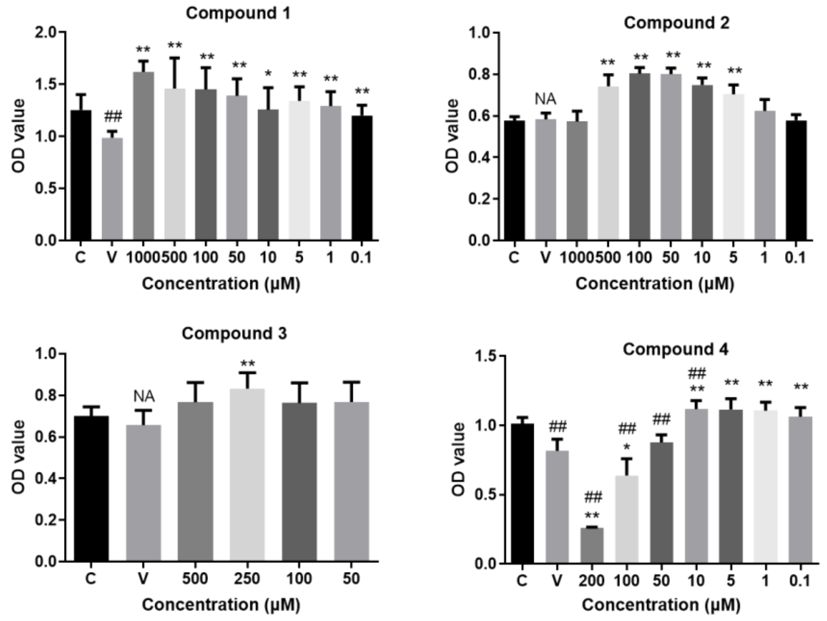

Fig. S5 A: Isoflavones tentatively assigned in HLQE samples. B: Proposed MS^2 fragmentation pathways of representative compound (calycosin-7-*O*-*β*-D-glucoside).

A **Isoflavones (cluster II, VII, and IX)**

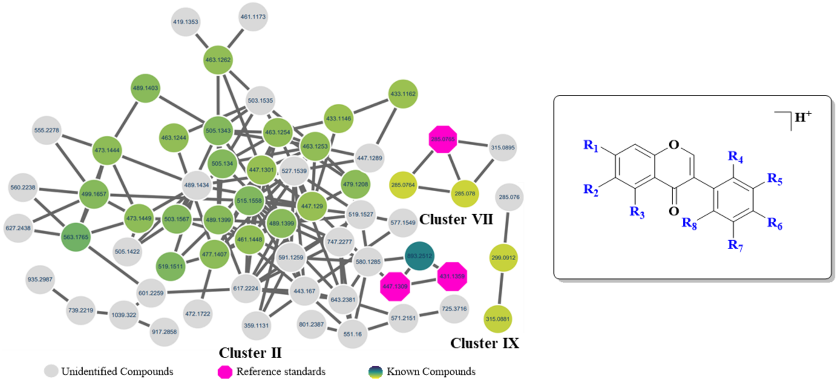

No.	Compounds				*m/z*
**1**	Isoflavone-3-(4′-glucopyranosyl-5′-hydroxy-2′-methoxyphenyl)-7-hydroxy-4*H*-chromen-4-one	R_1=R_5=OH	R_6=Glc	R_8=OCH_3	463.1204
**3**	3′-Hydroxy-formononetin-7-*O*-*β*-D-glucoside	R_1=Glc	R_5=OCH_3	R_7=OH	447.1270
**4**	Calycosin 7-*O*-*β*-D-glucoside	R_1=Glc	R_5=OH	R_6=OCH_3	447.1270
**5**	Odoratin 7-*O*-*β*-D-glucoside	R_1=Glc	R_2=R_6=OCH_3	R_5=OH	477.1395
**6**	Genistin	R_1=Glc	R_3=R_6=OH		433.1123
**8**	Isoflavone-*O*-glucoside	R_1=Glc	R_5=R_6=OH	R_8=OCH_3	463.1280
**9**	Sophoricoside	R_1=R_3=OH	R_6=Glc		433.1123
**10**	Pratensein 7-*O*-*β*-D-glucopyranoside	R_1=Glc	R_3=R_5=OH	R_6=OCH_3	463.1280
**13**	Calycosin 7-*O*-glc-4″-*O*-ace	R_1=4″-*O*-Ac-Glc	R_5=OH	R_6=OCH_3	489.1387
**14**	Isoflavone-*O*-glycoside	R_1=Glc	R_3=R_5=R_7=OH	R_6=OCH_3	479.1175
**15**	Formononetin-glc-api	R_1=Api-(1→2)-Glc	R_6=OCH_3		563.1771
**16**	Calycosin 7-*O*-glc-2″-*O*-ace isomer	R_1=2″-*O*-Ac-Glc	R_5=OH	R_6=OCH_3	489.1387
**17**	Ononin	R_1=Glc	R_5=OCH_3		431.1368
**18**	Calycosin 7-*O*-glc-6″-*O*-ace	R_1=6″-*O*-Ac-Glc	R_5=OH	R_6=OCH_3	489.1387
**20**	Odoratin 7-*O*-glc-ace	R_1=6″-*O*-Ac-Glc	R_2=R_6=OCH_3	R_5=OH	519.1510
**24**	Calycosin	R_1=OH	R_5=OH	R_6=OCH_3	285.0750
**25**	5-Hydroxy-3-[4-hydroxy-2-[(2*S*,3*R*,4*S*,5*S*,6*R*)-3,4,5-trihydroxy-6-(hydroxymethyl)oxan-2-yl]oxyphenyl]-7-methoxychromen-4-one	R_1=OCH_3	R_3=R_6=OH	R_4=Glc	463.1204
**28**	4''-*O*-acetyl-pratensein-7-*O*-*β*-D-glucoside isomer	R_1=4″-*O*-Ac-Glc	R_3=R_5=OH	R_6=OCH_3	505.1322
**30**	Odoratin	R_1=R_5=OH	R_2=R_6=OCH_3		315.0885
**31**	Formononetin 7-*O*-glc-4″-*O*-ace	R_1=4″-*O*-Ac-Glc	R_6=OCH_3		473.1422
**33**	2''-Acetyl-ononin isomer	R_1=2″-*O*-Ac-Glc	R_6=OCH_3		473.1422
**35**	Sissotrin	R_1=Glc	R_3=OH	R_6=OCH_3	447.1270
**37**	6″-*O*-acetyl-pratensein-7-*O*-*β*-D-glucoside	R_1=6″-*O*-Ac-Glc	R_3=R_5=OH	R_6=OCH_3	505.1322
**39**	Calycosin 7-*O*-*β*-D-{6''-(*E*)-but-2-enoyl]} -glucopyranoside	R_1=6″-(*E*)-but-2-enoyl-Glc	R_5=OH	R_6=OCH_3	515.1569
**45**	Formononetin 7-*O*-glc-6″-*O*-ace	R_1=6″-*O*-Ac-Glc	R_6=OCH_3		473.1422
**48**	6″-*O*-Acetylwistin	R_1=6″-*O*-Ac-Glc	R_2=R_6=OCH_3		503.1564
**59**	Aformosin	R_1=OH	R_2=R_6=OCH_3		299.0900
**62**	Ammopiptanoside A	R_1=6″-(*E*)-but-2-enoyl-Glc		R_6=OCH_3	499.1615

B

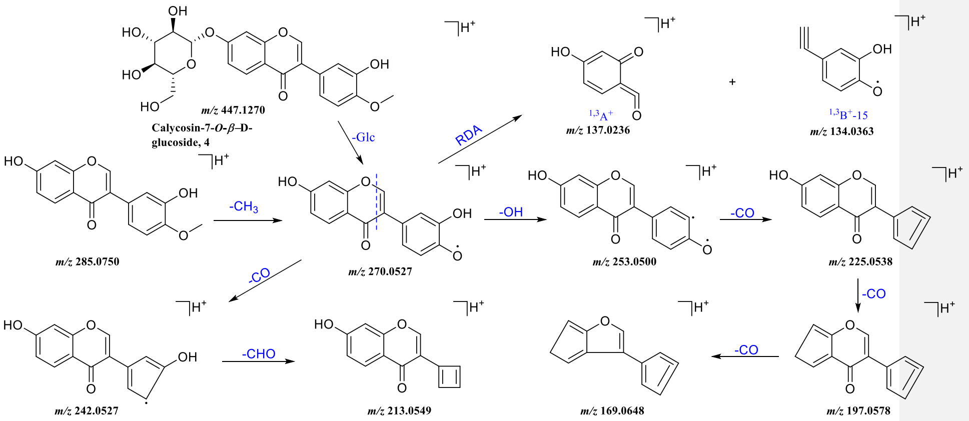

Fig. S6 A: 4-methoxyisoflavans and pterocarpenoids tentatively assigned in HLQE samples. B: Proposed MS^2 fragmentation pathways of representative compound (astragalinin A).

A **4-methoxyisoflavans and pterocarpenoids (cluster I)**

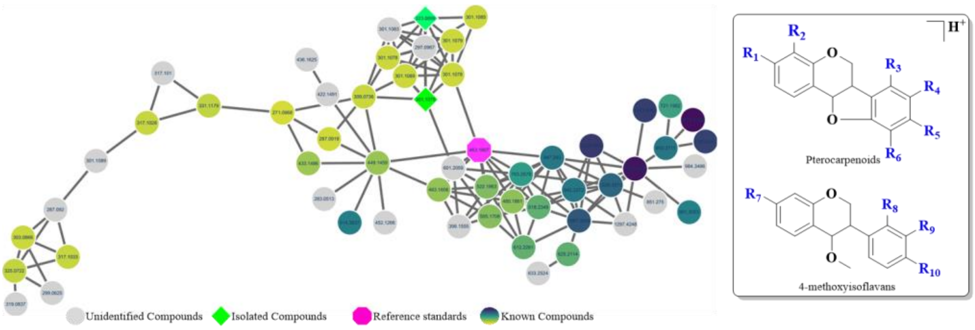

No.	Compounds				m/z
11	Licoagroside D	R_1=Glc	R_5=OCH$_3$	R_6=OH	449.1462
12	Pterocarpane-*O*-glycoside	R_1=Glc	R_4=R_6=OH	R_5=OCH$_3$	465.1374
19	Astrapterocarpanoside A	R_1=Api-(1→2)-Glc	R_5=R_6=OCH$_3$		595.2021
22	Pterocarpans	R_1=R_4=R_6=OH	R_5=OCH$_3$		303.0843
23	(-)-Methylnissolin-3-*O*-glucoside	R_1=Glc	R_5=R_6=OCH$_3$		463.1583
29	Medicarpin-3-*O*-*β*-D-glucopyranoside	R_1=Glc	R_5=OCH$_3$		433.1489
32	Vesticarpan	R_1=R_6=OH	R_5=OCH$_3$		287.0932
38	(-)-Methylinissolin 3-*O*-*β*-D-(6'-acetyl)-glucopyranoside isomer	R_1=2'-*O*-Ac-GlcGlc	R_5=R_6=OCH$_3$		505.1718
41	(6a*R*, 11a*R*)-3,8-dihydroxy-9,10-dimethoxypterocarpan	R_1=R_4=OH	R_5=R_6=OCH$_3$		317.1035
46	3,10-Dihydroxy-7,9-dimethoxypterocarpan	R_1=R_6=OH	R_3=R_5=OCH$_3$		317.1035
47	Astragalinin A	R_7=R_8=OH	R_9=R_{10}=OCH$_3$		333.1336
49	(-)-Methylinissolin 3-*O*-*β*-D-(6'-acetyl)-glucopyranoside	R_1=6'-*O*-Ac-Glc	R_5=R_6=OCH$_3$		505.1718
55	Micanspterocarpan	R_1=OH	R_2=R_3=R_4=OCH$_3$		331.1195
57	(-)-Methylnissolin	R_1=OH	R_5=R_6=OCH$_3$		301.1082
70	Astragalinin isomer	R_7=R_8=OH	R_9=R_{10}=OCH$_3$		333.1336

B

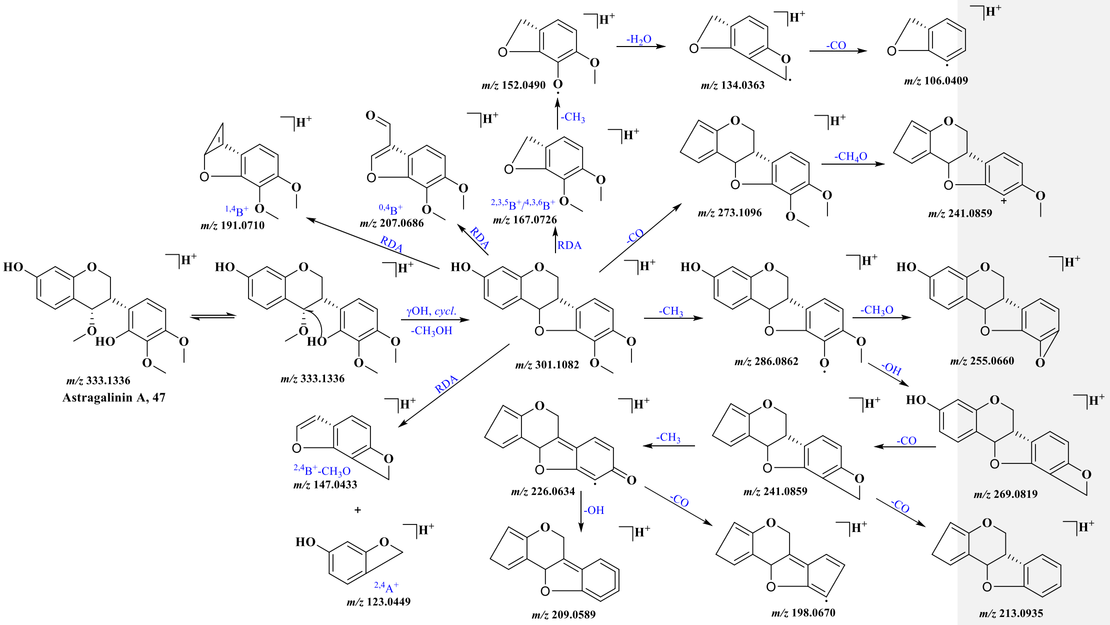

Fig. S7 A: Saponins tentatively assigned in HLQE samples. B: Proposed MS^2 fragmentation pathways of representative compound (isoastragaloside I).

A **Saponins (cluster III)**

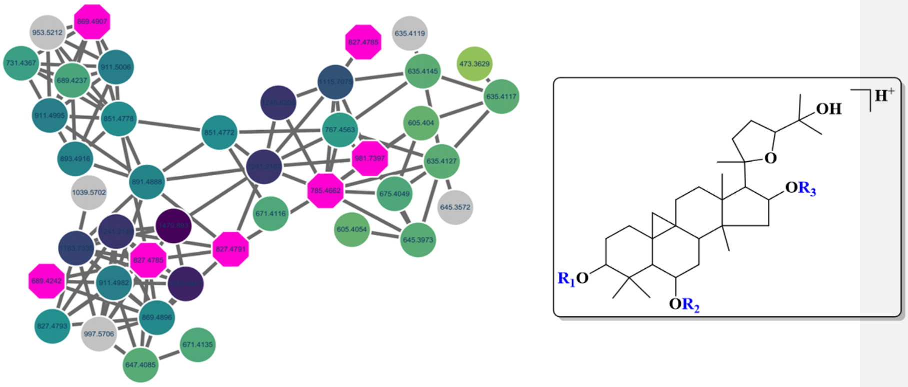

Unidentified Compounds Reference standards Known Compounds

No.	Compounds				*m/z*
65	Astralanosaponin H	R_1=2-*O*-Ac-Xyl	R_2=Glc	R_3=H	827.4745
67	Astragaloside IV	R_1=Xyl	R_2=Glc	R_3=H	785.4666
74	Cycloaraloside A	R_1=Glc	R_2=R_3=H		653.4241
75	Astramembrannin II	R_1=Xyl	R_2=R_3=H		623.4110
76	Astragaloside II	R_1=2-*O*-Ac-Xyl	R_2=Glc	R_3=H	827.4745
82	Isoastragaloside II	R_1=3-*O*-Ac-Xyl	R_2=Glc	R_3=H	827.4745
84	Cyclocephaloside II	R_1=4-*O*-Ac-Xyl	R_2=Glc	R_3=H	827.4745
85	Cycloglobiceposide A	R_1=2-*O*-Ac-Xyl	R_2=6'-*O*-Ac-Glc	R_3=H	869.4866
86	Cyclogaleginoside A	R_1=2-*O*-Ac-Xyl	R_2=R_3=H		665.4180
88	Cycloastragenol	R_1=R_2=R_3=H			491.3755
89	Astragaloside I	R_1=2,3-*O*-Ac_2-Xyl	R_2=Glc	R_3=H	869.4866
90	Isoastragaloside I	R_1=2,4-*O*-Ac_2-Xyl	R_2=Glc	R_3=H	869.4866
91	Neoastragaloside I	R_1=3,4-*O*-Ac_2-Xyl	R_2=Glc	R_3=H	869.4866
92	Acetylastragaloside I isomer	R_1=2,3,4-*O*-Ac_3-Xyl	R_2=Glc	R_3=H	911.4972
95	Trojanoside I	R_1=2,3-*O*-Ac_2-Xyl	R_2=Glc	R_3=Ac	911.4972
98	Acetylastragaloside I	R_1=2,3,4-*O*-Ac_3-Xyl	R_2=Glc	R_3=H	911.4972

B

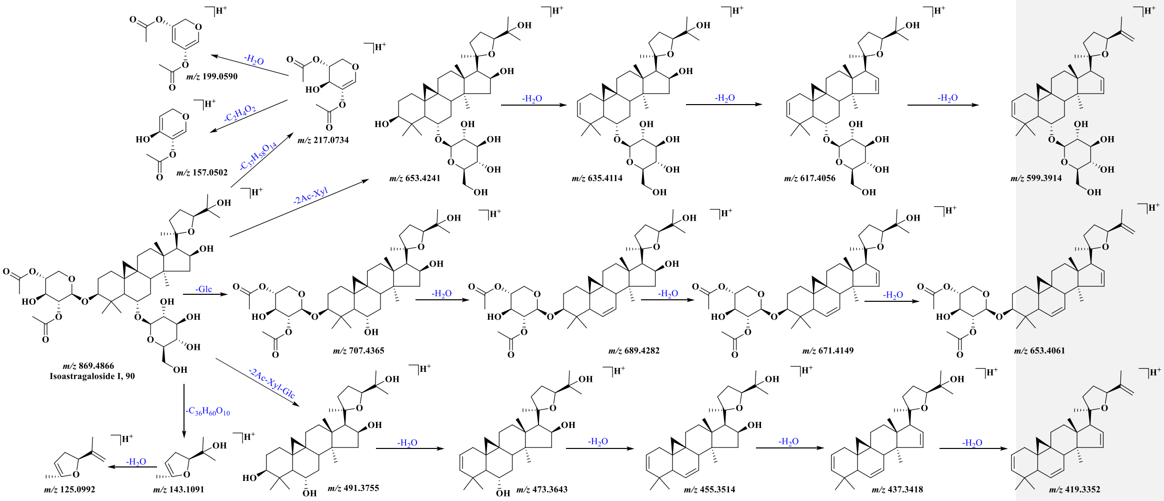

Fig. S8 A: Isoflavans tentatively assigned in HLQE samples. B: Proposed MS^2 fragmentation pathways of representative compounds (isomucronulatol-7-*O*-*β*-D-glucopyranoside and isomucronulatol).

A

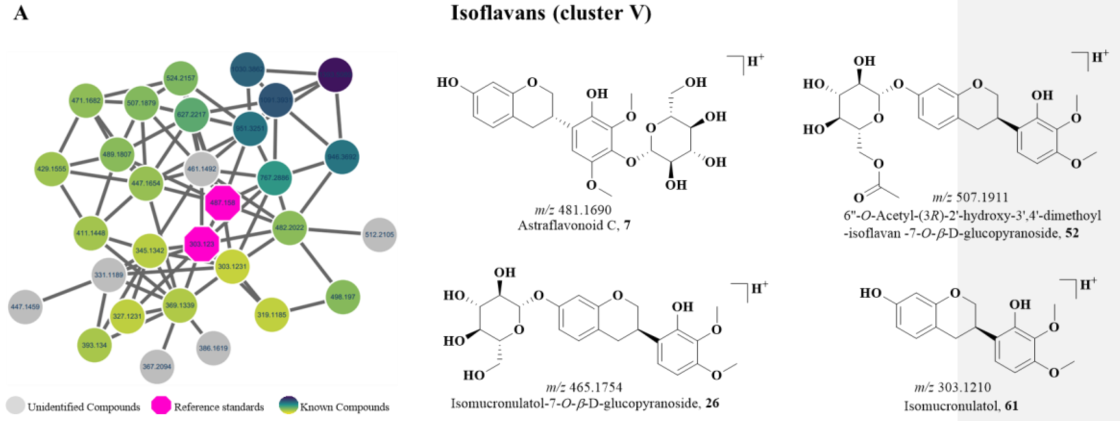

B

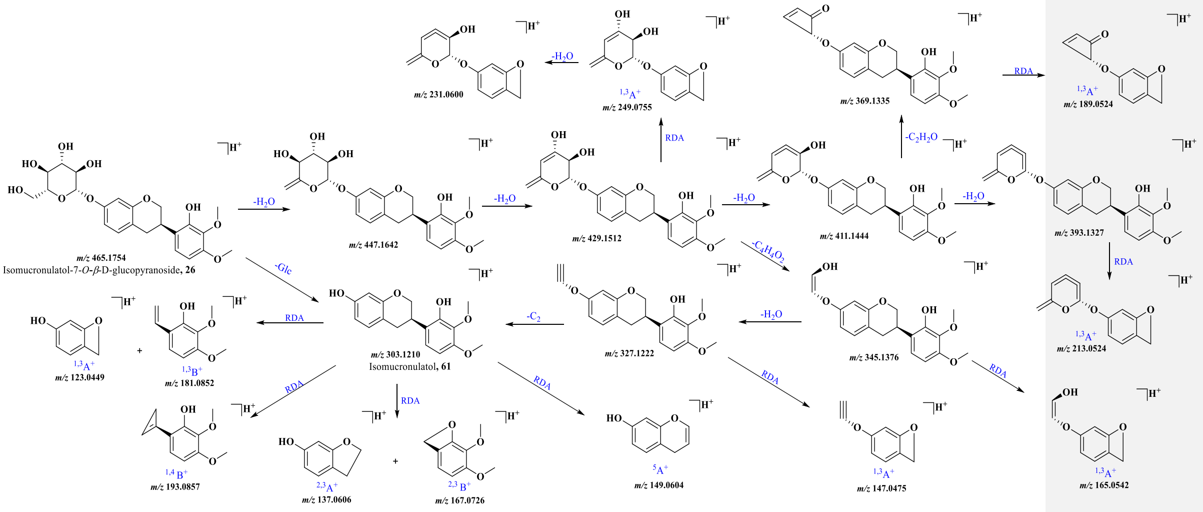

Fig. S9 A: Polymethoxyflavones tentatively assigned in HLQE samples. B: Proposed MS^2 fragmentation pathways of representative compound (nobiletin).

A

Polymethoxyflavones (cluster VI)

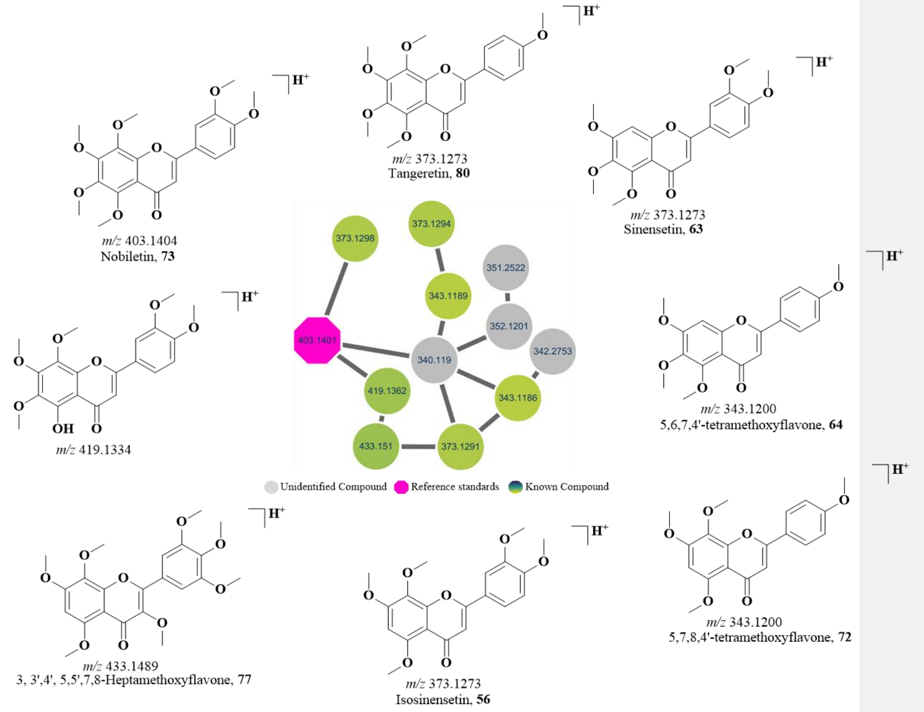

B

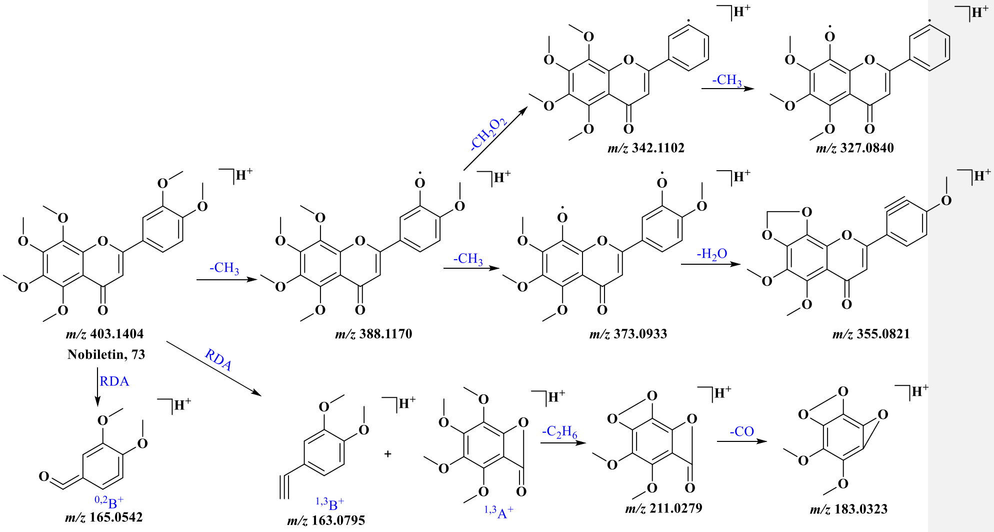

Fig. S10 A: Anthraquinones tentatively assigned in HLQE samples. B: Proposed MS^2 fragmentation pathways of representative compound (astragquinone).

A **Anthraquinones (cluster VIII)**

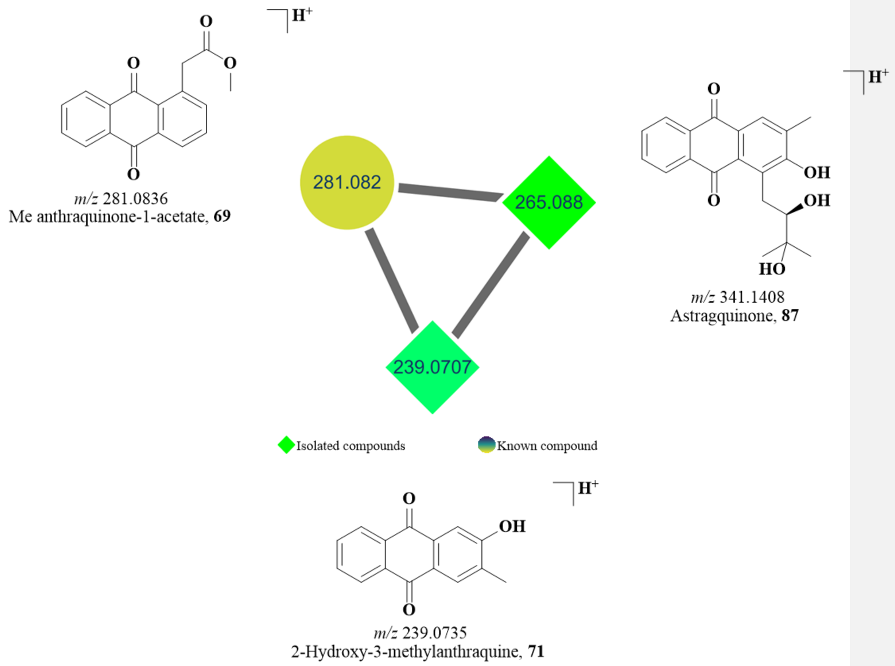

B

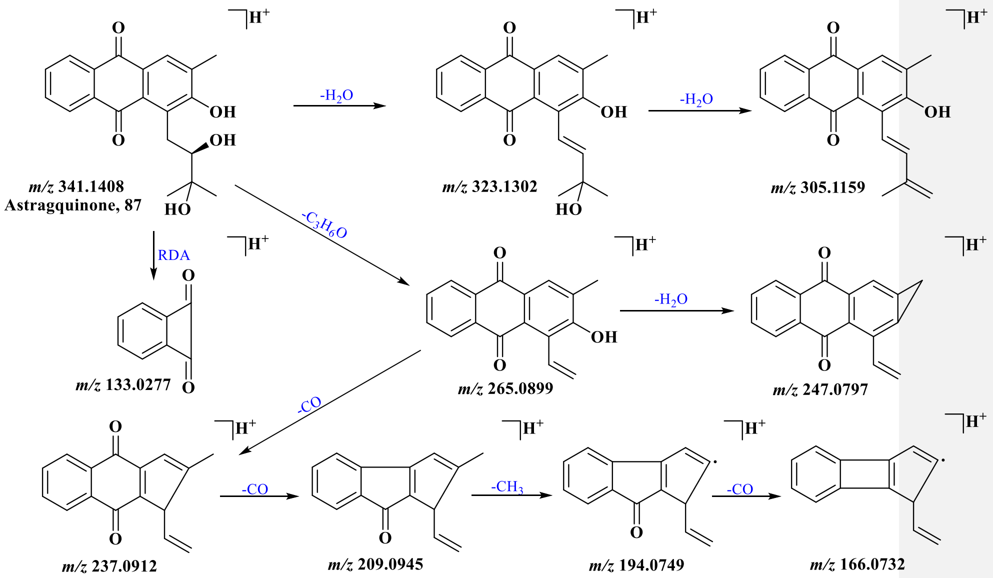

Fig. S11 A: Fatty acids tentatively assigned in HLQE samples. B: Proposed MS^2 fragmentation pathways of representative compound (13-*E*, *E*-oxooctadeca-9,11-dienoic acid).

A

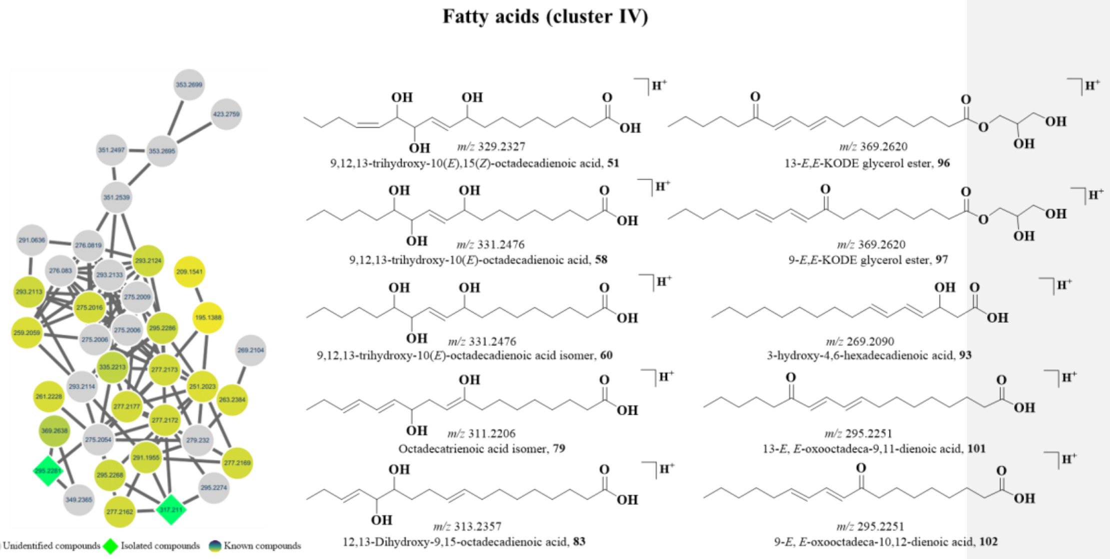

B

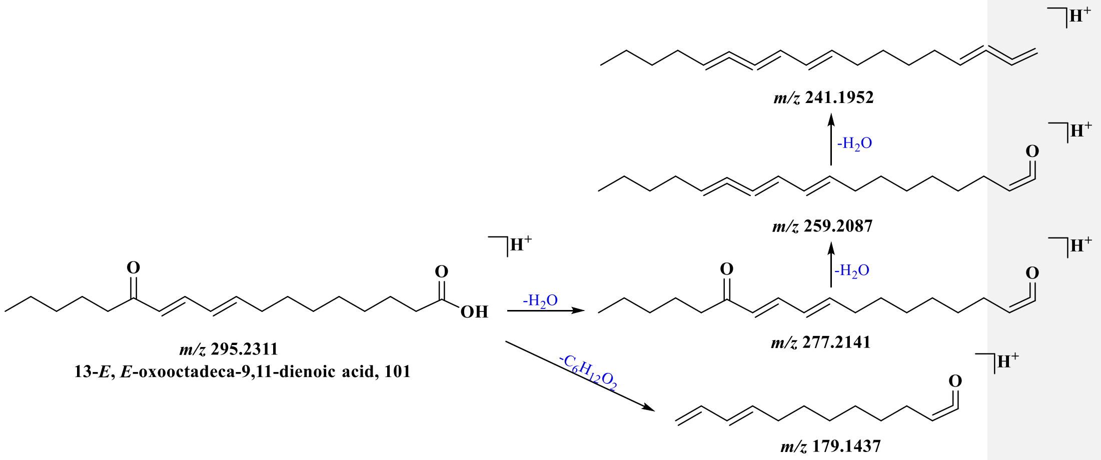

Fig. S12 ^{1}H NMR spectrum (500 MHz) of astragalinin A (peak **47**) in CD_3OD.

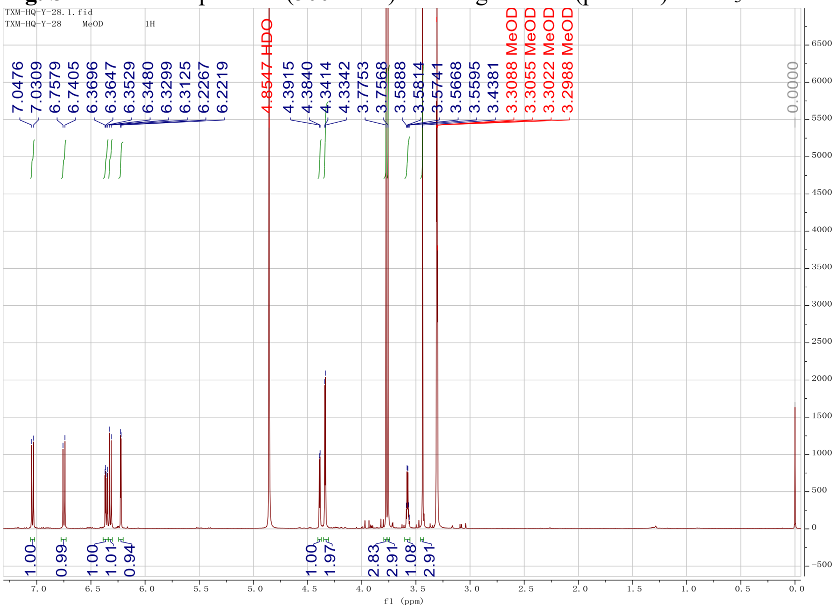

Fig. S13 ^{13}C NMR spectrum (125 MHz) of astragalinin A (peak **47**) in CD_3OD.

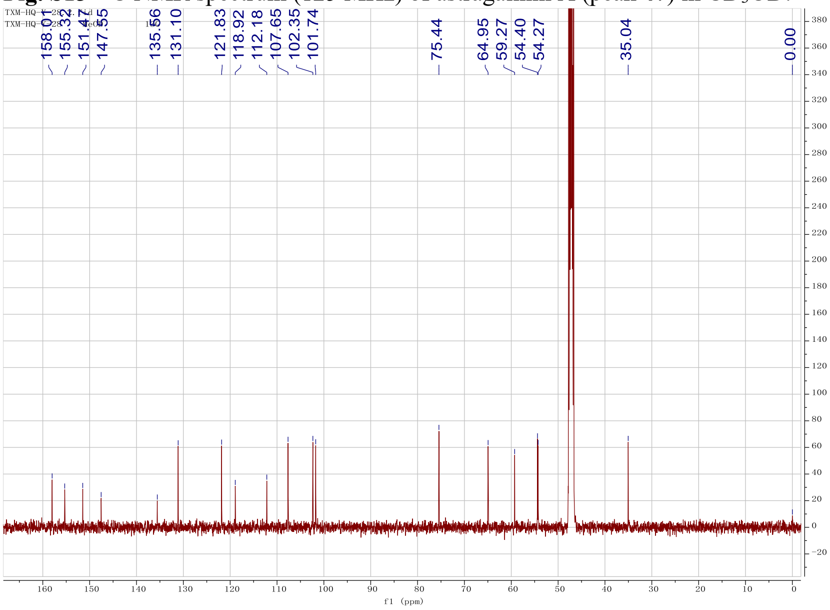

Fig. S14 ^{1}H-^{1}H COSY spectrum (500 MHz) of astragalinin A (peak **47**) in CD_3OD.

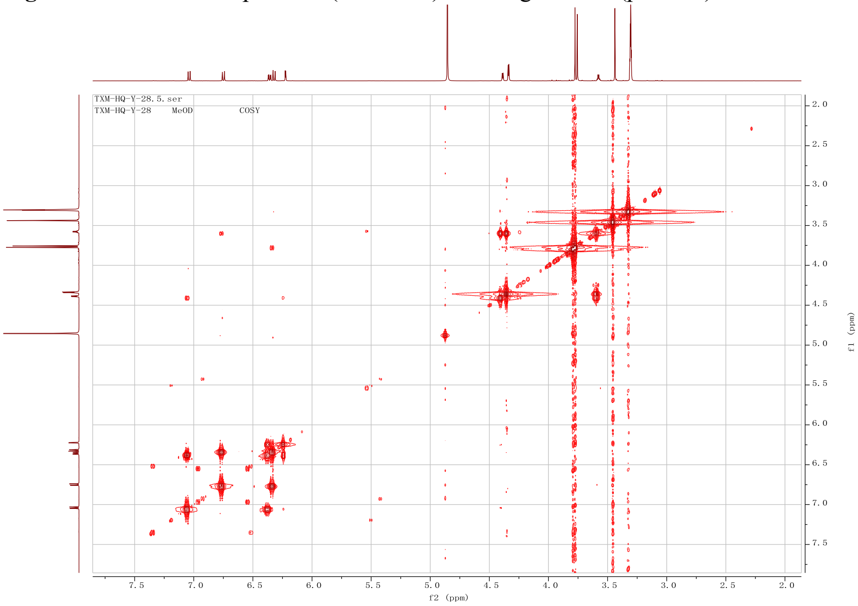

Fig. S15 HSQC spectrum (500 MHz) of astragalinin A (peak **47**) in CD_3OD.

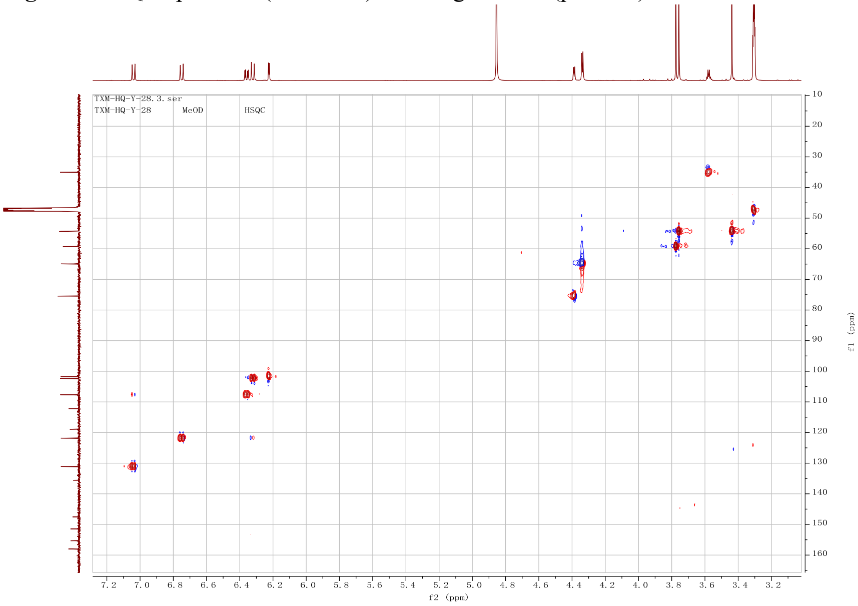

Fig. S16 HMBC spectrum (500 MHz) of astragalinin A (peak **47**) in CD_3OD.

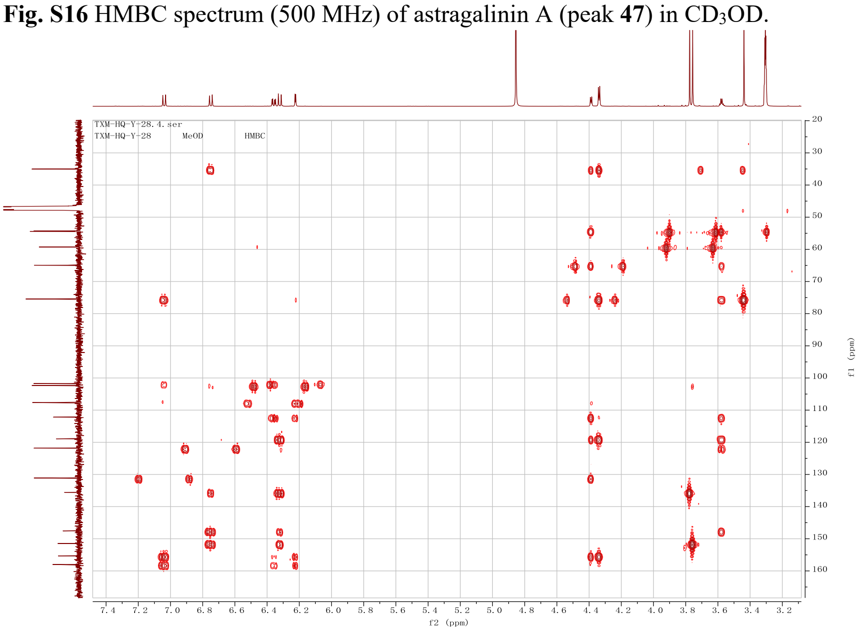

Fig. S17 ROESY spectrum (500 MHz) of astragalinin A (peak **47**) in CD_3OD.

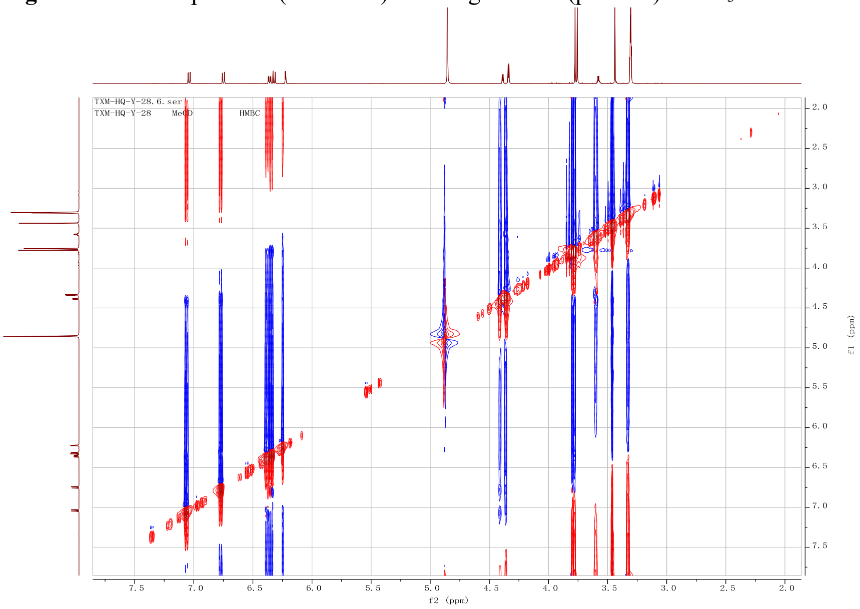

Fig. S18 UV spectrum of astragalinin A (peak **47**).

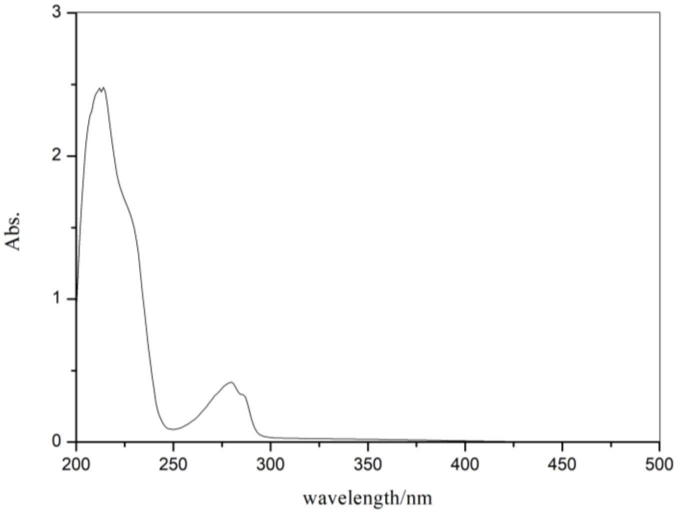

Fig. S19 IR spectrum of astragalinin A (peak **47**).

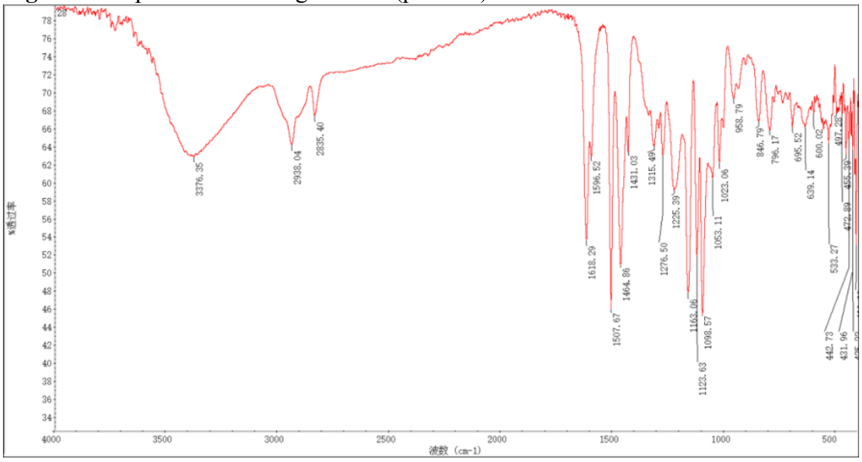

Fig. S20 CD spectrum of astragalinin A (peak **47**).

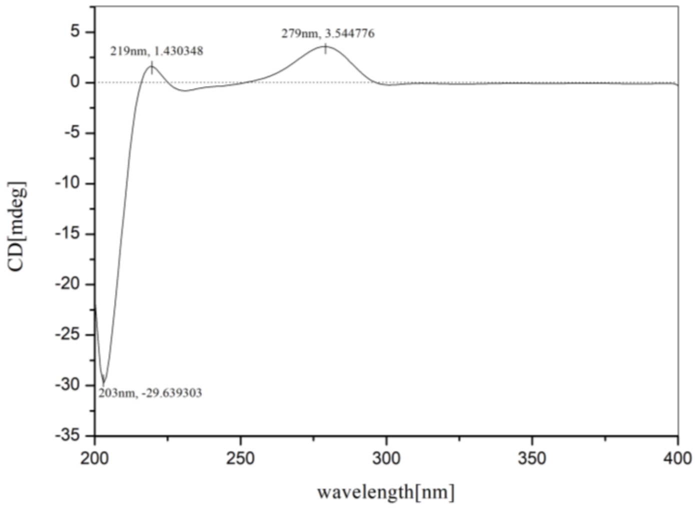

Fig. S21 ECD spectrum of astragalinin A (peak **47**).

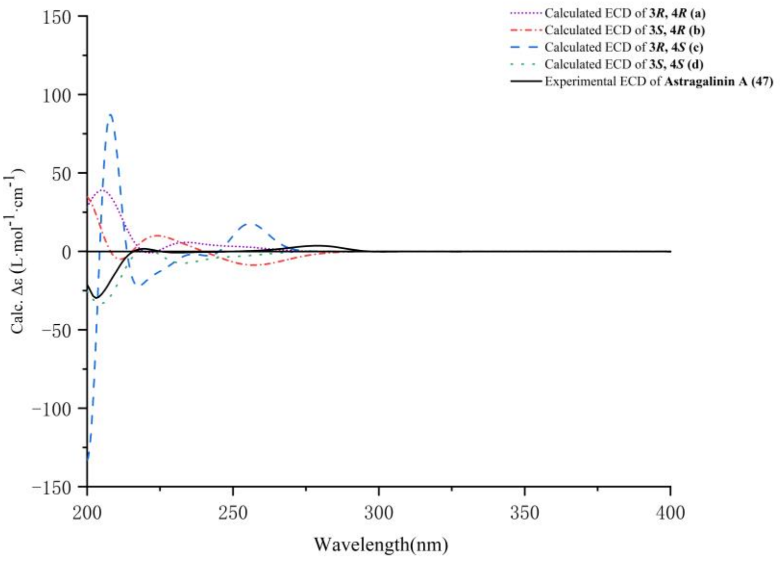

Fig. S22 ^{1}H NMR spectrum (500 MHz) of astragalinin B (peak **54**) in CD_3OD.

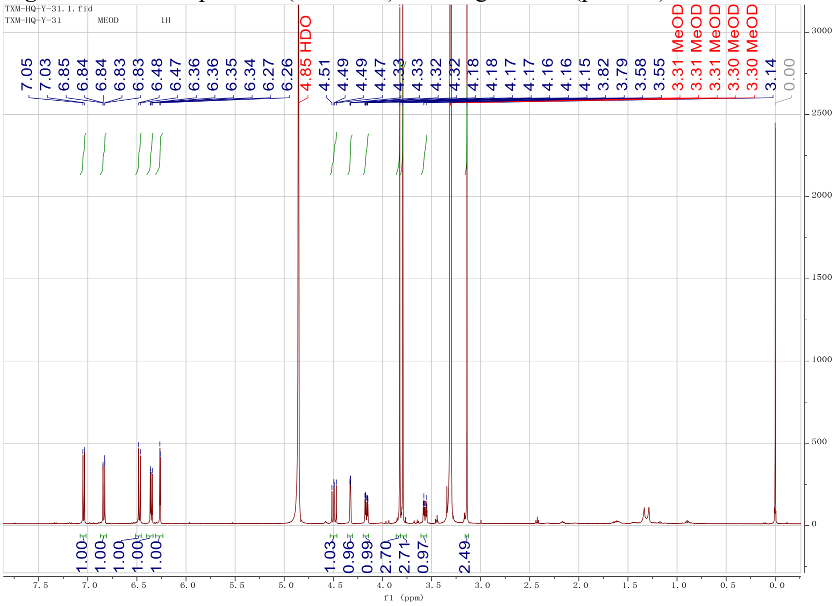

Fig. S23 ^{13}C NMR spectrum (125 MHz) of astragalinin B (peak **54**) in CD_3OD.

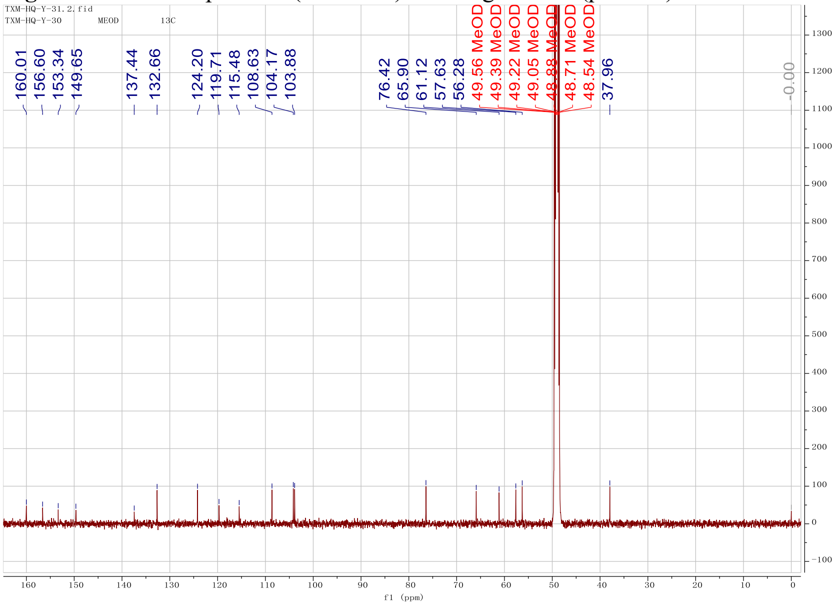

Fig. S24 ^{1}H-^{1}H COSY spectrum (500 MHz) of astragalinin B (peak **54**) in CD_3OD.

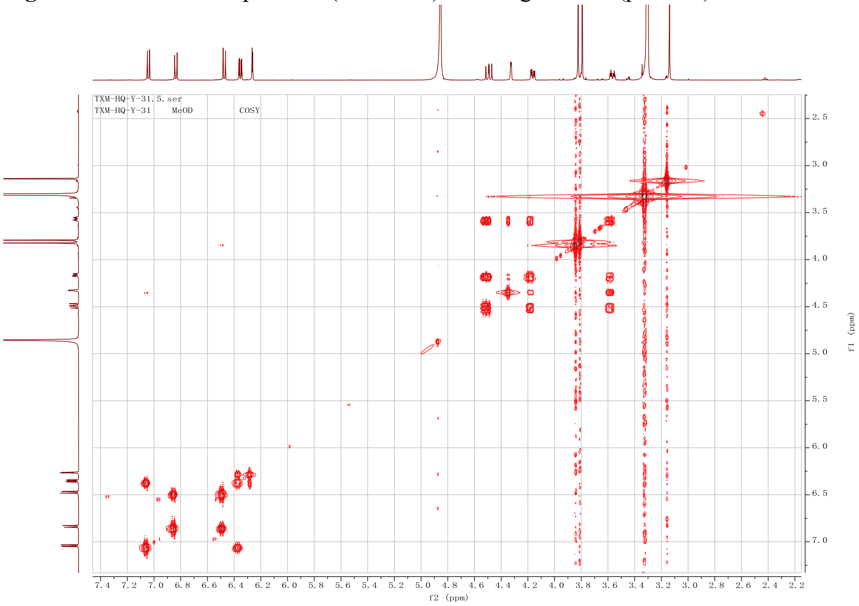

Fig. S25 HSQC spectrum (500 MHz) of astragalinin B (peak **54**) in CD_3OD.

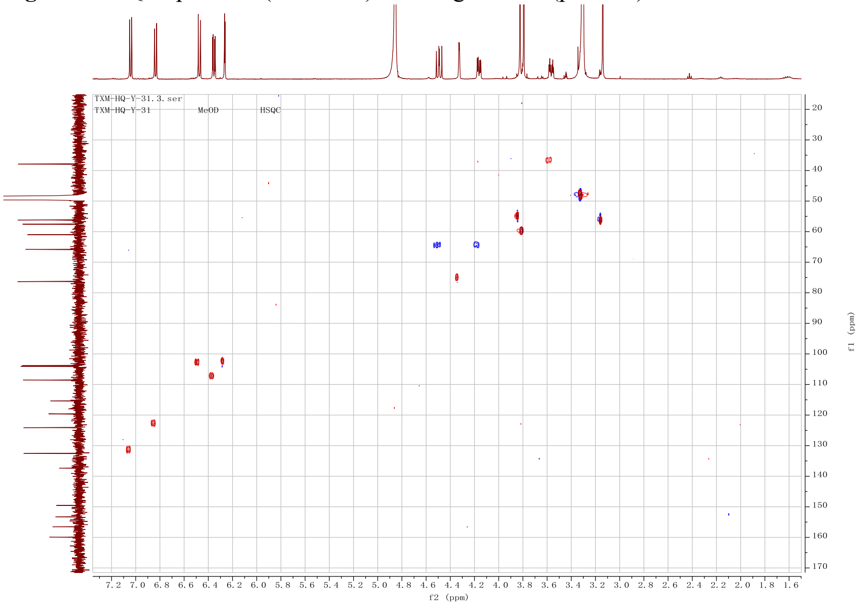

Fig. S26 HMBC spectrum (500 MHz) of astragalinin B (peak **54**) in CD_3OD.

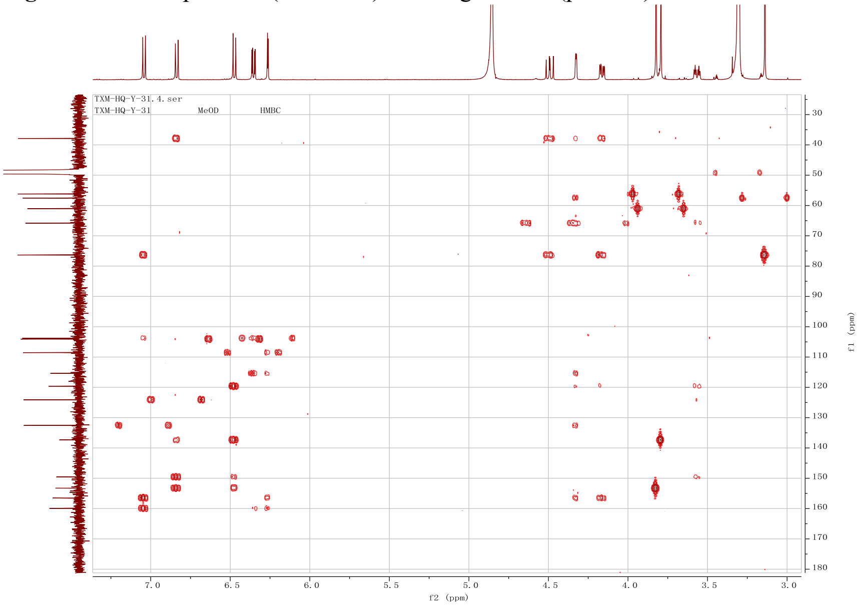

Fig. S27 ROESY spectrum (500 MHz) of astragalinin B (peak **54**) in CD_3OD.

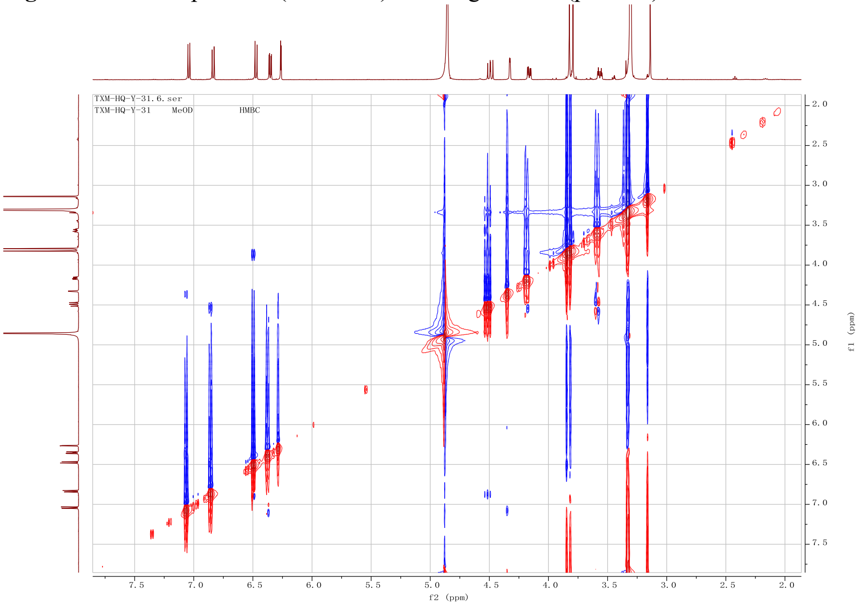

Fig. S28 UV spectrum of astragalinin B (peak **54**).

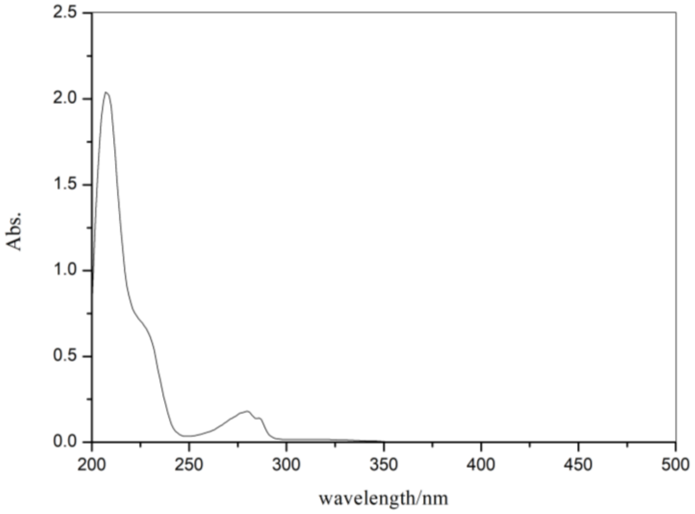

Fig. S29 IR spectrum of astragalinin B (peak **54**).

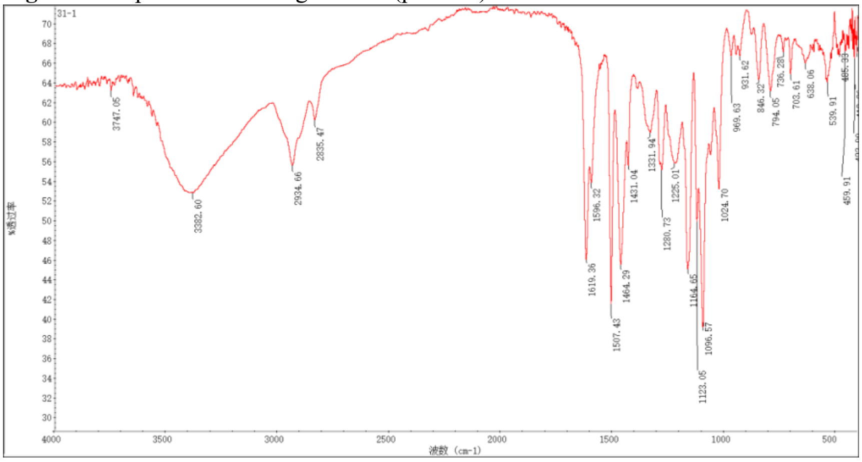

Fig. S30 CD spectrum of astragalinin B (peak **54**).

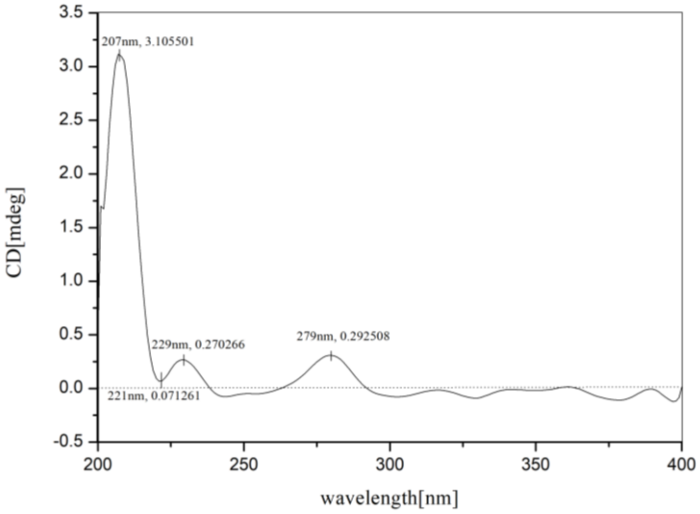

Fig. S31 ECD spectrum of astragalinin B (peak **54**).

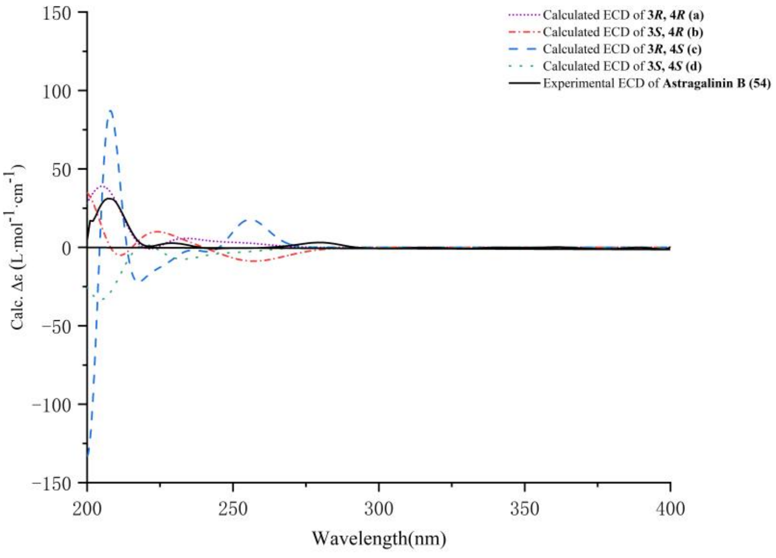

Fig. S32 ^{1}H NMR spectrum (500 MHz) of astragquinone (peak **87**) in CD_3OD.

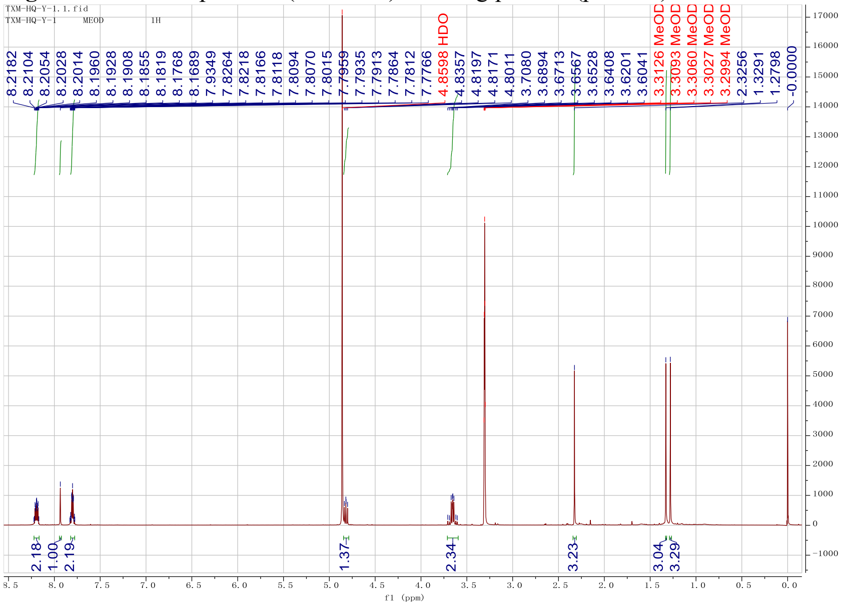

Fig. S33 ^{13}C NMR spectrum (125 MHz) of astragquinone (peak **87**) in CD_3OD.

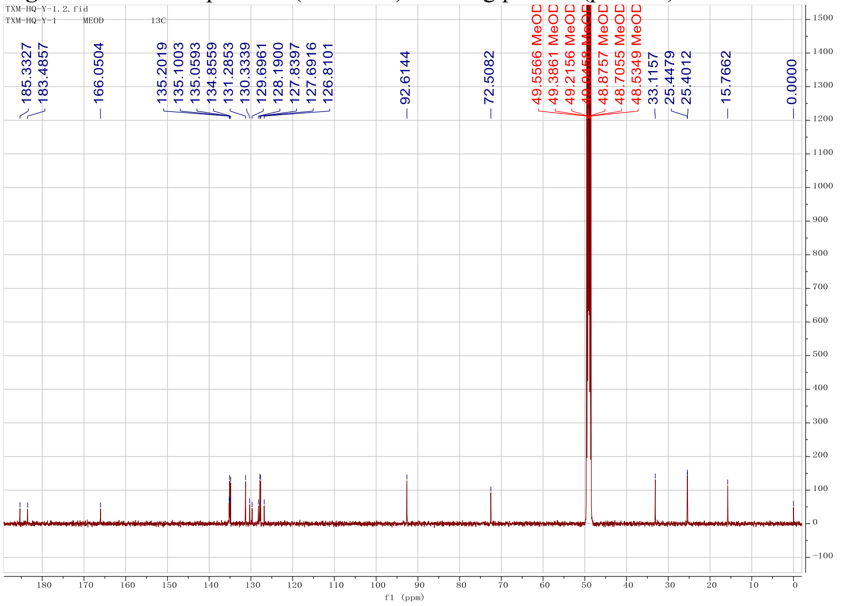

Fig. S34 ^{1}H-^{1}H COSY spectrum (500 MHz) of astragquinone (peak **87**) in CD$_3$OD.

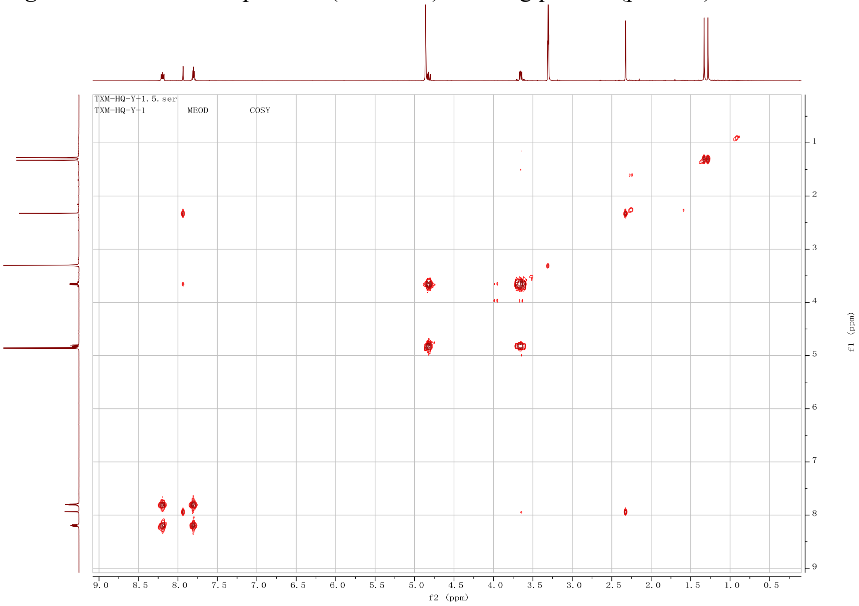

Fig. S35 HSQC spectrum (500 MHz) of astragquinone (peak **87**) in CD$_3$OD.

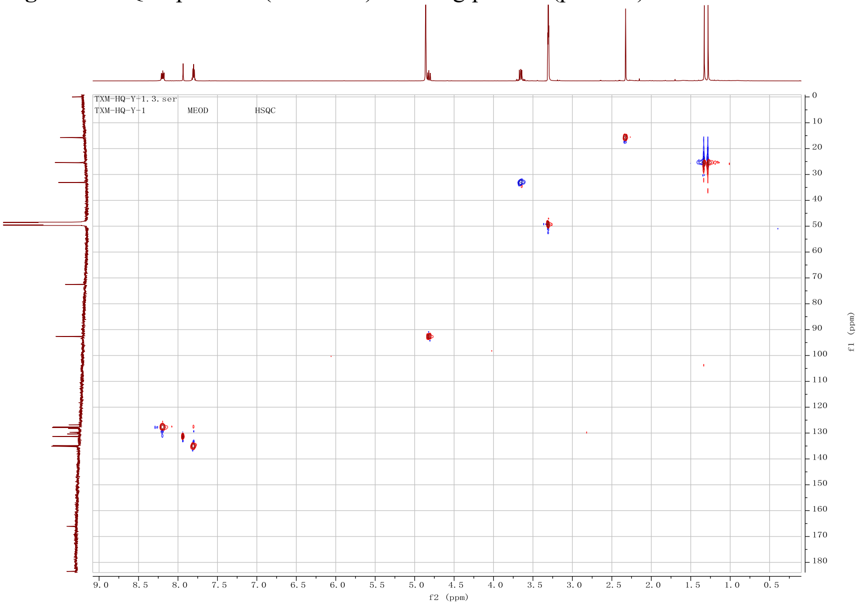

Fig. S36 HMBC spectrum (500 MHz) of astragquinone (peak **87**) in CD_3OD.

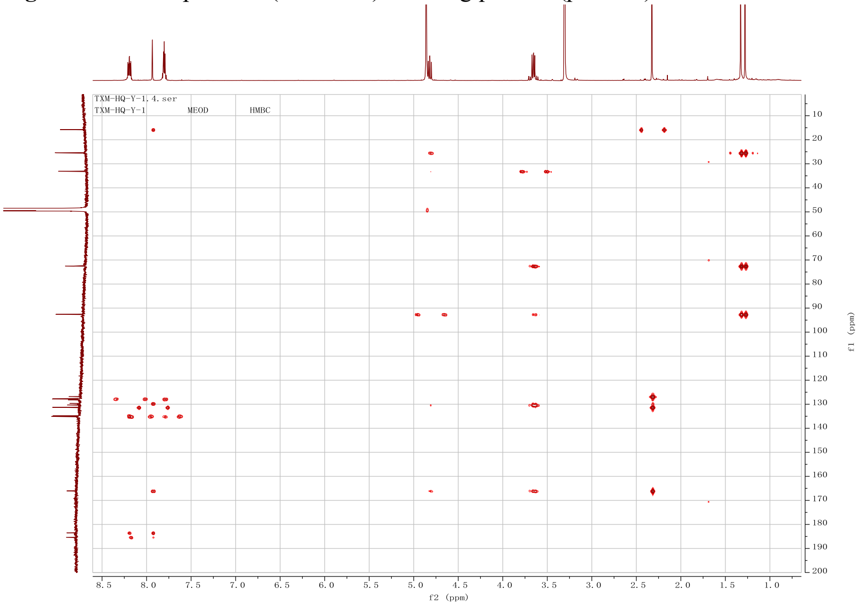

Fig. S37 ROESY spectrum (500 MHz) of astragquinone (peak **87**) in CD_3OD.

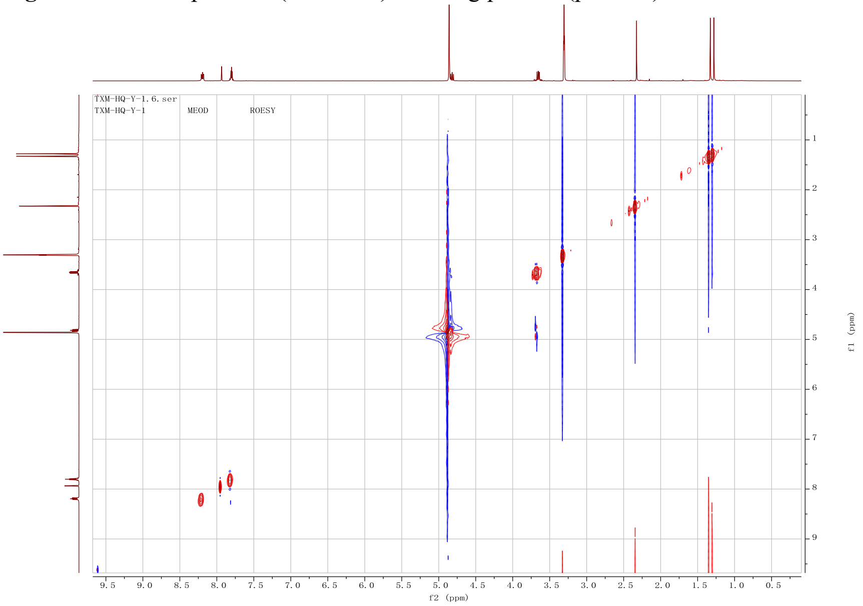

Fig. S38 UV spectrum of astragquinone (peak **87**).

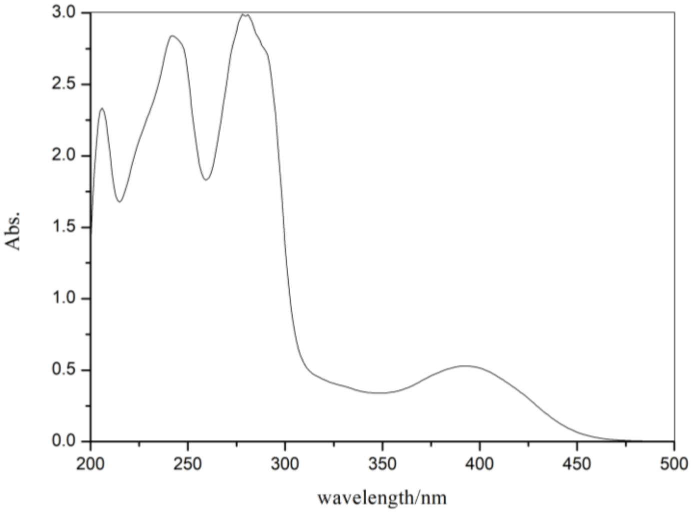

Fig. S39 IR spectrum of astragquinone (peak **87**).

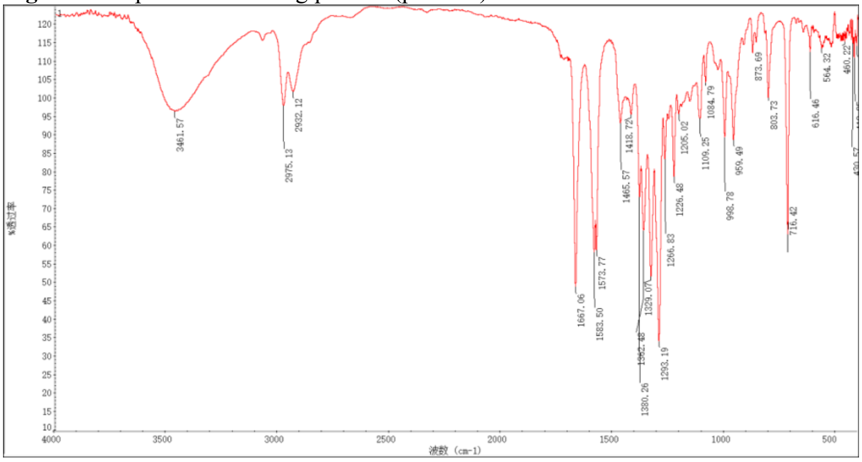

Fig. S40 CD spectrum of astragquinone (peak **87**).

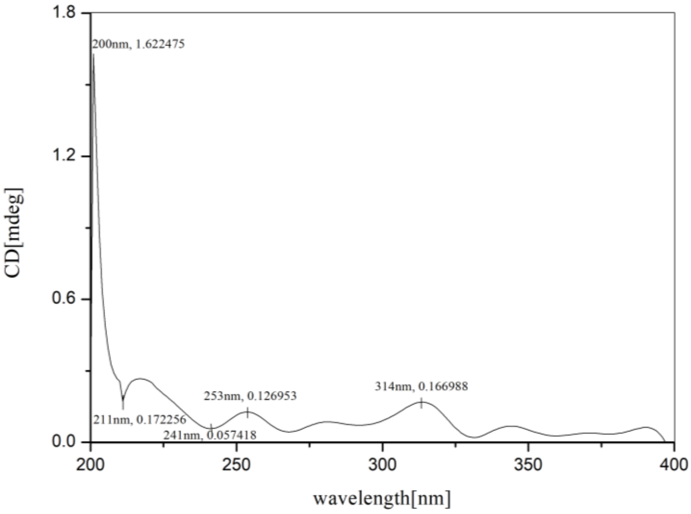

Fig. S41 ECD spectrum of astragquinone (peak **87**).

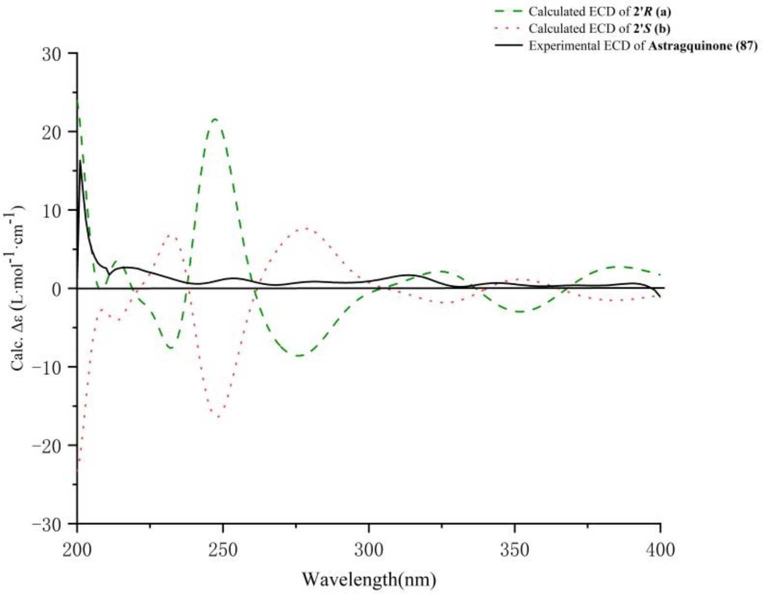

Figure S42 Dose–response curves of compound 1 on TNF-α and IL-6 production in LPS-stimulated RAW264.7 cells

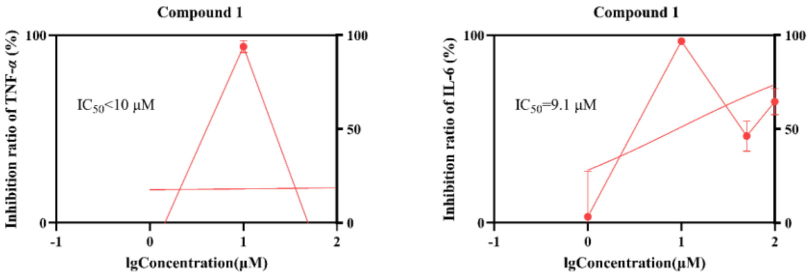

Figure S43 Dose–response curves of compound 2 on TNF-α and IL-6 production in LPS-stimulated RAW264.7 cells

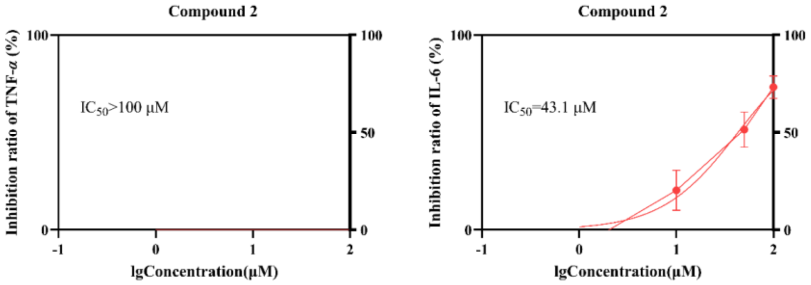

Figure S44 Dose–response curves of compound 3 on TNF-α and IL-6 production in LPS-stimulated RAW264.7 cells

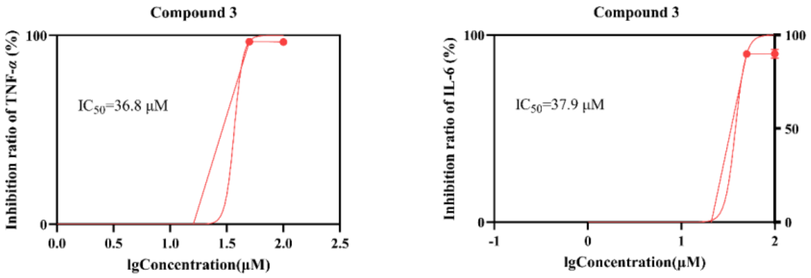

Figure S45 Dose–response curves of compound 4 on TNF-α and IL-6 production in LPS-stimulated RAW264.7 cells

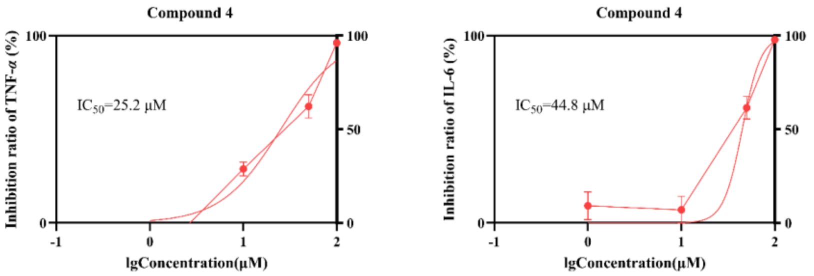

References

Chen, J., Wang, X., Xin, X., Zheng, Y., Hou, F., Li, C., Guo, L. & Wang, L., 2024. *Comprehensive comparison of two colour varieties of Perillae folium by UHPLC-Q-TOF/MS analysis combining with feature-based molecular networking.* Food Chemistry, 463, 141293. https://doi.org/10.1016/j.foodchem.2024.141293

~~Han, X.J., Yin, M.Z., Fang, Q.Y., Tan, X.M., Sun, H.B., Cheng, M.E., Peng, H.S., Huang, L.Q., 2023. Nutritional ingredients and functional components of cultivated and wild-simulated Astragali radix using widely targeted metabolomics. LWT-Food Sci. Technol. 185, 115186. https://doi.org/10.1016/j.lwt.2023.115186~~

~~Hou, J.L., Li, A.P., Wang, G.H., Qin, X.M., Liu, Y.T., 2025. Metabolomics analysis of Astragali Radix in Shanxi Province: Investigating the impact of various cultivation methods and growth years on metabolite profiles. Food Chem. 468, 142492. https://doi.org/10.1016/j.foodchem.2024.142492~~

~~Yin, M.Z., Yang, M., Chu, S.S., Li, R.Q., Zhao, Y.J., Peng, H.S., Zhan, Z.L., Sun, H.F., 2019a. Quality analysis of different specification grades of *Astragalus membranaceus* var. *mongholicus* (Huangqi) from Hunyuan, Shanxi. J. AOAC Int. 102, 734-740. https://doi.org/10.5740/jaoacint.18-0308~~

Formatted: Indent: Hanging: 0.85 cm, Line spacing: Exactly 20 pt, Don't adjust space between Latin and Asian text, Don't adjust space between Asian text and numbers
