# Supplementary material for: Molecular Networking-Guided Phytochemical Profiling and Anti-Inflammatory Evaluation of Honglanqi, an Underutilized Commercial Specification of Astragali Radix
Source: Plants (Basel). 2026 May 8;15(10):1442. doi: 10.3390/plants15101442 (PMC13211090; doi:10.3390/plants15101442)

Fig. S4 Fragmentation deduction of the annotated metabolites. (peaks **1-102**)

Peak **1** (t_R= 1.72min, $C_{22}H_{22}O_{11}$, Isoflavone-3-(4′-glucopyranosyl-5′-hydroxy-2′-methoxyphenyl)-7-hydroxy-4*H*-chromen-4-one)

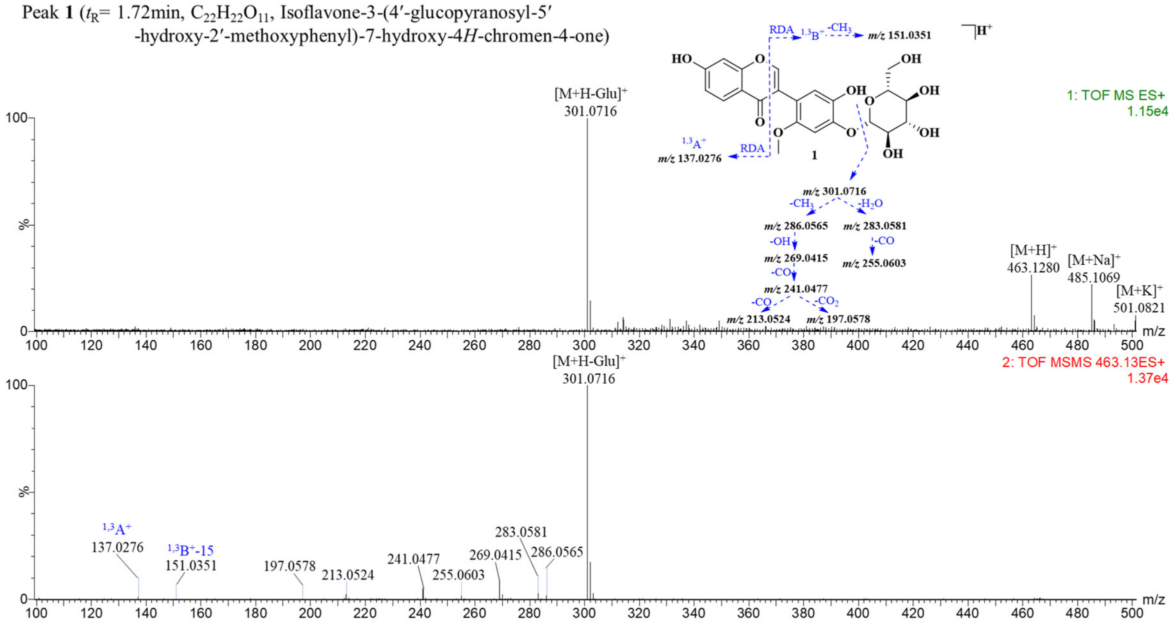

Peak **2** (t_R= 1.91min, $C_{21}H_{20}O_9$, Daidzin)

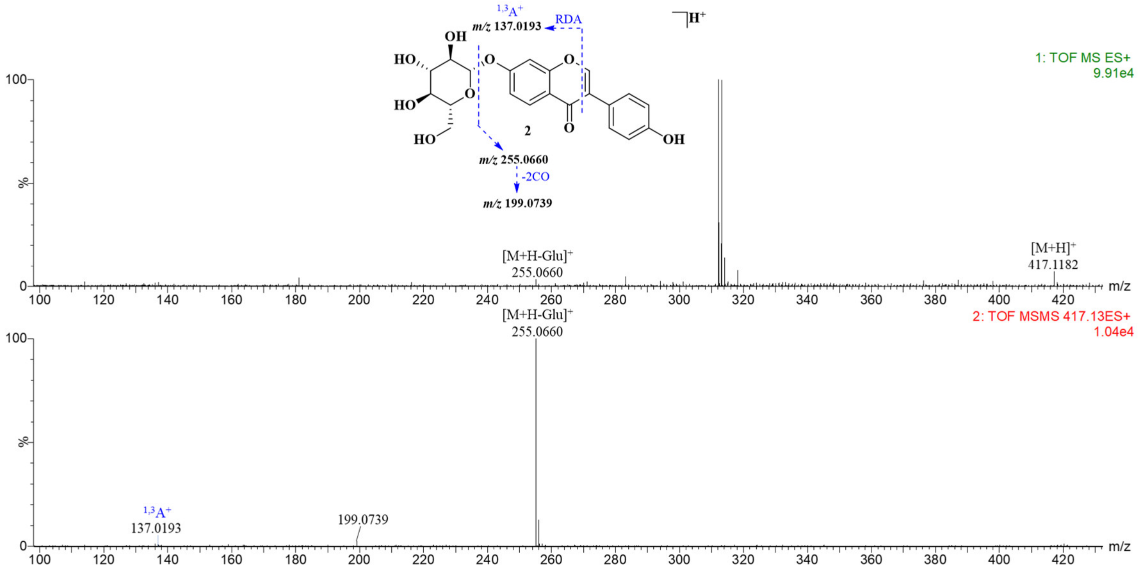

Peak **3** (t_R= 2.21min, $C_{22}H_{22}O_{10}$, 3′-hydroxy-formononetin-7-*O*-glucoside)

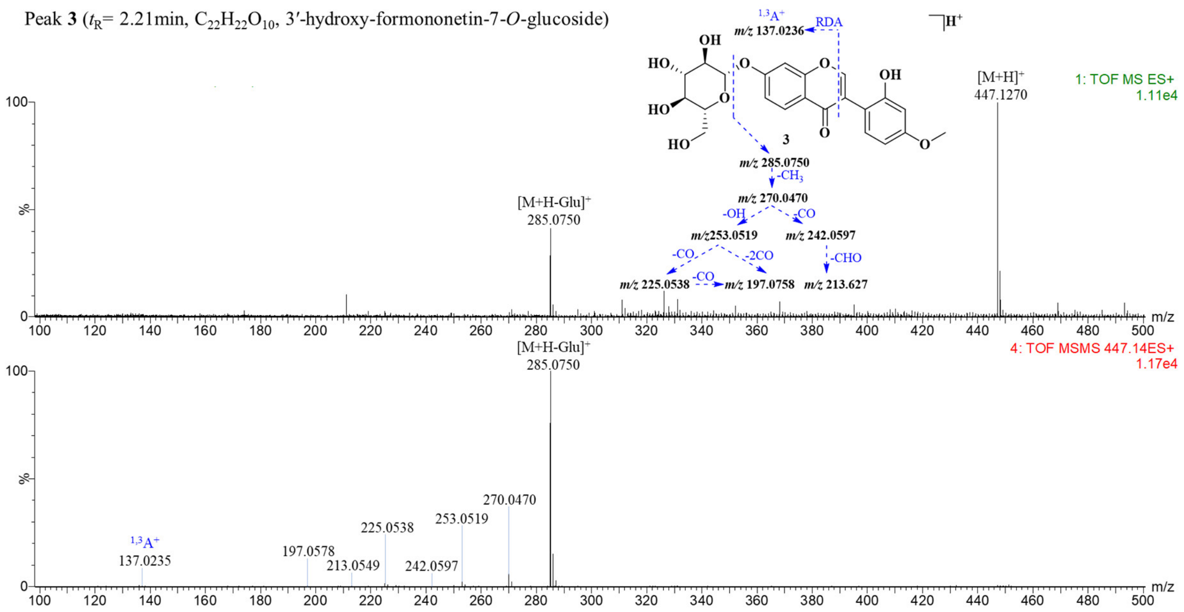

Peak **4** (t_R= 2.71min, $C_{22}H_{22}O_{10}$, Calycosin-7-*O*-*β*-D-glucoside)

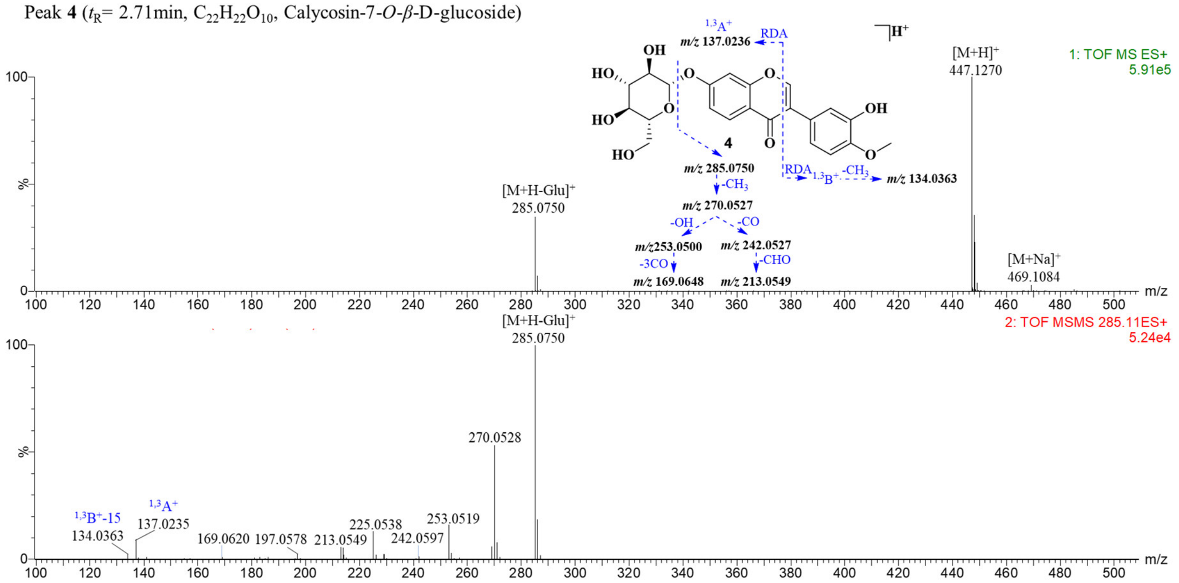

Peak **5** (t_R= 2.91min, $C_{23}H_{24}O_{11}$, Odoratin 7-*O*-*β*-D-glucopyranoside)

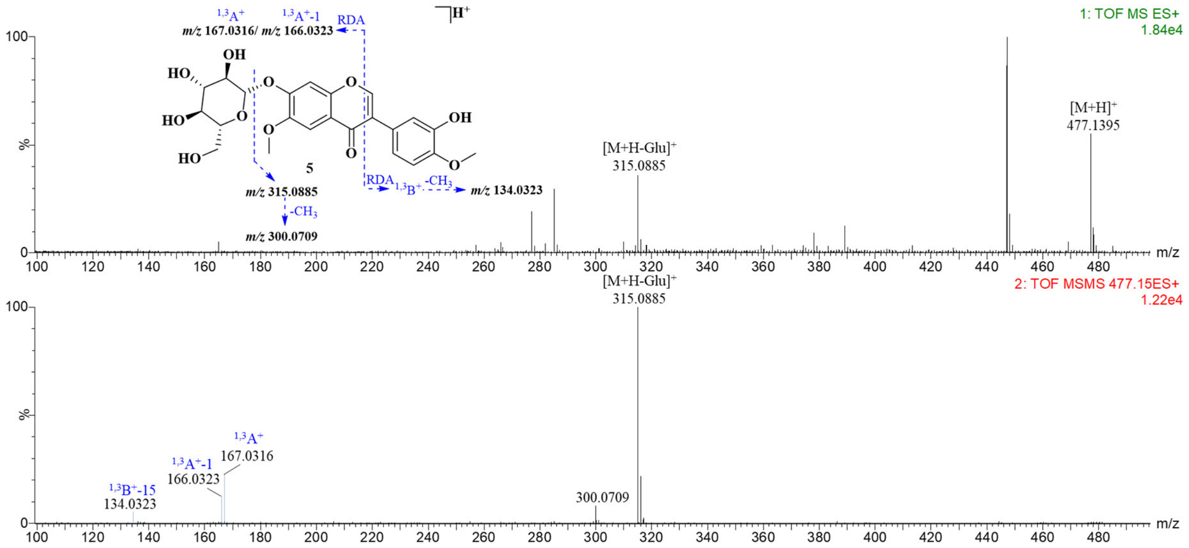

Peak **6** (t_R= 3.55min, $C_{21}H_{20}O_{10}$, Genistin)

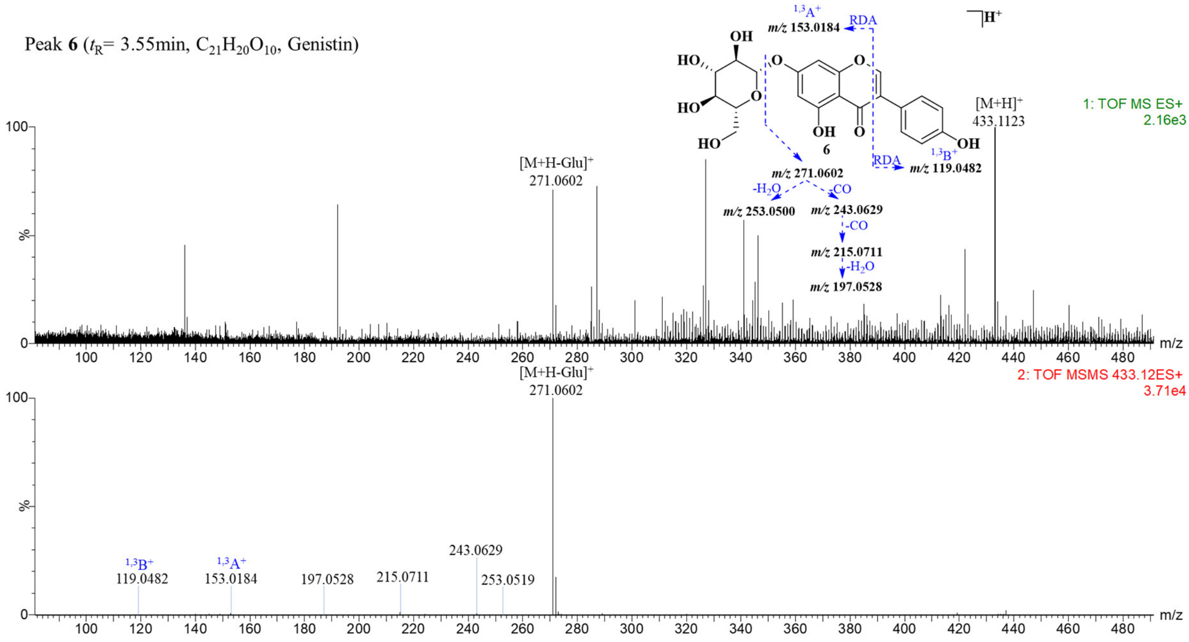

Peak **7** (t_R= 3.80min, $C_{23}H_{28}O_{11}$, Astraflavonoid C)

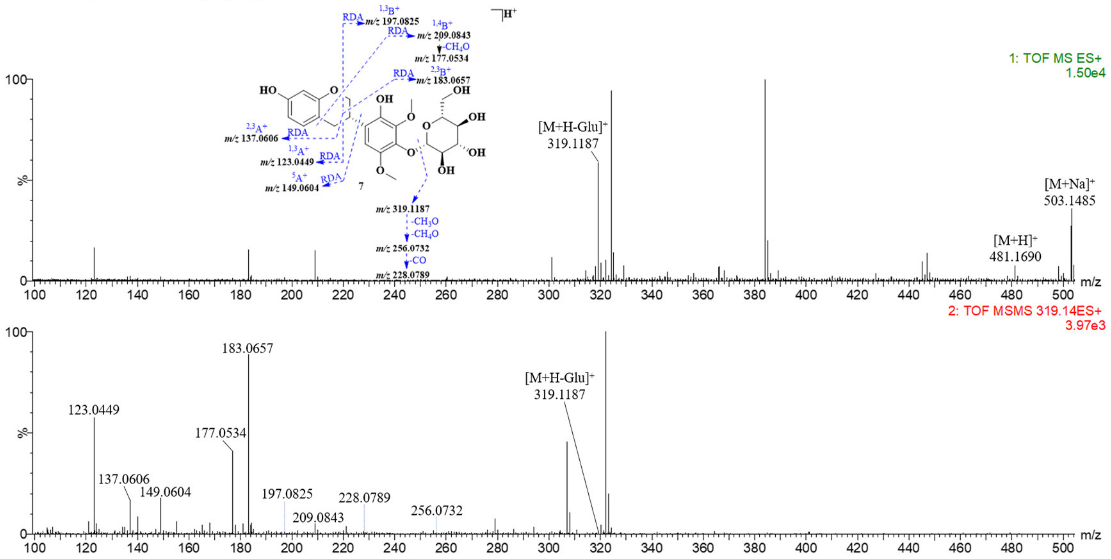

Peak **8** (t_R= 4.02min, $C_{22}H_{22}O_{11}$, Isoflavone-*O*-glycoside derivative)

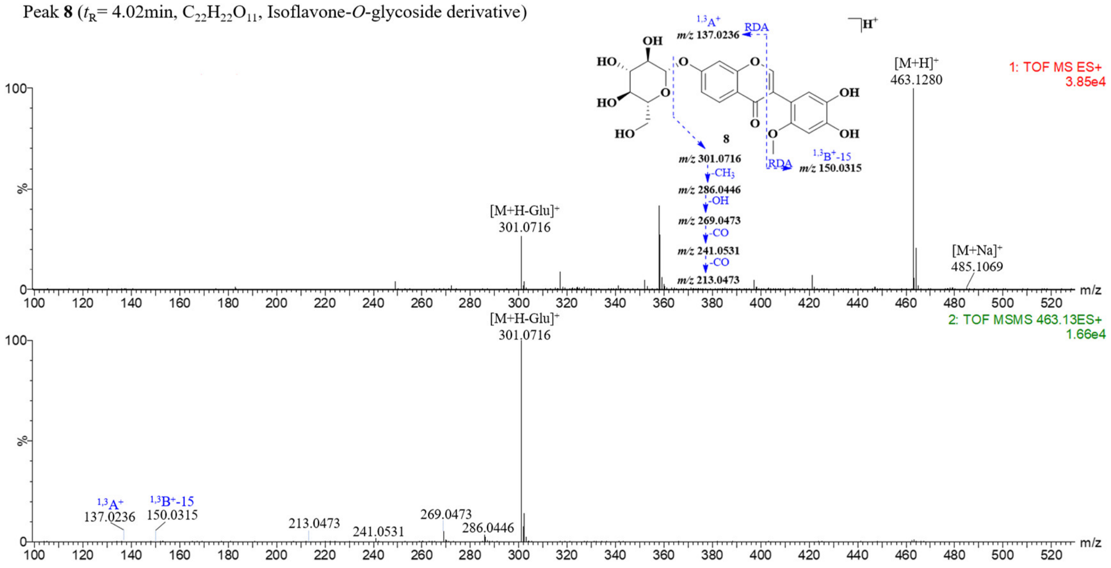

Peak **9** (t_R= 4.60min, $C_{21}H_{20}O_{10}$, Sophoricoside)

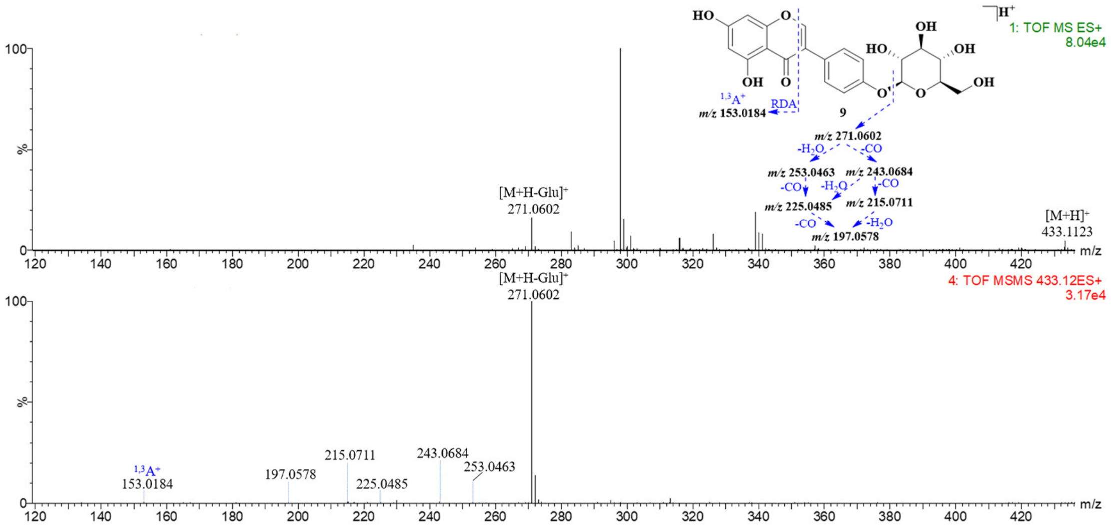

Peak **10** (t_R= 4.90min, $C_{22}H_{22}O_{11}$, Pratensein 7-*O*-*β*-D-glucopyranoside)

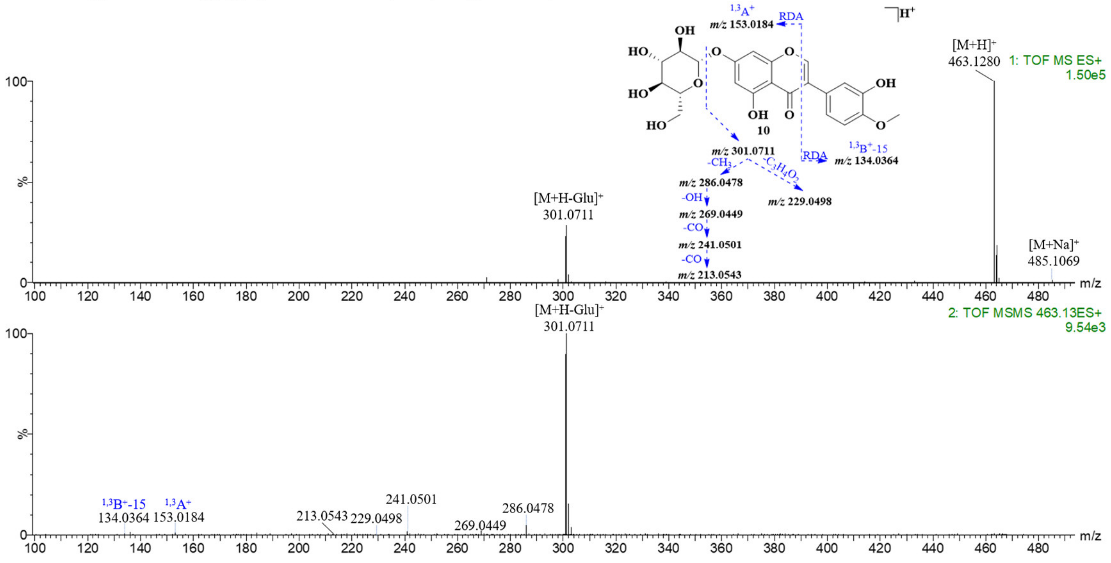

Peak **11** (t_R= 5.02min, $C_{22}H_{24}O_{10}$, Licoagroside D)

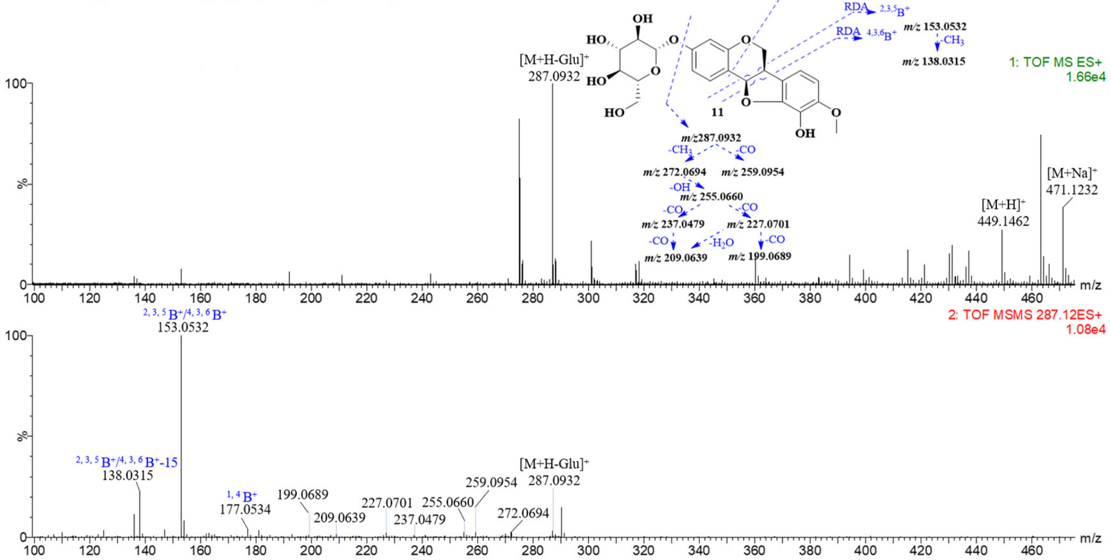

Peak **12** (t_R= 5.44min, $C_{22}H_{24}O_{11}$, Pterocarpane-*O*-glycoside)

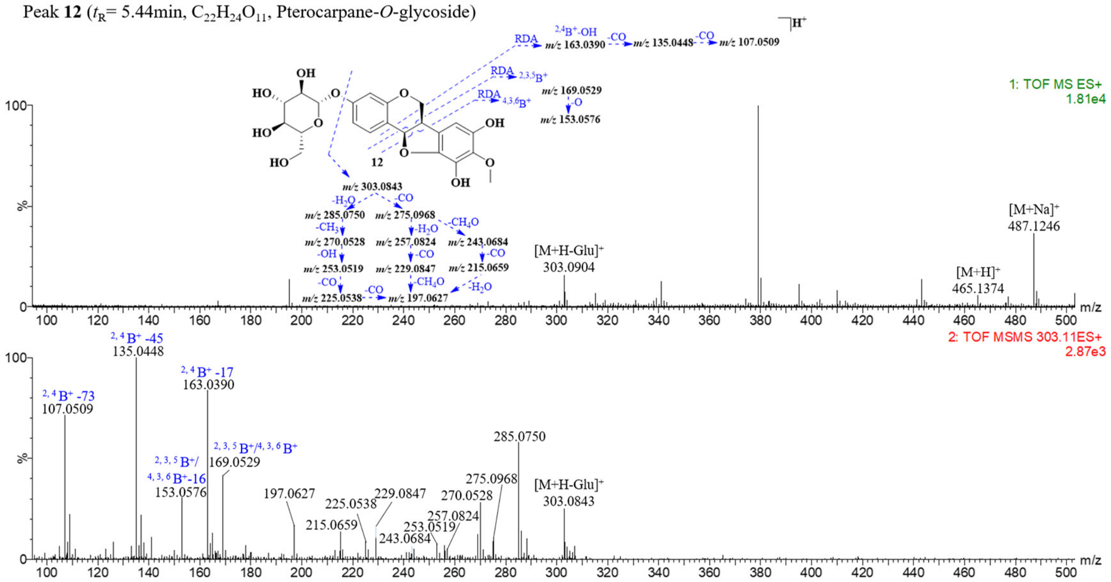

Peak **13** (t_R= 5.62min, $C_{24}H_{24}O_{11}$, Calycosin 7-*O*-Glc-4″-*O*-Ace)

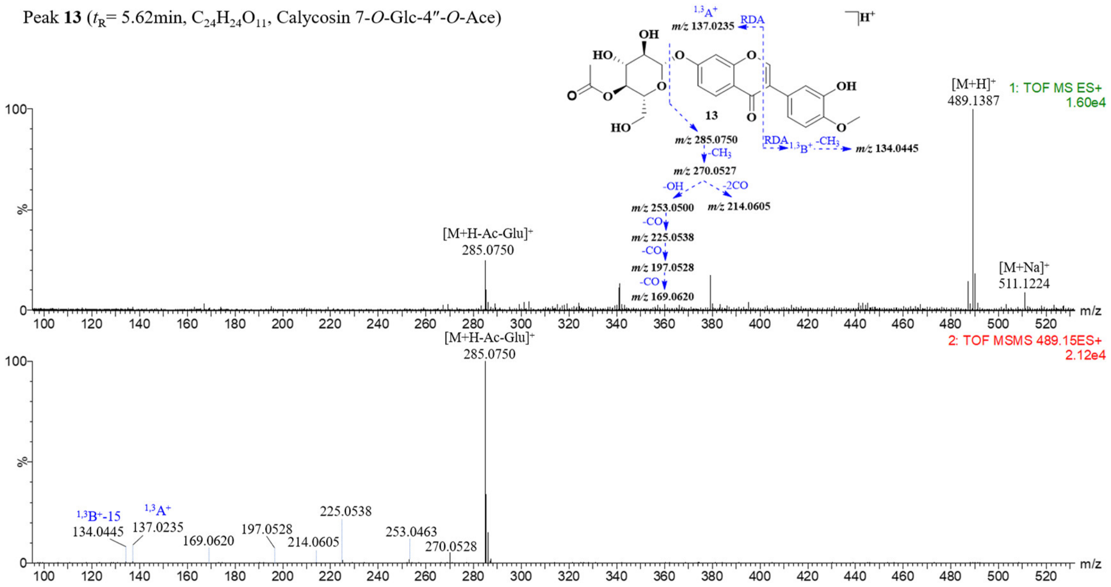

Peak **14** (t_R= 5.86min, $C_{23}H_{26}O_{11}$, Isoflavone-*O*-glycoside derivative)

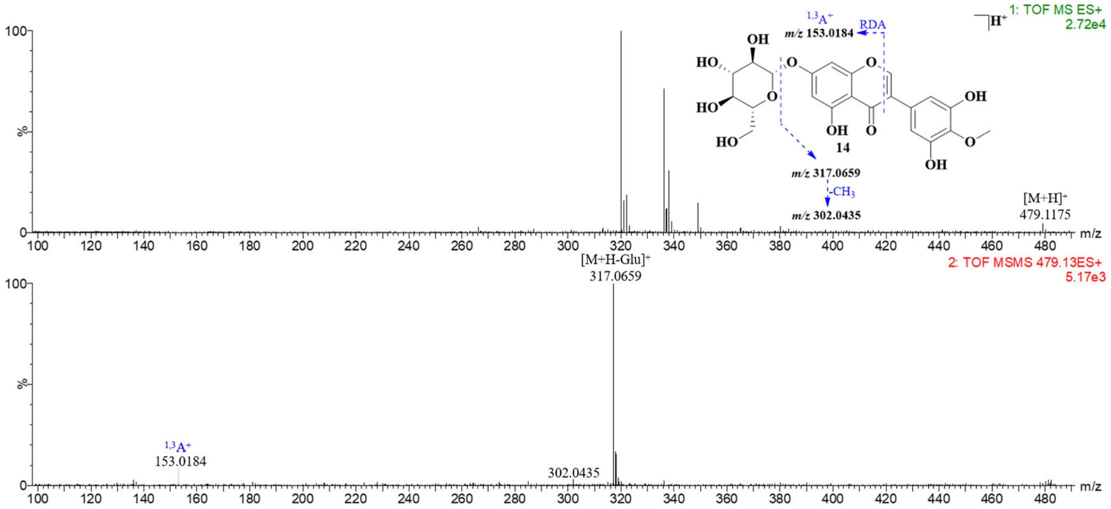

Peak **15** (t_R= 6.57min, $C_{27}H_{30}O_{13}$, Formononetin-Glc-Api)

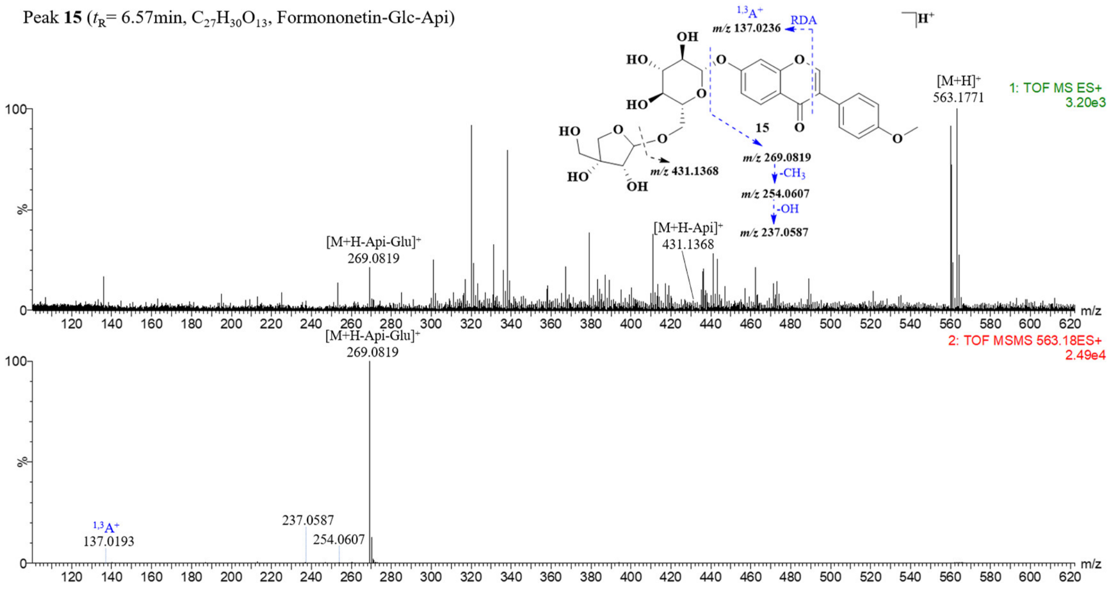

Peak **16** (t_R= 6.63min, $C_{24}H_{24}O_{11}$, Calycosin 7-*O*-Glc-2″-*O*-Ace isomer)

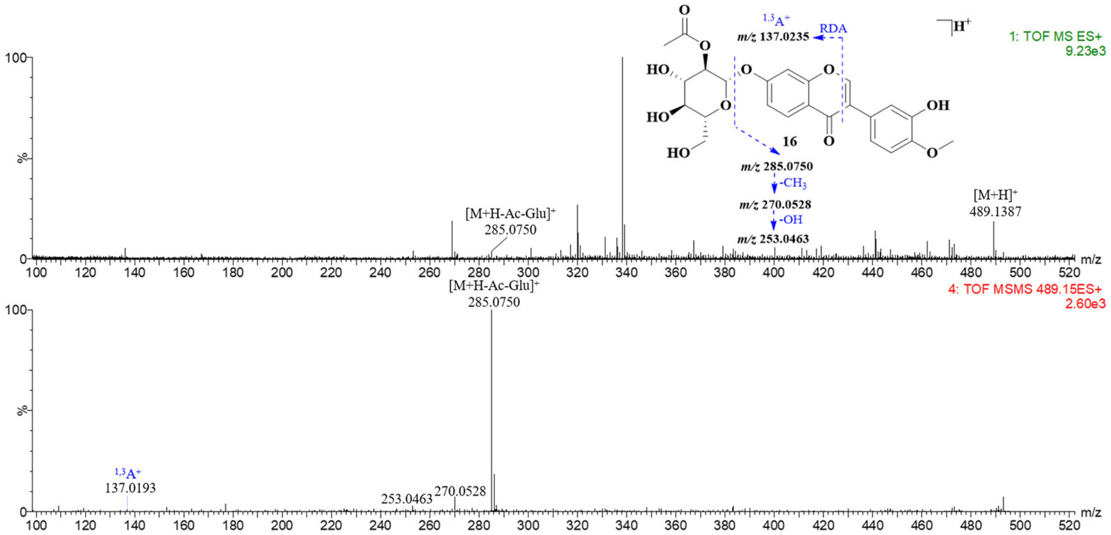

Peak **17** (t_R= 7.42min, $C_{22}H_{22}O_9$, Ononin)

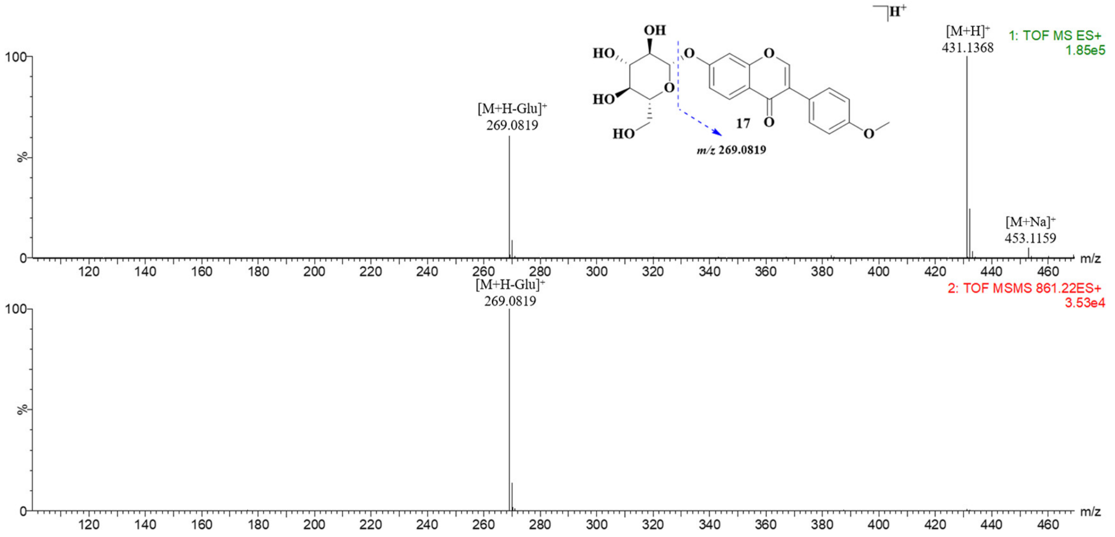

Peak **18** (t_R= 8.07min, $C_{24}H_{24}O_{11}$, Calycosin 7-*O*-Glc-6″-*O*-Ace)

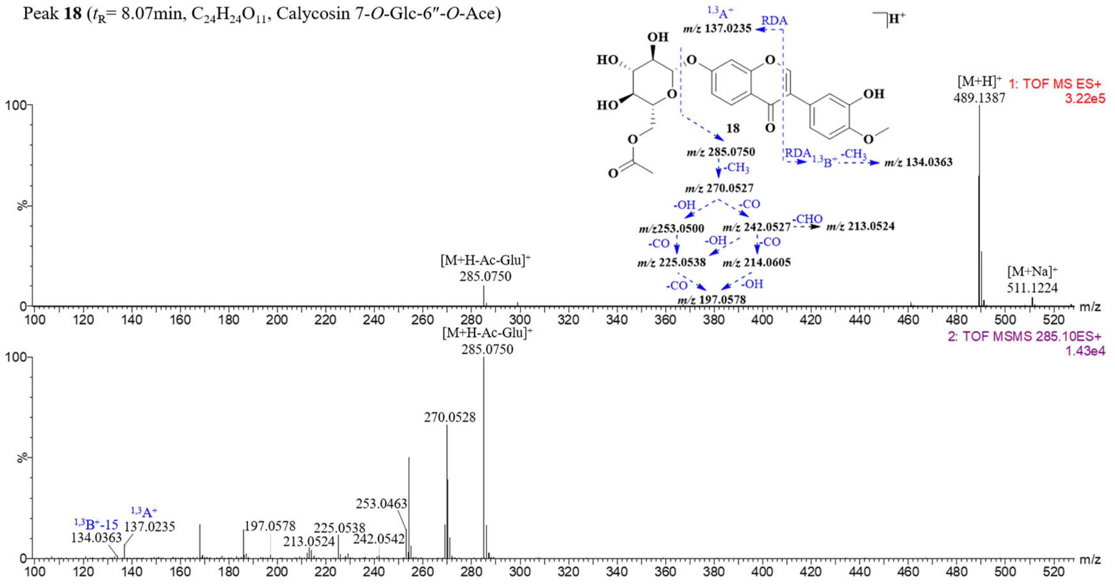

Peak **19** (t_R= 8.78min, $C_{28}H_{34}O_{14}$, Astrapterocarpanoside A)

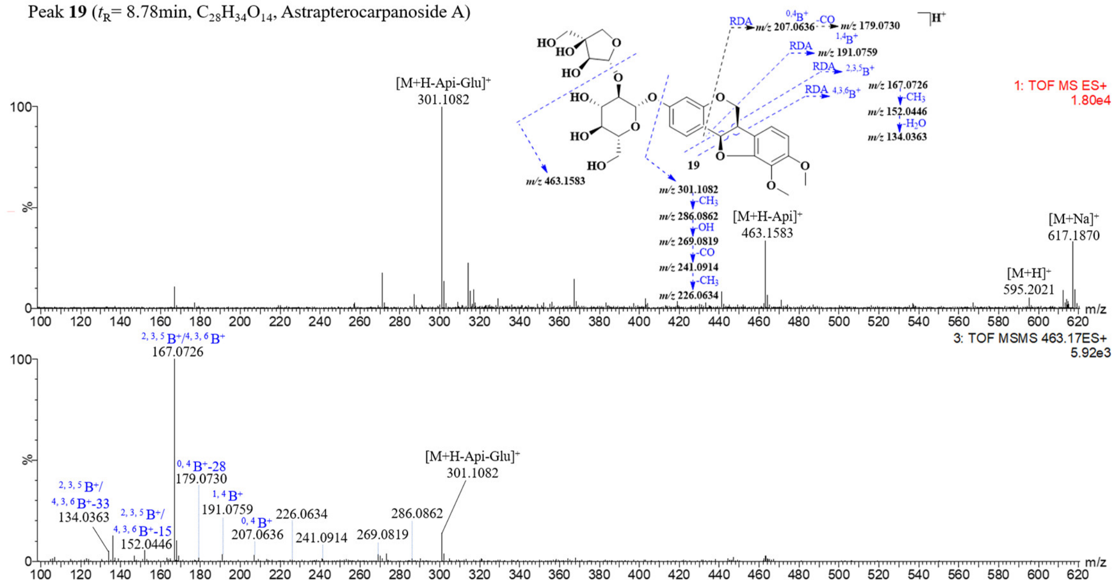

Peak **20** (t_R= 9.00min, $C_{25}H_{26}O_{12}$, Odoratin-7-*O*-Glc-Ace)

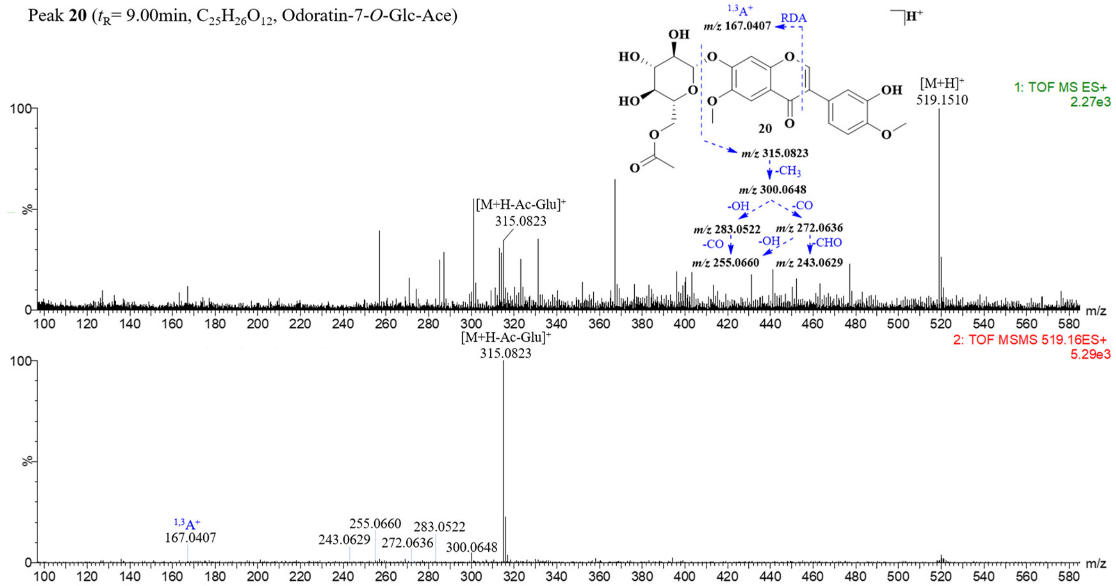

Peak **21** (t_R= 9.14min, $C_{16}H_{12}O_6$, Koparin)

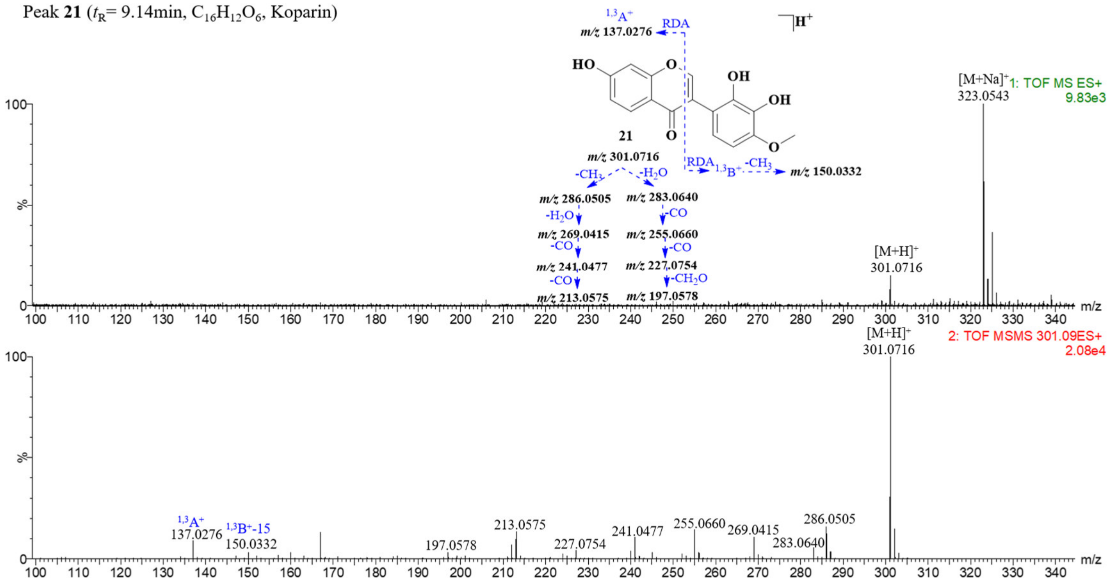

Peak **22** (t_R= 9.41min, $C_{16}H_{14}O_6$, Pterocarpans)

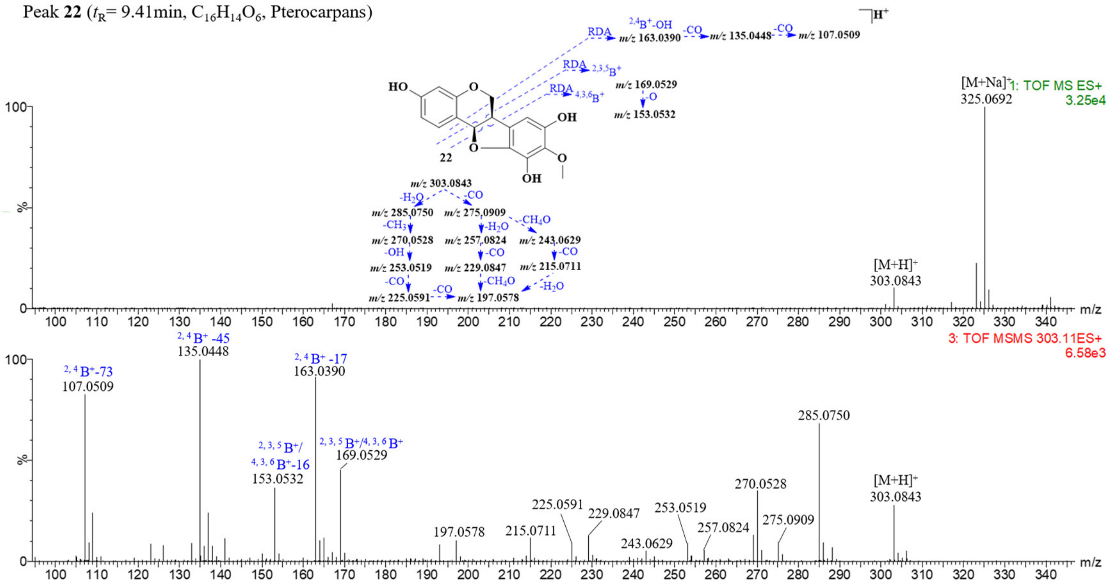

Peak **23** (t_R= 10.08min, $C_{23}H_{26}O_{10}$, Methylnissolin-3-*O*-glucoside)

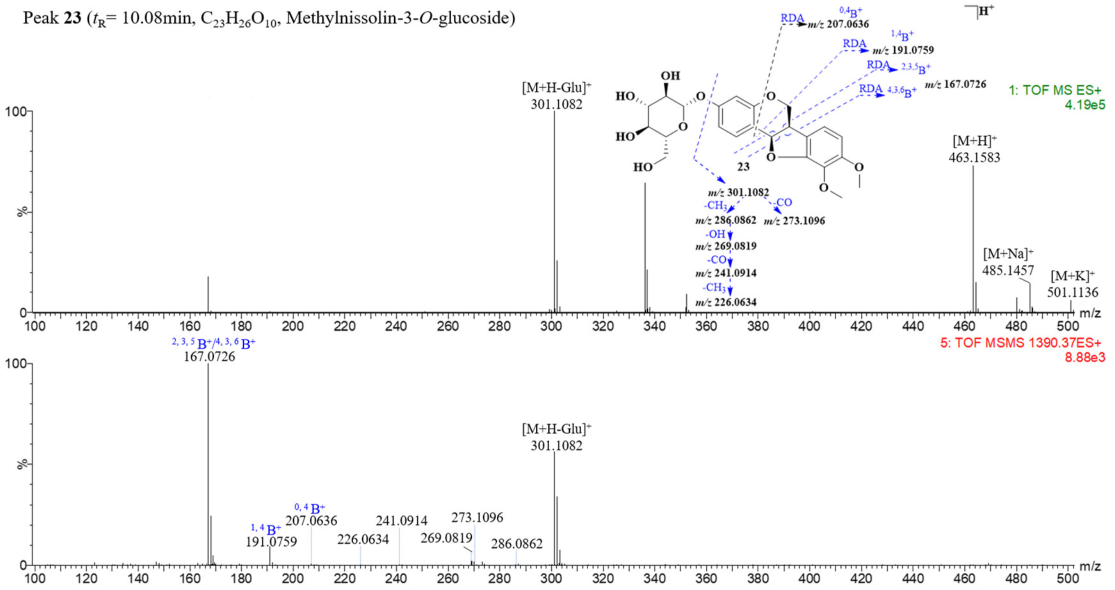

Peak **24** (t_R= 11.53min, $C_{16}H_{12}O_5$, Calycosin)

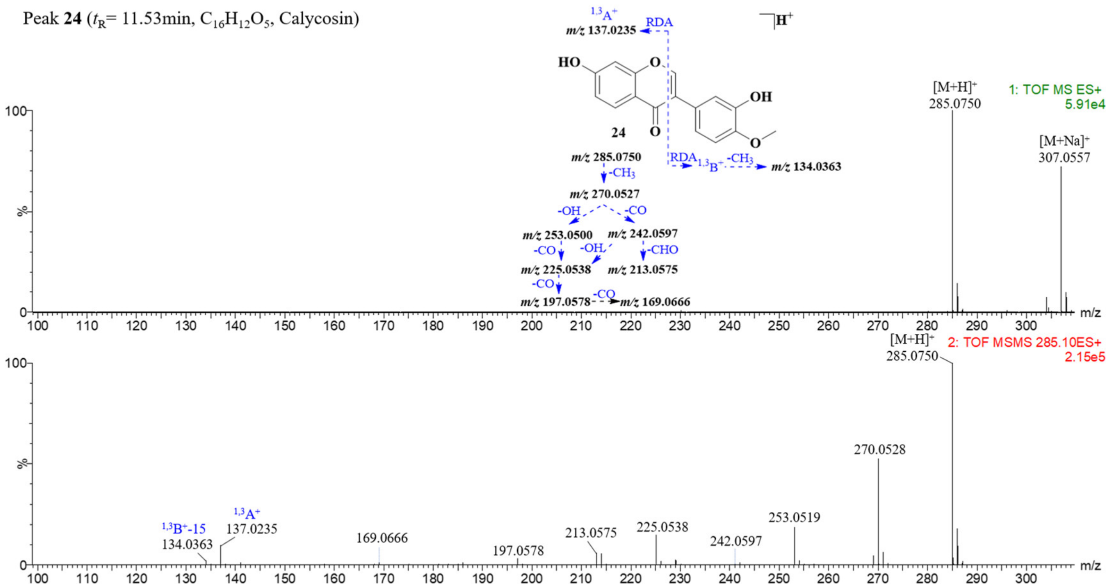

Peak **25** (t_R= 11.73min, $C_{22}H_{22}O_{11}$, 5-hydroxy-3-[4-hydroxy-2-[(2*S*,3*R*,4*S*,5*S*,6*R*)-3,4,5-trihydroxy-6-(hydroxymethyl)oxan-2-yl]oxyphenyl] -7-methoxychromen-4-one)

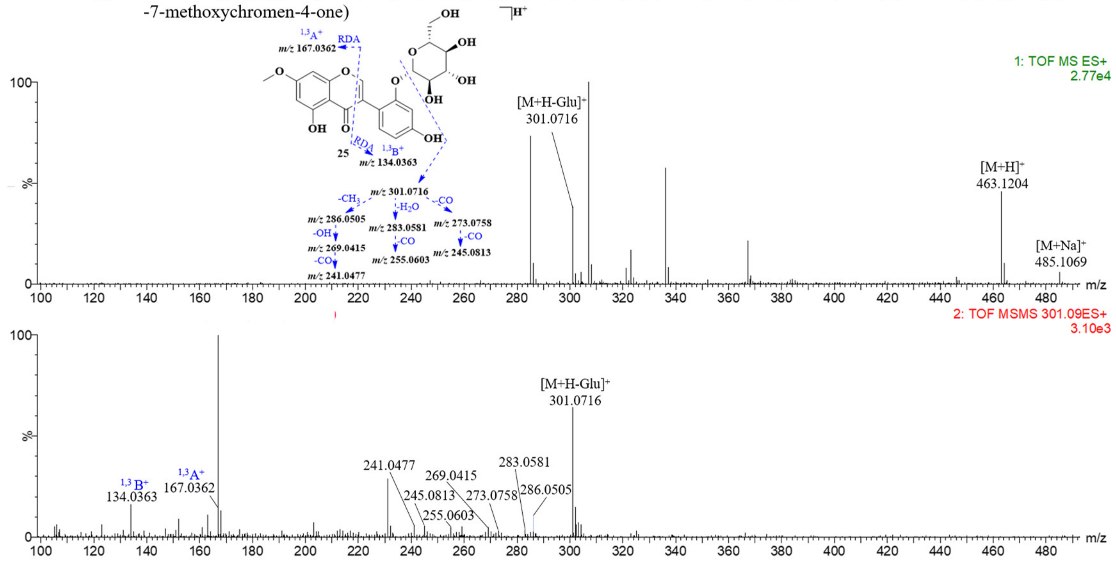

Peak **26** (t_R= 12.14min, $C_{23}H_{28}O_{10}$, Isomucronulatol-7-*O*-*β*-D-glucopyranoside)

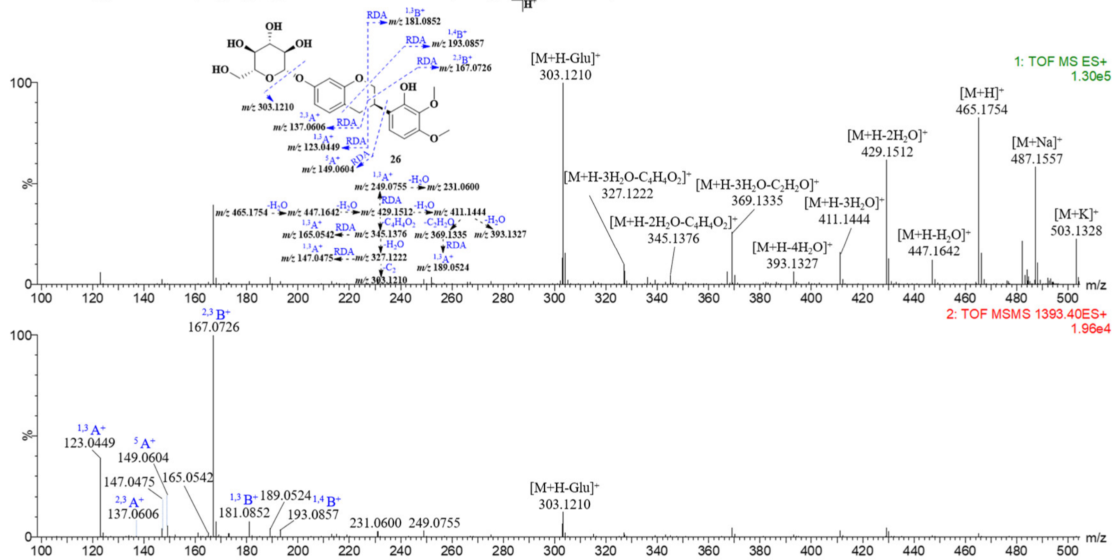

Peak **27** (t_R= 12.41min, $C_{23}H_{28}O_{10}$, Isomucronulatol-7-*O*-*β*-D-glucopyranoside isomer)

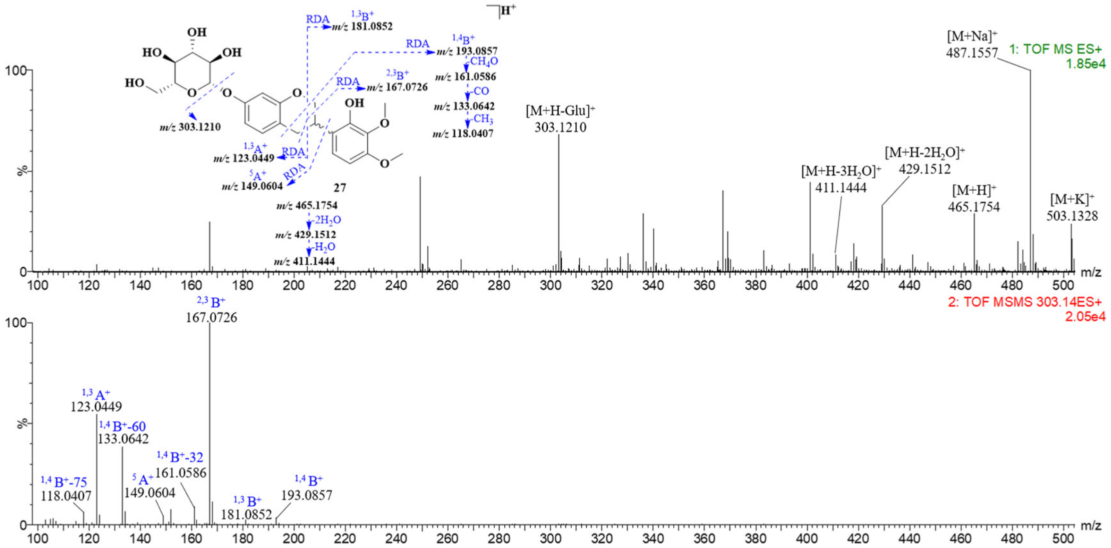

Peak **28** (t_R= 13.10min, $C_{24}H_{24}O_{12}$, 4''-*O*-acetyl-pratensein-7-*O*-*β*-D-glucoside isomer)

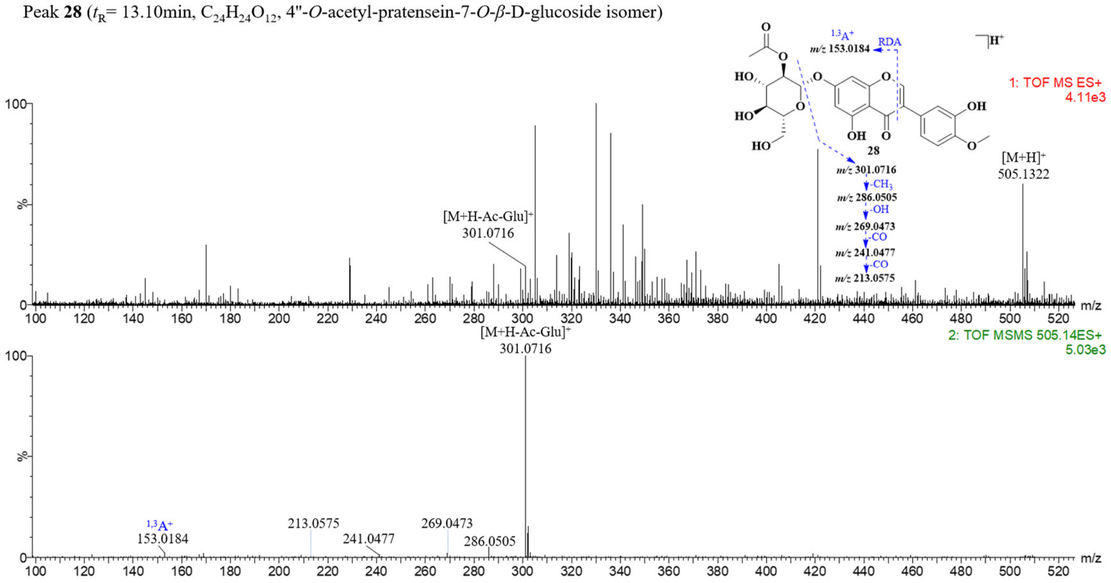

Peak **29** (t_R= 13.40min, $C_{22}H_{24}O_9$, Medicarpin-3-*O*-*β*-D-glucoside)

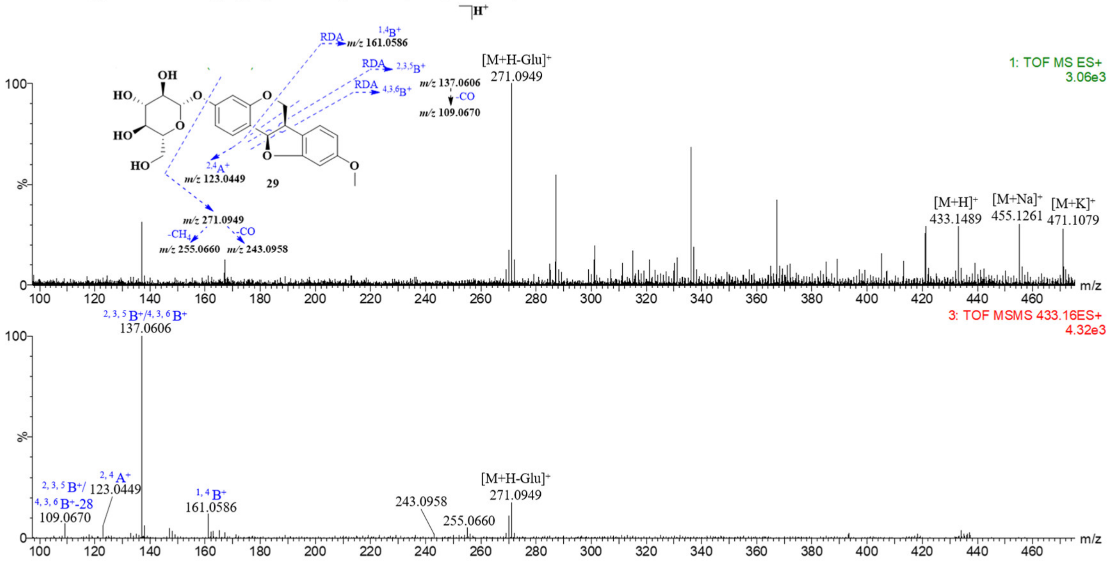

Peak **30** (t_R= 13.69min, $C_{17}H_{14}O_6$, Odoratin)

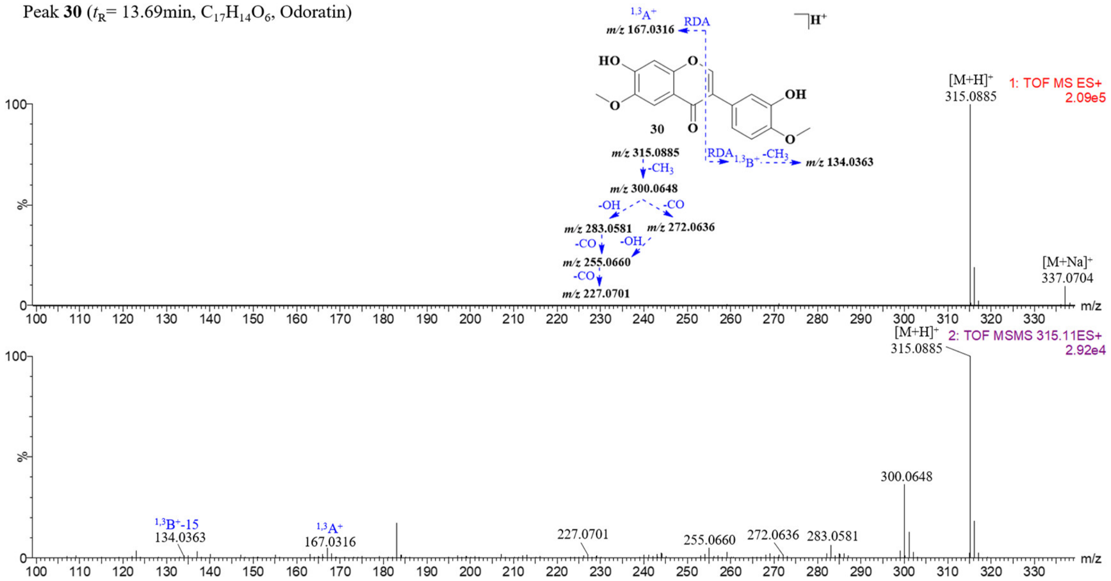

Peak **31** (t_R= 14.82min, $C_{24}H_{24}O_{10}$, Formononetin-7-*O*-Glc-4''-*O*-Ace)

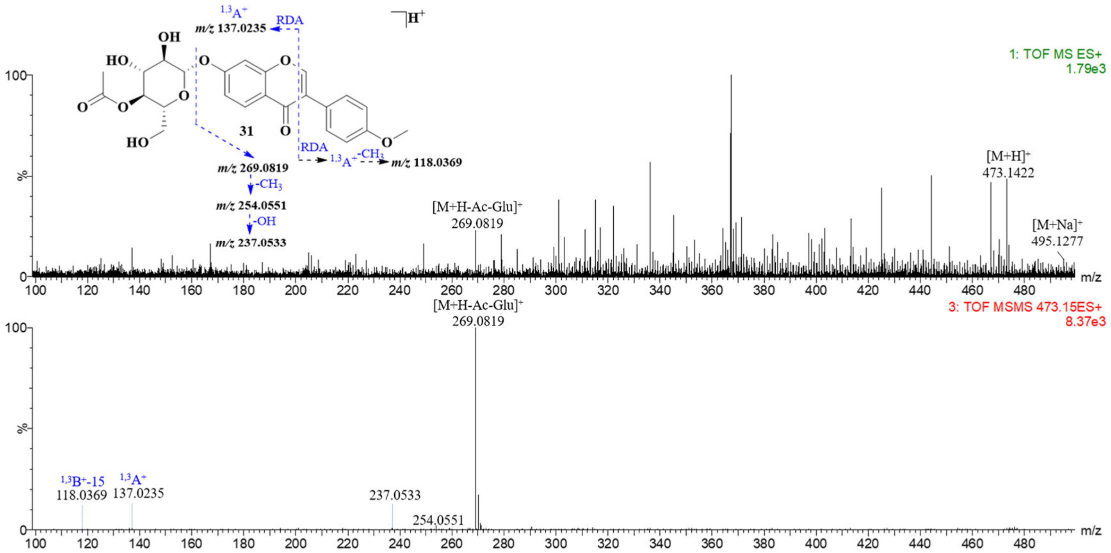

Peak **32** (t_R= 15.20min, $C_{16}H_{14}O_5$, Vesticarpan)

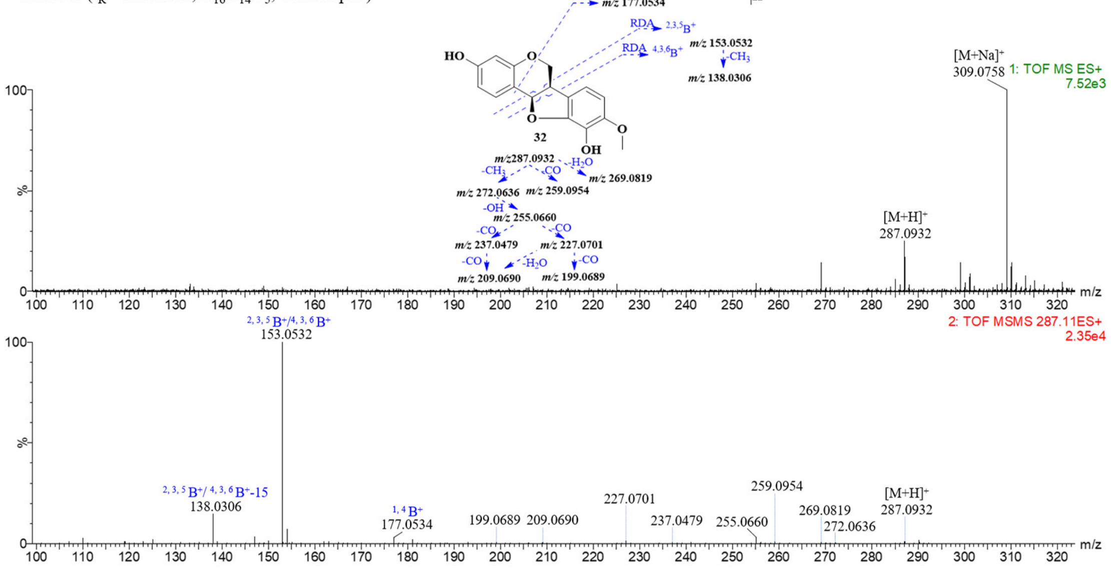

Peak **33** (t_R= 15.22min, $C_{24}H_{24}O_{10}$, 2"-Acetyl-ononin isomer)

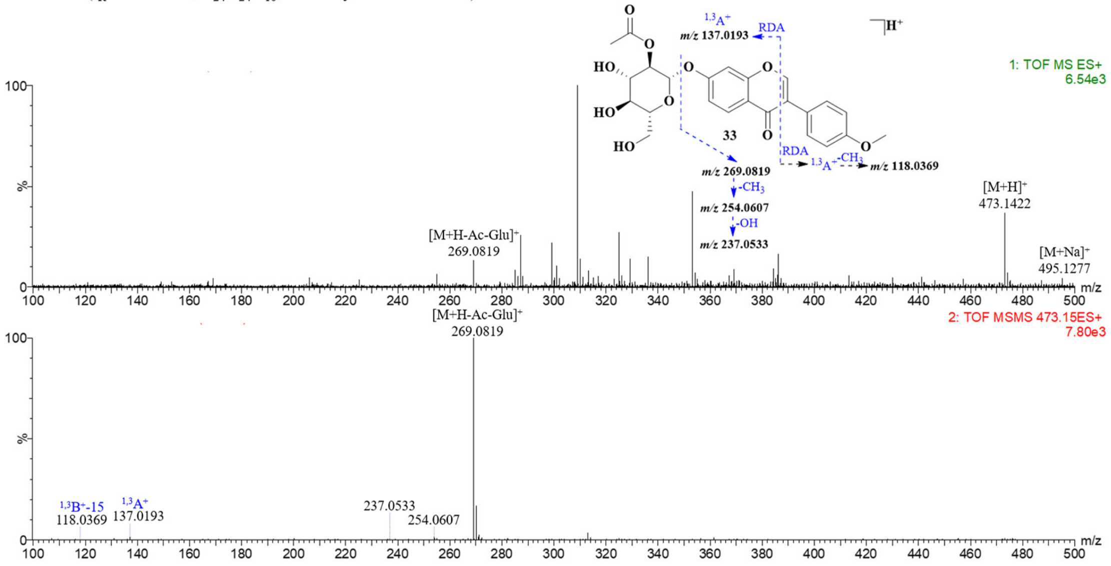

Peak **34** (t_R= 15.57min, $C_{17}H_{14}O_5$, 4´-hydroxy-7,3´-dimethoxyisoflavone)

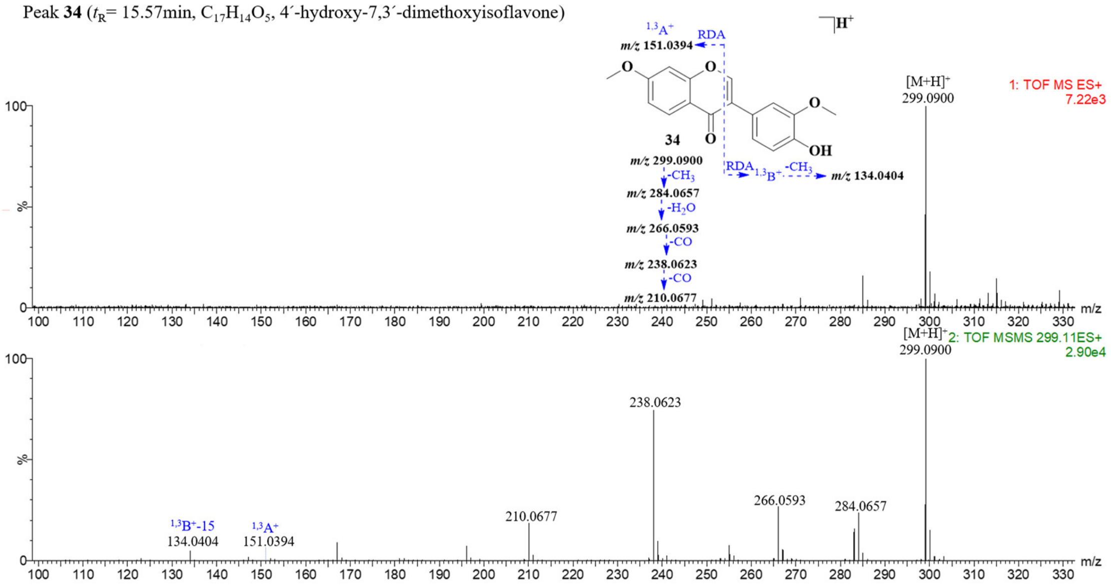

Peak **35** (t_R= 15.69min, $C_{22}H_{22}O_{10}$, Sissotrin)

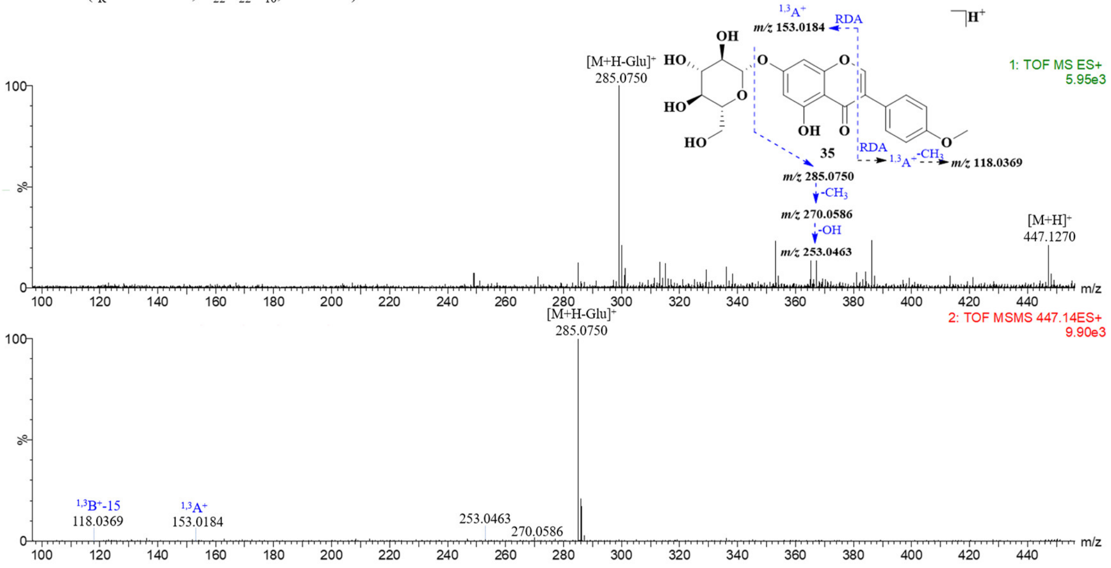

Peak **36** (t_R= 15.77min, $C_{17}H_{14}O_6$, 7,2´-Dihydroxy-3´,4´-dimethoxyisoflavone)

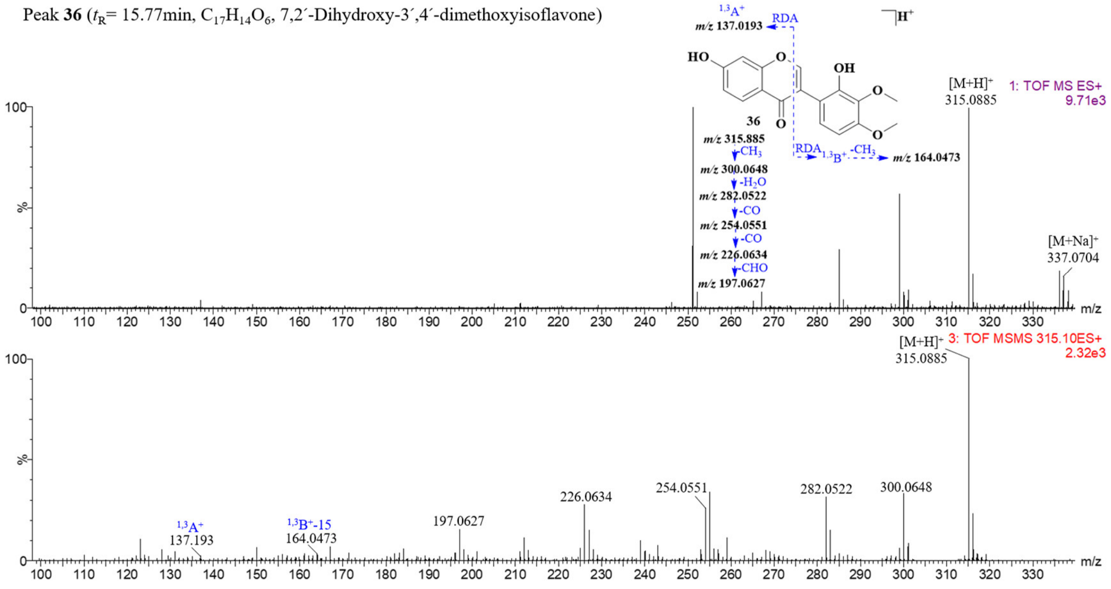

Peak **37** (t_R= 15.82min, $C_{24}H_{24}O_{12}$, 6''-*O*-acetyl-pratensein-7-*O*-*β*-D-glucoside)

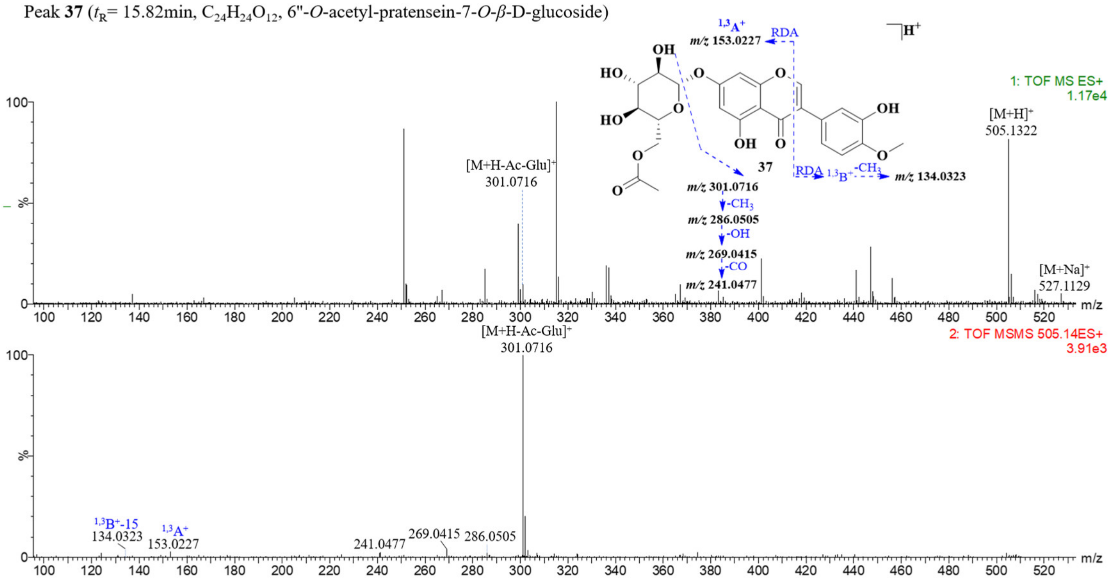

Peak **38** (t_R= 18.25min, $C_{25}H_{28}O_{11}$, Methylinissolin 3-*O*-*β*-D-(6'-acetyl) -glucoside isomer)

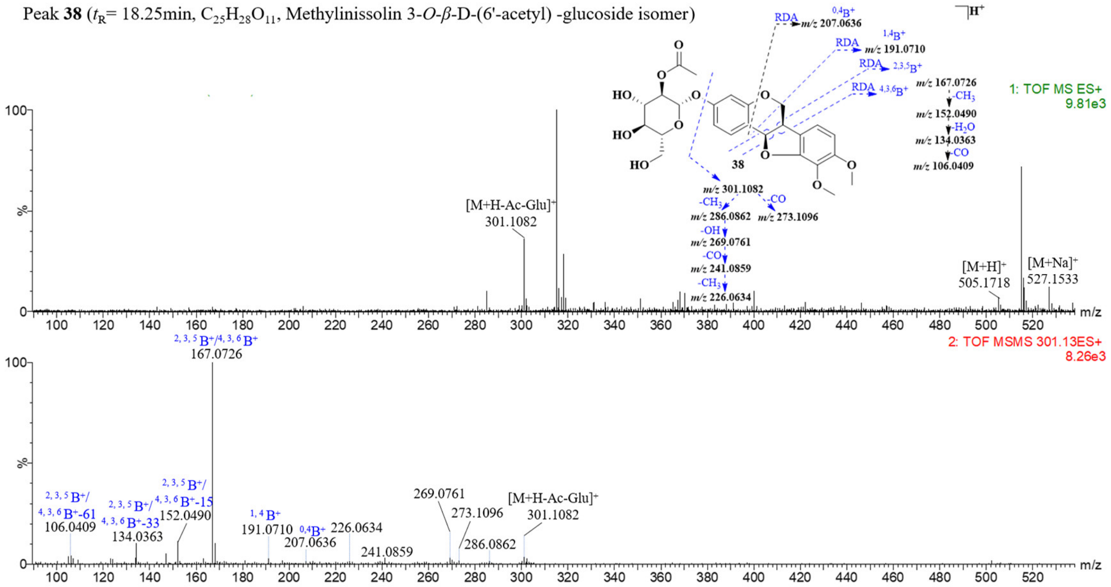

Peak **39** (t_R= 18.28min, $C_{26}H_{26}O_{11}$, Calycosin 7-*O*-*β*-D-{6''-(*E*)-but-2-enoyl}-glucopyranoside)

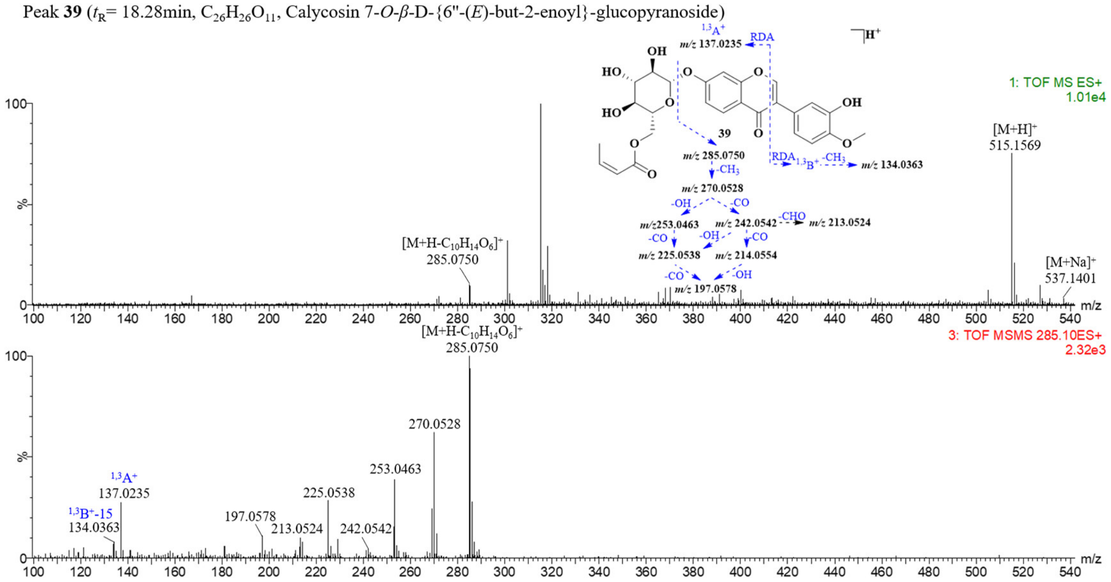

Peak **40** (t_R= 18.29min, $C_{18}H_{16}O_7$, 3',7-Dihydroxy-2',4',6-trimethoxyisoflavone)

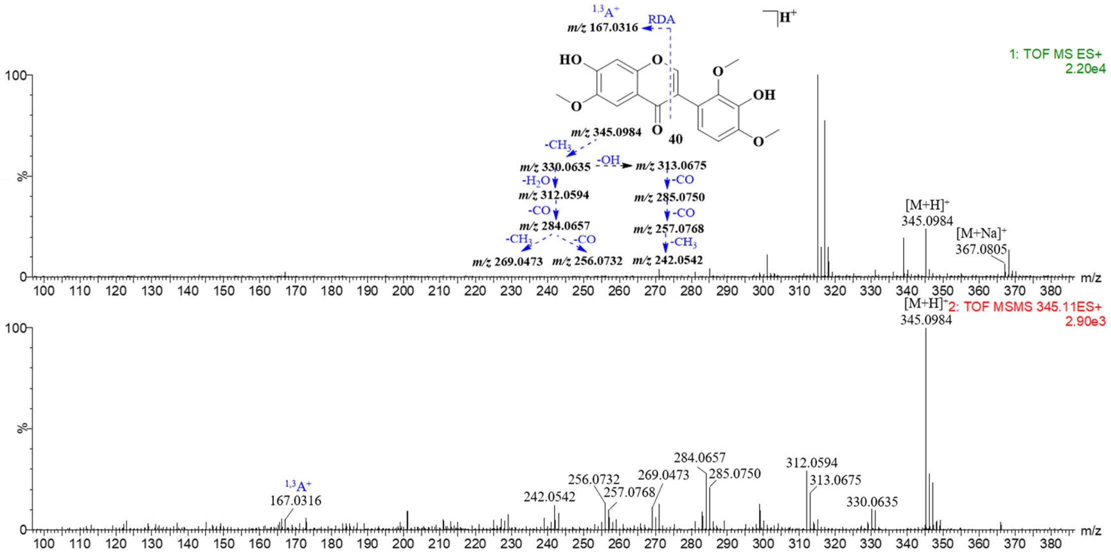

Peak **41** (t_R= 18.49min, $C_{17}H_{16}O_6$, (6a*R*,11a*R*)-3,8-dihydroxy-9,10-dimethoxypterocarpan)

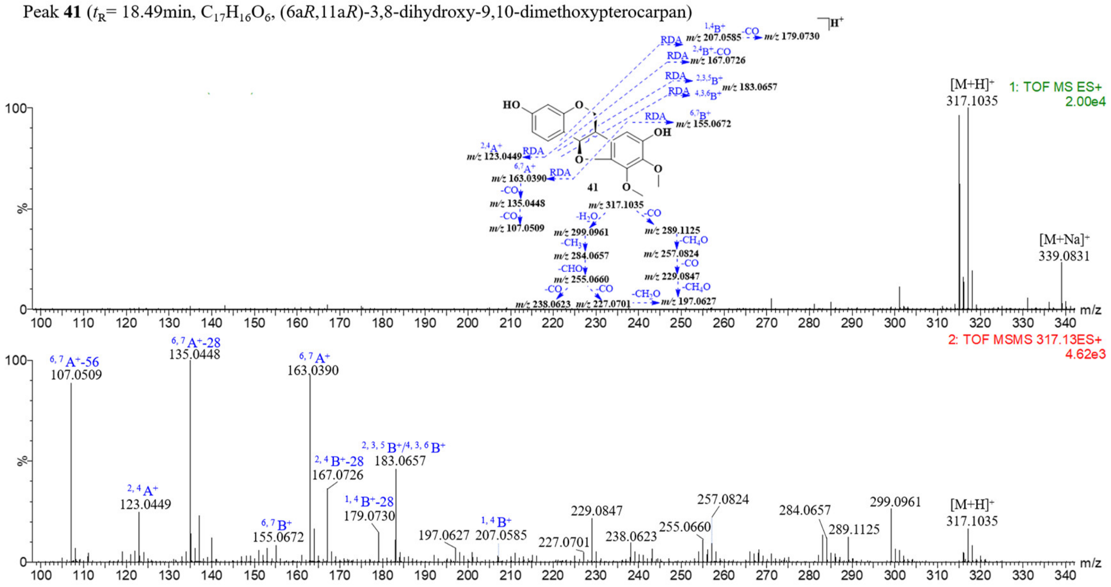

Peak **42** (t_R= 19.26min, $C_{17}H_{14}O_5$, 7-Hydroxy-3',4'-dimethoxyisoflavone)

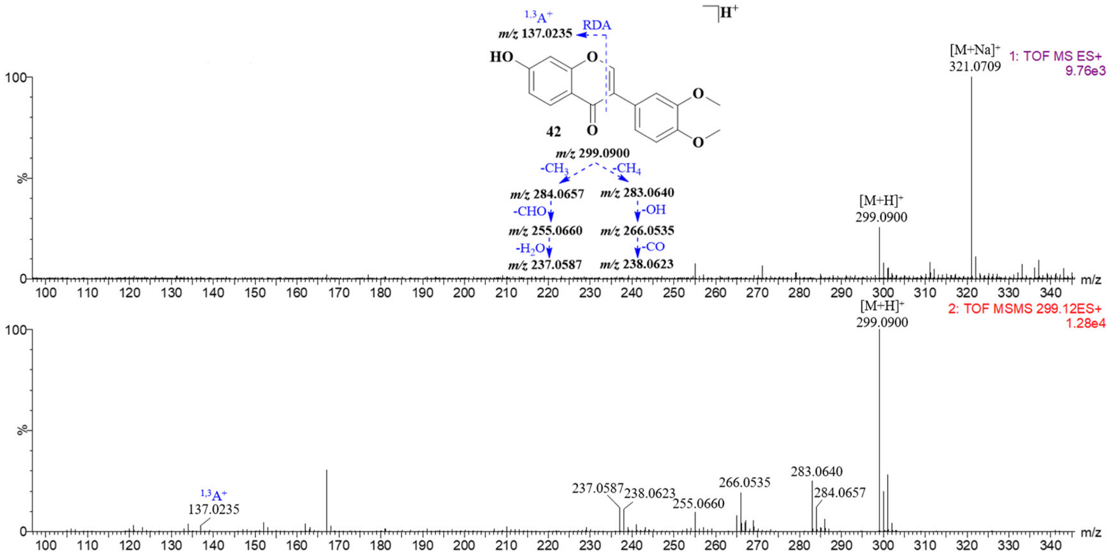

Peak **43** (t_R= 19.68min, $C_{17}H_{14}O_6$, Fistulaflavonoid B)

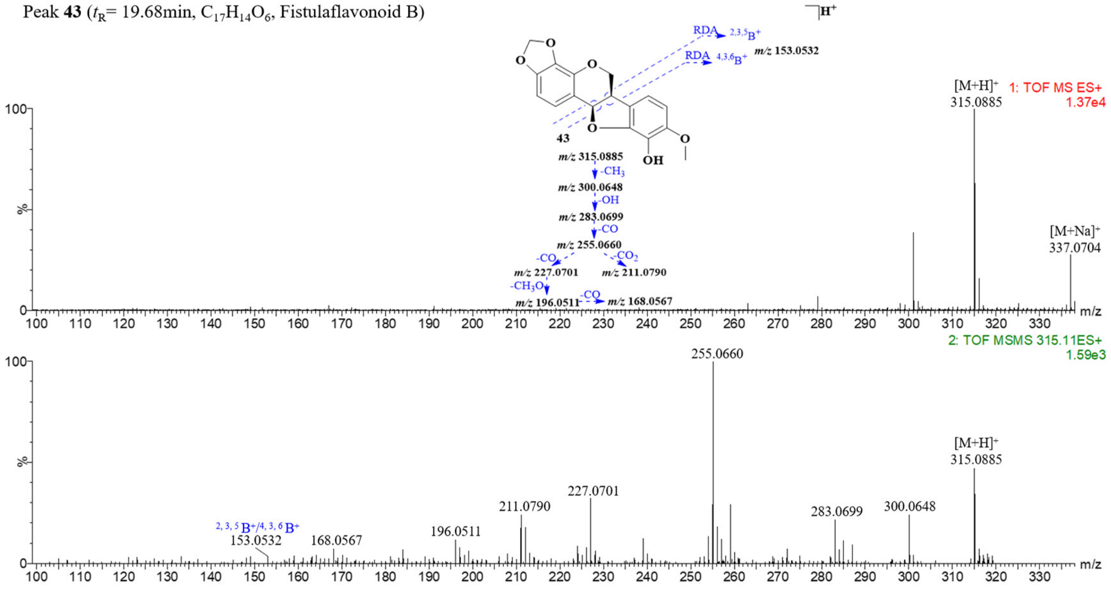

Peak **44** (t_R= 19.81min, $C_{15}H_{10}O_3$, 2-(Hydroxymethyl)anthraquinone)

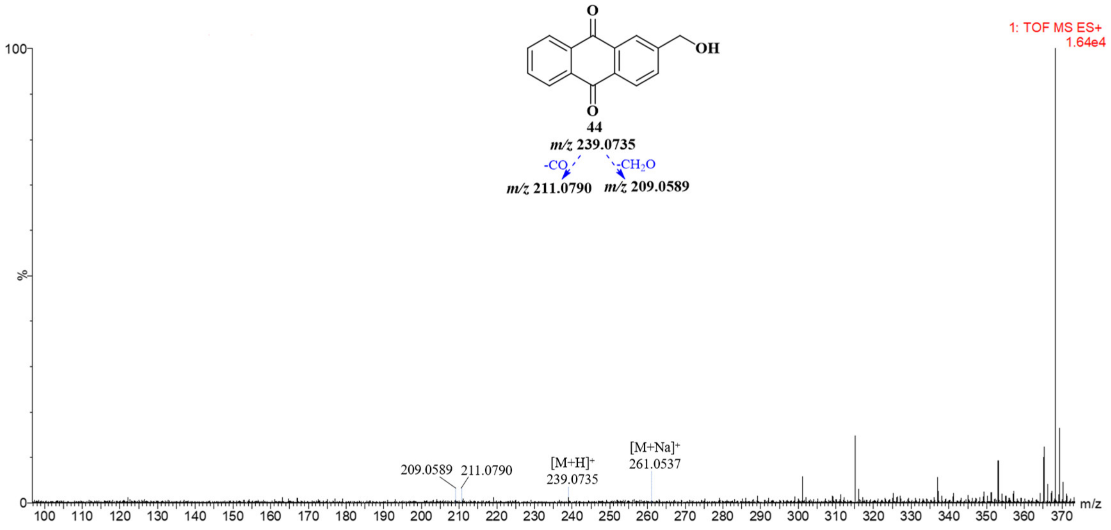

Peak **45** (t_R= 20.34min, $C_{24}H_{24}O_{10}$, Formononetin-7-*O*-Glc-6''-*O*-Ace)

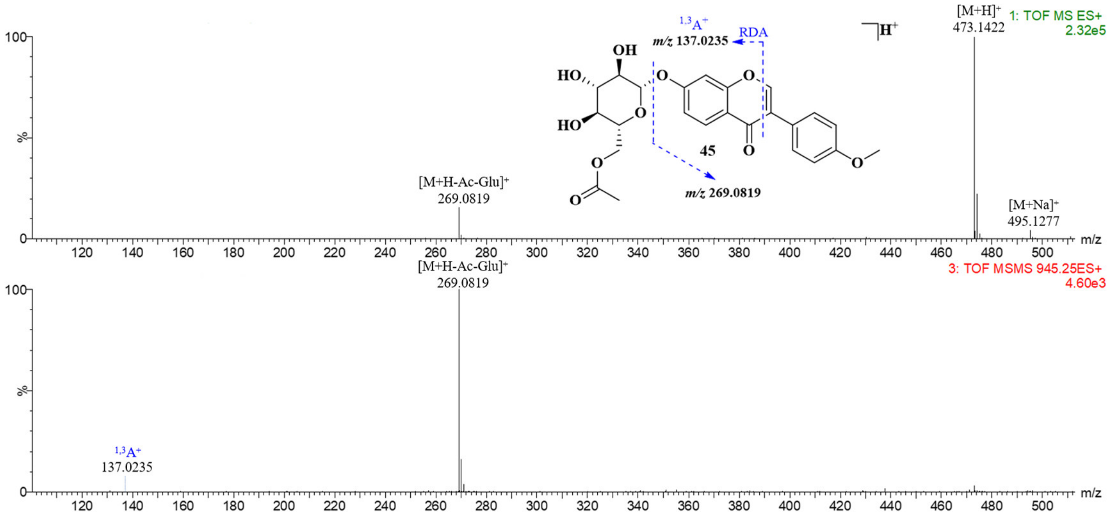

Peak **46** (t_R= 20.60min, $C_{17}H_{16}O_6$, 3,10-dihydroxy-7,9-dimethoxypterocarpan)

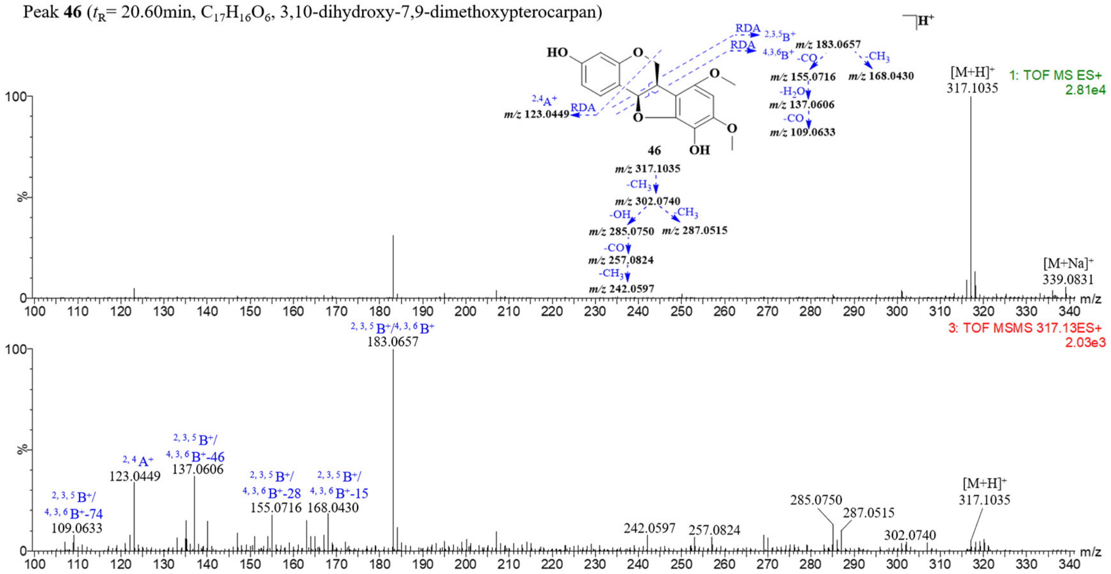

Peak **47** (t_R= 20.93min, $C_{18}H_{20}O_6$, Astragalinin A)

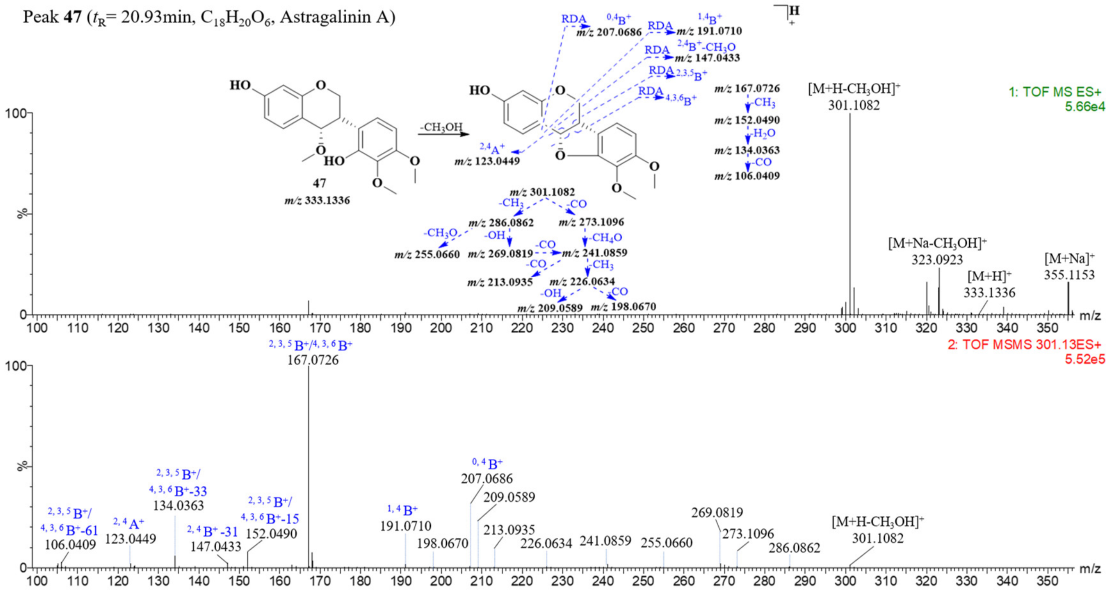

Peak **48** (t_R= 21.24min, $C_{25}H_{26}O_{11}$, 6″-*O*-Acetylwistin)

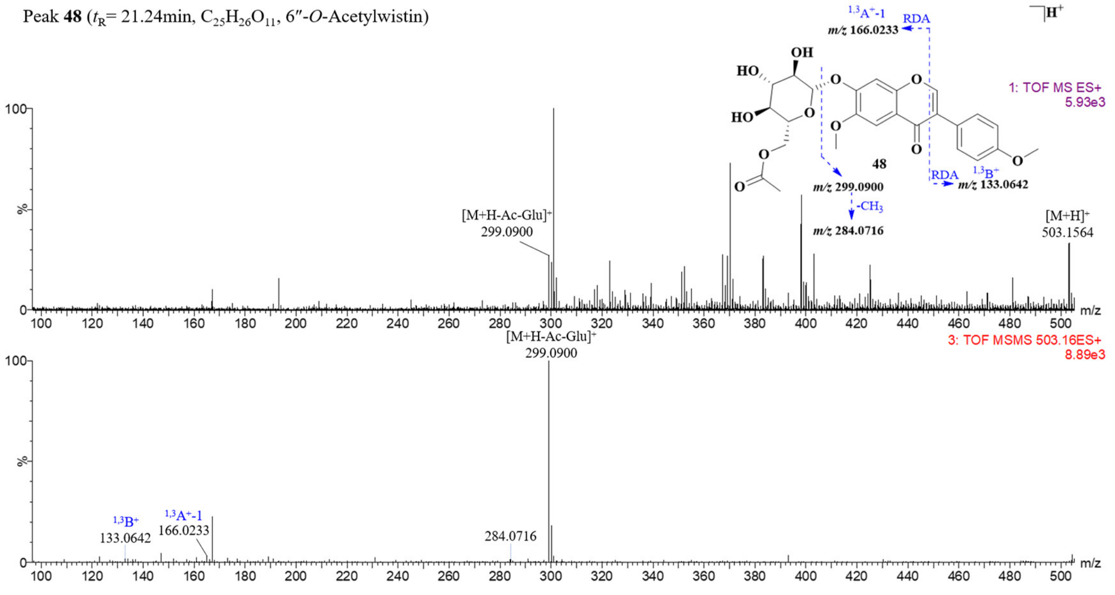

Peak **49** (t_R= 21.74min, $C_{25}H_{28}O_{11}$, (-)-Methylinissolin 3-*O*-*β*-D-(6'-acetyl) -glucoside)

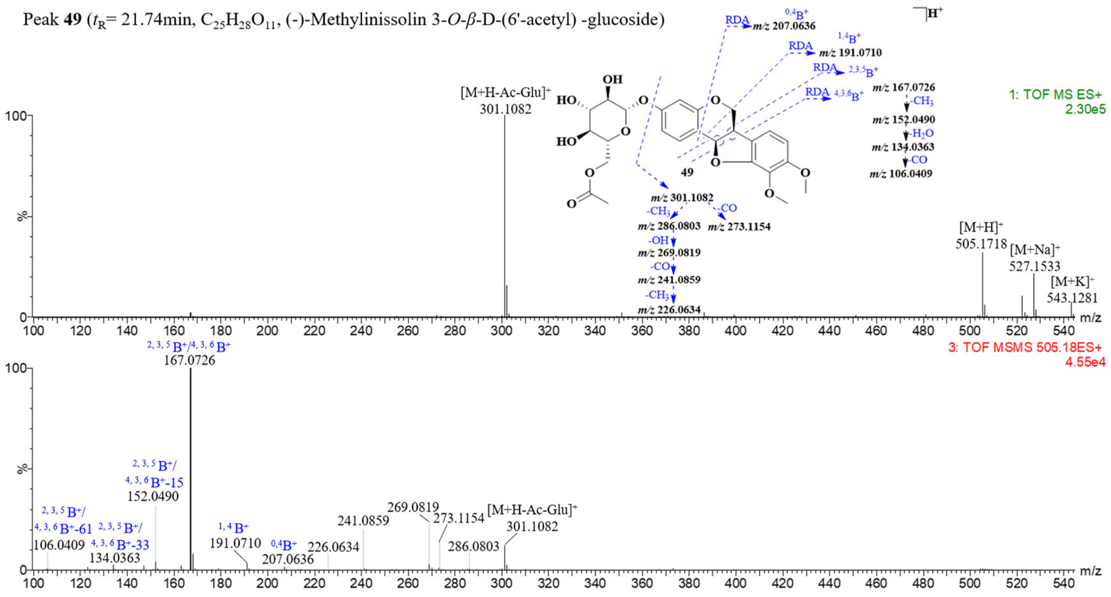

Peak **50** (t_R= 21.88min, $C_{15}H_{10}O_4$, 2-Hydroxy-1-methoxy-anthraquinone)

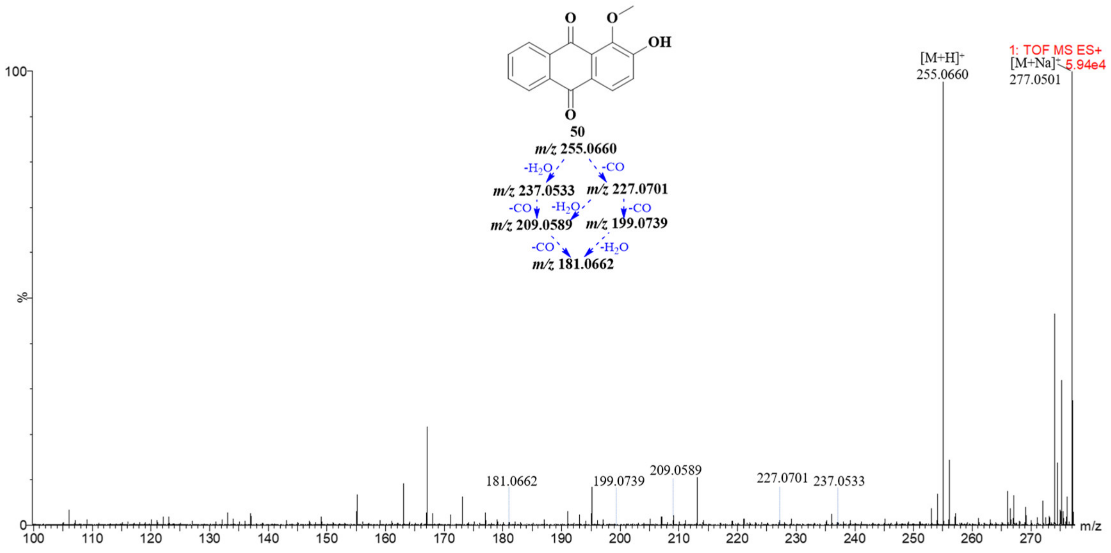

Peak **51** (t_R= 22.02min, $C_{18}H_{32}O_5$, 9,12,13-trihydroxy-10(*E*),15(*Z*)-octadecadienoic acid)

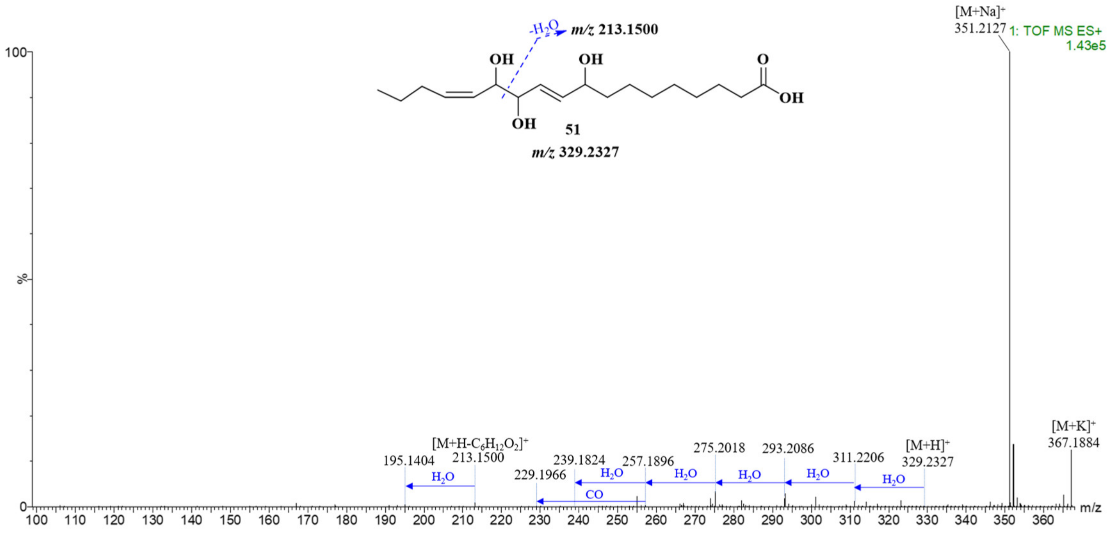

Peak **52** (t_R= 23.21min, $C_{25}H_{30}O_{11}$, 6''-*O*-Acetyl-(3*R*)-2'-hydroxy-3',4'-dimethoyl-isoflavan -7-*O*-*β*-D-glucopyranoside)

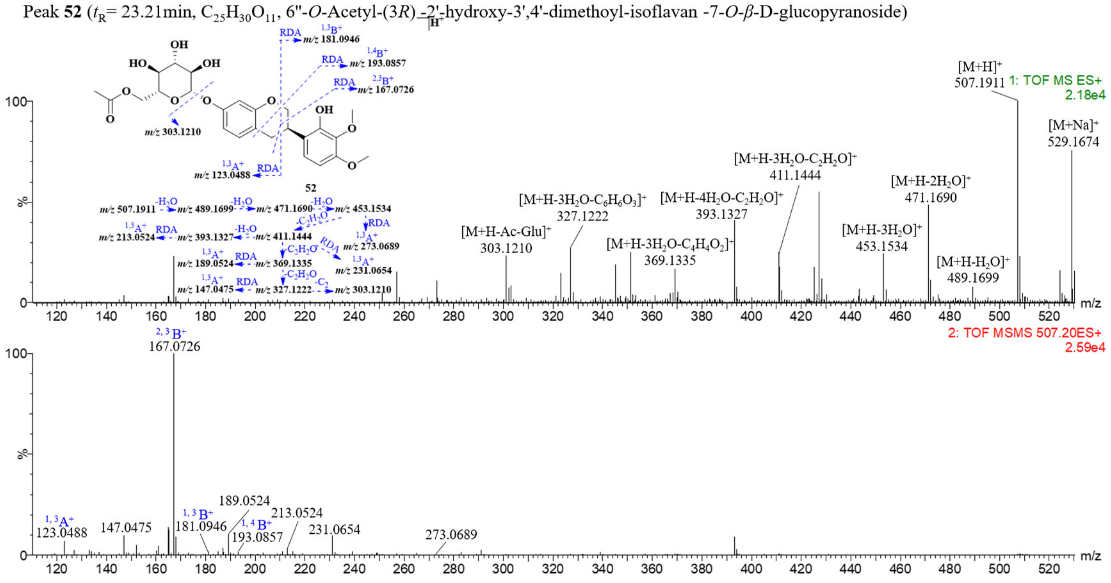

Peak **53** (t_R= 23.51min, $C_{16}H_{12}O_4$, Formononetin)

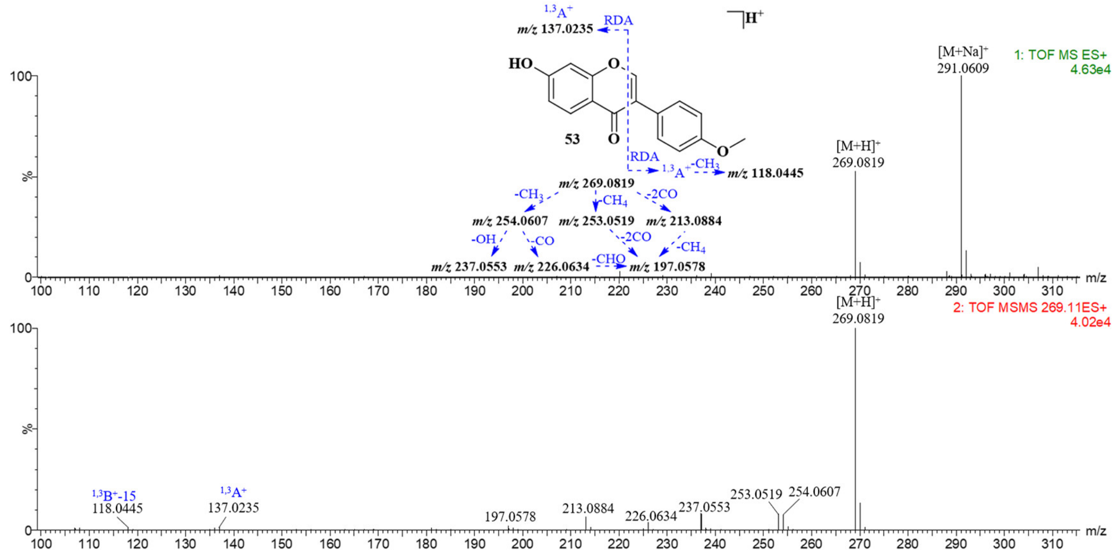

Peak **54** (t_R= 23.72min, $C_{18}H_{20}O_6$, Astragalinin B)

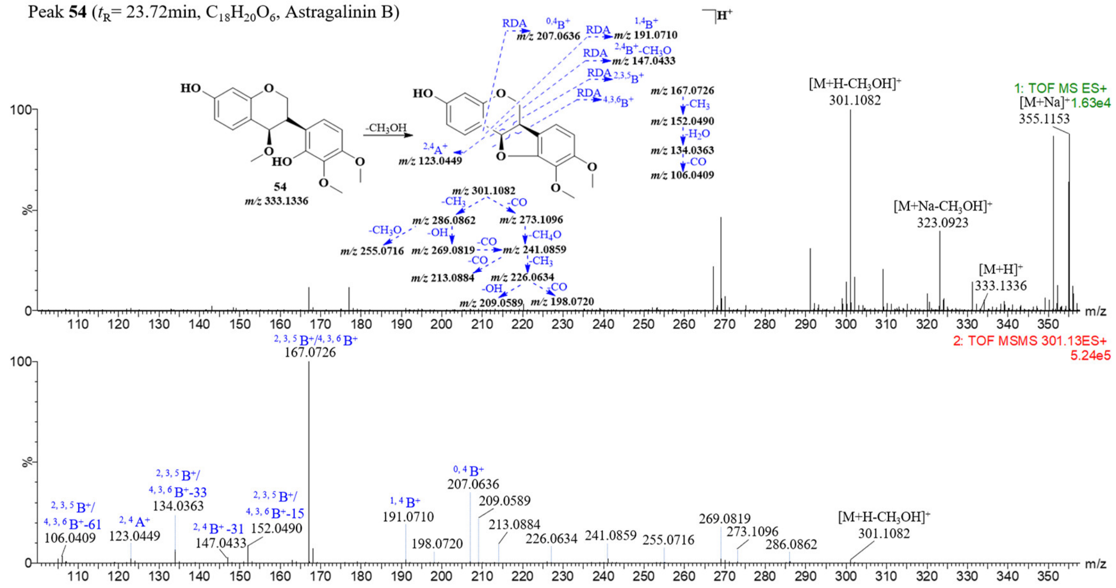

Peak **55** (t_R= 23.95min, $C_{18}H_{17}O_6$, Micanspterocarpan)

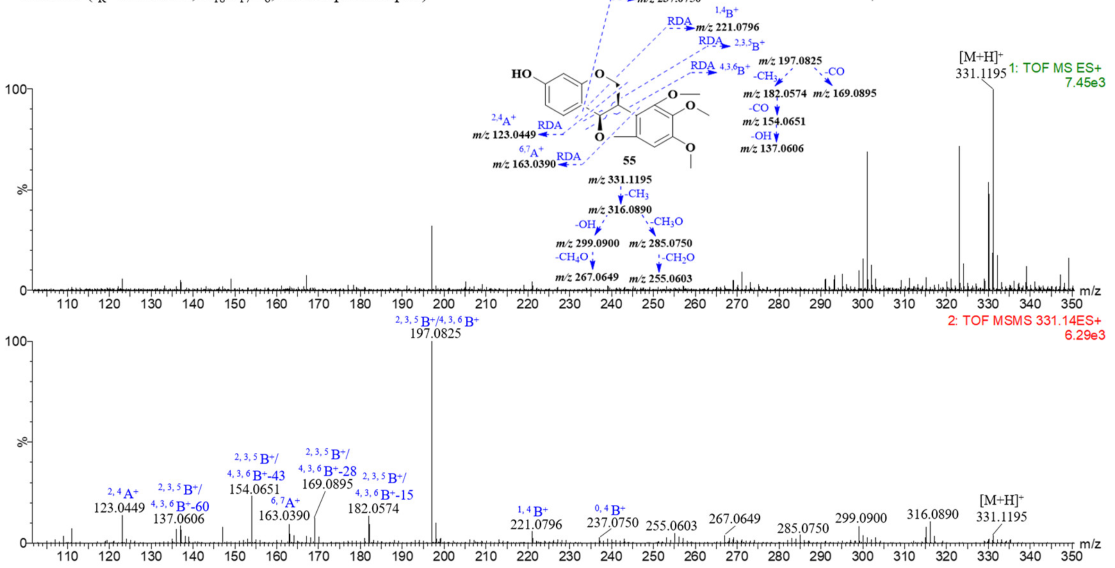

Peak **56** (t_R= 24.02min, $C_{20}H_{20}O_7$, Isosinensetin)

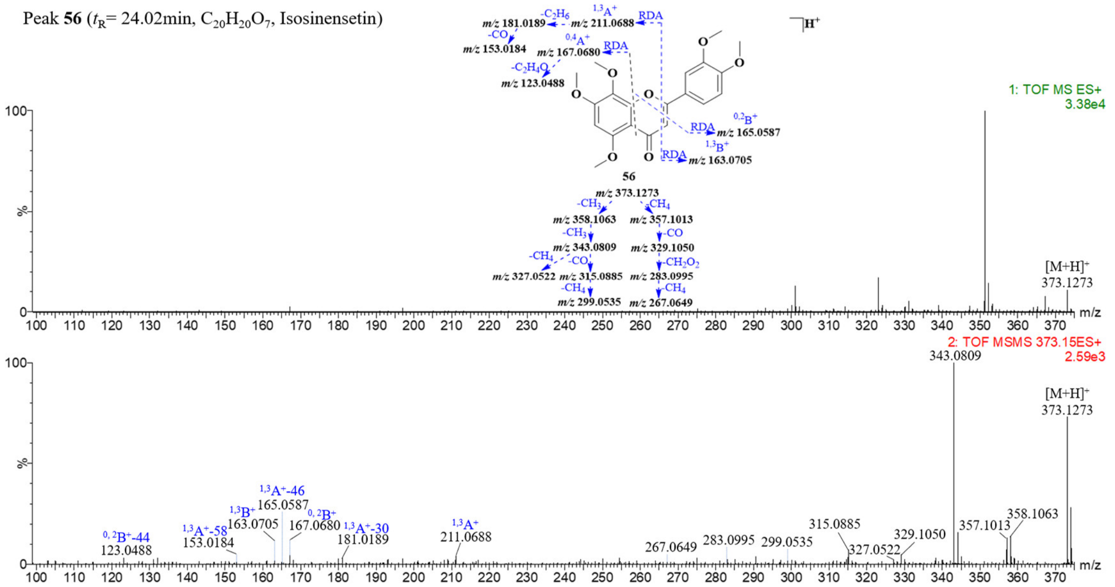

Peak **57** (t_R= 24.24min, $C_{17}H_{16}O_5$, (-)-methylnissolin)

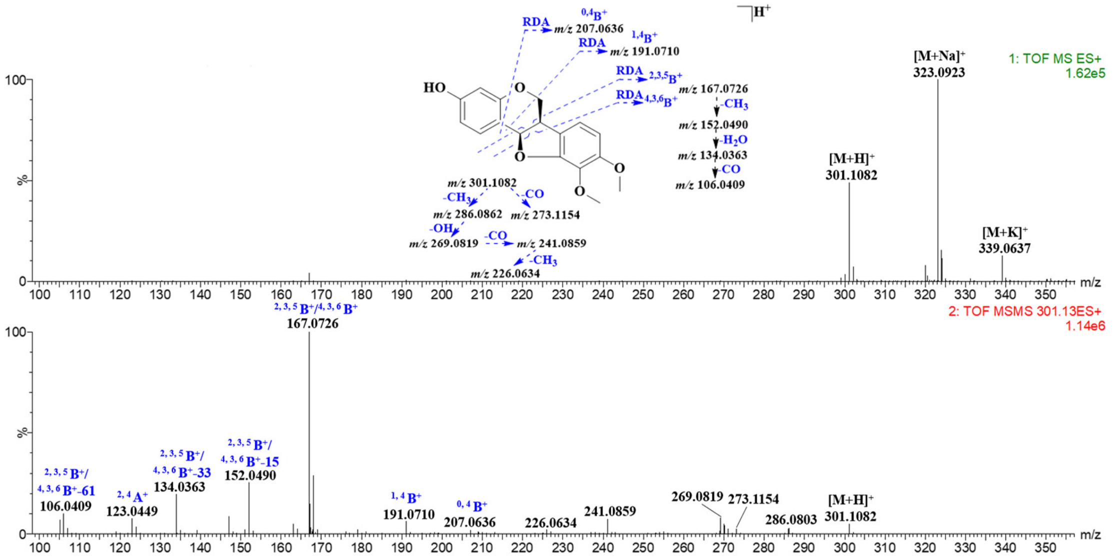

Peak **58** (t_R= 24.77min, $C_{18}H_{34}O_5$, 9,12,13-trihydroxy-10(*E*)-octadecadienoic acid)

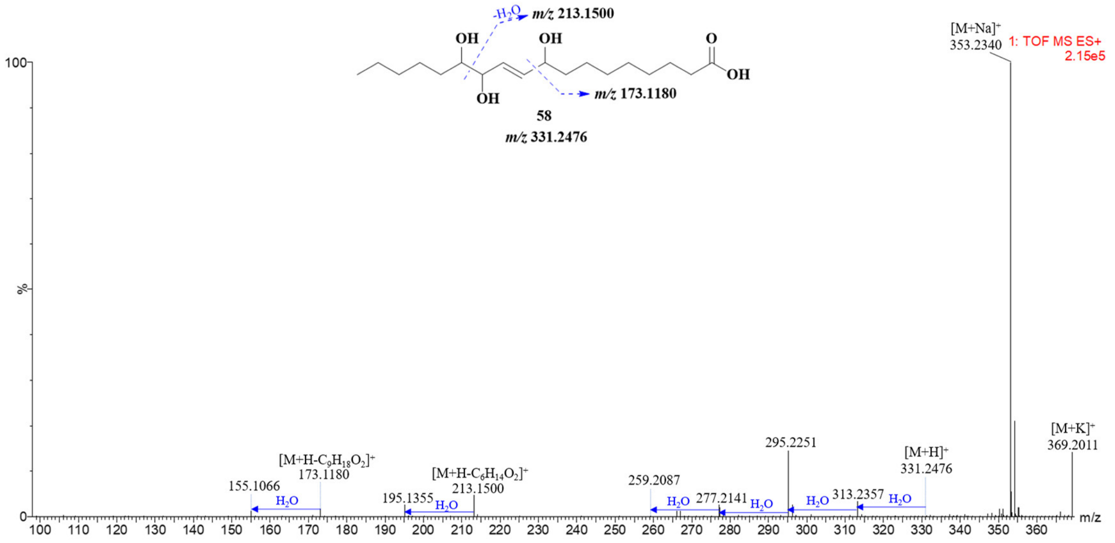

Peak **59** (t_R= 24.97min, $C_{17}H_{14}O_5$, Afrormosin)

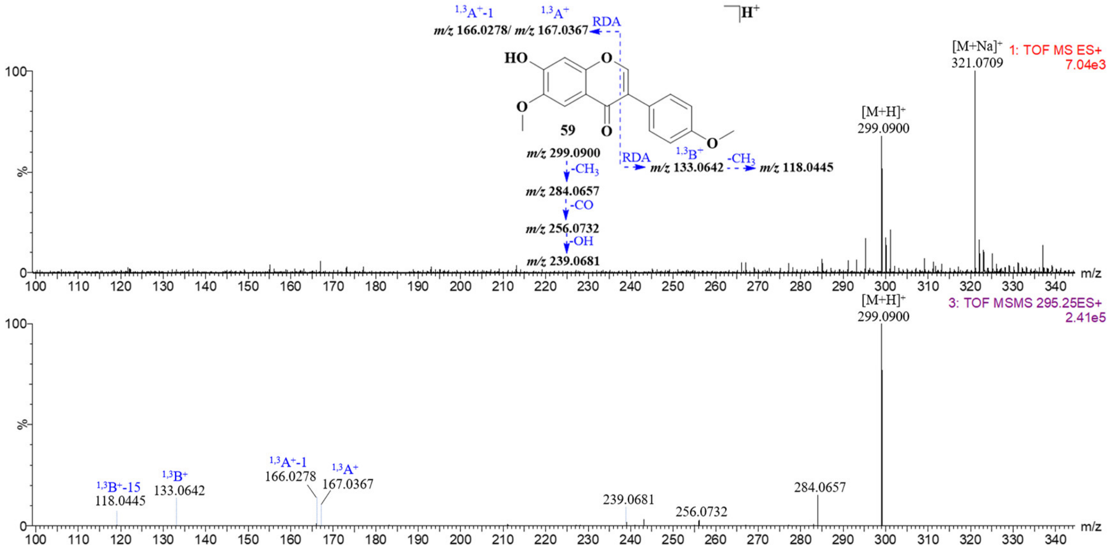

Peak **60** (t_R= 25.08min, $C_{18}H_{34}O_5$, 9,12,13-trihydroxy-10(*E*)-octadecadienoic acid isomer)

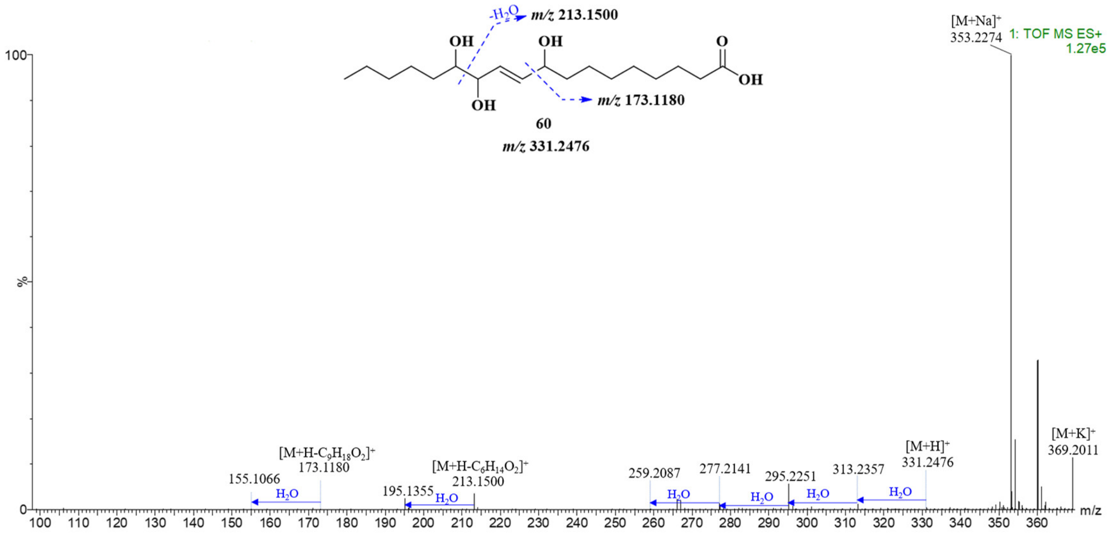

Peak **61** (t_R= 25.93min, $C_{17}H_{18}O_5$, Isomucronulatol)

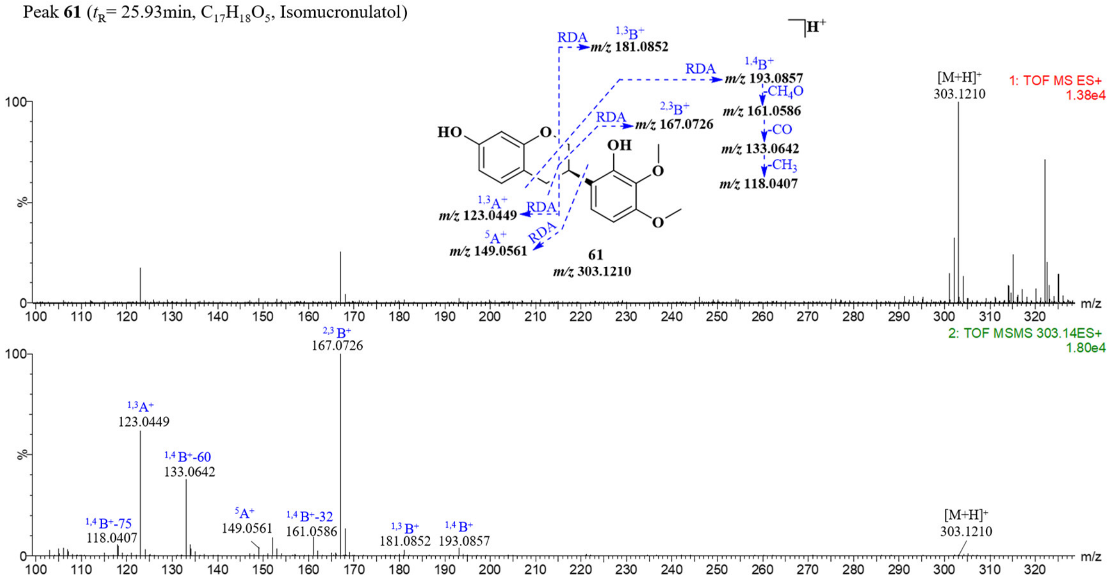

Peak **62** (t_R= 26.03min, $C_{26}H_{26}O_{10}$, Ammopiptanoside A)

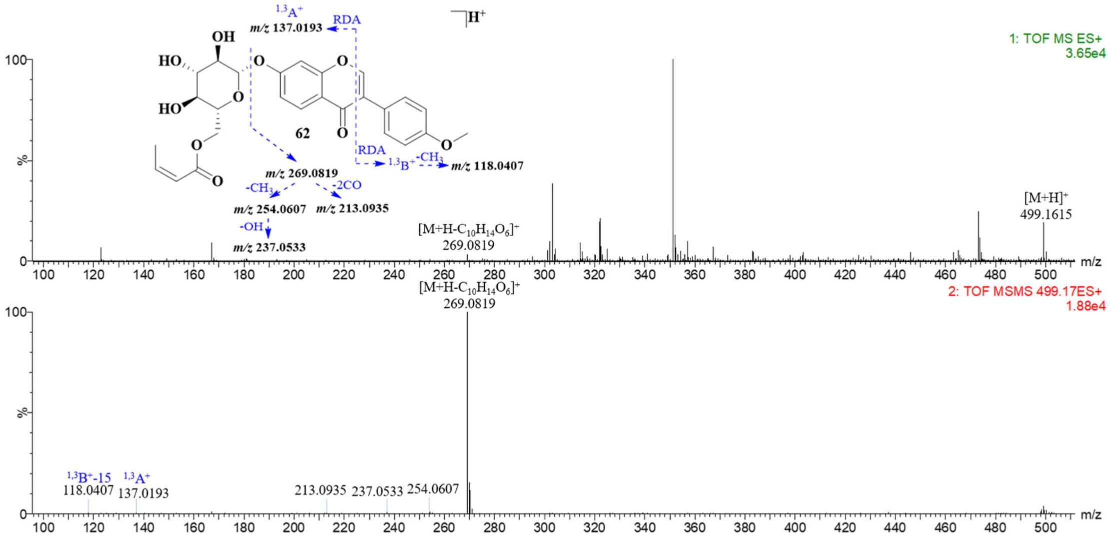

Peak **62** (t_R= 26.03min, $C_{26}H_{26}O_{10}$, Ammopiptanoside A)

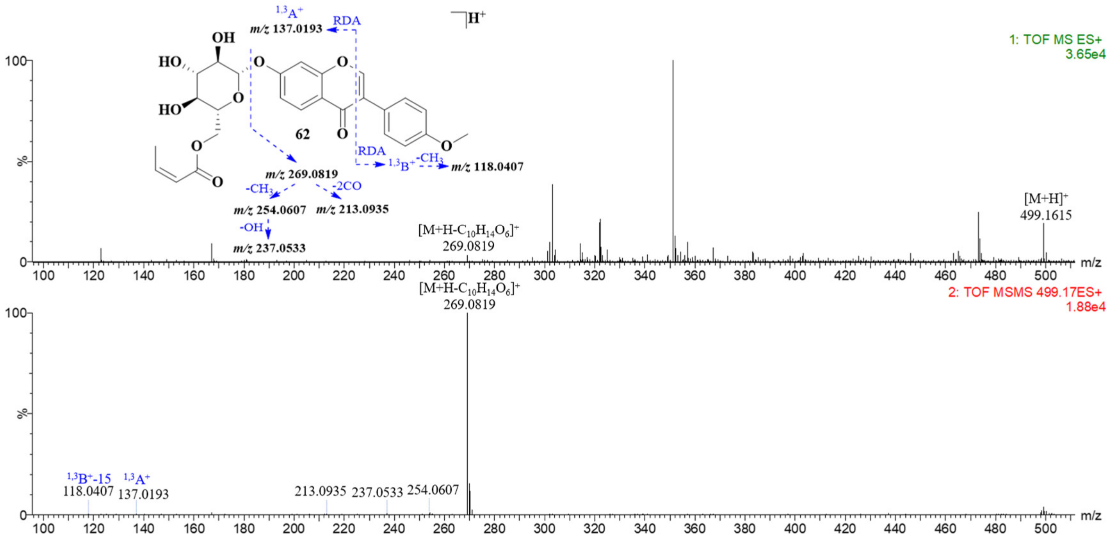

Peak **63** (t_R= 26.93min, $C_{20}H_{20}O_7$, Sinensetin)

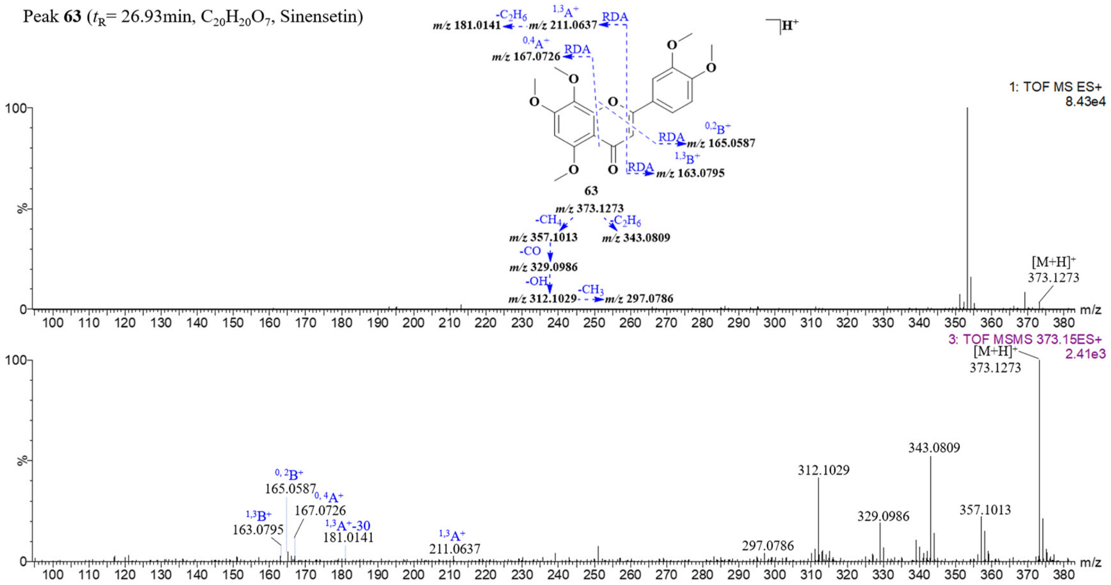

Peak **64** (t_R= 27.04min, $C_{19}H_{18}O_6$, 6-Demethoxytangeretin)

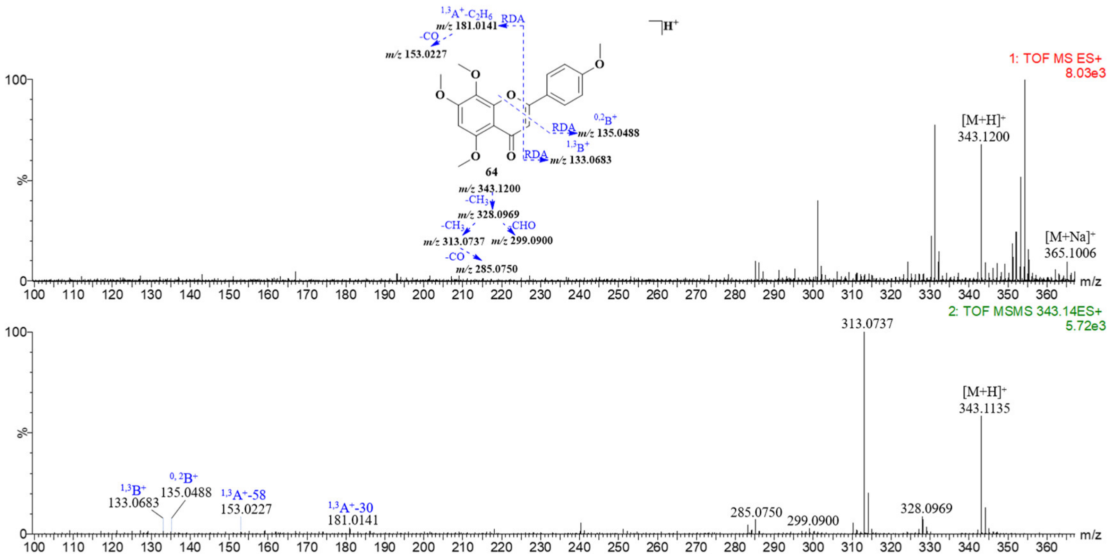

Peak **65** (t_R= 28.29min, $C_{43}H_{70}O_{15}$, Astralanosaponin H)

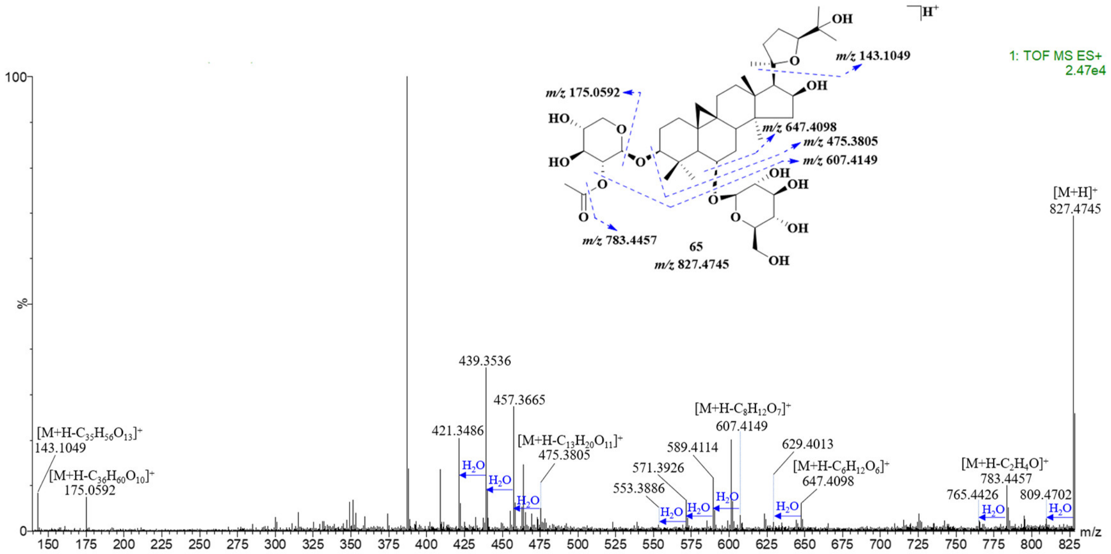

Peak **66** (t_R= 28.71min, $C_{43}H_{70}O_{15}$, Astralanosaponin H isomer)

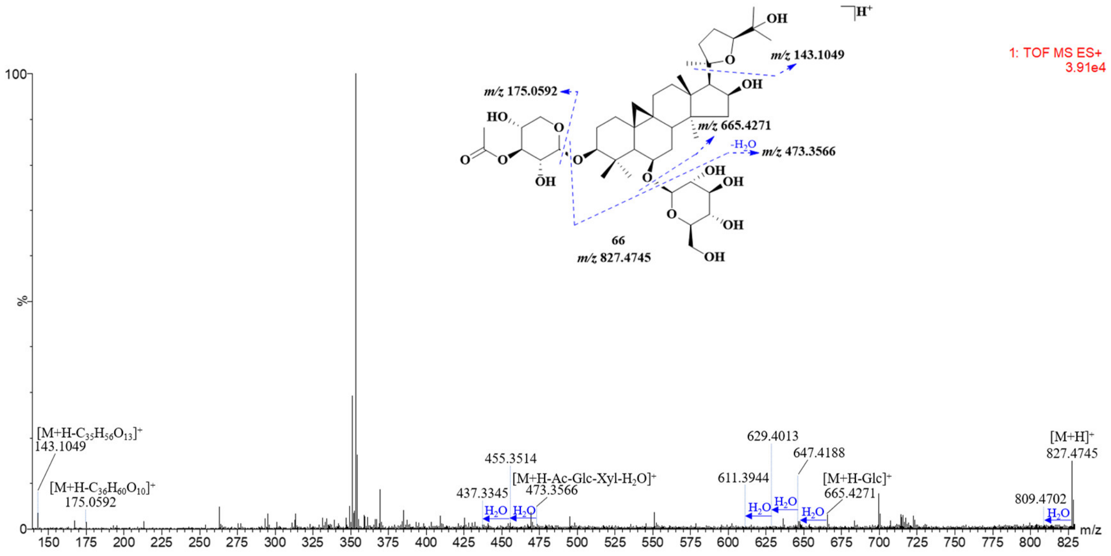

Peak **67** (t_R= 28.90min, $C_{41}H_{68}O_{14}$, Astragaloside IV)

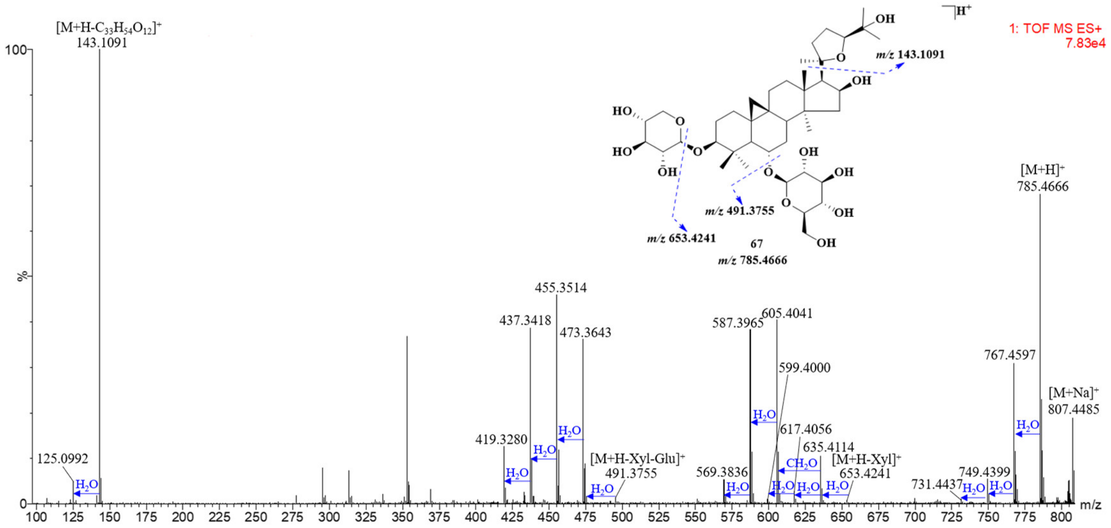

Peak **68** (t_R= 29.23min, $C_{41}H_{68}O_{14}$, Astragaloside III)

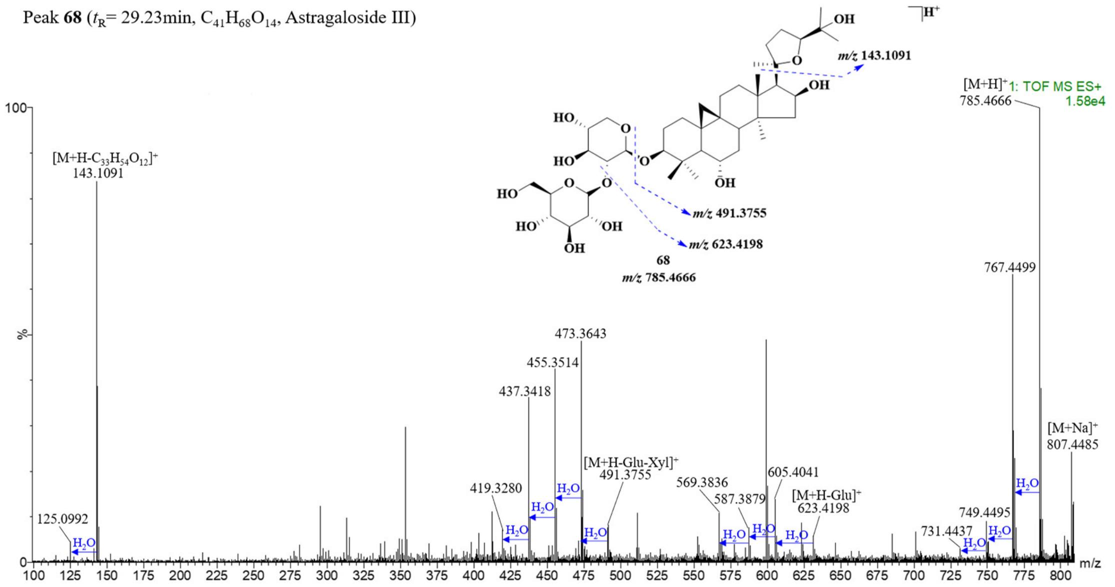

Peak **69** (t_R= 29.29min, $C_{17}H_{12}O_4$, Me anthraquinone-1-acetate)

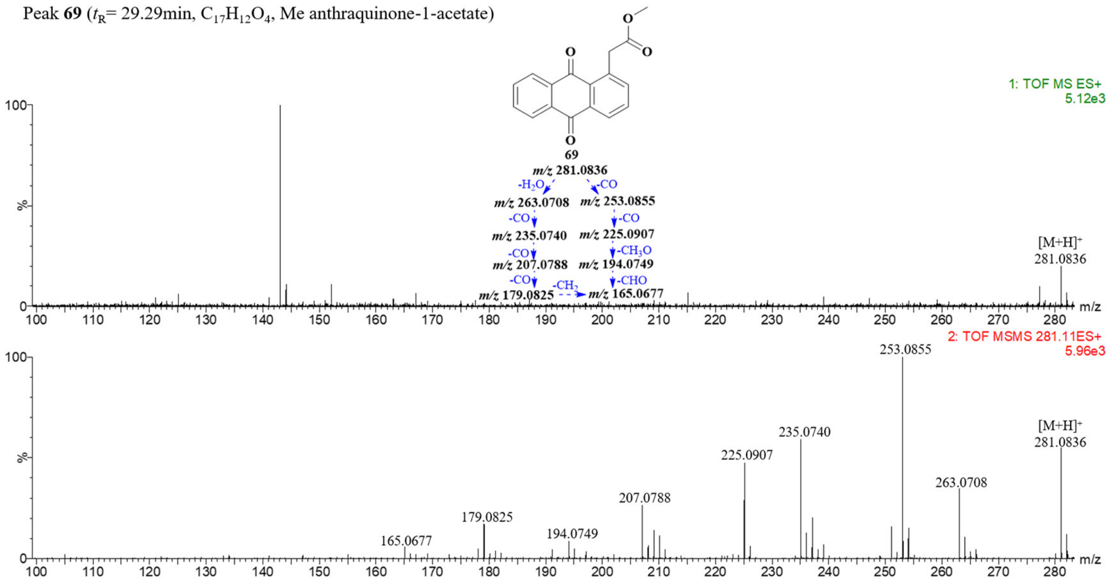

Peak **70** (t_R= 29.52min, $C_{18}H_{20}O_6$, Astragalinin isomer)

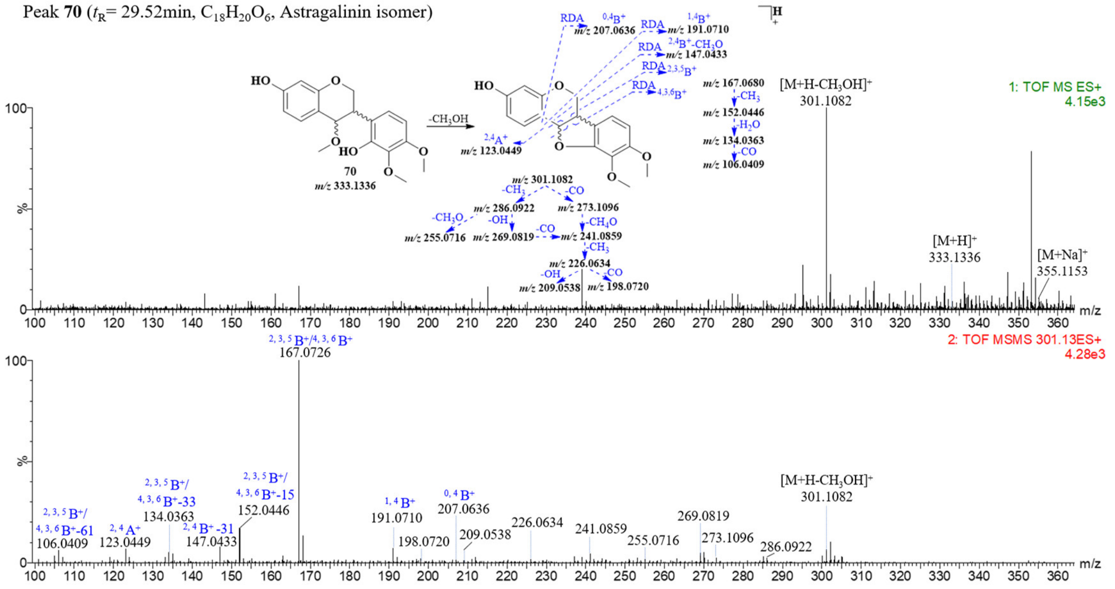

Peak **71** (t_R= 29.67min, $C_{15}H_{10}O_5$, 2-Hydroxy-3-methylanthraquine)

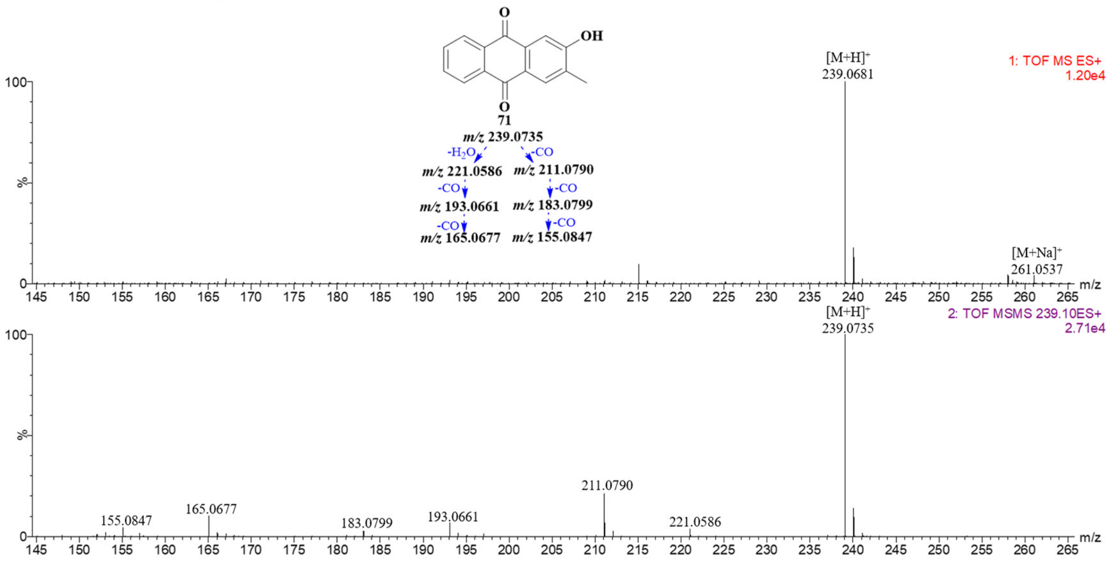

Peak **72** (t_R= 30.36min, $C_{19}H_{18}O_6$, Tetramethylscutellarein)

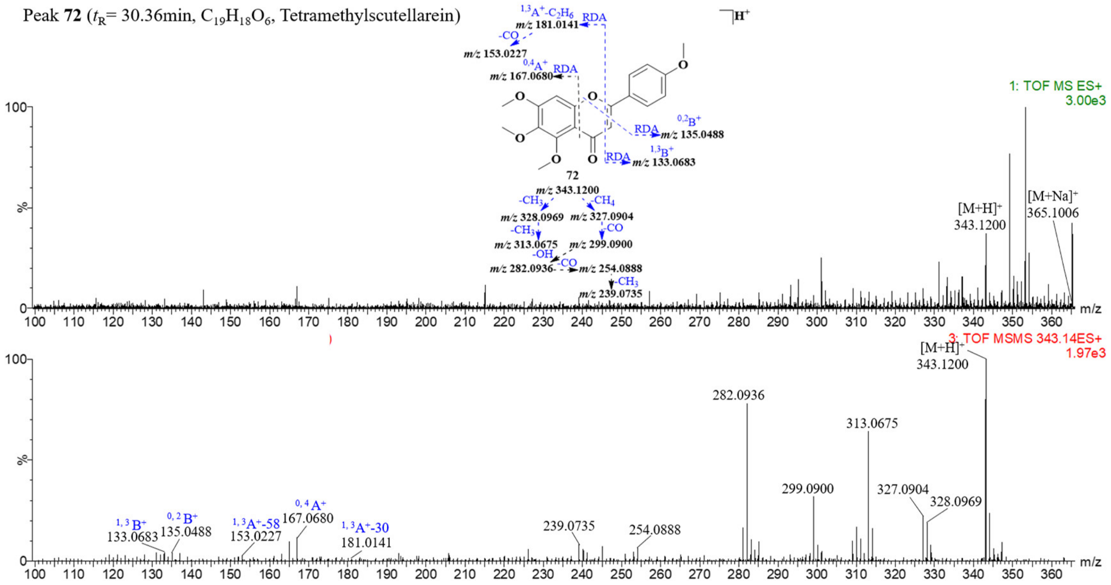

Peak **73** (t_R= 30.53min, $C_{21}H_{22}O_8$, Nobiletin)

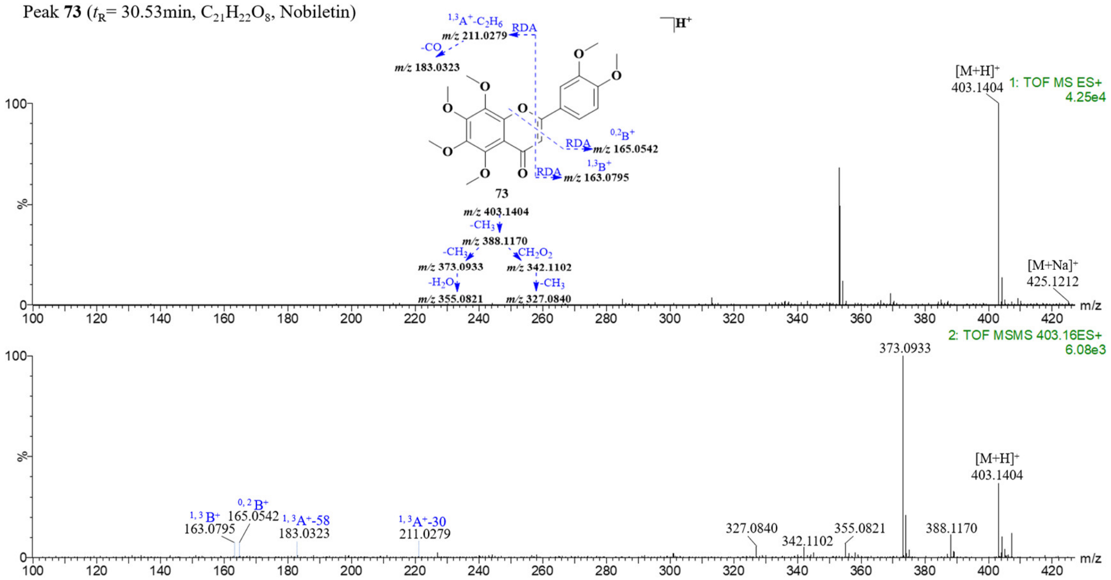

Peak **74** (t_R= 31.97min, $C_{36}H_{60}O_{10}$, Cycloaraloside A)

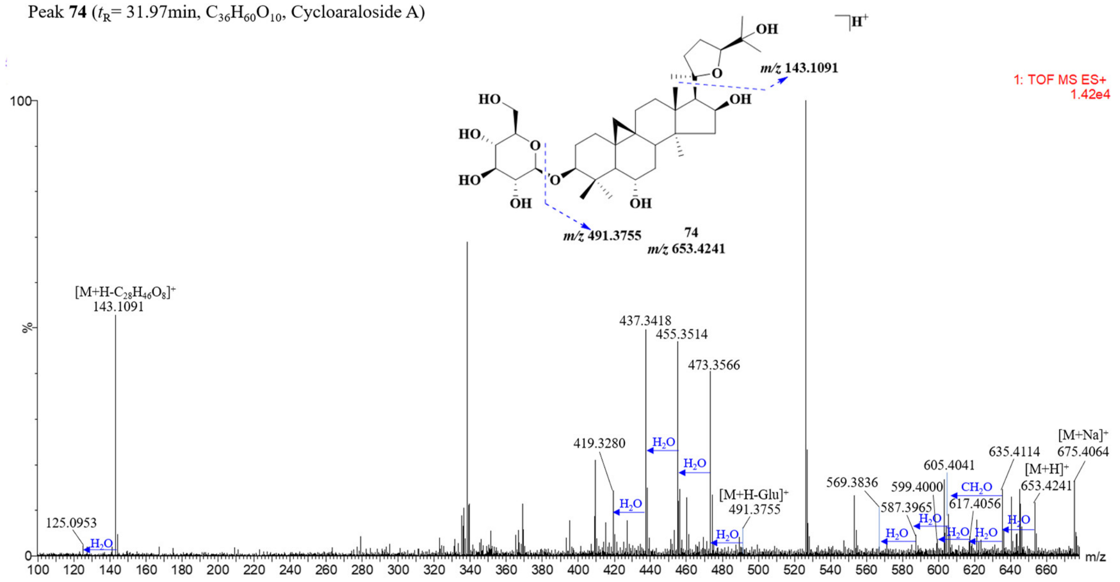

Peak **75** (t_R= 32.09min, $C_{35}H_{58}O_9$, Astramembrannin II)

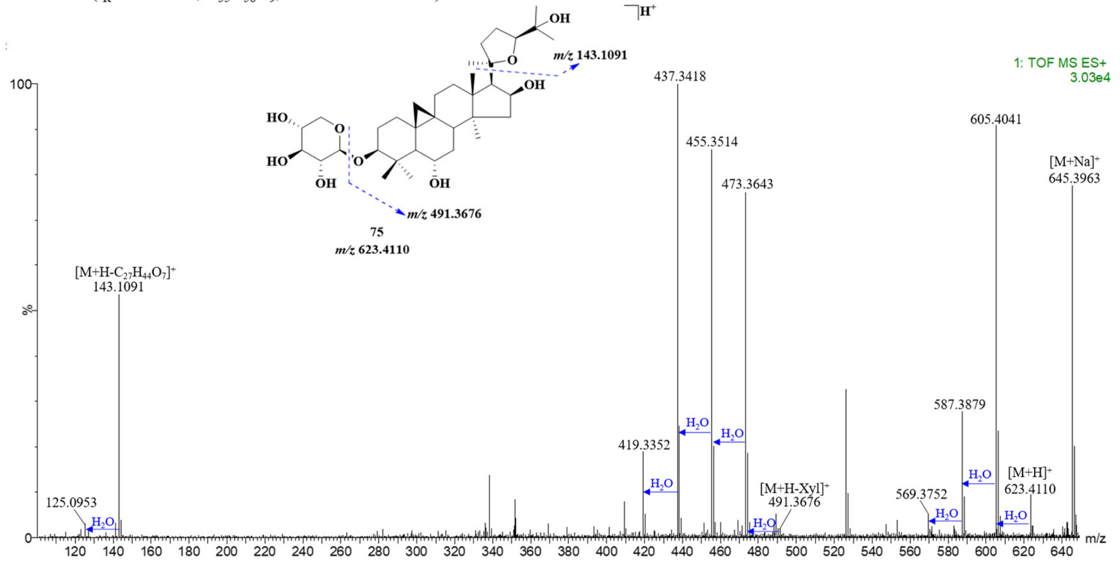

Peak **76** (t_R= 32.47min, $C_{43}H_{70}O_{15}$, Astragaloside II)

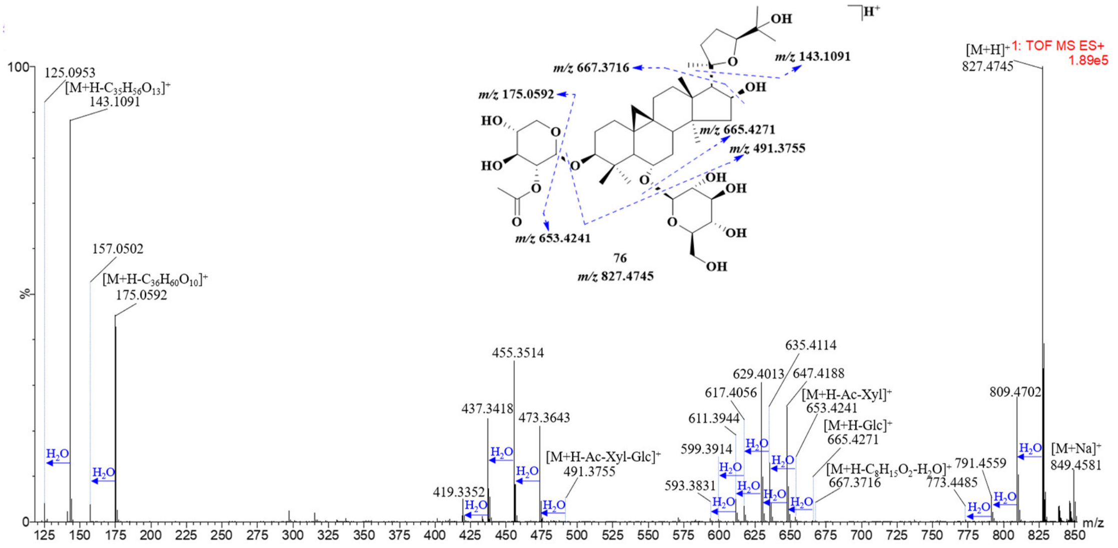

Peak **77** (t_R= 32.88min, $C_{22}H_{24}O_9$, 3, 5, 6, 7, 8, 3′, 4′-Heptamethoxyflavone)

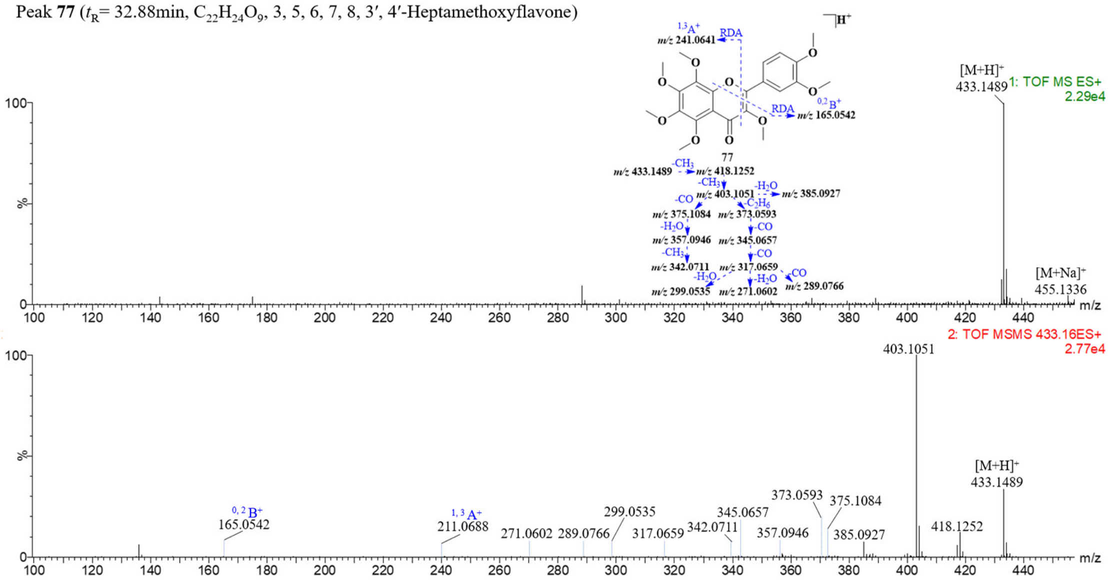

Peak **78** (t_R= 33.05min, $C_{43}H_{70}O_{15}$, Astralanosaponin I)

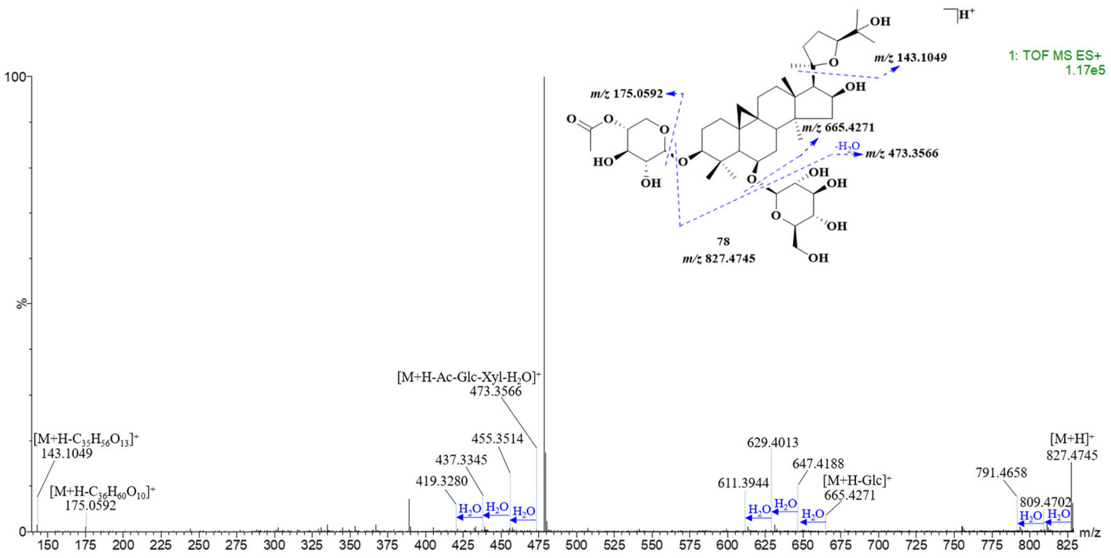

Peak **79** (t_R= 33.92min, $C_{18}H_{30}O_4$, Octadecatrienoic acid isomer)

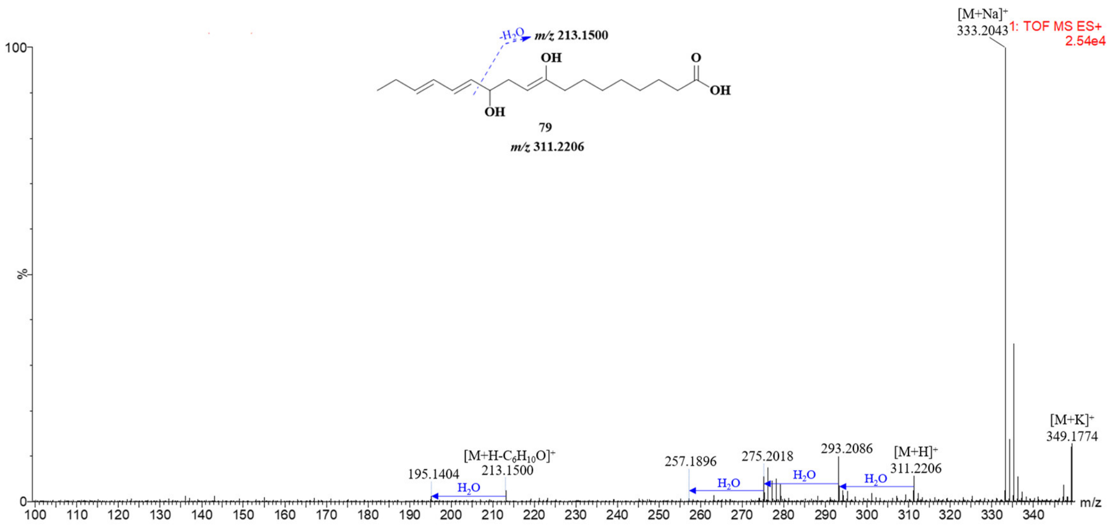

Peak **80** (t_R= 33.96min, $C_{20}H_{20}O_7$, Tangeretin)

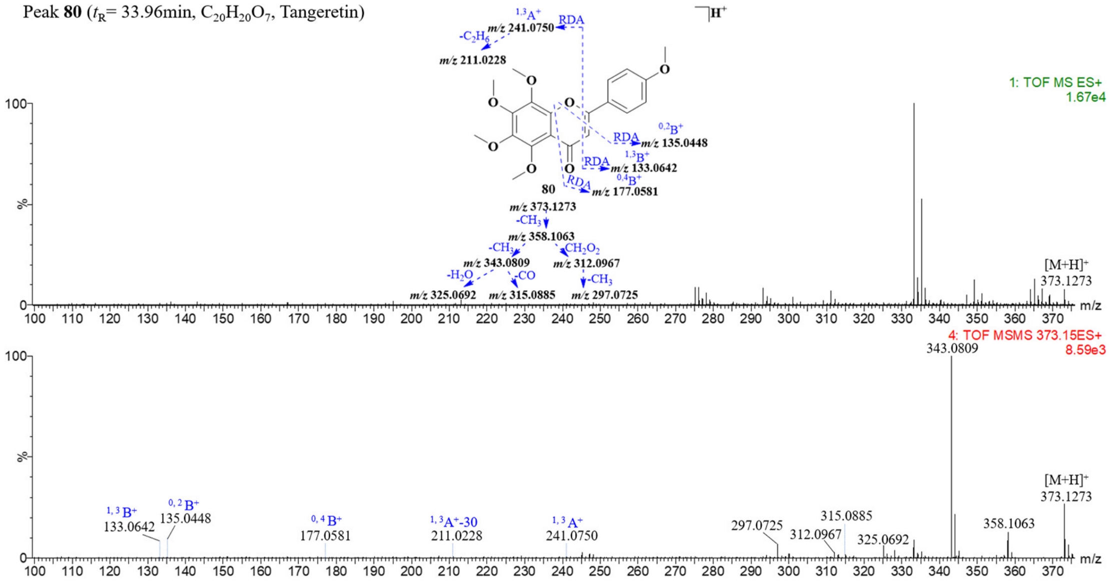

Peak **81** (t_R= 34.16min, $C_{21}H_{22}O_9$, 5-Hydroxy-3, 6, 7, 8, 3′, 4′-hexamethoxyflavone)

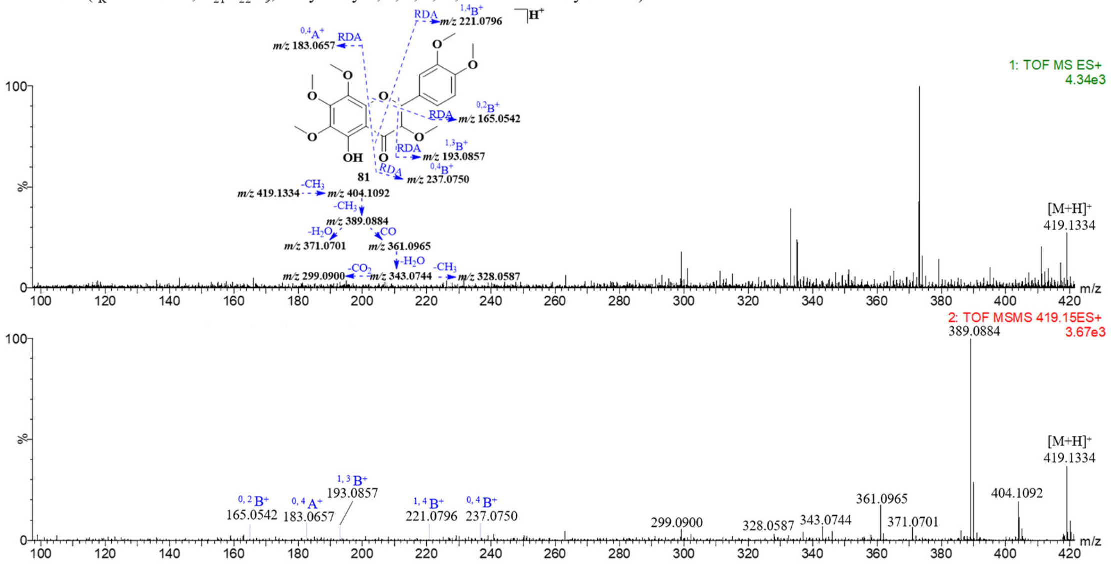

Peak **82** (t_R= 34.80min, $C_{43}H_{70}O_{15}$, Isoastragaloside II)

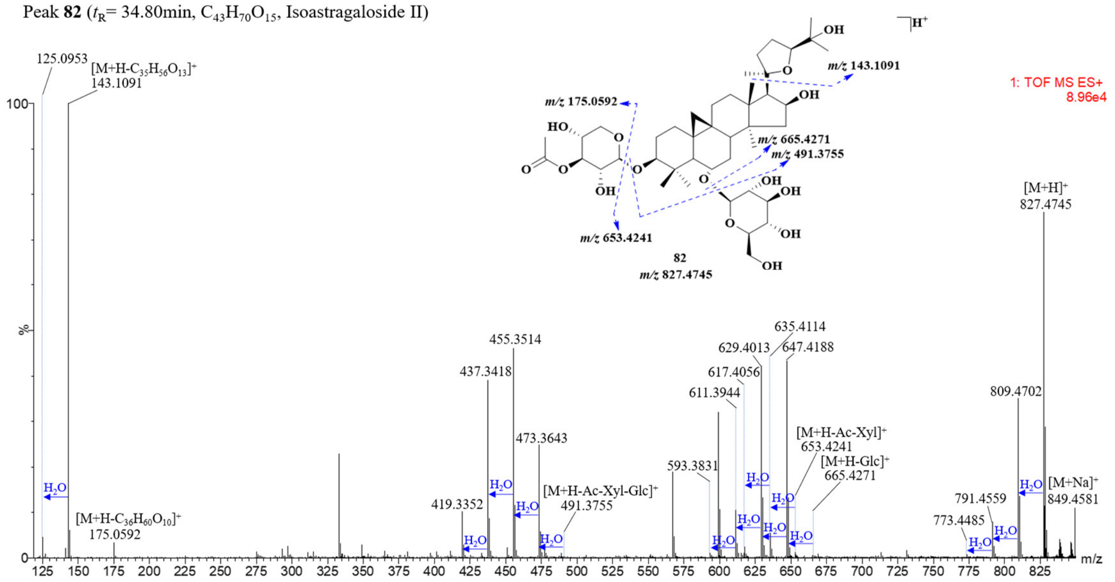

Peak **83** (t_R= 35.25min, $C_{18}H_{32}O_4$, 12,13-Dihydroxy-9,15-octadecadienoic acid)

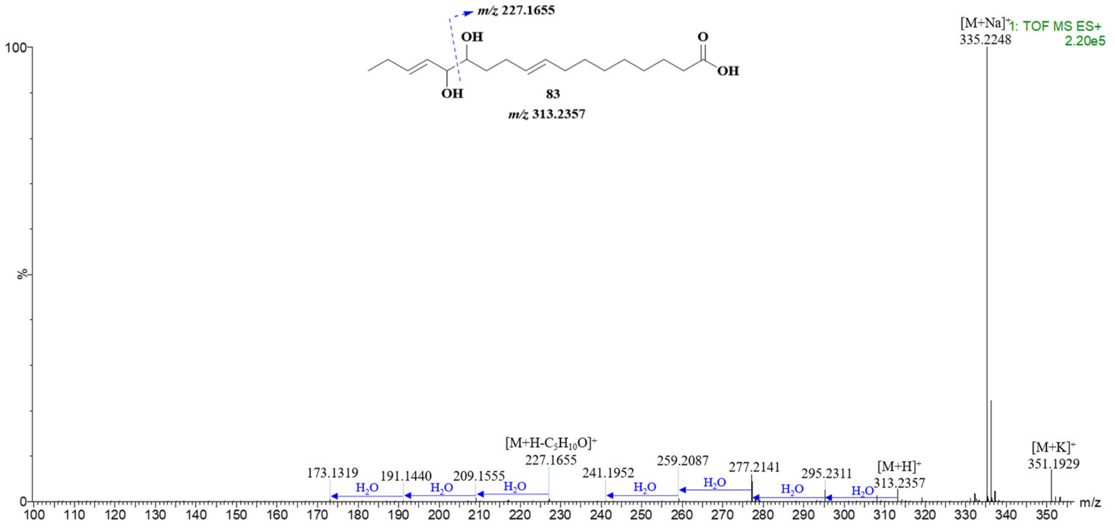

Peak **84** (t_R= 36.66min, $C_{43}H_{70}O_{15}$, Cyclocephaloside II)

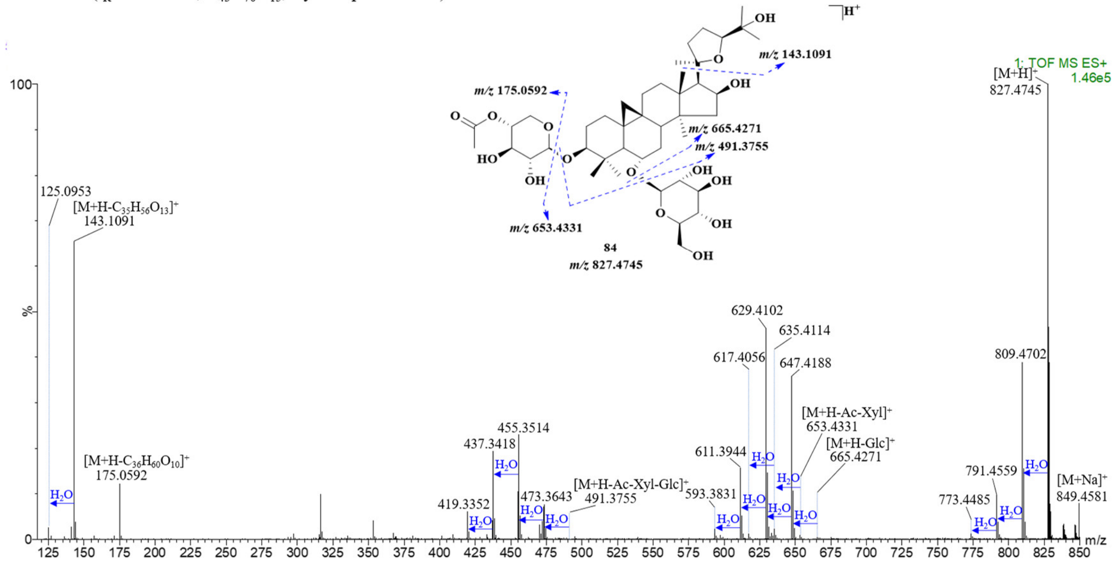

Peak **85** (t_R= 36.96min, $C_{45}H_{72}O_{16}$, Cycloglobiceposide A)

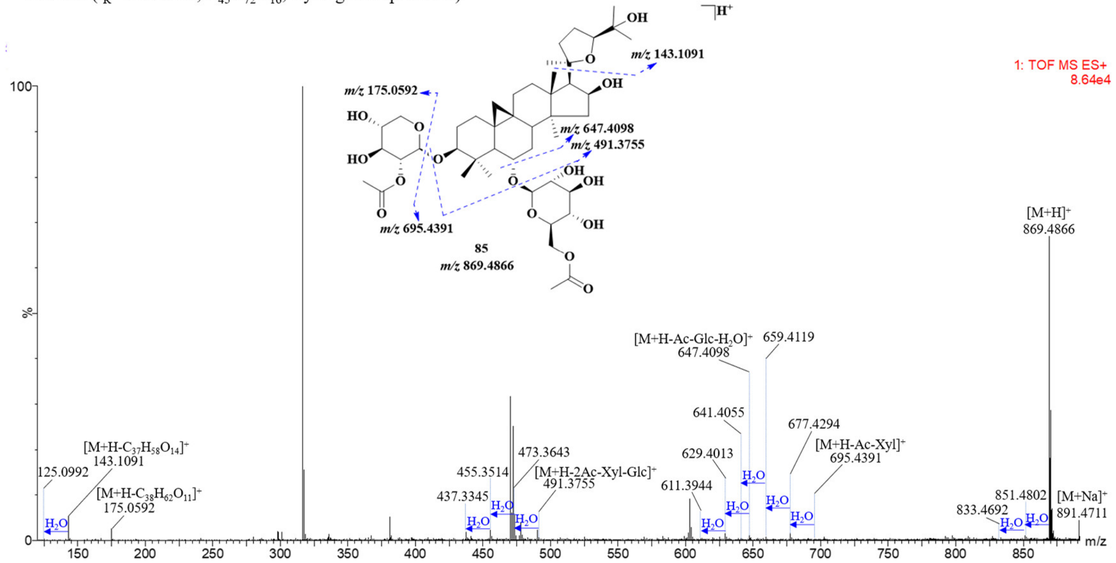

Peak **86** (t_R= 37.08min, $C_{37}H_{60}O_{10}$, Cyclogaleginoside A)

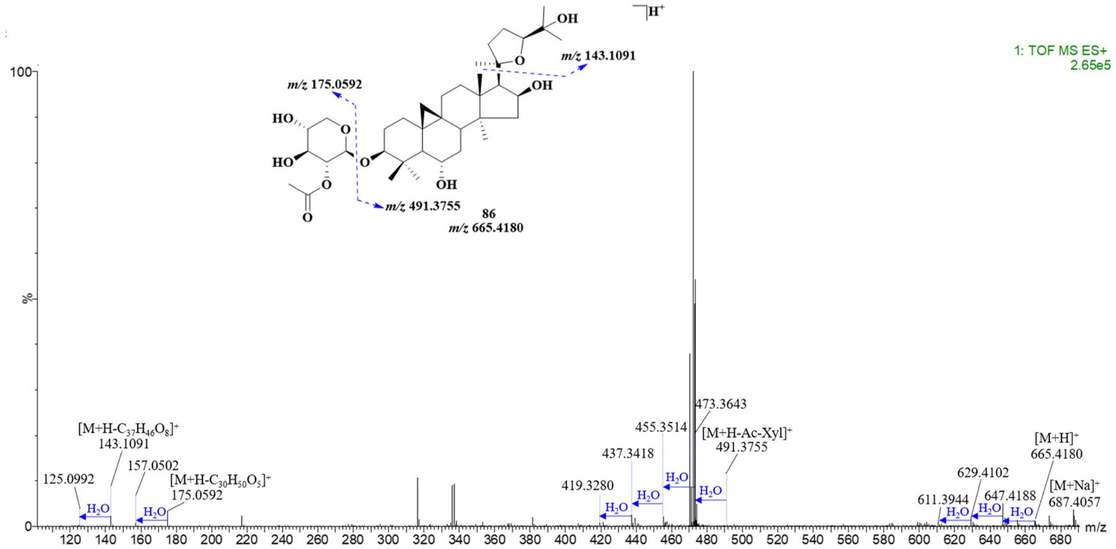

Peak **87** (t_R= 37.99min, $C_{20}H_{20}O_5$, Astragquinone)

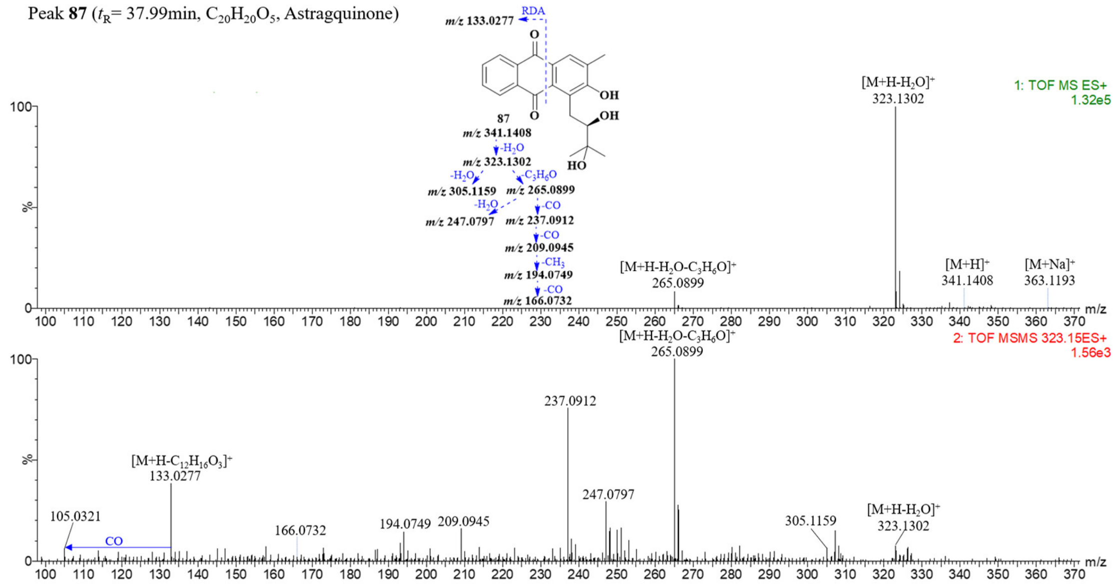

Peak **88** (t_R= 38.05min, $C_{30}H_{50}O_5$, Cycloastragenol)

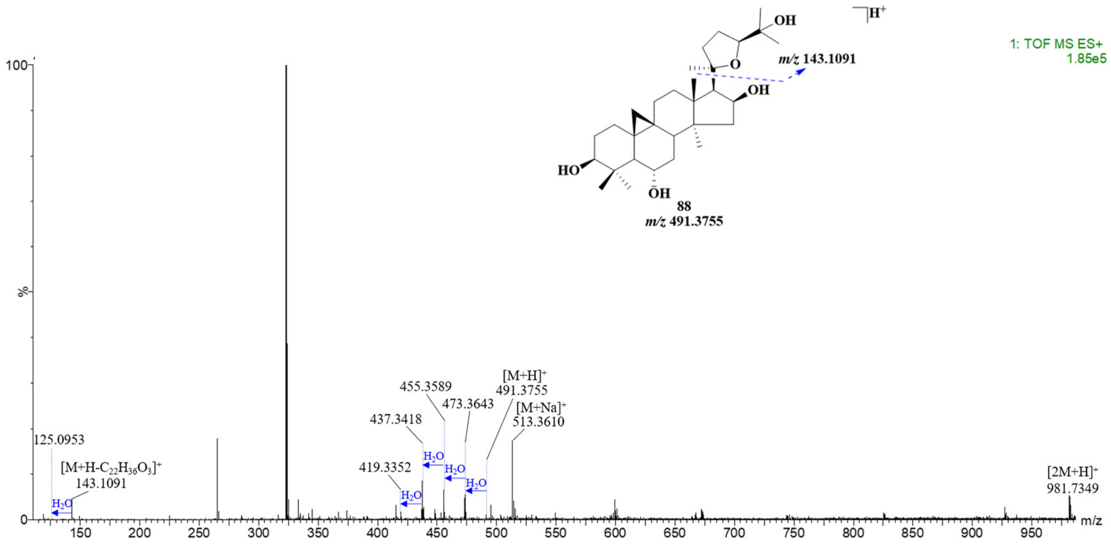

Peak **89** (t_R= 38.34min, $C_{45}H_{72}O_{16}$, Astragaloside I)

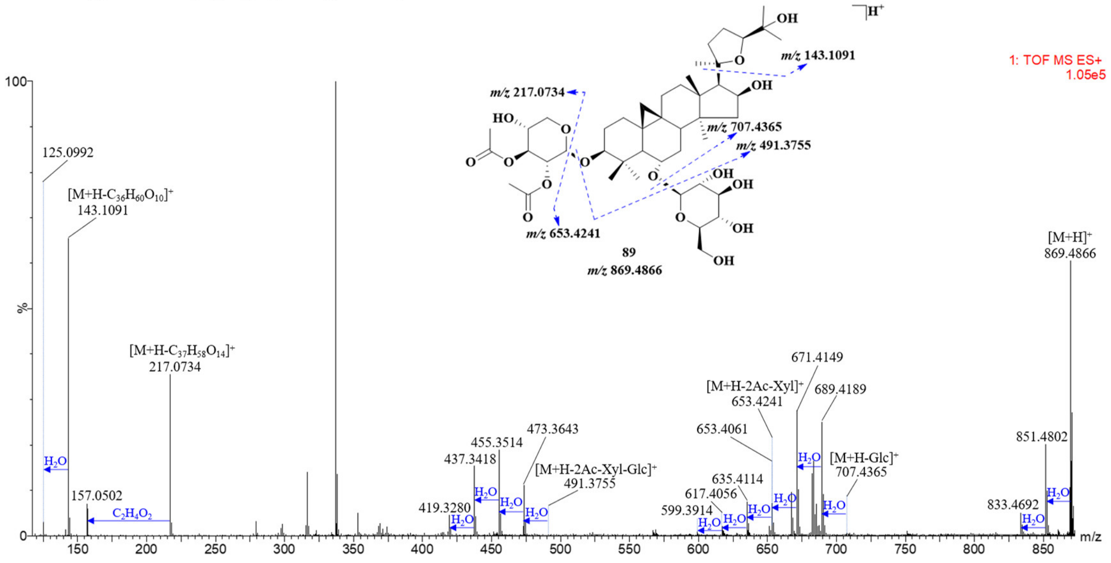

Peak **90** (t_R= 39.52min, $C_{45}H_{72}O_{16}$, Isoastragaloside I)

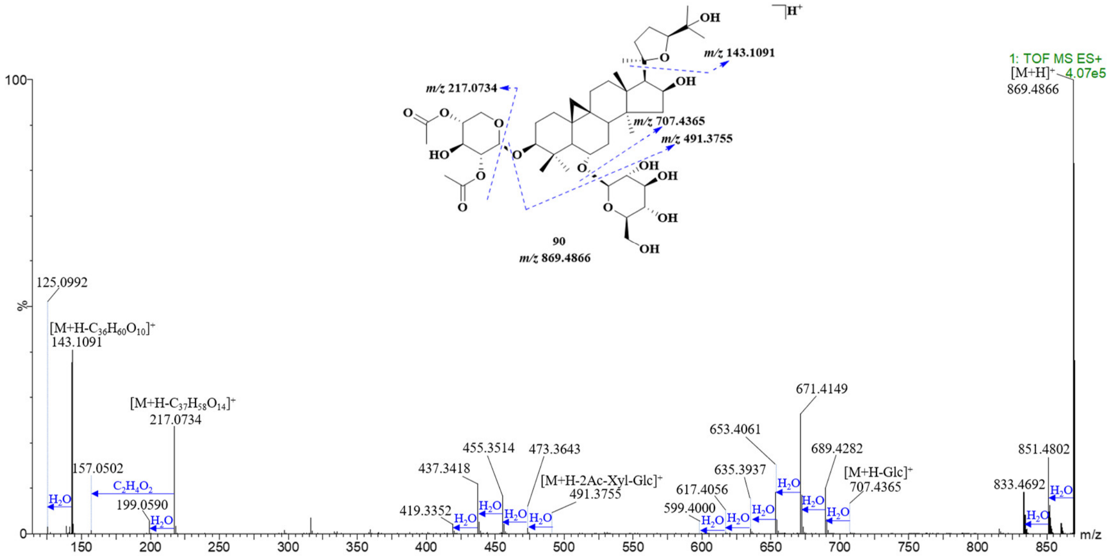

Peak **91** (t_R= 40.83min, $C_{45}H_{72}O_{16}$, Neoastragaloside I)

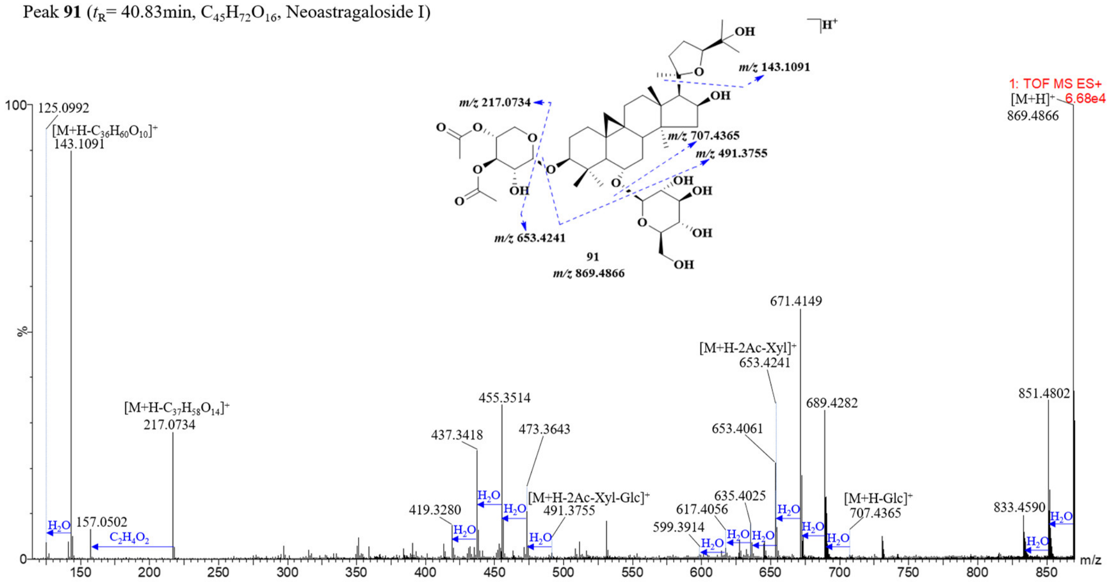

Peak **92** (t_R= 40.93min, $C_{47}H_{74}O_{17}$, Acetylastragaloside I isomer)

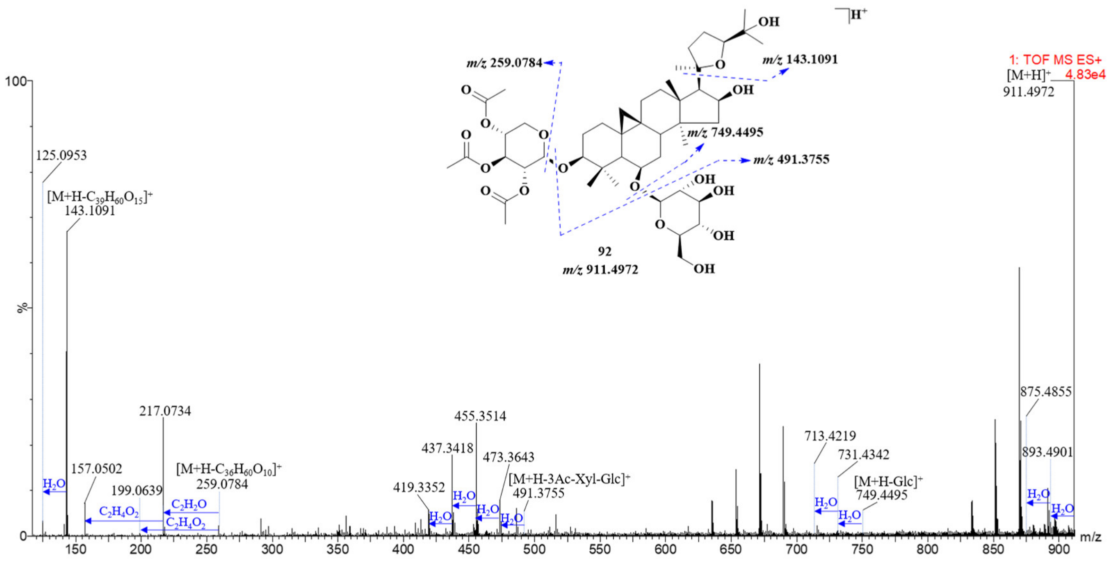

Peak **93** (t_R= 41.37min, $C_{16}H_{28}O_3$, 3-hydroxy-4,6-hexadecadienoic acid)

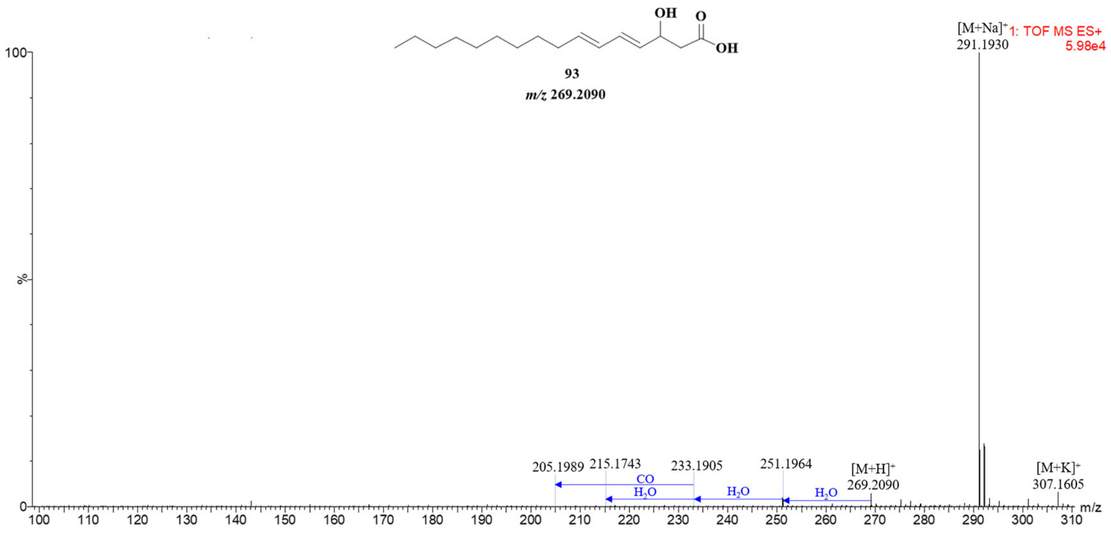

Peak **94** (t_R= 41.78min, $C_{21}H_{36}O_5$, 13-*Z*, *E*-KODE glycerol ester)

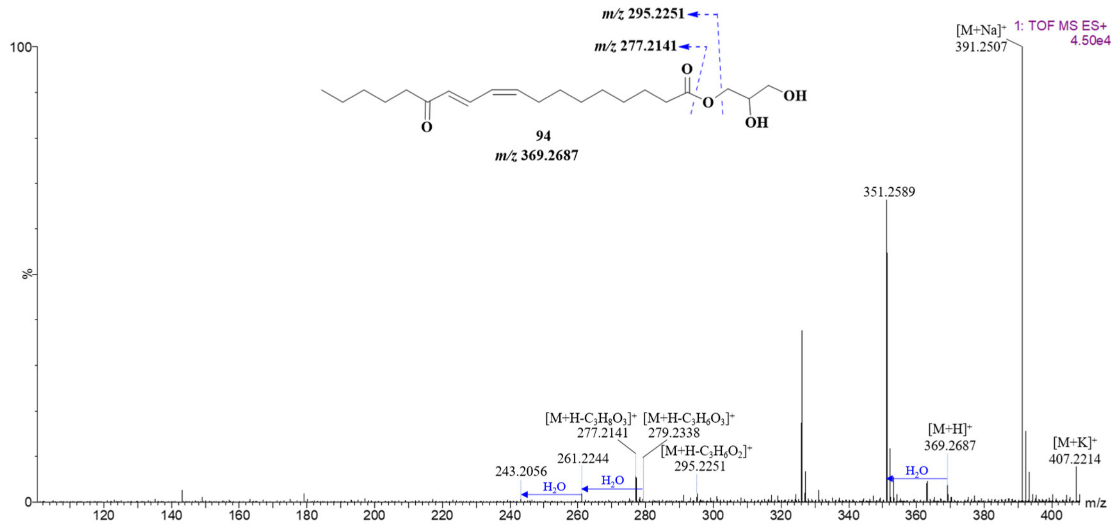

Peak **95** (t_R= 41.92min, $C_{47}H_{74}O_{17}$, Trojanoside I)

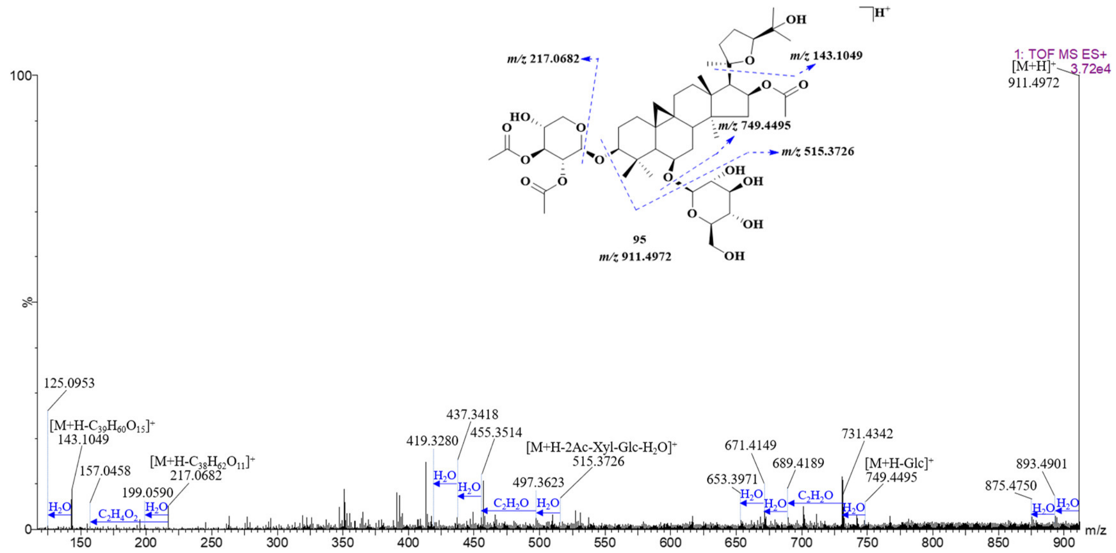

Peak **96** (t_R= 42.12min, $C_{21}H_{36}O_5$, 13-*E*, *E*-KODE glycerol ester)

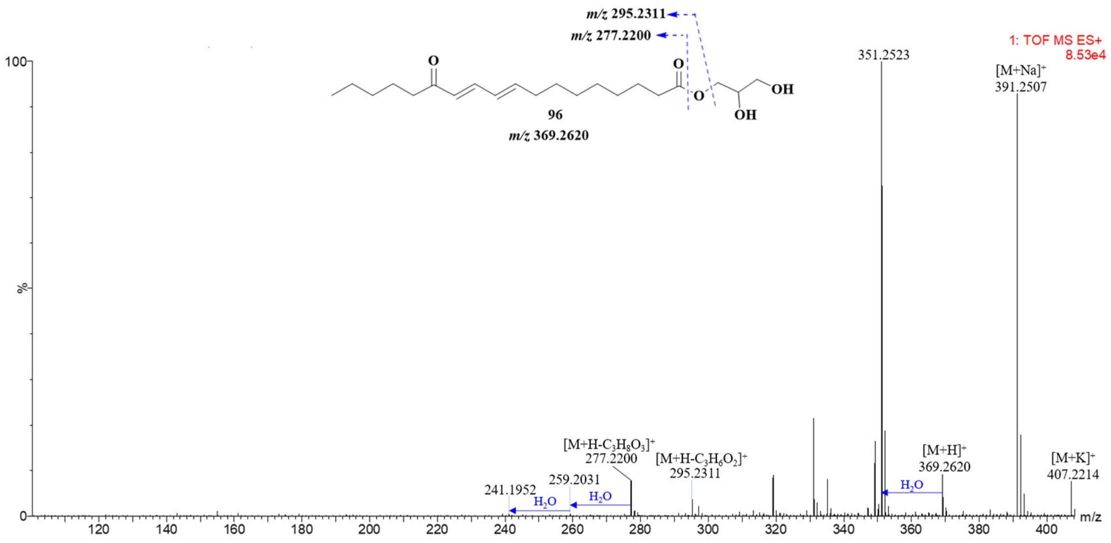

Peak **97** (t_R= 42.70min, $C_{21}H_{36}O_5$, 9-*E*, *E*-KODE glycerol ester)

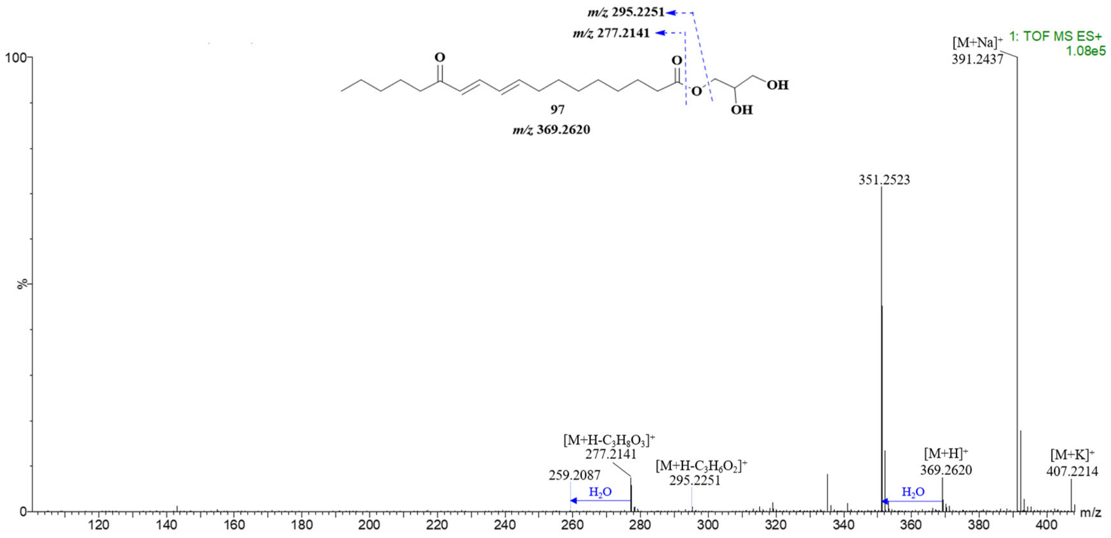

Peak **98** (t_R= 42.77min, $C_{47}H_{74}O_{17}$, Acetylastragaloside I)

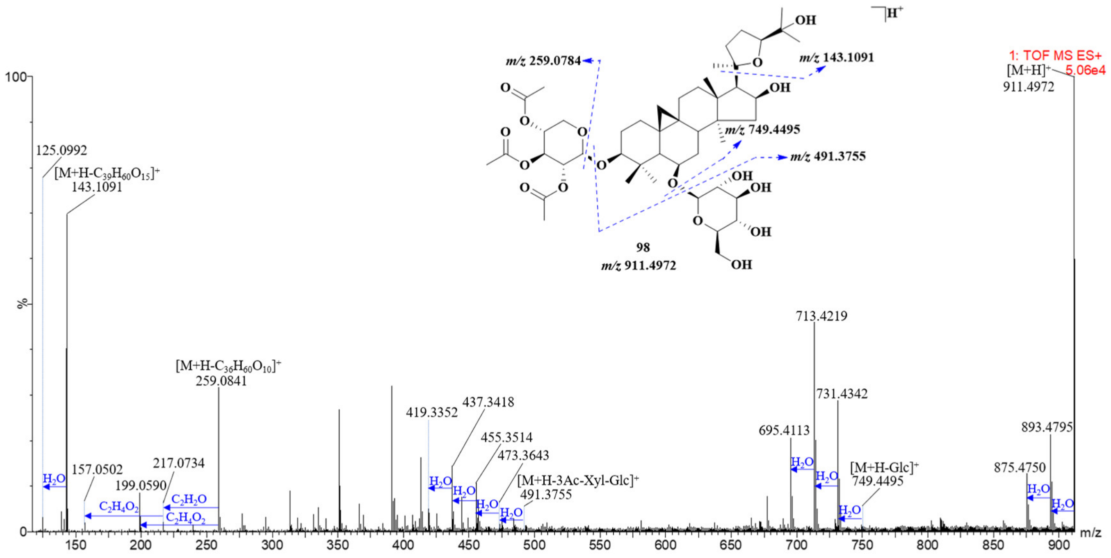

Peak **99** (t_R= 42.99min, $C_{47}H_{74}O_{17}$, Trojanoside I isomer)

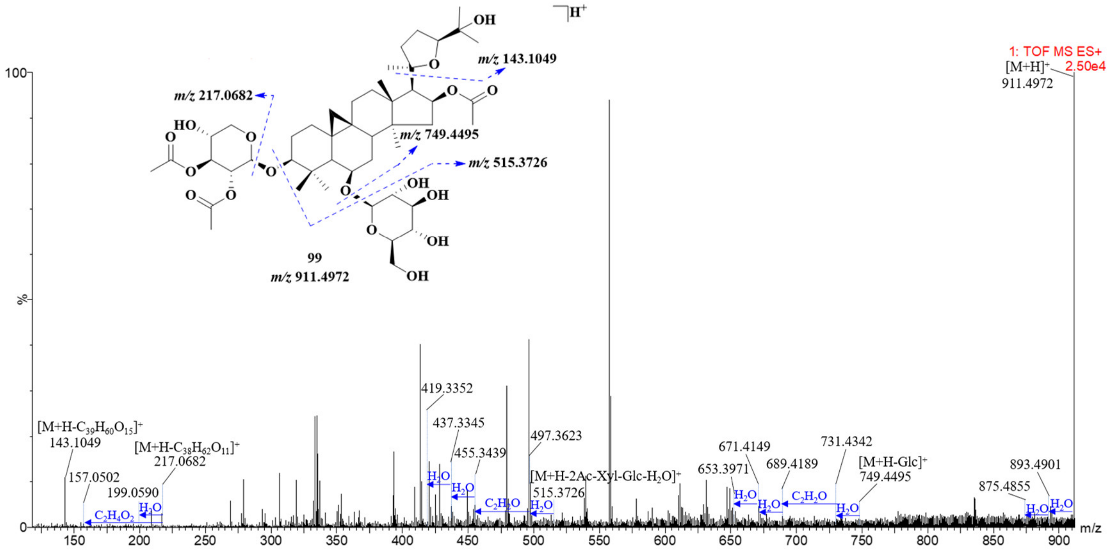

Peak **100** (t_R= 43.72min, $C_{18}H_{30}O_3$, 13-*Z*, *E*-oxooctadeca-9,11-dienoic acid)

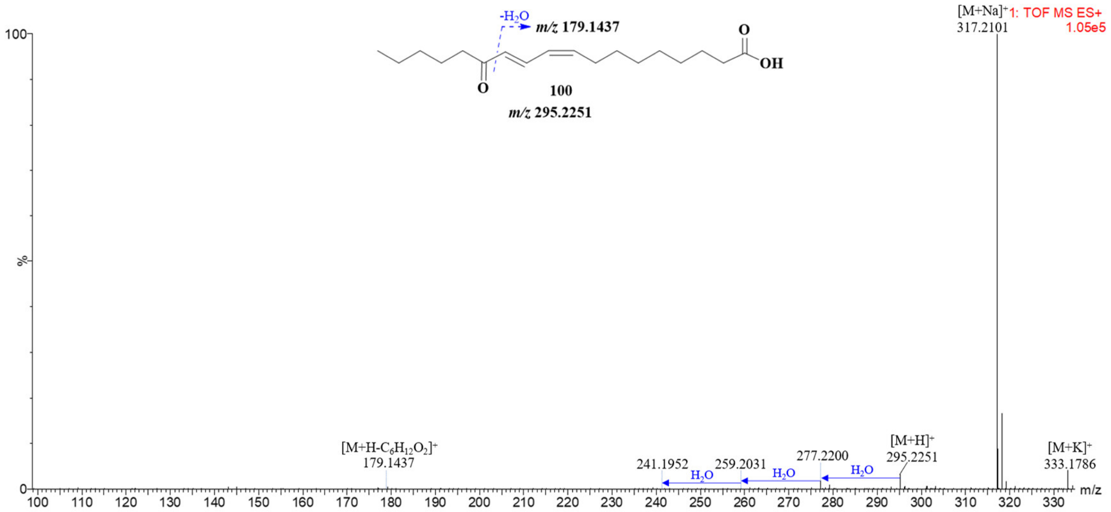

Peak **101** (t_R= 43.97min, $C_{18}H_{30}O_3$, 13-*E*, *E*-oxooctadeca-9,11-dienoic acid)

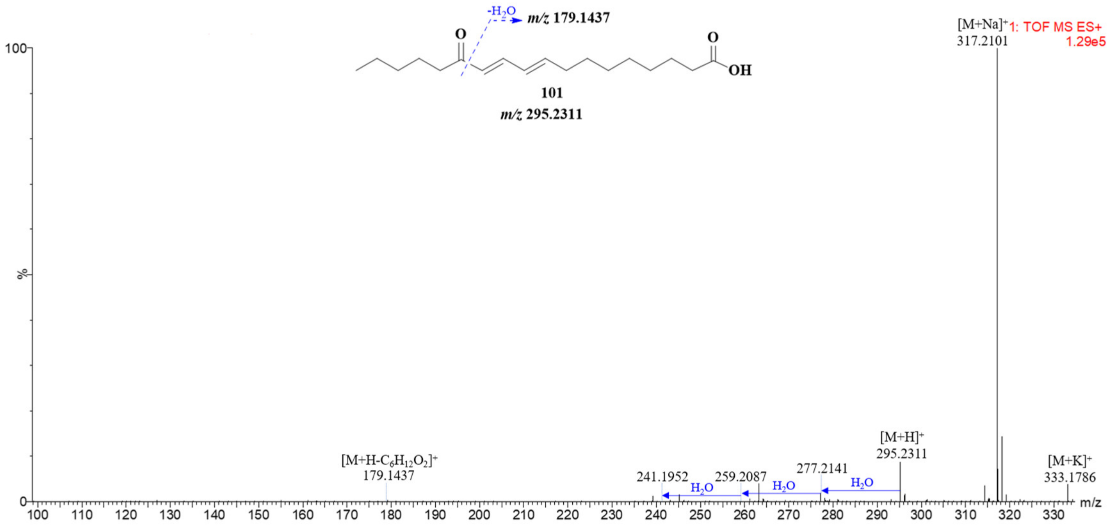

Peak **102** (t_R= 44.42min, $C_{18}H_{30}O_3$, 9-*E*, *E*-oxooctadeca-10,12-dienoic acid)

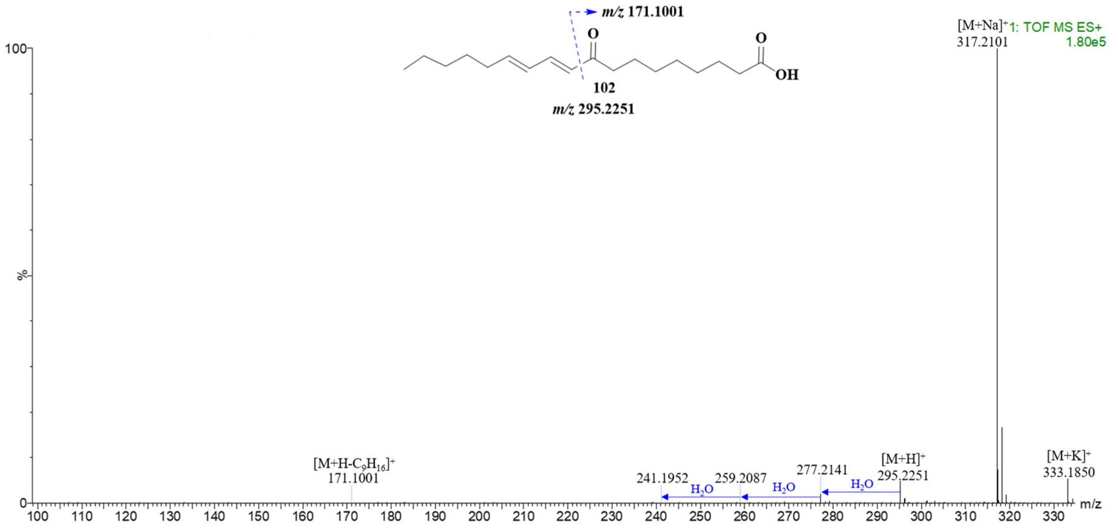

Supplement: Supplementary file 1 [file plants-15-01442-s001.zip › Supporting information_2.pdf]
